# Supplementary material for: Carbene-catalyzed double esterification enables enantioselective conformational self-locking of pillar[5]arenes
Source: Nat Commun. 2026 Mar 20;17:4253. doi: 10.1038/s41467-026-70809-1 (PMC13168476; doi:10.1038/s41467-026-70809-1)
Supplement: Supplementary file 1 — Supplementary Information [file 41467_2026_70809_MOESM1_ESM.pdf]

Carbene-Catalyzed Double Esterification Enables Enantioselective Conformational Self-Locking of Pillar[5]arenes

Vojtěch Dočekal[1], Ondřej Hladík[1], Ladislav Lóška[1], Martin Kamlar[1], Michael Franc[1], Ivana Císařová[2] & Jan Veselý[1*]*

[1] Department of Organic Chemistry, Faculty of Science, Charles University, Hlavova 2030/8, 128 00 Prague 2, Czech Republic
e-mail: vojtech.docekal@natur.cuni.cz (Vojtěch Dočekal), jan.vesely@natur.cuni.cz (Jan Veselý)

[2] Department of Inorganic Chemistry, Faculty of Science, Charles University, Hlavova 2030/8, 128 00 Prague 2, Czech Republic

Supplementary Information

Table of contents

Table of contents **2**

General **3**

Starting materials **3**

General procedure for preparing diformylpilllar[5]arenes *3*

Characterization data of starting materials *5*

Precursors of chiral NHC carbenes **9**

Organocatalytic esterification reaction **10**

Full optimization of the reaction conditions *10*

General procedure for organocatalytic esterification *15*

Product characterization data *15*

Follow-up transformations **35**

Proposed reaction mechanism **38**

Configuration stability **39**

Variation of enantiomeric purity as a function of temperature *39*

Experimental determination of the rotational barrier *39*

Crystallographic data **41**

NMR spectra **44**

Chiral HPLC **99**

References **131**

General

Chemicals and solvents were purchased from commercial suppliers and purified using standard techniques. Thin-layer chromatography (TLC) was performed on Merck 60 F_{254} silica gel plates. The compounds were visualized by irradiation with UV light and/ or by treatment with a phosphomolybdic acid (AMC) solution followed by heating. Column chromatography was performed on silica gel Fluka (40-63 µm) or SiliCycle-SiliaFlash P60 (particle size: 40-63 µm, pore diameter: 60 Å). ^{1}H, ^{13}C NMR, and ^{19}F spectra were recorded on a Bruker AVANCE III 400 NMR spectrometer. Proton chemical shifts are expressed as δ relative to tetramethylsilane (TMS) and referenced to residual protium in the NMR solvent (chloroform-*d*: δ_H = 7.26 ppm). Carbon chemical shifts are referenced to the carbon of the NMR solvent (chloroform-*d*: δ_C = 77.16 ppm). IR DRIFT spectra were recorded on a Nicolet AVATAR 370 FT-IR [1] spectrometer. Chiral HPLC was performed on a LC20AD Shimadzu liquid chromatograph with an SPD-M20A diode array detector with AD, IA, IC, and OD-H Daicel Chiralpak® columns. For chiral HPLC, the samples were prepared by dissolving them in $CHCl_3$/*i*-PrOH (1:1, *v/v*). Chloroform eluted as the first signal in all samples. Optical rotations were measured on an AU-Tomatica polarimeter, Autopol III. Specific optical rotations are expressed as concentration, *c* [g/100 ml]. The samples were prepared by dissolving them in a specific solvent for each compound. All melting points were measured on a Büchi melting point B-545 apparatus, in an open glass capillary. All values are uncorrected. The exact mass (HMRS) was measured on a Orbitrap IQ-X Tribrid mass spectrometer equipped with an electrospray ion source. The mobile phase consisted of acetonitrile at 150 µL/min flow rate. The sample was dissolved in acetonitrile and injected using a 10µL loop.

Starting materials

General procedure for preparing diformylpilllar[5]arenes

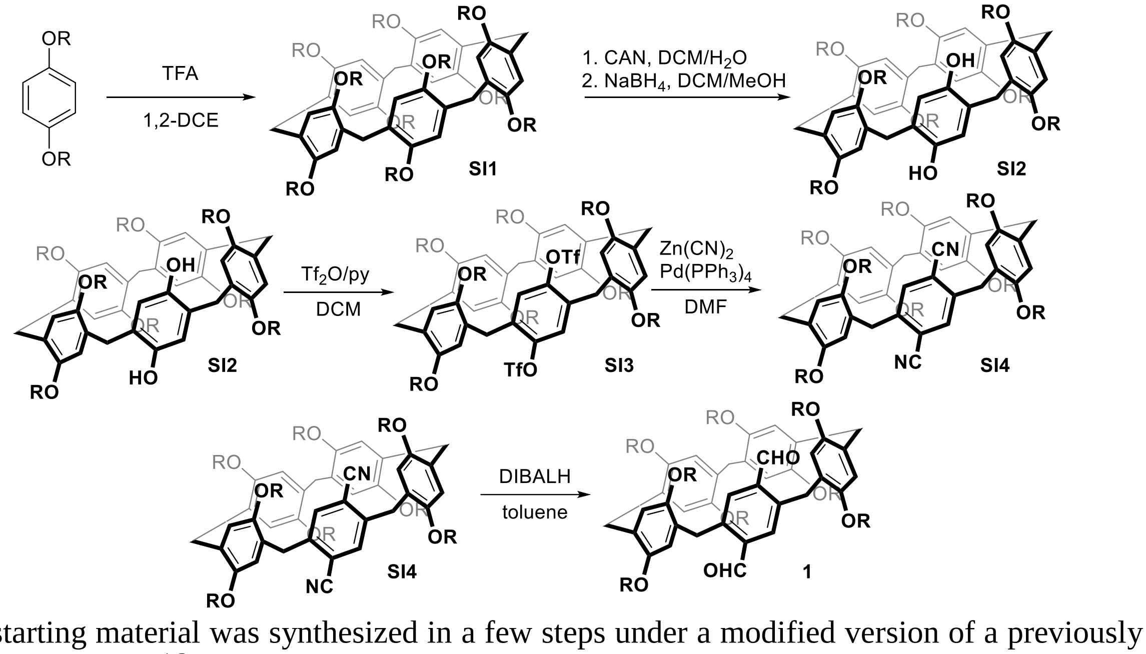

The starting material was synthesized in a few steps under a modified version of a previously reported protocol.[1,2]

Briefly, a round-bottom flask was charged with 1,4-dialkoxybenzene (1.0 equiv.), paraformaldehyde (1.0 equiv.) and suspended in 1,2-DCE (10.0M with respect to 1,4-dialkoxybenzene). Then, trifluoracetic acid (5 vol% with respect to 1,2-DCE) was added in one portion at room temperature. Subsequently, the resulting suspension was heated to reflux (oil bath) and left to stir for 2 hours at this temperature. The color turned dark green during this time. The mixture was cooled to room temperature. *Column-free work-up*: Methanol (equal

volume to 1,2-DCE) was added, and the resulting suspension was left to stir for 30 minutes at room temperature. The solids were filtered and washed with methanol. *Extractive work-up*: Water (equal volume to 1,2-DCE) was added. The organic phase was separated and washed with water (3×equal volume to 1,2-DCE), which may result in neutral pH for the last water phase; if not, addition washing is recommended. The organic phase was washed with brine (equal volume to 1,2-DCE) and dried under anhydrous $MgSO_4$. After filtration of the solid, the filtrate was concentrated under reduced pressure. The crude product was purified by column chromatography (hexane/EtOAc or hexane/DCM mixtures eluted as the least polar product).

A round-bottom flask was charged with pillar[5]arene **SI1** (1.0 equiv.) and dissolved in DCM (0.01M with respect to **SI1**) prior to adding a solution of CAN (2.0 equiv.) in water (0.001M with respect to **SI1**). The resulting biphasic reaction mixture was left to stir for 10 minutes at room temperature. *Note*: We tested various times and amounts of CAN, so we recommend using the reported time even without full conversion of the starting material. The organic phase was dried under anhydrous $MgSO_4$. After filtration of the solid, the filtrate was diluted with methanol (0.001M with respect to **SI1**), and sodium borohydride (4.0 equiv.) was added portion-wise (gas evolution) at room temperature. At this temperature, the reaction mixture was stirred for 1 hour (the reaction mixture turned colorless or yellow). The reaction was quenched by slowly adding diluted hydrochloric acid (1M, equal volume to methanol, gas evolution). The biphasic solution was diluted with water (before the formation of the biphasic solution). The organic phase was separated, and the water phase was extracted with DCM (3×10 ml, per 1.0 mmol of starting material). The organic phases were collected, washed with brine (1×10 ml, per 1.0 mmol of starting material) and dried under anhydrous $MgSO_4$. After filtration of the solid, the filtrate was concentrated under reduced pressure. The crude product was purified by column chromatography (elution with a hexane/EtOAc mixture).

A round-bottom flask was charged with **SI2** (1.0 equiv.), dissolved in anhydrous DCM (0.05M with respect to starting pillar[5]arene), before adding pyridine (13.0 equiv.). The reaction mixture was cooled to 0 °C (ice/water cooling bath). At this temperature, Tf_2O (2.1 equiv.) was added to the reaction mixture, which was slowly heated to room temperature and left to stir overnight (we observed full conversion of the starting material after 2 hours). Then, the reaction mixture was concentrated on a rotavap and dissolved in a minimal amount of DCM before adding silica gel. After DCM evaporation, the crude product on silica was directly loaded on a column and eluted with hex/EtOAc (10:1, the least polar compound).

A round-bottom flask was charged with **SI3** (1.0 equiv.), zinc cyanide (4.4 equiv.), dissolved in anhydrous DMF (0.05M with respect to the starting pillar[5]arene). The resulting mixture was degassed before adding $Pd(PPh_3)_4$ (0.2 equiv.). The reaction mixture was heated to 150 °C (oil bath) and left to stir overnight at this temperature. Then, the reaction mixture was cooled to room temperature and concentrated on a rotavap. The residue was diluted and dissolved in a water/EtOAc mixture (1/1). The organic phase was separated and washed with brine (2×10 ml, per 1.0 mmol of the starting material) and dried under anhydrous $MgSO_4$. After filtration of the solid, the filtrate was concentrated under reduced pressure. The crude product was purified by column chromatography (elution with a hexane/EtOAc mixture). *Note:* We recommend loading the crude reaction mixture directly onto a silica gel column. For a large-scale reaction, use more silica gel for loading. As an alternative, we recommend chloroform-washing the product after eluting the least polar byproduct with a hexane/EtOAc mixture.

A round-bottom flask was charged with **SI4** (1.0 equiv.) and dissolved in anhydrous toluene (0.05M with respect to pillar[5]arene). The resulting solution was cooled to -78 °C (dry ice/acetone cooling bath), followed by slow addition (for 30 minutes, using a syringe pump) of DIBALH (1.5 M in toluene, 2.5 equiv.) at this temperature *Note:* A faster DIBALH addition may cause yield deviation in large-scale synthesis. The reaction mixture was left to stir for 1 hour at -78 °C. With full conversion of the starting material, the reaction was quenched by

slowly adding EtOAc (the same volume as that of toluene) prior to adding diluted hydrochloric acid (1M, equal volume to toluene). The resulting cloudy mixture was slowly heated to room temperature and left to stir for 1 hour. Then, concentrated hydrochloric acid was slowly added to dissolved gummy residues. The organic phase was separated, and the water phase was extracted with EtOAc (3×30 ml, per 1.0 mmol of starting material). The organic phases were collected, washed with brine (1×30 ml, per 1.0 mmol of starting material) and dried under anhydrous $MgSO_4$. After filtration of the solid, the filtrate was concentrated under reduced pressure. The crude product was purified by column chromatography (elution with a hexane/EtOAc mixture).

Characterization data of starting materials

$3^2,3^5,5^2,5^5,7^2,7^5,9^2,9^5$-Octamethoxy-1,3,5,7,9(1,4)-pentabenzenacyclodecaphane-$1^2,1^5$-dicarbaldehyde (1a)

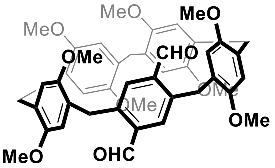

The title compound was synthesized according to the general procedure with 1,4-dimethoxybenzene (8.30 g, 60.0 mmol) using a column-free work-up, which provided **SI1a** (6.6 g, 74%), as expected. **SI1a** was used in the next steps. After a sequence of CAN oxidation and reduction to dihydroxypillar[5]arene **SI2a** (2.42 g, 38% over two step), this intermediate (**SI2a**) was used directly in the next step, which yielded **SI3a** (2.78 g, 84%) and **SI4a** (2.00 g, 96%) after cyanation. Ultimately, the title compound was obtained after reduction, which afforded **1a** (1.49 g, 74%) as an yellow amorphous solid.

^{1}H NMR (400 MHz, chloroform-*d*): δ 10.49 (s, 2H), 7.80 (s, 2H), 6.78 (s, 2H), 6.67 (s, 4H), 6.62 (s, 2H), 4.24 (s, 4H), 3.78 (s, 6H), 3.67 (d, J = 0.8 Hz, 6H), 3.64 (d, J = 0.8 Hz, 6H), 3.63 (s, 6H), 3.44 (s, 6H) ppm. ^{13}C{^{1}H} NMR (101 MHz, chloroform-*d*): δ 192.5 (2C), 151.3 (2C), 151.0 (2C), 150.9 (2C), 150.5 (2C), 141.7 (2C), 136.7 (2C), 132.6 (2C), 129.7 (2C), 128.6 (2C), 128.0 (2C), 126.4 (2C), 114.2 (2C), 114.12 (2C), 114.09 (2C), 113.6 (2C), 56.3 (2C), 56.98 (2C), 55.95 (2C), 55.2 (2C), 31.4 (2C), 30.0 (1C), 29.6 (2C) ppm. IR (ATR): ν = 1682 (C=O, aldehydes) cm^{-1}. HRMS (ESI+) m/z: calcd. for $C_{45}H_{46}O_{10}Na$ $[M + Na]^+$: 769.2983, found: 769.2985.

$3^2,3^5,5^2,5^5,7^2,7^5,9^2,9^5$-Octaethoxy-1,3,5,7,9(1,4)-pentabenzenacyclodecaphane-$1^2,1^5$-dicarbaldehyde (1b)

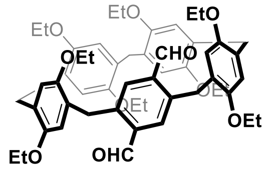

The title compound was synthesized according to the general procedure with 1,4-diethoxybenzene (5.0 g, 30.0 mmol) using an extractive work-up, which yielded **SI1b** (1.38 g, 26%), as expected. **SI1b** was used in the next steps. *Note:* Attempts at column-free work-up produced a mixture of the corresponding pillar[5]arene with pillar[6]arene (3:1, according to ^{1}H NMR). After a sequence of CAN oxidation (using 4.0 equiv. of CAN instead of 2.0 and extending the reaction time to 1 h) and reduction to **SI2b** (258 mg, 31% over two step), this intermediate (**SI2b**) was used directly in the next step, which led to **SI3b** (300 mg, 88%). Notably, **SI4b** was prepared by cyanation with a prolonged reaction time of 5 days, which did not result in full conversion. In addition to the expected intermediate, **SI4b** was isolated in 37% (100 mg). Ultimately, the title compound was obtained after DIBAL reduction, affording **1b** (77.0 mg, 90%) as a white amorphous solid.

^{1}H NMR (400 MHz, chloroform-*d*): δ 10.50 (s, 2H), 7.82 (s, 2H), 6.71 (s, 2H), 6.67 – 6.59 (m, 6H), 4.24 (s, 4H), 3.88 – 3.83 (m, 4H), 3.83 – 3.76 (m, 14H), 3.73 (t, J = 7.0 Hz, 4H), 1.27 (t, J = 6.9 Hz, 6H), 1.23 (td, J = 6.9, 2.8 Hz, 12H), 1.10 (t, J = 7.0 Hz, 6H) ppm. ^{13}C{^{1}H} NMR (101 MHz, chloroform-*d*): δ 192.5 (2C), 150.2 (2C), 150.00 (2C), 149.96 (2C), 149.4 (2C), 142.0 (2C), 136.5 (2C), 132.9 (2C), 130.1 (2C), 129.0 (2C), 128.3 (2C), 126.4 (2C), 115.3 (2C),

115.2 (2C), 114.9 (2C), 114.6 (2C), 64.4 (2C), 64.0 (4C), 63.5 (2C), 31.3 (2C), 30.0 (1C), 29.8 (2C), 15.2 (2C), 15.08 (2C), 15.05 (2C), 14.6 (2C) ppm. IR (ATR): ν = 1683 (C=O, aldehyde) cm^{-1}. HRMS (ESI+) *m*/*z*: calcd. for $C_{53}H_{62}O_{10}Na$ $[M + Na]^+$: 881.4235, found: 881.4237.

3^2,3^5,5^2,5^5,7^2,7^5,9^2,9^5-Octapropoxy-1,3,5,7,9(1,4)-pentabenzenacyclodecaphane-1^2,1^5-dicarbaldehyde

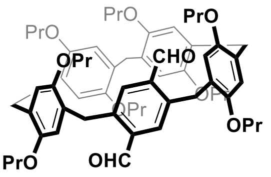

Attempts to synthesize of title compound was conducted according to the general procedure with 1,4-propoxybenzene (5.0 g, 25.7 mmol) using an extractive work-up, which yielded **SI1c** (2.0 g, 38%), **SI1c** was used in the next steps. *Note:* We observed significant formation of the corresponding pillar[6]arene. After a sequence of CAN oxidation (using 4.0 equiv. of CAN instead of 2.0 and extending the reaction time to 6 h) and reduction to **SI2c** (367 mg, 20% over two steps), this intermediate was used directly in the next step, producing **SI3c** (341 mg, 73%). However, only trace amounts of **SI4c** were observed during cyanation with a prolonged reaction time of 5 days, thus preventing the isolation of **SI4c**. To overcame this problem, we tested also deprotection of **1a** followed by alkylation. Unfortunately, we did not observe any formation of expected diformyl product under standard reaction conditions (BBr_3, DCM).

(2*E*,2'*E*)-3,3'-(3^2,3^5,5^2,5^5,7^2,7^5,9^2,9^5-octamethoxy-1,3,5,7,9(1,4)-pentabenzenacyclo-decaphane-1^2,1^5-diyl)diacrylaldehyde (1c)

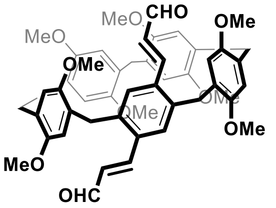

A round-bottom flask was charged with ((1,3-dioxolan-2-yl)methyl)triphenylphosphonium bromide (575 mg, 1.3 mmol, 5.0 equiv.), 18-crown-6 (aprox. 2 mg, catalytic amount) and dissolved in anhydrous THF (6 ml). Then, suspension of sodium hydride (60%wt., 64 mg, 1.6 mmol, 6.0 equiv.) was added portionwise at room temperature (decent gas evolution). At this temperature, the resulting mixture was left to stir for 20 minutes, followed by addition of pillar[5]arene **1a** (200 mg, 0.27 mmol, 1.0 equiv.). Immediately after addition, the reaction mixture was heated to 60°C (oil bath) and left to stir for 2 hours at 60 °C. With full conversion (TLC) of the starting diformyl derivative **1a**, the reaction was quenched by slow addition of wet EtOAc (5 ml + 0.5 ml of water). Followed by addition of silica gel. The residual solvents were evaporated on rotavap and crude product loaded on silica gel was directly loaded on silica gel column, the dioxolane-protected pillar[5]arene (180 mg) was eluted with hexane/EtOAc (1:1) mixture. This product was directly use in second step. Briefly, pillar[5]arene (180 mg) was dissolved in THF (6 ml), followed by addition of diluted hydrochloric acid (1M, 2.7 ml) at room temperature. The resulting reaction mixture was left to stir for 30 minutes at room temperature. With full conversion (TLC) of the starting pillar[5]arene, the reaction mixture was diluted and in a water/EtOAc mixture (30/30 ml). The organic phase was separated. The water phase was extracted with EtOAc (3×30 ml). Collected organic phases were washed with brine (1×30ml) and dried under anhydrous $MgSO_4$. After filtration of the solid, the filtrate was concentrated under reduced pressure affording **1c** (180 mg, 75% over 2 steps, pure *E* isomer) as a yellow amorphous solid.

Notes: We analyzed **1c** by chiral HPLC on columns used in this study for potential enantiomer separations (IA, IC and IG), but we detected only one peak without any separation. We were able to detect also other product isomers (*E*,*Z* or *Z*,*Z*) immediately after separation. Nevertheless, after standing in chloroform overnight resulted in an exclusive isomerization to *E*,*E*-isomer.

^{1}H NMR (400 MHz, chloroform-*d*): δ 9.71 (d, *J* = 7.8 Hz, 2H), 8.10 (d, *J* = 15.7 Hz, 2H), 7.48 (s, 2H), 6.80 (s, 2H), 6.71 (s, 2H), 6.68 (s, 2H), 6.55 – 6.42 (m, 4H), 4.30 (d, *J* = 14.1 Hz, 2H),

3.82 – 3.73 (m, 6H), 3.69-3.62 (m, 8H), 3.61 (s, 6H), 3.60 (s, 6H), 3.57 (s, 6H) ppm. $^{13}C\{^1H\}$ NMR (101 MHz, chloroform-*d*): δ 193.9 (2C), 151.3 (2C), 151.1 (2C), 150.9 (2C), 150.3 (2C), 140.1 (2C), 134.9 (2C), 129.82 (2C), 129.77 (4C), 129.5 (2C), 128.4 (2C), 127.8 (2C), 126.3 (2C), 114.33 (2C), 114.29 (2C), 114.0 (2C), 113.7 (2C), 56.2 (2C), 56.0 (2C), 55.9 (2C), 55.6 (2C), 32.3 (2C), 30.35 (1C), 29.9 (2C) ppm. IR (ATR): ν = 1672 (C=O, aldehyde) cm^{-1}. HRMS (ESI+) *m*/*z*: calcd. for $C_{49}H_{50}O_{10}Na$ $[M + Na]^+$: 821.3296, found: 821.3296.

1^5-([1,1'-Biphenyl]-4-yl)-3^2,3^5,5^2,5^5,7^2,7^5,9^2,9^5-octamethoxy-1,3,5,7,9(1,4)-pentabenzenacyclodecaphane-1^2-carbaldehyde (1d)

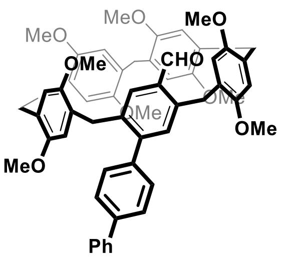

A round-bottom flask was charged with pillar[5]arene-triflate[3] (628 mg, 0.63 mmol, 1.0 equiv.) and zinc cyanide (161 mg, 1.4 mmol, 2.2 equiv.) dissolved in anhydrous DMF (12 ml). The resulting mixture was degassed before adding $Pd(PPh_3)_4$ (144 mg, 0.13 mmol, 0.2 equiv.). The reaction mixture was heated to 155 °C (oil bath) and left to stir overnight at this temperature. Then, the reaction mixture was cooled to room temperature and concentrated on a rotavap. The residue was diluted and dissolved in a water/EtOAc mixture (1/1). The organic phase was separated, washed with brine (2×5 ml, per 0.1 mmol of starting material) and dried under anhydrous $MgSO_4$. After filtration of the solid, the filtrate was concentrated under reduced pressure. The crude product (302 mg) was purified by column chromatography (elution with a hexane/EtOAc mixture).

A round-bottom flask was charged with pillar[5]arene (302 mg, 0.34 mmol, 1.0 equiv.) and dissolved in anhydrous toluene (5 ml). The resulting solution was cooled to -78 °C (dry ice/acetone cooling bath) before slowly adding (for 30 minutes, using syringe pump) DIBALH (1.5 M in toluene, 1.0 mmol, 0.7 ml, 3.0 equiv.) at this temperature. The reaction mixture was left to stir for 1 hour at -78 °C. Upon full conversion of the starting material, the reaction was quenched by slowly adding EtOAc (the same volume as toluene) before adding diluted hydrochloric acid (1M, equal volume to toluene). The resulting cloudy mixture was slowly heated to room temperature and left to stir for 1 hour. Then, concentrated hydrochloric acid was slowly added to dissolved gummy residues. The organic phase was separated, and the water phase was extracted with EtOAc (3×10 ml, per 0.1 mmol of starting material). The organic phases were collected, washed with brine (1×30 ml, per 1.0 mmol of starting material) and dried under anhydrous $MgSO_4$. After filtration of the solid, the filtrate was concentrated under reduced pressure. The crude product was purified by column chromatography (elution with hexane/EtOAc 10:1 to 3:1), affording **1d** (162 mg, 29% over 2 steps) as a white amorphous solid.

^1H NMR (400 MHz, chloroform-*d*): δ 10.36 (s, 1H), 7.84 (s, 1H), 7.72 – 7.65 (m, 2H), 7.54 (dd, *J* = 8.3, 6.8 Hz, 4H), 7.47 – 7.40 (m, 1H), 7.14 – 7.06 (m, 3H), 6.81 (s, 1H), 6.77 – 6.70 (m, 4H), 6.69 (s, 1H), 6.51 (s, 1H), 5.93 (s, 1H), 4.33 (s, 2H), 3.99 (s, 2H), 3.88 (d, *J* = 9.2 Hz, 4H), 3.77 (s, 2H), 3.67 (s, 3H), 3.66 (s, 3H), 3.66 (s, 3H), 3.63 (s, 3H), 3.61 (s, 3H), 3.54 (s, 3H), 3.38 (s, 3H), 3.32 (s, 3H) ppm. $^{13}C\{^1H\}$ NMR (101 MHz, chloroform-*d*): δ 192.8, 151.22, 151.15, 151.13, 151.09, 151.07, 150.8, 150.7 (2C), 146.7, 141.4, 140.6, 140.5, 140.0, 137.8, 133.9, 133.4, 132.4, 129.5 (2C), 129.4, 129.0 (2C), 128.73, 128.67, 128.6, 128.4, 128.2, 127.6 (2C), 127.5, 127.4, 127.0 (2C), 126.8 (2C), 114.6, 114.4 (2C), 114.11, 114.09, 114.0, 113.4, 56.2, 56.09, 56.07, 56.0 (2C), 55.6, 55.52, 55.51, 33.5, 31.5, 30.1, 30.0, 29.9 ppm. IR (ATR): ν = 1684 (C=O, aldehyde) cm^{-1}. HRMS (ESI+) *m*/*z*: calcd. for $C_{56}H_{54}O_9Na$ $[M + Na]^+$: 893.3660, found: 893.3662.

Naphthalen-2-yl 1^5-formyl-3^2,3^5,5^2,5^5,7^2,7^5,9^2,9^5-octamethoxy-1,3,5,7,9(1,4)-pentabenzenacyclodecaphane-1^2-carboxylate (1e)

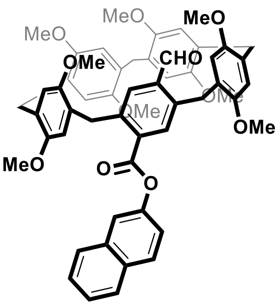

A round-bottom flask (100 ml) was charged with pillar[5]arene **1a** (650 mg, 0.87 mmol, 1.2 equiv.), naphthalen-2-ol (104.5 mg, 0.73 mmol, 1.0 equiv.), *pre*-**C1** (38.2 mg, 0.15 mmol, 0.2 equiv.), DQ (296.2 mg, 0.73 mmol, 1.0 equiv.) and Cs_2CO_3 (472.0 mg, 1.45 mmol, 2.0 equiv.) and dissolved in anhydrous DCM (40 ml). The reaction mixture was left to stir for 2 hours at room temperature. Upon full conversion of the starting material, the mixture was partly concentrated on a rotavap and directly loaded onto a column. The corresponding product was eluted with a hexane/EtOAc mixture (5:1), affording the less polar *rac*-**3a** (234 mg) and **1e** (230 mg, 35%) as a white foam.

^{1}H NMR (400 MHz, chloroform-*d*): δ 10.41 (s, 1H), 8.04 (s, 1H), 7.95 – 7.86 (m, 3H), 7.85 (s, 1H), 7.55 (ddd, J = 14.9, 6.9, 1.8 Hz, 3H), 7.30 – 7.26 (m, 1H), 6.76 (d, J = 1.3 Hz, 2H), 6.74 (d, J = 6.7 Hz, 2H), 6.70 (d, J = 2.0 Hz, 2H), 6.60 (s, 1H), 6.44 (s, 1H), 4.34 (d, J = 13.0 Hz, 4H), 3.85 (d, J = 10.2 Hz, 4H), 3.80 (s, 2H), 3.67 (s, 3H), 3.65 (s, 3H), 3.64 (s, 3H), 3.61 (s, 3H), 3.59 (s, 3H), 3.58 (s, 3H), 3.48 (s, 3H), 3.19 (s, 3H) ppm. ^{13}C{^{1}H} NMR (101 MHz, chloroform-*d*): δ 192.6, 166.6, 151.28, 151.25, 151.18, 151.16, 151.03, 151.02, 150.98, 150.7, 148.5, 141.0, 140.9, 135.5, 134.3, 134.03, 133.95, 133.9, 131.7, 129.7, 129.6, 129.4, 129.0, 128.8, 128.04, 127.97 (2C), 127.8, 127.4, 126.9, 126.7, 126.0, 121.2, 118.9, 114.5, 114.4, 114.3, 114.2, 114.1, 114.02, 113.97, 113.7, 56.2 (2C), 56.14, 56.08, 56.01, 55.97, 55.3, 55.2, 34.0, 31.7, 29.9, 29.7, 29.4 ppm. IR (ATR): ν = 1738 (C=O, ester), 1690 (C=O, aldehyde) cm^{-1}. HRMS (ESI+) *m*/*z*: calcd. for $C_{55}H_{52}O_{11}Na$ [M + Na]$^+$: 911.3402, found: 911.3403.

Precursors of chiral NHC carbenes

Precursors used in this study (Supplementary Figure 1) were purchased from commercial sources or prepared using literature procedures. Specifically, achiral *pre*-**C1** (CAS: 862893-81-0) and *pre*-**C6** (CAS: 862095-91-8) were obtained from Sigma-Aldrich (Merck) or BLDpharm; alternatively, they were synthesized according to literature procedures.[4] Similarly, *pre*-**C2** (Bode catalyst, CAS: 919102-70-8) was purchased from the same suppliers or prepared via the previously reported procedure.[5] The Rovis catalyst (*pre*-**C11**) and *pre*-**C13** were purchased from BLDpharm or prepared under previously reported condition.[6] The aminoindanol-based catalysts *pre*-**C3**,[7] *pre*-**C4**,[8] *pre*-**C7**,[9] *pre*-**C8**,[10] *pre*-**C9**,[7] *pre*-**C10**,[6] *pre*-**C12**,[11] and *pre*-**C14**[12] were synthesized according to established protocols. The following precursors were purchased from Sigma-Aldrich (Merck): *pre*-**C5** (CAS: 950842-71-4) and *pre*-**C18** (CAS: 925706-40-7), while *pre*-**C20** (CAS: 862095-77-0) was purchased from BLDpharm. All other catalysts were prepared utilizing previously reported procedures, including *pre*-**C15-16**,[13] *pre*-**C17**,[14] *pre*-**C19**,[15] *pre*-**C21-23**,[16] *pre*-**C24**,[17] *pre*-**C25**,[18] and *pre*-**C26**.[19]

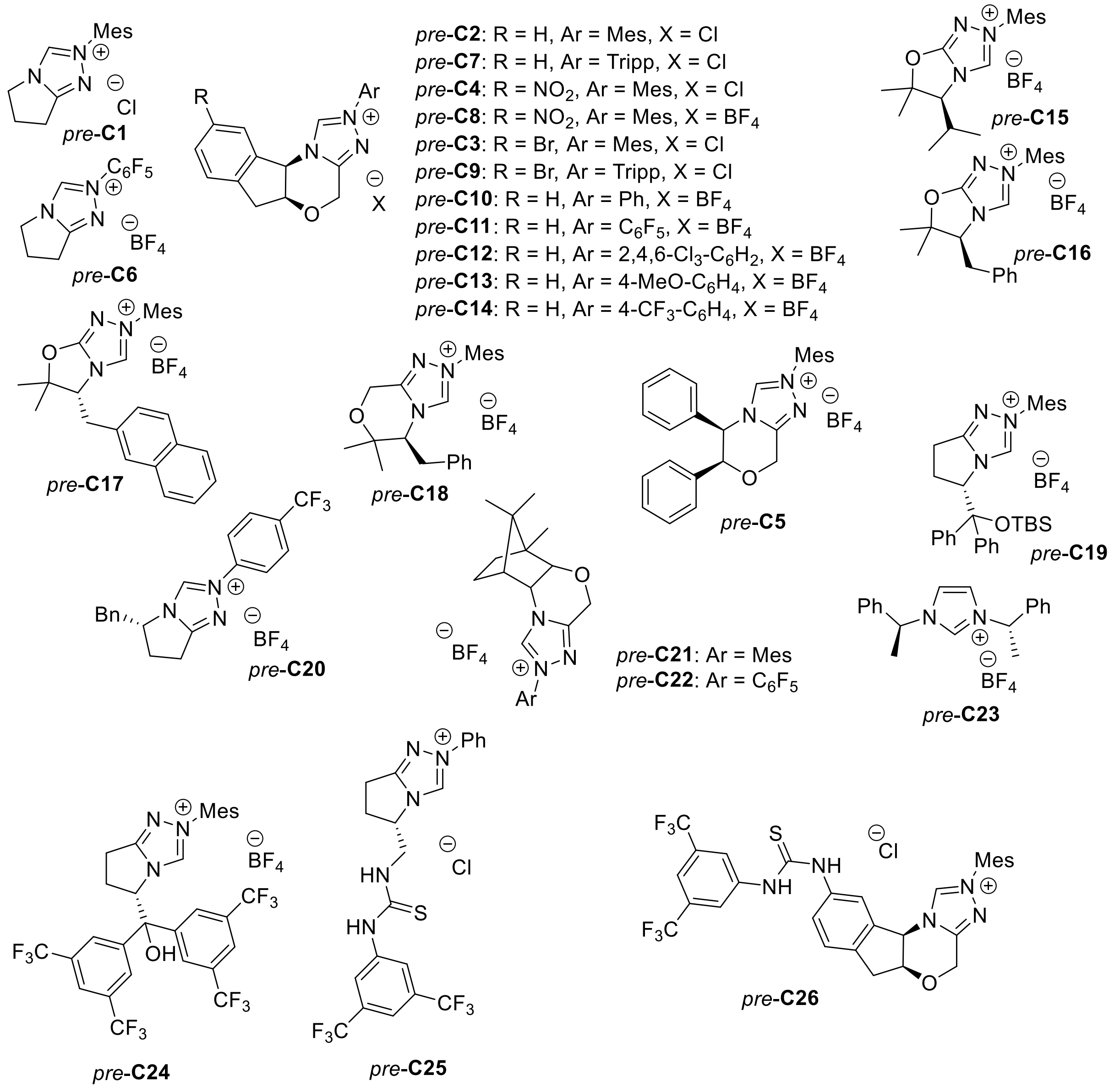

Supplementary Figure 1. Screened precursors

Organocatalytic esterification reaction

Full optimization of the reaction conditions

Supplementary Table 1. Precursor screening

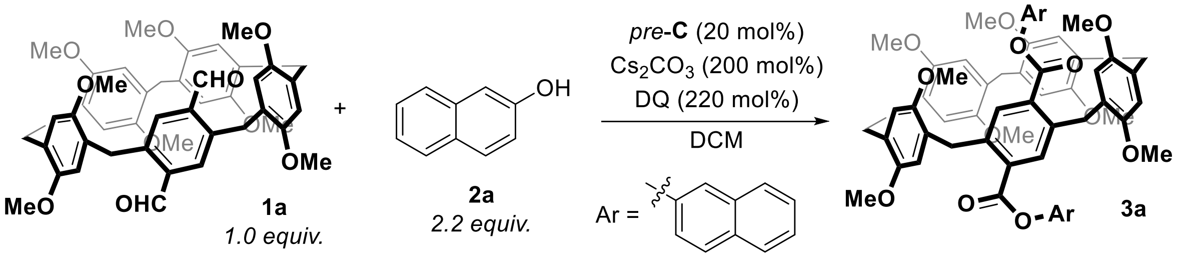

Entry	*pre*-NHC	Time (h)	Conversion	Yield (%)[a]	*er*[b]
1	*pre*-**C1**	2	full	68	50:50
2	*pre*-**C2**	2	full	69	75:25
3	*pre*-**C7**	24	full	73	83:17
4	*pre*-**C4**	3	full	59	88:12
5	*pre*-**C8**	18	full	60	88:12
6	*pre*-**C3**	2	full	85	85:15
7	*pre*-**C9**	42	full	43	85:15
8	*pre*-**C10**	18	full	49	29:71
9	*pre*-**C11**	24	full	57	79:21
10	*pre*-**C12**	1	full	86	86:14
11	*pre*-**C13**	72	not full	traces	-
12	*pre*-**C14**	24	full	40	22:78
13	*pre*-**C15**	72	not full	traces	-
14	*pre*-**C16**	72	not full	8	23:77
15	*pre*-**C17**	72	no	-	-
16	*pre*-**C18**	18	full	34	22:78
17	*pre*-**C5**	2	full	69	86:14
18	*pre*-**C19**	72	no	-	-
19	*pre*-**C20**	72	not full	traces	-
20	*pre*-**C21**	72	not full	22	77:23
21	*pre*-**C22**	72	not full	traces	-
22	*pre*-**C23**	72	no	-	-
23	*pre*-**C24**	24	full	55	88:12
24	*pre*-**C25**	72	no	-	-
25	*pre*-**C26**	72	not full	traces	-

[a] Isolated after column chromatography. [b] Determined by chiral HPLC.

Supplementary Table 2. Base screening

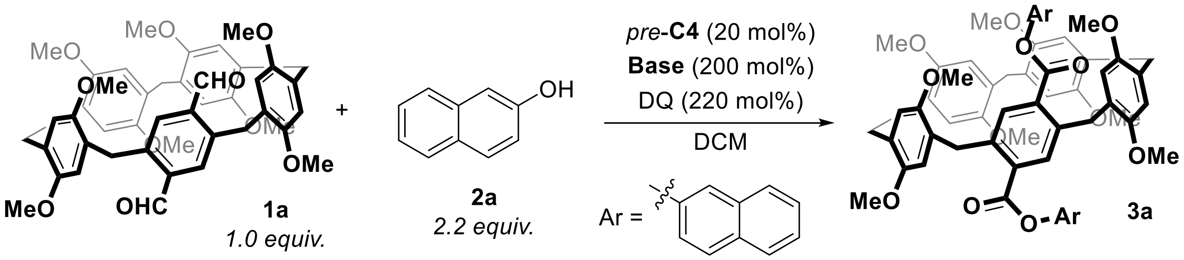

Entry	Base	Time (h)	Conversion	Yield (%)[a]	*er*[b]
1	Cs_2CO_3	3	full	59	88:12
2	Rb_2CO_3	24	full	78	88:12
3	K_2CO_3	72	full	20	92:8
4	Na_2CO_3	24	full	78	90:10
5	$NaHCO_3$	48	full	86	91:9
6	KO*t*Bu	2	full	43	93:7
7	NaOMe	24	full	80	89:11, 99.5:0.5[c]
8	NaO*t*Bu	72	not full	traces	-
9	AcONa	48	full	85	93:7, >99.5:0.5[c]
10	AcOK	48	full	78	93:7
11	$PhCO_2Na$	48	almost full	23	95:5
12	TEA	2	almost full	31	88:12
13	DIPEA	24	full	60	89:11
14	DABCO	72	full	42	91:9
15	DBU	2	full	55	84:16
16	pyridine	72	no	-	-
17	2,6-lutidine	72	no	-	-

[a] Isolated after column chromatography. [b] Determined by chiral HPLC. [c] After crystallization (from filtrate).

Supplementary Table 3. Solvent screening

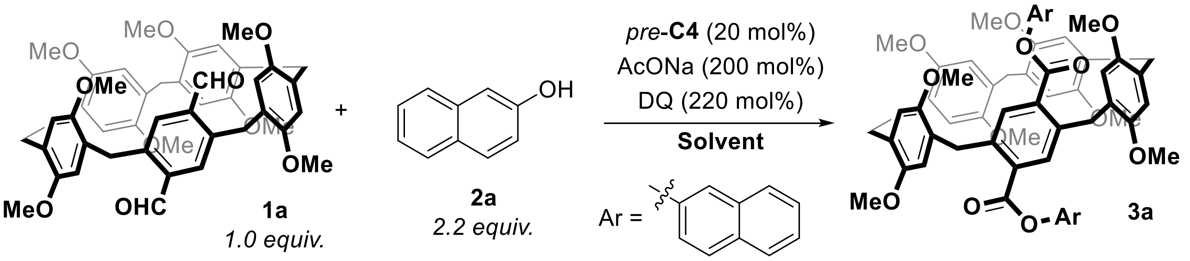

Entry	Solvent	Time (h)	Conversion	Yield (%)[a]	*er*[b]
1	DCM	48	full	85	93:7
2	$CHCl_3$	72	full	43	87:13
3	1,2-DCE	72	full	14	92:8
4	CCl_4	72	full	47	82:18
5	benzene	24	full	66	83:17
6	toluene	72	full	52	86:14
7	MeCN	72	full	23	86:14
8	EtOAc	72	full	98	90:10
9	MTBE	3	full	76	77:23
10	THF	1	full	98	90:10
11	DMSO	1	full	51	81:19
12	heptane	72	no	-	-

[a] Isolated after column chromatography. [b] Determined by chiral HPLC.

Supplementary Table 4. Oxidant screening

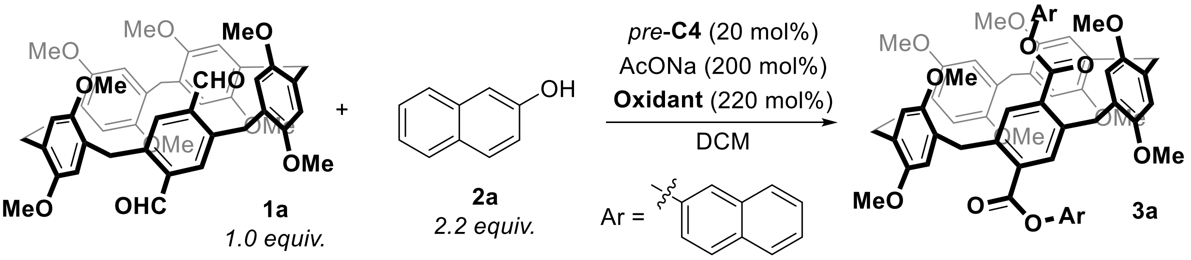

Entry	Oxidant	Time (h)	Conversion	Yield (%)[a]	*er*[b]
1	DQ	48	full	85	93:7
2	DDQ	72	no	-	-
3	TEMPO	72	full	12	90:10
4	acridine	72	no	-	-
5	nitrobenzene	72	no	-	-
6	MnO_2	72	no	-	-
7	CAN	72	no	-	-

[a] Isolated after column chromatography. [b] Determined by chiral HPLC.

Supplementary Table 5. Additive screening

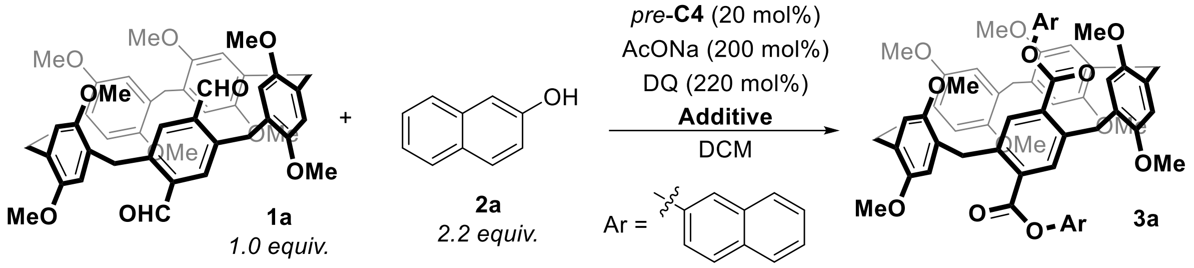

Entry	Additive	Time (h)	Conversion	Yield (%)[a]	*er*[b]
1	none	48	full	85	93:7
2	H_2O (100 mol%)	72	not full	30	91:9
3	MS (50mg, 3A)	72	not full	49	93:7
4	LiCl (50 mol%)	72	not full	65	93:7
5	(*R*,*R*)-TUC (20 mol%)	48	full	89	94:6
6	Schreiner THU (20 mol%)	72	not full	6	95:5

[a] Isolated after column chromatography. [b] Determined by chiral HPLC. (*R*,*R*)-TUC = 1-[3,5-Bis(trifluoromethyl)phenyl]-3-[(1*R*,2*R*)-(−)-2-(dimethylamino)cyclohexyl]thiourea, CAS No.: 620960-26-1, Schreiner THU = N,N′−bis[3,5−bis(trifluoromethyl)phenyl]−thiourea, CAS No.: 1060-92-0.

Supplementary Table 6. Catalyst, base and oxidant loading screening

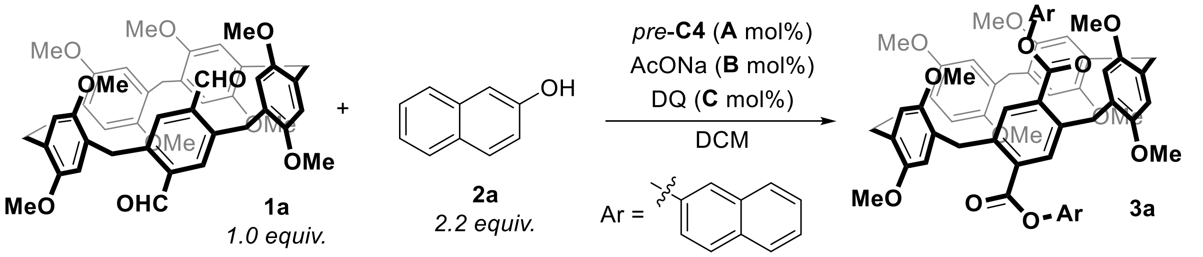

Entry	A	B	C	Time (h)	Conversion	Yield (%)[a]	*er*[b]
1	20	200	220	48	full	85	93:7
2	25	200	220	48	full	51	93:7
3	10	200	220	72	not full	1	93:7
4	5	200	220	72	not full	traces	-
5	1	200	220	72	not full	traces	-
6	20	250	220	72	not full	31	93:7
7	20	150	220	72	not full	21	92:8
8	20	100	220	72	not full	18	92:8
9	20	50	220	72	not full	28	92:8
10[c]	20	200	250	72	not full	38	93:7
11[c]	20	200	220	72	not full	33	93:7
12[c]	20	200	200	72	not full	23	93:7

[a] Isolated after column chromatography. [b] Determined by chiral HPLC. [c] Under inert atmosphere.

Supplementary Table 7. Concentration and temperature screening

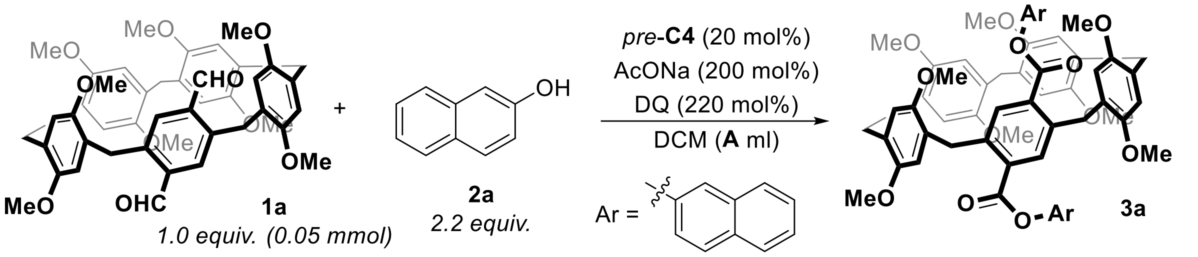

Entry	A	B	Temperature	Time (h)	Conversion	Yield (%)[a]	er[b]
1	2.2	1.0	r.t. (~30°C)	48	full	85	93:7
2	2.5	1.0	r.t (~21°C)	72	not full	19	93:7
3	2.1	1.0	r.t. (~21°C)	72	not full	25	93:7
4	2.2	2.0	r.t. (~21°C)	72	not full	29	92:8
5	2.2	0.5	r.t (~21°C)	72	not full	8	94:5
6	2.2	1.0	60°C	4	full	83	90:10
7	2.2	1.0	40°C	18	full	82	92:8
8	2.2	1.0	0°C	72	no	-	-

[a] Isolated after column chromatography. [b] Determined by chiral HPLC.

Supplementary Table 8. Control experiments

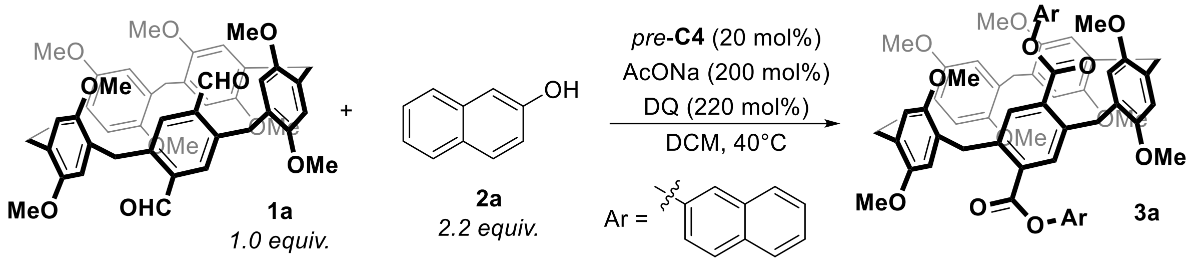

Entry	Deviation from conditions	Time (h)	Conversion	Yield (%)[a]	er[b]
1	none	18	full	82	92:8
2	AcONa (200 mol%), **THF**, 0°C	24	full	28	91:9
3	**Cs_2CO_3** (200 mol%), **THF**, r.t.	1	full	75	84:16
4	**KOtBu** (200 mol%), **THF**, r.t.	1	full	35	87:13

[a] Isolated after column chromatography. [b] Determined by chiral HPLC.

General procedure for organocatalytic esterification

General procedure A: esterification of dialdehydes

The vial (4 ml) was charged with **1** (0.05 mmol, 1.0 equiv.), the corresponding alcohol **2** (0.11 mmol, 2.2 equiv.), *pre*-**C4** (4.1 mg, 0.01 mmol, 0.2 equiv.), DQ (45.0 mg, 0.11 mmol, 2.2 equiv.) and AcONa (8.2 mg, 0.10 mmol, 2.0 equiv.) and dissolved in DCM (1.0 ml) at room temperature (~20 °C). The reaction mixture was heated to 40 °C (heating block), and the reaction mixture was stirred for the indicated time. Once the starting pillar[5]arene or the corresponding monoester was no longer detected by thin-layer chromatography (TLC), the reaction mixture was directly loaded into a silica gel column for chromatography, and the product was eluted with hexane/EtOAc mixtures.

Note: Racemic samples were prepared in reactions with *pre*-**C1** at room temperature.

General procedure B: esterification of monoaldehydes

The vial (4 ml) was charged with **1** (0.05 mmol, 1.0 equiv.), the corresponding alcohol **2** (0.06 mmol, 1.2 equiv.), *pre*-**C4** (4.1 mg, 0.01 mmol, 0.2 equiv.), DQ (24.5 mg, 0.06 mmol, 1.2 equiv.) and AcONa (8.2 mg, 0.10 mmol, 2.0 equiv.) and dissolved in DCM (1.0 ml) at room temperature (~20 °C). The reaction mixture was heated to 40 °C (heating block), and the reaction mixture was stirred for the indicated time. Once aldehyde **1** was no longer detected by TLC, the reaction mixture was directly loaded into a silica gel column for chromatography, and the product was eluted with hexane/EtOAc mixtures.

Note: Racemic samples were prepared in reactions with *pre*-**C1** at room temperature.

Product characterization data

Di(naphthalen-2-yl) (*pS*)-3^2,3^5,5^2,5^5,7^2,7^5,9^2,9^5-octamethoxy-1,3,5,7,9(1,4)-pentabenzenacyclodecaphane-1^2,1^5-dicarboxylate (3a)

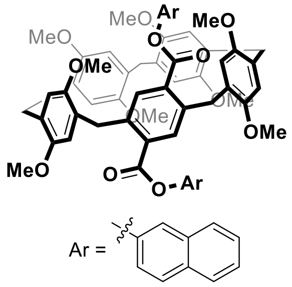

The title compound was synthesized according to the general procedure A (reaction time: 18 h), using pillar[5]arene **1a** (37.3 mg, 0.05 mmol) and naphthalen-2-ol (15.9 mg, 0.11 mmol). The crude product was purified by column chromatography (hexane/EtOAc – 5:1), affording **3a** (42.3 mg, 82%) as a white foam.

The title compound was synthesized according to the general procedure B (reaction time: 18 h), using pillar[5]arene **1d** (44.5 mg, 0.05 mmol) and naphthalen-2-ol (8.7 mg, 0.06 mmol). The crude product was purified by column chromatography (hexane/EtOAc – 5:1), affording **3a** (45.4 mg, 88%) as a white foam.

Notes: We recommend loading the crude reaction mixture directly onto a silica gel column. Dry loading may lead to inconsistent results because the product may crystallize on the column. For the same reason, we recommend using the smallest column possible and minimizing the time that the sample remains on the column. To facilitate naphthol identification by TLC, we recommend using an AMC stain, wherein naphthol produces an intense a blue spot, while the product appears as a less intense blue-to-green spot.

Single crystals of **3a** suitable for X-ray analysis were obtained by dissolving the compound (40 mg) in a minimal amount of chloroform (0.2 mL), followed by the addition of *i*PrOH (0.3 mL). The solution was allowed to crystallize at room temperature overnight. The resulting crystals were analyzed by X-ray diffraction (CCDC: 2498606). Subsequent HPLC analysis of the mother liquor revealed that the remaining material was enantiopure (see Chiral HPLC section). To determine the absolute configuration, single crystals of this enantiopure material were grown by dissolving the amorphous solid from the mother liquor (10 mg) in chloroform

(0.2 mL) followed by the addition of *i*PrOH (0.3 mL). Slow crystallization at room temperature over 48 hours yielded crystals suitable for X-ray analysis (CCDC: 2498607)
Er = 92:8 (*ee* = 84%), the enantiomeric excess of product **3a** was determined by HPLC on a Chiralpak® IC column (*n*-heptane/*i*-PrOH – 70:30, flow rate = 1.0 ml/min, λ = 190 nm, *t* = 25 °C): t_R = 9.3 min (major), t_R = 12.8 min (minor). $[\alpha]_D^{20}$ = +5.3 (*c* = 1.7, $CHCl_3$). ^{1}H NMR (400 MHz, chloroform-*d*): δ 8.09 (s, 2H), 7.95 – 7.88 (m, 6H), 7.59 – 7.52 (m, 4H), 7.52 – 7.49 (m, 2H), 7.24 (dd, *J* = 8.8, 2.3 Hz, 2H), 6.74 (d, *J* = 1.0 Hz, 4H), 6.66 (s, 2H), 6.33 (s, 2H), 4.67 (d, *J* = 13.6 Hz, 2H), 4.03 – 3.96 (m, 4H), 3.88 (s, 2H), 3.73 (d, *J* = 14.4 Hz, 2H), 3.68 (s, 6H), 3.51 (s, 6H), 3.47 (s, 6H), 3.15 (s, 6H) ppm. ^{13}C{^{1}H} NMR (101 MHz, chloroform-*d*): δ 166.6 (2C), 151.5 (2C), 151.3 (2C), 151.1 (2C), 150.9 (2C), 148.6 (2C), 140.3 (2C), 134.5 (2C), 134.0 (2C), 132.2 (2C), 131.7 (2C), 129.6 (2C), 129.4 (2C), 129.3 (2C), 128.0 (2C), 127.9 (2C), 127.8 (2C), 127.4 (2C), 126.9 (2C), 126.0 (2C), 121.4 (2C), 119.0 (2C), 114.8 (2C), 114.3 (2C), 114.1 (2C), 113.7 (2C), 56.3 (2C), 56.01 (2C), 55.98 (2C), 55.2 (2C), 34.2 (2C), 29.8 (2C), 29.0 (1C) ppm. IR (ATR): ν = 1736 (C=O, esters) cm^{-1}. HRMS (ESI+) *m*/*z*: calcd. $C_{65}H_{58}O_{12}Na$ $[M + Na]^+$: 1053.3821, found: 1053.3823.

Di(naphthalen-2-yl) (*pR*)-3^2,3^5,5^2,5^5,7^2,7^5,9^2,9^5-octamethoxy-1,3,5,7,9(1,4)-pentabenzenacyclodecaphane-1^2,1^5-dicarboxylate (*ent*-3a)

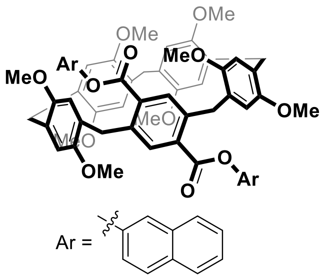

The title compound was synthesized according to the modified general procedure A (reaction time: 18 h), using pillar[5]arene **1a** (37.3 mg, 0.05 mmol), naphthalen-2-ol (15.9 mg, 0.11 mmol) and *ent*-*pre*-**C4** instead of *pre*-**C4**. The crude product was purified by column chromatography (hexane/EtOAc – 5:1), affording *ent*-**3a** (43.3 mg, 84%) as a white foam.
Er = 92:8 (*ee* = 84%), the enantiomeric excess of product **3a** was determined by HPLC on a Chiralpak® IC column (*n*-heptane/*i*-PrOH – 70:30, flow rate = 1.0 ml/min, λ = 190 nm, *t* = 25 °C): t_R = 9.3 min (minor), t_R = 12.8 min (major). $[\alpha]_D^{20}$ = -5.3 (*c* = 1.3, $CHCl_3$). Other analytical data agree with the data on the opposite enantiomer (**3a**).

Di(naphthalen-2-yl) (*pS*)-3^2,3^5,5^2,5^5,7^2,7^5,9^2,9^5-octaethoxy-1,3,5,7,9(1,4)-pentabenzenacyclodecaphane-1^2,1^5-dicarboxylate (3b)

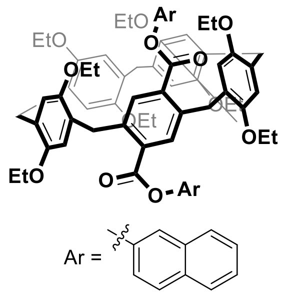

The title compound was synthesized according to the general procedure A (reaction time: 18 h), using pillar[5]arene **1b** (43.0 mg, 0.05 mmol) and naphthalen-2-ol (15.9 mg, 0.11 mmol). The crude product was purified by column chromatography (hexane/EtOAc – 8:1), affording **3b** (57.2 mg, 96%) as a white foam.
Er = 84:16 (*ee* = 68%), the enantiomeric excess of product **3b** was determined by HPLC on a Chiralpak® IC column (*n*-heptane/*i*-PrOH – 70:30, flow rate = 1.0 ml/min, λ = 190 nm, *t* = 25 °C): t_R = 4.6 min (major), t_R = 6.5 min (minor). $[\alpha]_D^{20}$ = -22.0 (*c* = 1.6, $CHCl_3$). ^{1}H NMR (400 MHz, chloroform-*d*): δ 8.17 (s, 2H), 8.00 – 7.86 (m, 6H), 7.63 – 7.49 (m, 6H), 7.22 (dd, *J* = 8.8, 2.3 Hz, 2H), 6.79 (s, 2H), 6.75 (s, 2H), 6.69 (s, 2H), 6.57 (s, 2H), 4.65 (d, *J* = 13.6 Hz, 2H), 4.11 (d, *J* = 13.6 Hz, 2H), 3.91 – 3.59 (m, 22H), 1.25 (td, *J* = 6.9, 3.1 Hz, 12H), 1.10 (t, *J* = 6.9 Hz, 6H), 0.99 (t, *J* = 6.9 Hz, 6H) ppm. ^{13}C{^{1}H} NMR (101 MHz, chloroform-*d*): δ 166.6 (2C), 150.2 (2C), 150.1 (2C), 149.98 (2C), 149.96 (2C), 148.6 (2C), 141.1 (2C), 134.4 (2C), 134.0 (2C), 131.9 (2C), 131.7 (2C), 129.7 (2C), 129.6 (2C), 129.1 (2C), 128.5 (2C), 128.0 (2C), 127.9 (2C), 127.3 (2C), 126.8 (2C), 126.0 (2C), 121.4 (2C), 119.0 (2C), 115.5 (2C), 115.4 (2C), 115.3 (2C), 114.8 (2C), 64.4 (2C), 64.2 (2C), 64.0 (2C), 63.3 (2C), 33.3 (2C), 29.9 (1C),

29.8 (2C), 15.8 (2C), 15.15 (2C), 15.0 (2C), 14.5 (2C) ppm. IR (ATR): ν = 1734 (C=O, esters) cm^{-1}. HRMS (ESI+) *m*/*z*: calcd. for $C_{73}H_{74}O_{12}Na$ $[M + Na]^+$: 1165.5073, found: 1165.5076.

Di(naphthalen-2-yl) (*pS*)-3,3'-($3^2,3^5,5^2,5^5,7^2,7^5,9^2,9^5$-octamethoxy-1,3,5,7,9(1,4)-pentabenzenacyclodecaphane-$1^2,1^5$-diyl)(2*E*,2'*E*)-diacrylate (3c)

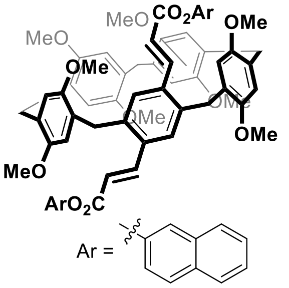

The title compound was synthesized according to the general procedure A (reaction time: 2 h), using pillar[5]arene **1c** (39.9 mg, 0.05 mmol) and naphthalen-2-ol (15.9 mg, 0.11 mmol). The crude product was purified by column chromatography (hexane/EtOAc – 9:1), affording **3c** (11.4 mg, 21%) as a white foam.

Note: The racemic sample was prepared in reaction conducted in presence of *rac*-*pre*-**C4** instead of *pre*-**C1.**

Er = 53:47 (*ee* = 5%), the enantiomeric excess of product **3c** was determined by HPLC on a Chiralpak® IC column (*n*-heptane/*i*-PrOH – 90:10, flow rate = 1.0 ml/min, λ = 222 nm, *t* = 25 °C): t_R = 23.0 min (major), t_R = 30.8 min (minor). $[\alpha]_D^{20}$ ~ 0 (*c* = 1.0, $CHCl_3$). ^1H NMR (400 MHz, chloroform-*d*): δ 8.52 (d, *J* = 15.8 Hz, 2H), 8.00 – 7.80 (m, 6H), 7.66 (d, *J* = 2.3 Hz, 2H), 7.63 (s, 2H), 7.58 – 7.47 (m, 4H), 7.32 (dd, *J* = 8.8, 2.4 Hz, 2H), 6.78 (s, 2H), 6.69 (s, 2H), 6.67 (s, 2H), 6.65 (s, 2H), 6.49 (d, *J* = 15.8 Hz, 2H), 4.23 (d, *J* = 14.0 Hz, 2H), 3.90 – 3.76 (m, 8H), 3.64 (s, 6H), 3.63 (s, 6H), 3.61 (s, 6H), 3.56 (s, 6H) ppm. $^{13}C\{^1H\}$ NMR (101 MHz, chloroform-*d*): δ 165.6 (2C), 151.2 (2C), 151.1 (4C), 150.8 (2C), 148.7 (2C), 145.1 (2C), 139.8 (2C), 134.7 (2C), 134.0 (2C), 131.6 (2C), 129.64 (2C), 129.61 (2C), 129.5 (2C), 128.6 (2C), 128.2 (2C), 128.0 (2C), 127.8 (2C), 126.8 (4C), 125.9 (2C), 121.3 (2C), 118.7 (2C), 118.4 (2C), 114.4 (2C), 114.3 (4C), 113.7 (2C), 56.3 (2C), 56.11 (2C), 56.05 (2C), 55.7 (2C), 32.9 (2C), 30.3 (1C), 29.9 (2C) ppm. IR (ATR): ν = 1722 (C=O, esters) cm^{-1}. HRMS (ESI+) *m*/*z*: calcd. for $C_{69}H_{62}O_{12}Na$ $[M + Na]^+$: 1105.4133, found: 1105.4136.

Di(anthracen-2-yl) (*pS*)-$3^2,3^5,5^2,5^5,7^2,7^5,9^2,9^5$-octamethoxy-1,3,5,7,9(1,4)-pentabenzenacyclodecaphane-$1^2,1^5$-dicarboxylate (3d)

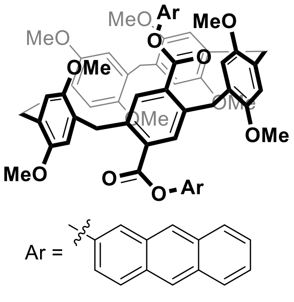

The title compound was synthesized according to the general procedure A (reaction time: 48 h), using pillar[5]arene **1a** (37.3 mg, 0.05 mmol) and anthracen-2-ol (21.4 mg, 0.11 mmol). The crude product was purified by column chromatography (hexane/EtOAc – 10:1-3:1), affording **3d** (45.2 mg, 80%) as a white foam.

Er = 90:10 (*ee* = 80%), the enantiomeric excess of product **3d** was determined by HPLC on a Chiralpak® IC column (*n*-heptane/*i*-PrOH – 70:30, flow rate = 1.0 ml/min, λ = 190 nm, *t* = 25 °C): t_R = 16.3 min (major), t_R = 25.3 min (minor). $[\alpha]_D^{20}$ = -18.9 (*c* = 2.1, $CHCl_3$). ^1H NMR (400 MHz, chloroform-*d*): δ 8.54 – 8.45 (m, 4H), 8.14 – 8.01 (m, 8H), 7.66 (dd, *J* = 2.3, 0.8 Hz, 2H), 7.57 – 7.47 (m, 4H), 7.23 (dd, *J* = 9.1, 2.3 Hz, 2H), 6.77 (d, *J* = 1.3 Hz, 4H), 6.65 (s, 2H), 6.37 (s, 2H), 4.70 (d, *J* = 13.7 Hz, 2H), 4.03 (d, *J* = 14.1 Hz, 4H), 3.90 (s, 2H), 3.76 (d, *J* = 14.4 Hz, 2H), 3.70 (s, 6H), 3.51 (s, 6H), 3.46 (s, 6H), 3.20 (s, 6H) ppm. $^{13}C\{^1H\}$ NMR (101 MHz, chloroform-*d*): δ 166.6 (2C), 151.5 (2C), 151.3 (2C), 151.1 (2C), 151.0 (2C), 148.1 (2C), 140.4 (2C), 134.5 (2C), 132.3 (2C), 132.2 (2C), 131.8 (2C), 131.7 (2C), 130.1 (2C), 130.0 (2C), 129.4 (2C), 129.3 (2C), 128.4 (2C), 128.2 (2C), 128.0 (2C), 127.4 (2C), 126.6 (2C), 126.2 (2C), 126.1 (2C), 125.7 (2C), 121.8 (2C), 118.3 (2C), 114.8 (2C), 114.3 (2C), 114.1 (2C), 113.8 (2C), 56.4 (2C), 56.00 (2C), 55.98 (2C), 55.2 (2C), 34.3 (2C), 30.0 (2C), 29.1 (1C) ppm. IR (ATR): ν = 1736 (C=O, esters) cm^{-1}. HRMS (ESI+) *m*/*z*: calcd. for $C_{73}H_{62}O_{12}Na$ $[M + Na]^+$: 1153.4134, found: 1153.4135.

Bis(7-methylnaphthalen-2-yl) (*pS*)-$3^2,3^5,5^2,5^5,7^2,7^5,9^2,9^5$-octamethoxy-1,3,5,7,9(1,4)-pentabenzenacyclodecaphane-$1^2,1^5$-dicarboxylate (3e)

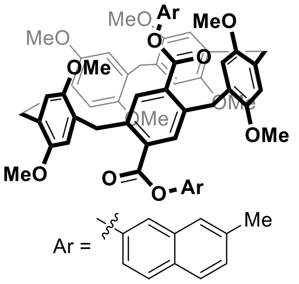

The title compound was synthesized according to the general procedure A (reaction time: 24 h), using pillar[5]arene **1a** (37.3 mg, 0.05 mmol) and 7-methylnaphthalen-2-ol (17.4 mg, 0.11 mmol). The crude product was purified by column chromatography (hexane/EtOAc – 10:1-7:1), affording **3e** (39.2 mg, 74%) as a white foam.

Er = 92:8 (*ee* = 84%), the enantiomeric excess of product **3e** was determined by HPLC on a Chiralpak® IC column (*n*-heptane/*i*-PrOH – 70:30, flow rate = 1.0 ml/min, λ = 190 nm, t = 25 °C): t_R = 8.9 min (major), t_R = 15.3 min (minor). $[\alpha]_D^{20}$ = -3.0 (c = 1.3, $CHCl_3$). ^1H NMR (400 MHz, chloroform-*d*): δ 8.10 (s, 2H), 7.88 (d, J = 8.8 Hz, 2H), 7.80 (d, J = 8.4 Hz, 2H), 7.65 (s, 2H), 7.44 (d, J = 2.3 Hz, 2H), 7.36 (dd, J = 8.4, 1.8 Hz, 2H), 7.16 (dd, J = 8.8, 2.3 Hz, 2H), 6.76 (d, J = 6.2 Hz, 4H), 6.66 (s, 2H), 6.38 (s, 2H), 4.69 (d, J = 13.6 Hz, 2H), 3.99 (dd, J = 16.0, 13.9 Hz, 4H), 3.88 (s, 2H), 3.75 (d, J = 14.3 Hz, 2H), 3.67 (s, 6H), 3.54 (s, 6H), 3.47 (s, 6H), 3.21 (s, 6H), 2.56 (s, 6H) ppm. $^{13}C\{^1H\}$ NMR (101 MHz, chloroform-*d*): δ 166.6 (2C), 151.4 (2C), 151.3 (2C), 151.1 (2C), 150.9 (2C), 148.7 (2C), 140.4 (2C), 136.6 (2C), 134.5 (2C), 134.3 (2C), 132.2 (2C), 130.0 (2C), 129.30 (2C), 129.26 (4C), 128.3 (2C), 128.0 (2C), 127.7 (2C), 127.5 (2C), 126.8 (2C), 120.4 (2C), 118.3 (2C), 114.7 (2C), 114.3 (2C), 114.1 (2C), 113.8 (2C), 56.3 (2C), 56.1 (2C), 56.0 (2C), 55.2 (2C), 34.0 (2C), 29.9 (2C), 29.2 (1C), 21.9 (2C) ppm. IR (ATR): ν = 1734 (C=O, esters) cm^{-1}. HRMS (ESI+) *m*/*z*: calcd. for $C_{67}H_{62}O_{12}Na$ $[M + Na]^+$: 1081.4134, found: 1081.4136.

Bis(7-methoxynaphthalen-2-yl) (*pS*)-$3^2,3^5,5^2,5^5,7^2,7^5,9^2,9^5$-octamethoxy-1,3,5,7,9(1,4)-pentabenzenacyclodecaphane-$1^2,1^5$-dicarboxylate (3f)

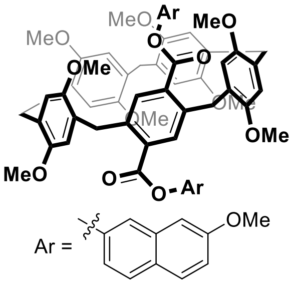

The title compound was synthesized according to the general procedure A (reaction time: 48 h), using pillar[5]arene **1a** (37.3 mg, 0.05 mmol) and 7-methoxynaphthalen-2-ol (19.2 mg, 0.11 mmol). The crude product was purified by column chromatography (hexane/EtOAc – 10:1-5:1), affording **3f** (37.6 mg, 69%) as a white foam.

Er = 92:8 (*ee* = 84%), the enantiomeric excess of product **3f** was determined by HPLC on a Chiralpak® IC column (*n*-heptane/*i*-PrOH – 70:30, flow rate = 1.0 ml/min, λ = 190 nm, t = 25 °C): t_R = 18.4 min (major), t_R = 27.0 min (minor). $[\alpha]_D^{20}$ = +5.8 (c = 1.7, $CHCl_3$). ^1H NMR (400 MHz, chloroform-*d*): δ 8.07 (s, 2H), 7.84 (d, J = 8.7 Hz, 2H), 7.79 (d, J = 9.0 Hz, 2H), 7.32 (d, J = 2.4 Hz, 2H), 7.24 (d, J = 2.5 Hz, 2H), 7.18 (dd, J = 8.9, 2.5 Hz, 2H), 7.09 (dd, J = 8.7, 2.3 Hz, 2H), 6.74 (d, J = 2.5 Hz, 4H), 6.67 (s, 2H), 6.22 (s, 2H), 4.65 (d, J = 13.6 Hz, 2H), 4.14 – 3.98 (m, 4H), 3.97 (s, 7H), 3.92 (s, 2H), 3.69 (s, 8H), 3.49 (s, 6H), 3.48 (s, 6H), 3.04 (s, 6H) ppm. $^{13}C\{^1H\}$ NMR (101 MHz, chloroform-*d*): δ 166.6 (2C), 158.4 (2C), 151.5 (2C), 151.3 (2C), 151.0 (2C), 150.8 (2C), 149.2 (2C), 140.1 (2C), 135.4 (2C), 134.5 (2C), 132.2 (2C), 129.7 (2C), 129.4 (2C), 129.3 (2C), 129.2 (2C), 127.7 (2C), 127.4 (2C), 127.2 (2C), 118.88 (2C), 118.86 (2C), 118.1 (2C), 115.0 (2C), 114.1 (2C), 114.0 (2C), 113.5 (2C), 105.8 (2C), 56.4 (2C), 55.91 (2C), 55.86 (2C), 55.5 (2C), 55.0 (2C), 34.5 (2C), 29.7 (2C), 28.7 (1C) ppm. IR (ATR): ν = 1736 (C=O, esters) cm^{-1}. HRMS (ESI+) *m*/*z*: calcd. for $C_{67}H_{62}O_{14}Na$ $[M + Na]^+$: 1113.4032, found: 1113.4036.

Bis(7-(methoxycarbonyl)naphthalen-2-yl) (*pS*)-3²,3⁵,5²,5⁵,7²,7⁵,9²,9⁵-octamethoxy-1,3,5,7,9(1,4)-pentabenzenacyclodecaphane-1²,1⁵-dicarboxylate (3g)

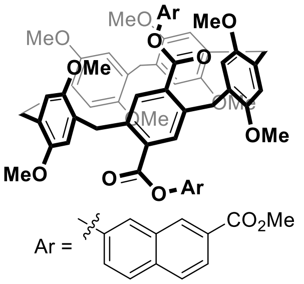

The title compound was synthesized according to the general procedure A (reaction time: 48 h), using pillar[5]arene **1a** (37.3 mg, 0.05 mmol) and methyl 7-hydroxy-2-naphthoate (22.2 mg, 0.11 mmol). The crude product was purified by column chromatography (hexane/EtOAc – 10:1-5:1), affording **3g** (53.2 mg, 93%) as a white foam.

Er = 92:8 (*ee* = 84%), the enantiomeric excess of product **3g** was determined by HPLC on a Chiralpak® IG column (*n*-heptane/*i*-PrOH – 50:50, flow rate = 1.0 ml/min, λ = 220 nm, t = 25 °C): t_R = 52.5 min (major), t_R = 101.1 min (minor). $[\alpha]_D^{20}$ = -13.5 (c = 2.3, $CHCl_3$). ^{1}H NMR (400 MHz, chloroform-*d*): δ 8.67 (d, J = 1.6 Hz, 2H), 8.15 – 8.09 (m, 4H), 7.97 (dd, J = 8.7, 7.3 Hz, 4H), 7.67 (d, J = 2.3 Hz, 2H), 7.34 (dd, J = 8.8, 2.3 Hz, 2H), 6.76 (s, 2H), 6.74 (s, 2H), 6.70 (s, 2H), 6.38 (s, 2H), 4.70 (d, J = 13.6 Hz, 2H), 4.08 – 3.92 (m, 10H), 3.88 (s, 2H), 3.76 (d, J = 14.3 Hz, 2H), 3.67 (s, 6H), 3.54 (s, 6H), 3.52 (s, 6H), 3.20 (s, 6H) ppm. ^{13}C{^{1}H} NMR (101 MHz, chloroform-*d*): δ 167.2 (2C), 166.3 (2C), 151.4 (2C), 151.3 (2C), 151.0 (2C), 150.9 (2C), 149.1 (2C), 140.5 (2C), 134.5 (2C), 133.7 (2C), 133.1 (2C), 132.0 (2C), 130.8 (2C), 129.50 (2C), 129.46 (2C), 129.4 (2C), 128.5 (2C), 128.2 (2C), 127.9 (2C), 127.3 (2C), 125.5 (2C), 123.8 (2C), 120.3 (2C), 114.7 (2C), 114.24 (2C), 114.20 (2C), 113.7 (2C), 56.3 (2C), 56.1 (2C), 56.0 (2C), 55.1 (2C), 52.5 (2C), 34.1 (2C), 29.7 (2C), 29.1 (1C) ppm. IR (ATR): ν = 1720 (C=O, esters) cm^{-1}. HRMS (ESI+) *m*/*z*: calcd. for $C_{69}H_{62}O_{16}Na$ $[M + Na]^+$: 1169.3930, found: 1169.3932.

Bis(7-cyanonaphthalen-2-yl) (*pS*)-3²,3⁵,5²,5⁵,7²,7⁵,9²,9⁵-octamethoxy-1,3,5,7,9(1,4)-pentabenzenacyclodecaphane-1²,1⁵-dicarboxylate (3h)

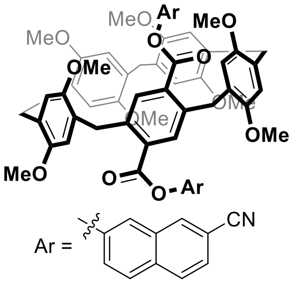

The title compound was synthesized according to the general procedure A (reaction time: 20 h), using pillar[5]arene **1a** (37.3 mg, 0.05 mmol) and 7-hydroxy-2-naphthonitrile (18.6 mg, 0.11 mmol). The crude product was purified by column chromatography (hexane/EtOAc – 3:1), affording **3h** (43.8 mg, 81%) as a white foam.

Er = 92:8 (*ee* = 83%), the enantiomeric excess of product **3h** was determined by HPLC on a Chiralpak® IC column (*n*-heptane/*i*-PrOH – 40:60, flow rate = 1.0 ml/min, λ = 220 nm, t = 35 °C): t_R = 79.0 min (major), t_R = 142.1 min (minor). $[\alpha]_D^{20}$ = +6.5 (c = 1.2, $CHCl_3$). ^{1}H NMR (400 MHz, chloroform-*d*): δ 8.30 – 8.25 (m, 2H), 8.05 (s, 2H), 8.00 (dd, J = 8.8, 2.6 Hz, 4H), 7.67 (dd, J = 8.5, 1.6 Hz, 2H), 7.55 (d, J = 2.2 Hz, 2H), 7.38 (dd, J = 8.8, 2.3 Hz, 2H), 6.74 (s, 2H), 6.69 (s, 2H), 6.62 (s, 2H), 6.29 (s, 2H), 4.64 (d, J = 13.8 Hz, 2H), 3.98 (dd, J = 14.1, 10.8 Hz, 4H), 3.89 (s, 2H), 3.69 (s, 8H), 3.48 (s, 6H), 3.44 (s, 6H), 3.10 (s, 6H) ppm. ^{13}C{^{1}H} NMR (101 MHz, chloroform-*d*): δ 166.2 (2C), 151.5 (2C), 151.3 (2C), 151.0 (2C), 150.9 (2C), 149.8 (2C), 140.5 (2C), 134.4 (2C), 133.9 (2C), 133.0 (2C), 132.9 (2C), 131.9 (2C), 129.8 (2C), 129.7 (2C), 129.6 (2C), 129.3 (2C), 127.7 (2C), 127.1 (2C), 126.7 (2C), 124.8 (2C), 119.7 (2C), 119.1 (2C), 115.0 (2C), 114.2 (2C), 114.1 (2C), 113.7 (2C), 110.6 (2C), 56.5 (2C), 56.0 (2C), 55.9 (2C), 55.1 (2C), 34.5 (2C), 29.9 (2C), 28.8 (1C) ppm. IR (ATR): ν = 2227 (CN), 1738 (C=O, esters) cm^{-1}. HRMS (ESI+) *m*/*z*: calcd. for $C_{67}H_{56}O_{12}N_2Na$ $[M + Na]^+$: 1103.3726, found: 1103.3724.

Bis(7-fluoronaphthalen-2-yl) (*pS*)-$3^2,3^5,5^2,5^5,7^2,7^5,9^2,9^5$-octamethoxy-1,3,5,7,9(1,4)-pentabenzenacyclodecaphane-$1^2,1^5$-dicarboxylate (3i)

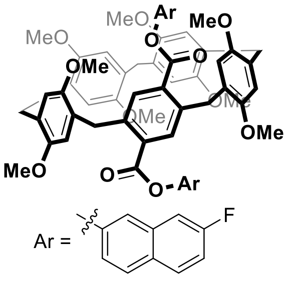

The title compound was synthesized according to the general procedure A (reaction time: 22 h), using pillar[5]arene **1a** (37.3 mg, 0.05 mmol) and 7-fluoronaphthalen-2-ol (17.8 mg, 0.11 mmol). The crude product was purified by column chromatography (hexane/EtOAc – 10:1-6:1), affording **3i** (43.7 mg, 82%) as a white foam.

Er = 93:7 (*ee* = 87%), the enantiomeric excess of product **3i** was determined by HPLC on a Chiralpak® IC column (*n*-heptane/*i*-PrOH – 70:30, flow rate = 1.0 ml/min, λ = 190 nm, t = 25 °C): t_R = 9.9 min (major), t_R = 14.2 min (minor). $[\alpha]_D^{20}$ = +8.7 (c = 2.1, $CHCl_3$). ^{1}H NMR (400 MHz, chloroform-*d*): δ 8.07 (s, 2H), 7.96 – 7.83 (m, 4H), 7.52 (dd, J = 9.8, 2.5 Hz, 2H), 7.42 (d, J = 2.2 Hz, 2H), 7.31 (td, J = 8.7, 2.5 Hz, 2H), 7.20 (dd, J = 8.8, 2.3 Hz, 2H), 6.74 (d, J = 9.5 Hz, 4H), 6.64 (s, 2H), 6.31 (s, 2H), 4.65 (d, J = 13.7 Hz, 2H), 4.00 (dd, J = 14.1, 7.6 Hz, 4H), 3.89 (s, 2H), 3.73 (d, J = 14.5 Hz, 2H), 3.69 (s, 6H), 3.51 (s, 6H), 3.45 (s, 6H), 3.14 (s, 6H) ppm. ^{13}C{^{1}H} NMR (101 MHz, chloroform-*d*): δ 166.4 (2C), 161.4 (d, J = 247.1 Hz, 2C), 151.5 (2C), 151.3 (2C), 151.0 (2C), 150.9 (2C), 149.5 (2C), 140.3 (2C), 135.0 (d, J = 9.8 Hz, 2C), 134.4 (2C), 132.1 (2C), 130.4 (d, J = 9.4 Hz, 2C), 129.6 (2C), 129.5 (2C), 129.4 (2C), 128.7 (2C), 127.8 (2C), 127.3 (2C), 120.7 (d, J = 2.7 Hz, 2C), 118.54 (2C), 118.49 (2C), 116.5 (d, J = 25.4 Hz, 2C), 114.9 (2C), 114.2 (d, J = 8.1 Hz, 2C), 113.7 (2C), 111.0 (d, J = 21.1 Hz, 2C), 56.4 (2C), 56.0 (2C), 55.9 (2C), 55.1 (2C), 34.3 (2C), 29.9 (2C), 28.9 (1C) ppm. ^{19}F NMR (376 MHz, chloroform-*d*): δ -113.43 (td, J = 9.2, 5.6 Hz) ppm. IR (ATR): ν = 1738 (C=O, esters) cm^{-1}. HRMS (ESI+) *m*/*z*: calcd. for $C_{65}H_{56}O_{12}F_2Na$ $[M + Na]^+$: 1089.3632, found: 1089.3633.

Bis(7-chloronaphthalen-2-yl) (*pS*)-$3^2,3^5,5^2,5^5,7^2,7^5,9^2,9^5$-octamethoxy-1,3,5,7,9(1,4)-pentabenzenacyclodecaphane-$1^2,1^5$-dicarboxylate (3j)

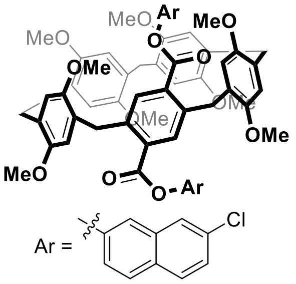

The title compound was synthesized according to the general procedure A (reaction time: 47 h), using pillar[5]arene **1a** (37.3 mg, 0.05 mmol) and 7-chloronaphthalen-2-ol (19.7 mg, 0.11 mmol). The crude product was purified by column chromatography (hexane/EtOAc – 10:1-6:1), affording **3j** (42.3 mg, 77%) as a white foam.

Er = 90:10 (*ee* = 80%), the enantiomeric excess of product **3j** was determined by HPLC on a Chiralpak® IC column (*n*-heptane/*i*-PrOH – 70:30, flow rate = 1.0 ml/min, λ = 190 nm, t = 25 °C): t_R = 8.9 min (major), t_R = 13.9 min (minor). $[\alpha]_D^{20}$ = -2.9 (c = 2.1, $CHCl_3$). ^{1}H NMR (400 MHz, chloroform-*d*): δ 8.07 (s, 2H), 7.90 (d, J = 8.9 Hz, 2H), 7.88 (d, J = 2.1 Hz, 2H), 7.84 (d, J = 8.8 Hz, 2H), 7.47 (dd, J = 8.8, 2.1 Hz, 2H), 7.42 (d, J = 2.2 Hz, 2H), 7.22 (dd, J = 8.8, 2.3 Hz, 2H), 6.73 (d, J = 7.3 Hz, 4H), 6.64 (s, 2H), 6.33 (s, 2H), 4.65 (d, J = 13.7 Hz, 2H), 4.01 – 3.95 (m, 4H), 3.88 (s, 2H), 3.73 (d, J = 14.4 Hz, 2H), 3.68 (s, 6H), 3.51 (s, 6H), 3.46 (s, 6H), 3.15 (s, 6H) ppm. ^{13}C{^{1}H} NMR (101 MHz, chloroform-*d*): δ 166.4 (2C), 151.5 (2C), 151.3 (2C), 151.1 (2C), 150.9 (2C), 149.5 (2C), 140.4 (2C), 134.7 (2C), 134.5 (2C), 132.9 (2C), 132.0 (2C), 129.9 (2C), 129.6 (2C), 129.5 (2C), 129.5 (2C), 129.4 (2C), 127.9 (2C), 127.3 (2C), 127.0 (2C), 126.5 (2C), 121.7 (2C), 118.3 (2C), 114.8 (2C), 114.24 (2C), 114.15 (2C), 113.7 (2C), 56.4 (2C), 56.1 (2C), 56.0 (2C), 55.1 (2C), 34.2 (2C), 29.9 (2C), 29.0 (1C) ppm. IR (ATR): ν = 1738 (C=O, ester) cm^{-1}. HRMS (ESI+) *m*/*z*: calcd. for $C_{65}H_{56}O_{12}Cl_2Na$ $[M + Na]^+$: 1121.3041, found: 1121.3043.

Bis(7-bromonaphthalen-2-yl) (*pS*)-3^2,3^5,5^2,5^5,7^2,7^5,9^2,9^5-octamethoxy-1,3,5,7,9(1,4)-pentabenzenacyclodecaphane-1^2,1^5-dicarboxylate (3k)

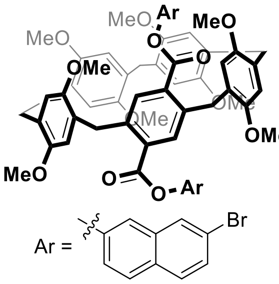

The title compound was synthesized according to the general procedure A (reaction time: 72 h), using pillar[5]arene **1a** (37.3 mg, 0.05 mmol) and 7-bromonaphthalen-2-ol (24.5 mg, 0.11 mmol). The crude product was purified by column chromatography (hexane/EtOAc – 10:1-5:1), affording **3k** (49.3 mg, 83%) as a white foam.

Er = 87:13 (*ee* = 74%), the enantiomeric excess of product **3k** was determined by HPLC on a Chiralpak® IC column (*n*-heptane/*i*-PrOH – 70:30, flow rate = 1.0 ml/min, λ = 190 nm, t = 25 °C): t_R = 9.8 min (major), t_R = 16.0 min (minor). $[\alpha]_D^{20}$ ~ 0 (c = 1.5, $CHCl_3$). ^{1}H NMR (400 MHz, chloroform-*d*): δ 8.07 (s, 2H), 8.06 (d, J = 1.9 Hz, 2H), 7.90 (d, J = 8.9 Hz, 2H), 7.77 (d, J = 8.7 Hz, 2H), 7.60 (dd, J = 8.7, 2.0 Hz, 2H), 7.42 (d, J = 2.3 Hz, 2H), 7.24 (dd, J = 8.8, 2.3 Hz, 2H), 6.74 (d, J = 5.8 Hz, 4H), 6.65 (s, 2H), 6.34 (s, 2H), 4.66 (d, J = 13.6 Hz, 2H), 4.04 – 3.95 (m, 4H), 3.89 (s, 2H), 3.74 (d, J = 14.4 Hz, 2H), 3.68 (s, 6H), 3.52 (s, 6H), 3.47 (s, 6H), 3.16 (s, 6H) ppm. ^{13}C{^{1}H} NMR (101 MHz, chloroform-*d*): δ 166.4 (2C), 151.4 (2C), 151.3 (2C), 151.0 (2C), 150.9 (2C), 149.4 (2C), 140.4 (2C), 135.1 (2C), 134.5 (2C), 132.0 (2C), 130.1 (2C), 129.8 (2C), 129.64 (2C), 129.58 (2C), 129.48 (4C), 129.4 (2C), 127.9 (2C), 127.3 (2C), 121.9 (2C), 121.1 (2C), 118.2 (2C), 114.8 (2C), 114.23 (2C), 114.16 (2C), 113.7 (2C), 56.4 (2C), 56.1 (2C), 56.0 (2C), 55.1 (2C), 34.2 (2C), 29.9 (2C), 29.0 (1C) ppm. IR (ATR): ν = 1734 (C=O, esters) cm^{-1}. HRMS (ESI+) *m*/*z*: calcd. for $C_{65}H_{56}O_{12}Br_2Na$ $[M + Na]^+$: 1209.2031, found: 1209.2035.

Bis(6-methylnaphthalen-2-yl) (*pS*)-3^2,3^5,5^2,5^5,7^2,7^5,9^2,9^5-octamethoxy-1,3,5,7,9(1,4)-pentabenzenacyclodecaphane-1^2,1^5-dicarboxylate (3l)

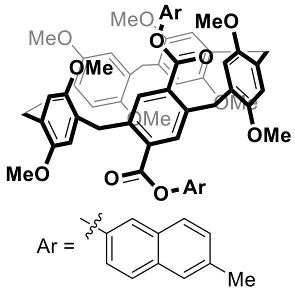

The title compound was synthesized according to the general procedure A (reaction time: 22 h), using pillar[5]arene **1a** (37.3 mg, 0.05 mmol) and 6-methylnaphthalen-2-ol (17.4 mg, 0.11 mmol). The crude product was purified by column chromatography (hexane/EtOAc – 10:1-8:1), affording **3l** (34.3 mg, 65%) as a white foam.

Er = 95:5 (*ee* = 89%), the enantiomeric excess of product **3l** was determined by HPLC on a Chiralpak® IC column (*n*-heptane/*i*-PrOH – 70:30, flow rate = 1.0 ml/min, λ = 190 nm, t = 25 °C): t_R = 10.4 min (major), t_R = 14.2 min (minor). $[\alpha]_D^{20}$ ~ 0 (c = 2.0, $CHCl_3$). ^{1}H NMR (400 MHz, chloroform-*d*): δ 8.08 (s, 2H), 7.83 (d, J = 8.8 Hz, 2H), 7.79 (d, J = 8.4 Hz, 2H), 7.67 (s, 2H), 7.44 (d, J = 2.3 Hz, 2H), 7.39 (dd, J = 8.4, 1.7 Hz, 2H), 7.19 (dd, J = 8.8, 2.3 Hz, 2H), 6.74 (d, J = 1.5 Hz, 4H), 6.65 (s, 2H), 6.33 (s, 2H), 4.66 (d, J = 13.6 Hz, 2H), 3.99 (d, J = 14.0 Hz, 4H), 3.88 (s, 2H), 3.73 (d, J = 14.4 Hz, 2H), 3.67 (s, 6H), 3.50 (s, 6H), 3.46 (s, 6H), 3.16 (s, 6H), 2.55 (s, 6H) ppm. ^{13}C{^{1}H} NMR (101 MHz, chloroform-*d*): δ 166.7 (2C), 151.5 (2C), 151.3 (2C), 151.1 (2C), 150.9 (2C), 147.9 (2C), 140.3 (2C), 135.7 (2C), 134.4 (2C), 132.21 (2C), 132.15 (2C), 131.9 (2C), 129.4 (2C), 129.3 (2C), 129.2 (2C), 128.9 (2C), 127.9 (2C), 127.6 (2C), 127.4 (2C), 126.9 (2C), 121.3 (2C), 118.7 (2C), 114.7 (2C), 114.3 (2C), 114.1 (2C), 113.7 (2C), 56.3 (2C), 56.0 (4C), 55.2 (2C), 34.2 (2C), 29.9 (2C), 29.0 (1C), 21.8 (2C) ppm. IR (ATR): ν = 1736 (C=O, esters) cm^{-1}. HRMS (ESI+) *m*/*z*: calcd. for $C_{67}H_{62}O_{12}Na$ $[M + Na]^+$: 1081.4134, found: 1081.4035.

Bis(6-methoxynaphthalen-2-yl) (*pS*)-3^2,3^5,5^2,5^5,7^2,7^5,9^2,9^5-octamethoxy-1,3,5,7,9(1,4)-pentabenzenacyclodecaphane-1^2,1^5-dicarboxylate (3m)

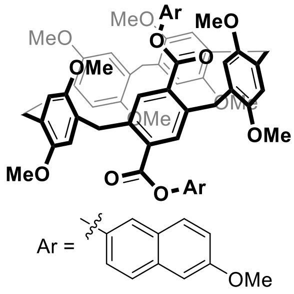

The title compound was synthesized according to the general procedure A (reaction time: 48 h), using pillar[5]arene **1a** (37.3 mg, 0.05 mmol) and 6-methoxynaphthalen-2-ol (19.2 mg, 0.11 mmol). The crude product was purified by column chromatography (hexane/EtOAc – 10:1-5:1), affording **3m** (47.5 mg, 87%) as a white foam.

Er = 96:4 (*ee* = 92%), the enantiomeric excess of product **3m** was determined by HPLC on a Chiralpak® IC column (*n*-heptane/*i*-PrOH – 70:30, flow rate = 1.0 ml/min, λ = 190 nm, t = 25 °C): t_R = 19.8 min (major), t_R = 33.7 min (minor). $[\alpha]_D^{20}$ = +1.3 (c = 1.9, $CHCl_3$). ^1H NMR (400 MHz, chloroform-*d*): δ 8.06 (s, 2H), 7.80 (t, J = 8.6 Hz, 4H), 7.41 (d, J = 2.3 Hz, 2H), 7.25 – 7.16 (m, 6H), 6.73 (d, J = 2.6 Hz, 4H), 6.64 (s, 2H), 6.30 (s, 2H), 4.65 (d, J = 13.6 Hz, 2H), 4.04 – 3.95 (m, 10H), 3.88 (s, 2H), 3.72 (d, J = 14.5 Hz, 2H), 3.67 (s, 6H), 3.50 (s, 6H), 3.45 (s, 6H), 3.13 (s, 6H) ppm. $^{13}C\{^1H\}$ NMR (101 MHz, chloroform-*d*): δ 166.8, 157.8, 151.5, 151.3, 151.1, 150.9, 146.9, 140.2, 134.4, 132.9, 132.2, 129.5, 129.29, 129.25 (4C), 128.2, 127.9, 127.4, 121.7, 119.8, 118.9, 114.8, 114.2, 114.1, 113.7, 106.0, 56.4, 57.0, 56.0, 55.5, 55.2, 34.2, 29.9, 29.0 (1C) ppm. IR (ATR): ν = 1736 (C=O, esters) cm^{-1}. HRMS (ESI+) *m*/*z*: calcd. for $C_{67}H_{62}O_{14}Na$ $[M + Na]^+$: 1113.4032, found: 1113.4036.

Bis(6-(methoxycarbonyl)naphthalen-2-yl) (*pS*)-3^2,3^5,5^2,5^5,7^2,7^5,9^2,9^5-octamethoxy-1,3,5,7,9(1,4)-pentabenzenacyclodecaphane-1^2,1^5-dicarboxylate (3n)

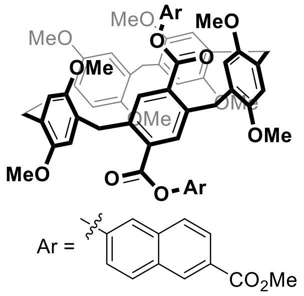

The title compound was synthesized according to the general procedure A (reaction time: 72 h), using pillar[5]arene **1a** (37.3 mg, 0.05 mmol) and methyl 6-hydroxy-2-naphthoate (22.2 mg, 0.11 mmol). The crude product was purified by column chromatography (hexane/EtOAc – 3:1), affording **3n** (42.7 mg, 74%) as a white foam.

Er = 91:9 (*ee* = 82%), the enantiomeric excess of product **3n** was determined by HPLC on a Chiralpak® IG column (*n*-heptane/*i*-PrOH – 50:50, flow rate = 1.0 ml/min, λ = 223 nm, t = 25 °C): t_R = 40.6 min (major), t_R = 62.3 min (minor). $[\alpha]_D^{20}$ = -5.9 (c = 1.0, $CHCl_3$). ^1H NMR (400 MHz, chloroform-*d*): δ 8.69 – 8.64 (m, 2H), 8.14 (dd, J = 8.6, 1.7 Hz, 2H), 8.05 (d, J = 10.3 Hz, 4H), 7.94 (d, J = 8.6 Hz, 2H), 7.52 (d, J = 2.3 Hz, 2H), 7.28 (dd, J = 8.8, 2.3 Hz, 2H), 6.74 (s, 2H), 6.70 (s, 2H), 6.63 (s, 2H), 6.29 (s, 2H), 4.64 (d, J = 13.7 Hz, 2H), 4.03 – 3.95 (m, 10H), 3.88 (s, 2H), 3.73 – 3.61 (m, 10H), 3.47 (s, 6H), 3.44 (s, 6H), 3.11 (s, 6H) ppm. $^{13}C\{^1H\}$ NMR (101 MHz, chloroform-*d*): δ 167.2 (2C), 166.3 (2C), 151.5 (2C), 151.3 (2C), 151.0 (2C), 150.9 (2C), 150.4 (2C), 140.4 (2C), 136.3 (2C), 134.5 (2C), 132.0 (2C), 131.1 (2C), 131.0 (2C), 130.8 (2C), 129.6 (2C), 129.4 (2C), 128.1 (2C), 127.8 (2C), 127.7 (2C), 127.2 (2C), 126.3 (2C), 122.4 (2C), 119.1 (2C), 114.9 (2C), 114.2 (2C), 114.1 (2C), 113.7 (2C), 56.4 (2C), 56.0 (2C), 55.9 (2C), 55.1 (2C), 52.5 (2C), 34.4 (2C), 29.9 (2C), 28.9 (1C) ppm. IR (ATR): ν = 1716 (C=O, esters) cm^{-1}. HRMS (ESI+) *m*/*z*: calcd. for $C_{69}H_{62}O_{16}Na$ $[M + Na]^+$: 1169.3930, found: 1169.3939.

Bis(6-(1,3-dioxolan-2-yl)naphthalen-2-yl) (*pS*)-$3^2,3^5,5^2,5^5,7^2,7^5,9^2,9^5$-octamethoxy-1,3,5,7,9(1,4)-pentabenzenacyclodecaphane-$1^2,1^5$-dicarboxylate (3o)

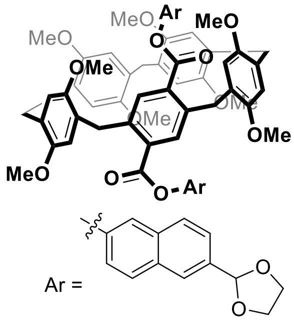

The title compound was synthesized according to the general procedure A (reaction time: 2 h), using pillar[5]arene **1a** (37.3 mg, 0.05 mmol) and 6-(1,3-dioxolan-2-yl)naphthalen-2-ol (23.8 mg, 0.11 mmol). The crude product was purified by column chromatography (hexane/EtOAc – 2:1-1:1), affording **3o** (23.2 mg, 58%) as a yellow amorphous solid.

Er = 92:8 (*ee* = 84%), the enantiomeric excess of product **3o** was determined by HPLC on a Chiralpak® IC column (*n*-heptane/*i*-PrOH – 70:30, flow rate = 1.0 ml/min, λ = 220 nm, t = 25 °C): t_R = 42.4 min (major), t_R = 89.9 min (minor). $[\alpha]_D^{20}$ ~ 0 (c = 1.4, $CHCl_3$). ^{1}H NMR (400 MHz, chloroform-*d*): δ 8.06 (s, 2H), 8.03 – 7.99 (m, 2H), 7.94 (t, J = 8.0 Hz, 4H), 7.67 (dd, J = 8.6, 1.7 Hz, 2H), 7.48 (d, J = 2.3 Hz, 2H), 7.24 (dd, J = 8.8, 2.3 Hz, 2H), 6.74 (s, 2H), 6.71 (s, 2H), 6.64 (s, 2H), 6.29 (s, 2H), 6.01 (s, 2H), 4.64 (d, J = 13.6 Hz, 2H), 4.26 – 4.17 (m, 4H), 4.17 – 4.08 (m, 4H), 4.00 (dd, J = 14.1, 12.0 Hz, 4H), 3.89 (s, 2H), 3.69 (s, 8H), 3.47 (s, 6H), 3.44 (s, 6H), 3.11 (s, 6H) ppm. ^{13}C{^{1}H} NMR (101 MHz, chloroform-*d*): δ 166.5 (2C), 151.5 (2C), 151.3 (2C), 151.0 (2C), 150.9 (2C), 149.0 (2C), 140.3 (2C), 135.6 (2C), 134.5 (4C), 132.1 (2C), 131.2 (2C), 130.0 (2C), 129.5 (2C), 129.3 (2C), 128.3 (2C), 127.8 (2C), 127.3 (2C), 126.1 (2C), 124.9 (2C), 121.8 (2C), 118.9 (2C), 114.8 (2C), 114.2 (2C), 114.1 (2C), 113.6 (2C), 104.0 (2C), 65.6 (4C), 56.5 (2C), 55.9 (4C), 55.1 (2C), 34.3 (2C), 29.9 (2C), 28.9 (1C) ppm. IR (ATR): ν = 1730 (C=O, esters) cm^{-1}. HRMS (ESI+) *m*/*z*: calcd. for $C_{71}H_{66}O_{16}Na$ $[M + Na]^+$: 1197.4243, found: 1197.4244.

Bis(6-fluoronaphthalen-2-yl) (*pS*)-$3^2,3^5,5^2,5^5,7^2,7^5,9^2,9^5$-octamethoxy-1,3,5,7,9(1,4)-pentabenzenacyclodecaphane-$1^2,1^5$-dicarboxylate (3p)

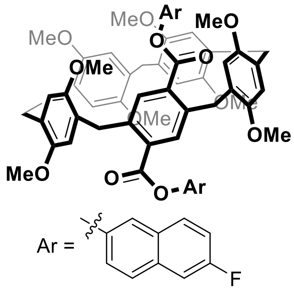

The title compound was synthesized according to the general procedure A (reaction time: 23 h), using pillar[5]arene **1a** (37.3 mg, 0.05 mmol) and 6-fluoronaphthalen-2-ol (17.8 mg, 0.11 mmol). The crude product was purified by column chromatography (hexane/EtOAc – 10:1-6:1), affording **3p** (41.2 mg, 77%) as a white foam.

Er = 93:7 (*ee* = 86%), the enantiomeric excess of product **3p** was determined by HPLC on a Chiralpak® IC column (*n*-heptane/*i*-PrOH – 70:30, flow rate = 1.0 ml/min, λ = 190 nm, t = 25 °C): t_R = 11.9 min (major), t_R = 23.6 min (minor). $[\alpha]_D^{20}$ = +12.8 (c = 1.6, $CHCl_3$). ^{1}H NMR (400 MHz, chloroform-*d*): δ 8.05 (s, 2H), 7.92 – 7.83 (m, 4H), 7.56 – 7.49 (m, 2H), 7.47 (d, J = 2.3 Hz, 2H), 7.38 – 7.33 (m, 2H), 7.24 (d, J = 2.4 Hz, 2H), 6.73 (s, 2H), 6.71 (s, 2H), 6.63 (s, 2H), 6.27 (s, 2H), 4.64 (d, J = 13.7 Hz, 2H), 3.99 (dd, J = 16.2, 14.1 Hz, 4H), 3.89 (s, 2H), 3.68 (s, 8H), 3.48 (s, 6H), 3.44 (s, 6H), 3.09 (s, 6H) ppm. ^{13}C{^{1}H} NMR (101 MHz, chloroform-*d*): δ 166.6 (2C), 160.8 (d, J = 246.1 Hz, 2C), 151.5 (2C), 151.3 (2C), 151.0 (2C), 150.9 (2C), 148.0 (d, J = 3.0 Hz, 2C), 140.3 (2C), 134.4 (2C), 132.4 (2C), 132.3 (2C), 132.1 (2C), 130.9 (2C), 130.2 (d, J = 9.0 Hz, 2C), 129.6 (2C), 129.4 (2C), 128.9 (d, J = 5.5 Hz, 2C), 127.8 (2C), 127.3 (2C), 122.5 (2C), 119.1 (2C), 117.4 (d, J = 25.4 Hz, 2C), 114.9 (2C), 114.2 (d, J = 4.6 Hz, 2C), 113.7 (2C), 111.1 (d, J = 20.6 Hz, 2C), 56.4 (2C), 56.0 (2C), 55.9 (2C), 55.1 (2C), 34.4 (2C), 29.9 (2C), 28.9 (1C) ppm. ^{19}F NMR (376 MHz, chloroform-*d*): δ -114.67 (td, J = 8.9, 5.4 Hz, 2F) ppm. IR (ATR): ν = 1738 (C=O, esters) cm^{-1}. HRMS (ESI+) *m*/*z*: calcd. for $C_{65}H_{56}O_{12}F_2Na$ $[M + Na]^+$: 1089.3632, found: 1089.3635.

Bis(6-chloronaphthalen-2-yl) (*pS*)-3²,3⁵,5²,5⁵,7²,7⁵,9²,9⁵-octamethoxy-1,3,5,7,9(1,4)-pentabenzenacyclodecaphane-1²,1⁵-dicarboxylate (3q)

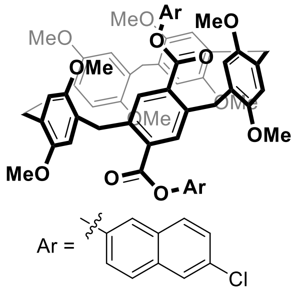

The title compound was synthesized according to the general procedure A (reaction time: 22 h), using pillar[5]arene **1a** (37.3 mg, 0.05 mmol) and 6-chloronaphthalen-2-ol (19.7 mg, 0.11 mmol). The crude product was purified by column chromatography (hexane/EtOAc – 10:1-6:1), affording **3q** (41.8 mg, 76%) as a white foam.

Single crystals of **3q** suitable for X-ray analysis were obtained by dissolving the compound (40 mg) in a minimal amount of chloroform (0.2 mL), followed by the addition of *i*PrOH (0.3 mL). The solution was allowed to crystallize at room temperature overnight. The resulting crystals were analyzed by X-ray diffraction (CCDC: 2498608).

Er = 93:7 (*ee* = 86%), the enantiomeric excess of product **3q** was determined by HPLC on a Chiralpak® IC column (*n*-heptane/*i*-PrOH – 70:30, flow rate = 1.0 ml/min, λ = 190 nm, t = 25 °C): t_R = 11.6 min (major), t_R = 19.3 min (minor). $[\alpha]_D^{20}$ = +2.5 (c = 1.8, $CHCl_3$). ^{1}H NMR (400 MHz, chloroform-*d*): δ 8.04 (s, 2H), 7.89 (d, J = 2.0 Hz, 2H), 7.86 (s, 2H), 7.83 (s, 2H), 7.51 (dd, J = 8.7, 2.1 Hz, 2H), 7.45 (d, J = 2.2 Hz, 2H), 7.24 (d, J = 2.3 Hz, 2H), 6.74 (s, 2H), 6.70 (s, 2H), 6.63 (s, 2H), 6.27 (s, 2H), 4.63 (d, J = 13.7 Hz, 2H), 3.99 (dd, J = 18.1, 14.1 Hz, 4H), 3.90 (s, 2H), 3.69 (s, 8H), 3.47 (s, 6H), 3.44 (s, 6H), 3.09 (s, 6H) ppm. ^{13}C{^{1}H} NMR (101 MHz, chloroform-*d*): δ 166.5 (2C), 151.5 (2C), 151.3 (2C), 151.0 (2C), 150.9 (2C), 148.7 (2C), 140.3 (2C), 134.4 (2C), 132.2 (4C), 132.1 (2C), 131.8 (2C), 129.6 (2C), 129.39 (2C), 129.36 (2C), 128.7 (2C), 127.9 (2C), 127.8 (2C), 127.3 (2C), 126.7 (2C), 122.6 (2C), 119.1 (2C), 114.9 (2C), 114.2 (2C), 114.1 (2C), 113.6 (2C), 56.4 (2C), 56.0 (2C), 55.9 (2C), 55.1 (2C), 34.4 (2C), 29.9 (2C), 28.8 (1C) ppm. IR (ATR): ν = 1740 (C=O, esters) cm^{-1}. HRMS (ESI+) *m*/*z*: calcd. for $C_{65}H_{56}O_{12}Cl_2Na$ $[M + Na]^+$: 1121.3041, found: 1121.3044.

Bis(6-bromonaphthalen-2-yl) (*pS*)-3²,3⁵,5²,5⁵,7²,7⁵,9²,9⁵-octamethoxy-1,3,5,7,9(1,4)-pentabenzenacyclodecaphane-1²,1⁵-dicarboxylate (3r)

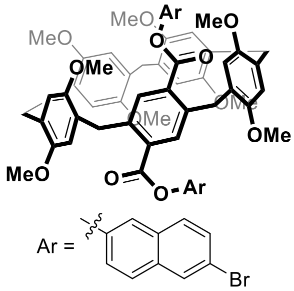

The title compound was synthesized according to the general procedure A (reaction time: 72 h), using pillar[5]arene **1a** (37.3 mg, 0.05 mmol) and 6-bromonaphthalen-2-ol (24.5 mg, 0.11 mmol). The crude product was purified by column chromatography (hexane/EtOAc – 10:1-5:1), affording **3r** (48.5 mg, 82%) as a white foam.

Er = 90:10 (*ee* = 79%), the enantiomeric excess of product **3r** was determined by HPLC on a Chiralpak® IC column (*n*-heptane/*i*-PrOH – 70:30, flow rate = 1.0 ml/min, λ = 190 nm, t = 25 °C): t_R = 12.7 min (major), t_R = 21.7 min (minor). $[\alpha]_D^{20}$ = +0.5 (c = 2.0, $CHCl_3$). ^{1}H NMR (400 MHz, chloroform-*d*): δ 8.08 (d, J = 1.9 Hz, 2H), 8.05 (d, J = 1.3 Hz, 2H), 7.84 (d, J = 8.9 Hz, 2H), 7.78 (d, J = 8.8 Hz, 2H), 7.64 (dd, J = 8.7, 1.8 Hz, 2H), 7.45 (d, J = 2.2 Hz, 2H), 7.26 – 7.21 (m, 2H), 6.74 (d, J = 1.3 Hz, 2H), 6.70 (s, 2H), 6.63 (d, J = 1.3 Hz, 2H), 6.27 (s, 2H), 4.64 (d, J = 13.7 Hz, 2H), 4.00 (dd, J = 19.5, 14.1 Hz, 4H), 3.90 (s, 2H), 3.76 – 3.66 (m, 8H), 3.47 (d, J = 1.3 Hz, 6H), 3.44 (d, J = 1.3 Hz, 6H), 3.09 (d, J = 1.3 Hz, 6H) ppm. ^{13}C{^{1}H} NMR (101 MHz, chloroform-*d*): δ 166.4 (2C), 151.5 (2C), 151.3 (2C), 151.0 (2C), 150.8 (2C), 148.8 (2C), 140.3 (2C), 134.4 (2C), 132.7 (2C), 132.4 (2C), 132.1 (2C), 130.3 (2C), 130.0 (2C), 129.6 (2C), 129.5 (2C), 129.4 (2C), 128.7 (2C), 127.7 (2C), 127.3 (2C), 122.6 (2C), 120.0 (2C), 119.1 (2C), 114.9 (2C), 114.13 (2C), 114.07 (2C), 113.6 (2C), 56.4 (2C), 55.93 (2C), 55.90 (2C), 55.1 (2C), 34.4 (2C), 29.9 (2C), 28.8 (1C) ppm. IR (ATR): ν = 1738 (C=O, esters) cm^{-1}. HRMS (ESI+) *m*/*z*: calcd. for $C_{65}H_{56}O_{12}Br_2Na$ $[M + Na]^+$: 1209.2031, found: 1209.2041.

Bis(5,6,7,8-tetrahydronaphthalen-2-yl) (*pS*)-$3^2,3^5,5^2,5^5,7^2,7^5,9^2,9^5$-octamethoxy-1,3,5,7,9(1,4)-pentabenzenacyclodecaphane-$1^2,1^5$-dicarboxylate (3s)

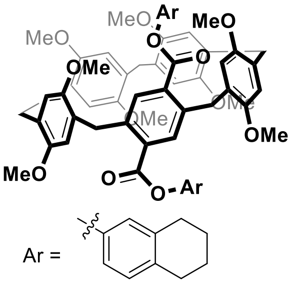

The title compound was synthesized according to the general procedure A (reaction time: 24 h), using pillar[5]arene **1a** (37.3 mg, 0.05 mmol) and 5,6,7,8-tetrahydronaphthalen-2-ol (16.3 mg, 0.11 mmol). The crude product was purified by column chromatography (hexane/EtOAc – 12:1), affording **3s** (31.2 mg, 60%) as a white foam. *Er* = 95:5 (*ee* = 89%), the enantiomeric excess of product **3s** was determined by HPLC on a Chiralpak® IC column (*n*-heptane/*i*-PrOH – 70:30, flow rate = 1.0 ml/min, λ = 190 nm, *t* = 25 °C): t_R = 10.4 min (major), t_R = 17.5 min (minor). $[\alpha]_D^{20}$ = +5.3 (*c* = 1.3, $CHCl_3$). ^1H NMR (400 MHz, chloroform-*d*): δ 8.03 (d, *J* = 1.1 Hz, 2H), 7.13 (d, *J* = 8.1 Hz, 2H), 6.84 – 6.77 (m, 4H), 6.75 (s, 2H), 6.71 (s, 2H), 6.68 (s, 2H), 6.38 (s, 2H), 4.65 (d, *J* = 13.5 Hz, 2H), 3.98 – 3.83 (m, 6H), 3.75 (d, *J* = 14.2 Hz, 2H), 3.65 (d, *J* = 1.0 Hz, 6H), 3.61 (d, *J* = 1.0 Hz, 6H), 3.51 (d, *J* = 1.0 Hz, 6H), 3.21 (s, 6H), 2.82 (q, *J* = 6.2 Hz, 8H), 1.85 (h, *J* = 3.8 Hz, 8H) ppm. $^{13}C\{^1H\}$ NMR (101 MHz, chloroform-*d*): δ 166.7 (2C), 151.3 (2C), 151.2 (2C), 151.1 (2C), 150.9 (2C), 148.5 (2C), 140.3 (2C), 138.7 (2C), 135.0 (2C), 134.4 (2C), 132.1 (2C), 130.1 (2C), 129.1 (4C), 128.1 (2C), 127.6 (2C), 121.9 (2C), 118.9 (2C), 114.5 (2C), 114.4 (2C), 114.1 (2C), 113.7 (2C), 56.2 (2C), 56.1 (2C), 56.0 (2C), 55.2 (2C), 33.8 (2C), 29.7 (2C), 29.6 (2C), 29.3 (1C), 29.1 (2C), 23.3 (2C), 23.1 (2C) ppm. IR (ATR): ν = 1737 (C=O, esters) cm^{-1}. HRMS (ESI+) *m*/*z*: calcd. for $C_{65}H_{66}O_{12}Na$ $[M + Na]^+$: 1061.4447, found: 1061.4448.

Di(naphthalen-1-yl) (*pS*)-$3^2,3^5,5^2,5^5,7^2,7^5,9^2,9^5$-octamethoxy-1,3,5,7,9(1,4)-pentabenzenacyclodecaphane-$1^2,1^5$-dicarboxylate (3t)

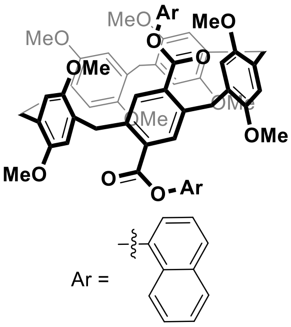

The title compound was synthesized according to the general procedure A (reaction time: 24 h), using pillar[5]arene **1a** (37.3 mg, 0.05 mmol) and naphthalen-1-ol (15.9 mg, 0.11 mmol). The crude product was purified by column chromatography (hexane/EtOAc – 10:1-5:1), affording **3t** (40.5 mg, 79%) as a white foam.

Er = 88:12 (*ee* = 76%), the enantiomeric excess of product **3t** was determined by HPLC on a Chiralpak® IC column (*n*-heptane/*i*-PrOH – 70:30, flow rate = 1.0 ml/min, λ = 190 nm, *t* = 25 °C): t_R = 6.4 min (major), t_R = 9.0 min (minor). $[\alpha]_D^{20}$ = +50.9 (*c* = 1.7, $CHCl_3$). ^1H NMR (400 MHz, chloroform-*d*): δ 8.39 (s, 2H), 7.98 – 7.91 (m, 2H), 7.84 (dt, *J* = 8.4, 1.1 Hz, 2H), 7.77 (dq, *J* = 8.0, 0.8 Hz, 2H), 7.62 – 7.51 (m, 6H), 7.19 (dd, *J* = 7.5, 1.1 Hz, 2H), 6.79 (s, 2H), 6.75 (s, 2H), 6.67 (s, 2H), 6.40 (s, 2H), 4.75 (d, *J* = 13.5 Hz, 2H), 4.08 (d, *J* = 13.5 Hz, 2H), 3.95 (d, *J* = 14.2 Hz, 2H), 3.89 (s, 2H), 3.72 (s, 8H), 3.44 (s, 6H), 3.43 (s, 6H), 3.23 (s, 6H) ppm. $^{13}C\{^1H\}$ NMR (101 MHz, chloroform-*d*): δ 166.2 (2C), 151.5 (2C), 151.2 (2C), 151.0 (2C), 150.8 (2C), 147.0 (2C), 141.3 (2C), 135.1 (2C), 134.9 (2C), 131.6 (2C), 129.4 (2C), 129.1 (2C), 128.3 (2C), 127.9 (2C), 127.4 (2C), 127.2 (2C), 126.9 (2C), 126.7 (2C), 126.4 (2C), 125.6 (2C), 121.3 (2C), 118.4 (2C), 114.8 (2C), 114.3 (2C), 114.0 (2C), 113.6 (2C), 56.4 (2C), 55.9 (2C), 55.8 (2C), 55.2 (2C), 33.9 (2C), 29.9 (2C), 28.8 (1C) ppm. IR (ATR): ν = 1738 (C=O, esters) cm^{-1}. HRMS (ESI+) *m*/*z*: calcd. for $C_{65}H_{58}O_{12}Na$ $[M + Na]^+$: 1053.3821, found: 1053.3823.

Bis(5,6,7,8-tetrahydronaphthalen-1-yl) (*pS*)-$3^2,3^5,5^2,5^5,7^2,7^5,9^2,9^5$-octamethoxy-1,3,5,7,9(1,4)-pentabenzenacyclodecaphane-$1^2,1^5$-dicarboxylate (3u)

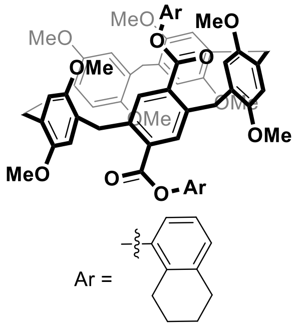

The title compound was synthesized according to the general procedure A (reaction time: 72 h, full conversion of monoester was not observed), using pillar[5]arene **1a** (37.3 mg, 0.05 mmol) and 5,6,7,8-tetrahydronaphthalen-1-ol (16.3 mg, 0.11 mmol). The crude product was purified by column chromatography (hexane/EtOAc – 12:1), affording **3u** (18.2 mg, 36%) as a white foam.

Er = 98:2 (*ee* = 95%), the enantiomeric excess of product **3u** was determined by HPLC on a Chiralpak® IC column (*n*-heptane/*i*-PrOH – 70:30, flow rate = 1.0 ml/min, λ = 190 nm, t = 25 °C): t_R = 5.3 min (major), t_R = 7.4 min (minor). $[\alpha]_D^{20}$ = +16.8 (c = 0.8, $CHCl_3$). ^1H NMR (400 MHz, chloroform-*d*): δ 8.13 (s, 2H), 7.16 (t, J = 7.7 Hz, 2H), 7.04 (dd, J = 7.5, 1.3 Hz, 2H), 6.79 (s, 2H), 6.75 – 6.67 (m, 6H), 6.43 (s, 2H), 4.66 (d, J = 13.4 Hz, 2H), 4.04 – 3.86 (m, 3H), 3.84 (s, 3H), 3.75 (d, J = 14.0 Hz, 2H), 3.67 (s, 6H), 3.64 (s, 6H), 3.49 (s, 6H), 3.27 (s, 6H), 2.84 (d, J = 5.9 Hz, 4H), 2.61 (q, J = 4.0 Hz, 4H), 1.82 (q, J = 6.1 Hz, 8H) ppm. $^{13}C\{^1H\}$ NMR (101 MHz, chloroform-*d*): δ 166.0 (2C), 151.4 (2C), 151.2 (2C), 151.1 (2C), 150.9 (2C), 149.3 (2C), 140.8 (2C), 139.4 (2C), 134.7 (2C), 131.8 (2C), 129.7 (1C), 129.11 (2C), 129.09 (2C), 128.0 (2C), 127.5 (2C), 127.2 (2C), 126.1 (2C), 119.1 (2C), 114.5 (2C), 114.4 (2C), 114.2 (2C), 113.8 (2C), 56.18 (2C), 56.16 (2C), 56.1 (2C), 55.3 (2C), 33.5 (2C), 29.9 (2C), 29.6 (2C), 29.23 (1C), 23.5 (2C), 22.8 (2C), 22.7 (2C) ppm. IR (ATR): ν = 1732 (C=O, ester) cm^{-1}. HRMS (ESI+) *m*/*z*: calcd. for $C_{65}H_{66}O_{12}Na$ $[M + Na]^+$: 1061.4447, found: 1061.4448.

Diphenyl $3^2,3^5,5^2,5^5,7^2,7^5,9^2,9^5$-octamethoxy-1,3,5,7,9(1,4)-pentabenzenacyclodecaphane-$1^2,1^5$-dicarboxylate (4a)

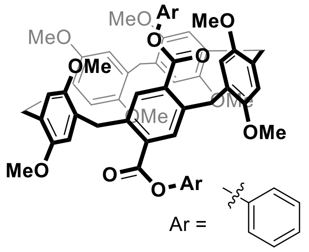

The title compound was synthesized according to the general procedure A (reaction time: 1 h), using pillar[5]arene **1a** (37.3 mg, 0.05 mmol) and phenol (10.4 mg, 0.11 mmol). The crude product was purified by column chromatography (hexane/EtOAc – 5:1), affording **4a** (37.8 mg, 81%) as a pale oil.

Note: We analyzed *rac*-**4a** by chiral HPLC on chiral columns used in this study for enantiomer separations (IA, IC, IG), but we detected only one peak without any separation.

$[\alpha]_D^{20}$ = 0 (c = 1.9, $CHCl_3$). ^1H NMR (400 MHz, chloroform-*d*): δ 8.01 (s, 2H), 7.51 – 7.37 (m, 4H), 7.33 – 7.27 (m, 2H), 7.12 – 7.01 (m, 4H), 6.72 (d, J = 4.4 Hz, 4H), 6.64 (s, 2H), 6.43 (s, 2H), 4.64 (d, J = 13.7 Hz, 2H), 3.98 (s, 2H), 3.81 (d, J = 29.8 Hz, 6H), 3.66 (s, 6H), 3.56 (s, 6H), 3.46 (s, 6H), 3.26 (s, 6H) ppm. $^{13}C\{^1H\}$ NMR (101 MHz, chloroform-*d*): δ 166.3 (2C), 151.4 (2C), 151.2 (2C), 151.1 (2C), 151.0 (2C), 150.9 (2C), 140.4 (2C), 134.3 (2C), 132.1 (2C), 129.6 (4C), 129.2 (2C), 129.1 (2C), 128.1 (2C), 127.4 (2C), 126.1 (2C), 121.9 (4C), 114.5 (2C), 114.4 (2C), 114.1 (2C), 113.9 (2C), 56.2 (2C), 56.1 (2C), 56.0 (2C), 55.2 (2C), 33.8 (1C), 29.9 (2C), 29.2 (1C) ppm. IR (ATR): ν = 1734 (C=O, esters) cm^{-1}. HRMS (ESI+) *m*/*z*: calcd. for $C_{57}H_{54}O_{12}Na$ $[M + Na]^+$: 953.3508, found: 953.3509.

Bis(4-bromophenyl) $3^2,3^5,5^2,5^5,7^2,7^5,9^2,9^5$-octamethoxy-1,3,5,7,9(1,4)-pentabenzenacyclodecaphane-$1^2,1^5$-dicarboxylate (4b)

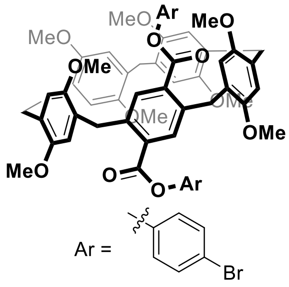

The title compound was synthesized according to the general procedure A (reaction time: 24 h), using pillar[5]arene **1a** (37.3 mg, 0.05 mmol) and 4-bromophenol (19.0 mg, 0.11 mmol). The crude product was purified by column chromatography (hexane/EtOAc – 10:1), affording **4b** (45.5 mg, 87%) as a white foam.

Note: We analyzed *rac*-**4b** by chiral HPLC on columns used in this study for enantiomer separations (IA, IC and IG), but we detected only one peak without any separation.

$[\alpha]_D^{20}$ = 0 (*c* = 2.1, $CHCl_3$). ^1H NMR (400 MHz, chloroform-*d*): δ 7.96 (s, 2H), 7.61 – 7.52 (m, 4H), 6.98 – 6.90 (m, 4H), 6.72 (s, 2H), 6.68 (s, 2H), 6.66 (s, 2H), 6.38 (s, 2H), 4.60 (d, *J* = 13.7 Hz, 2H), 3.93 (d, *J* = 13.7 Hz, 2H), 3.90 – 3.83 (m, 4H), 3.75 (d, *J* = 14.2 Hz, 2H), 3.69 (s, 6H), 3.57 (s, 6H), 3.49 (s, 6H), 3.18 (s, 6H) ppm. $^{13}C\{^1H\}$ NMR (101 MHz, chloroform-*d*): δ 165.9 (2C), 151.4 (2C), 151.1 (2C), 151.0 (2C), 150.9 (2C), 149.9 (2C), 140.4 (2C), 134.2 (2C), 132.7 (4C), 131.9 (2C), 129.33 (2C), 129.32 (2C), 127.9 (2C), 127.2 (2C), 123.7 (4C), 119.2 (2C), 114.7 (2C), 114.3 (2C), 114.0 (2C), 113.8 (2C), 56.3 (2C), 56.1 (2C), 56.0 (2C), 55.1 (2C), 34.0 (2C), 29.8 (2C), 29.0 (1C) ppm. IR (ATR): ν = 1738 (C=O, esters) cm^{-1}. HRMS (ESI+) *m*/*z*: calcd. for $C_{57}H_{52}O_{12}Br_2Na$ $[M + Na]^+$: 1109.1718, found: 1109.1723.

Di-*p*-tolyl $3^2,3^5,5^2,5^5,7^2,7^5,9^2,9^5$-octamethoxy-1,3,5,7,9(1,4)-pentabenzenacyclodecaphane-$1^2,1^5$-dicarboxylate (4c)

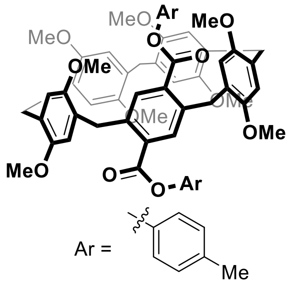

The title compound was synthesized according to the general procedure A (reaction time: 24 h), using pillar[5]arene **1a** (37.3 mg, 0.05 mmol) and 4-methylphenol (11.9 mg, 0.11 mmol). The crude product was purified by column chromatography (hexane/EtOAc – 10:1-5:1), affording **4c** (40.5 mg, 84%) as a white foam.

Note: We analyzed *rac*-**4c** by chiral HPLC on chiral columns used in this study for enantiomer separations (IA, IC and IG), but we detected only one peak without any separation.

$[\alpha]_D^{20}$ = 0 (*c* = 1.8, $CHCl_3$). ^1H NMR (400 MHz, chloroform-*d*): δ 8.02 (s, 2H), 7.25 – 7.16 (m, 4H), 7.00 – 6.91 (m, 4H), 6.72 (d, *J* = 7.0 Hz, 4H), 6.67 (s, 2H), 6.43 (s, 2H), 4.64 (d, *J* = 13.6 Hz, 2H), 3.95 (d, *J* = 13.6 Hz, 2H), 3.88 – 3.71 (m, 6H), 3.65 (s, 6H), 3.57 (s, 6H), 3.49 (s, 6H), 3.26 (s, 6H), 2.39 (s, 6H) ppm. $^{13}C\{^1H\}$ NMR (101 MHz, chloroform-*d*): δ 166.6 (2C), 151.3 (2C), 151.2 (2C), 151.1 (2C), 150.9 (2C), 148.7 (2C), 140.4 (2C), 135.8 (2C), 134.3 (2C), 132.1 (2C), 130.1 (4C), 129.2 (2C), 129.1 (2C), 128.1 (2C), 127.5 (2C), 121.5 (4C), 114.5 (2C), 114.4 (2C), 114.1 (2C), 113.8 (2C), 56.2 (2C), 56.1 (2C), 56.1 (2C), 55.2 (2C), 33.6 (2C), 29.8 (2C), 29.3 (1C), 21.1 ppm. IR (ATR): ν = 1734 (C=O, esters) cm^{-1}. HRMS (ESI+) *m*/*z*: calcd. for $C_{59}H_{58}O_{12}Na$ $[M + Na]^+$: 981.3821, found: 981.3824.

Bis(4-(*tert*-butyl)phenyl) (*pS*)-$3^2,3^5,5^2,5^5,7^2,7^5,9^2,9^5$-octamethoxy-1,3,5,7,9(1,4)-pentabenzenacyclodecaphane-$1^2,1^5$-dicarboxylate (4d)

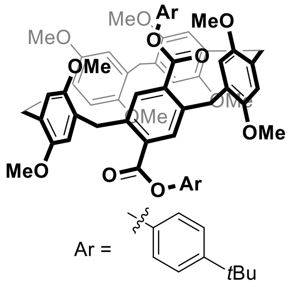

The title compound was synthesized according to the general procedure A (reaction time: 24 h), using pillar[5]arene **1a** (37.3 mg, 0.05 mmol) and 4-*tert*-butylphenol (16.5 mg, 0.11 mmol). The crude product was purified by column chromatography (hexane/EtOAc – 9:1), affording **4d** (38.4 mg, 74%) as a light-yellow foam.

Er = 95:5 (*ee* = 89%), the enantiomeric excess of product **4d** was determined by HPLC on a Chiralpak® IC column (*n*-heptane/*i*-PrOH – 80:20, flow rate = 1.0 ml/min, λ = 190 nm, t = 25 °C): t_R = 9.3 min (major), t_R = 12.6 min (minor). $[\alpha]_D^{20}$ = -4.1 (c = 1.6, $CHCl_3$). ^1H NMR (400 MHz, chloroform-*d*): δ 7.97 (s, 2H), 7.48 – 7.40 (m, 4H), 6.99 – 6.91 (m, 4H), 6.75 (s, 2H), 6.70 (s, 2H), 6.59 (s, 2H), 6.47 (s, 2H), 4.62 (d, J = 13.7 Hz, 2H), 3.97 (d, J = 13.7 Hz, 2H), 3.90 – 3.73 (m, 6H), 3.68 (s, 6H), 3.55 (s, 6H), 3.41 (s, 6H), 3.30 (s, 6H), 1.38 (s, 18H) ppm. $^{13}C\{^1H\}$ NMR (101 MHz, chloroform-*d*): δ 166.5 (2C), 151.4 (2C), 151.2 (2C), 151.0 (2C), 150.9 (2C), 149.0 (2C), 148.6 (2C), 140.3 (2C), 134.1 (2C), 132.2 (2C), 129.1 (2C), 129.0 (2C), 128.2 (2C), 127.5 (2C), 126.5 (4C), 121.2 (4C), 114.6 (2C), 114.4 (2C), 113.98 (2C), 113.96 (2C), 56.3 (2C), 56.02 (2C), 55.99 (2C), 55.3 (2C), 34.7 (2C), 33.7 (2C), 31.60 (6C), 30.1 (2C), 29.2 (1C) ppm. IR (ATR): ν = 1736 (C=O, esters) cm^{-1}. HRMS (ESI+) *m/z*: calcd. for $C_{65}H_{70}O_{12}Na$ $[M + Na]^+$: 1065.4765, found: 1065.4769.

Di([1,1'-biphenyl]-4-yl) (*pS*)-$3^2,3^5,5^2,5^5,7^2,7^5,9^2,9^5$-octamethoxy-1,3,5,7,9(1,4)-pentabenzenacyclodecaphane-$1^2,1^5$-dicarboxylate (4e)

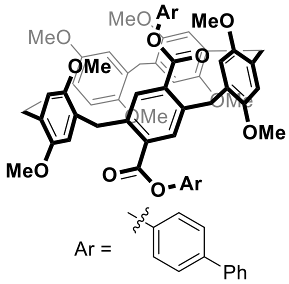

The title compound was synthesized according to the general procedure A (reaction time: 40 h), using pillar[5]arene **1a** (37.3 mg, 0.05 mmol) and 4-phenylphenol (18.7 mg, 0.11 mmol). The crude product was purified by column chromatography (hexane/DCM/EtOAc – 10:1:1), affording **4e** (48.8 mg, 90%) as a white foam.

Er = 93:7 (*ee* = 86%), the enantiomeric excess of product **4e** was determined by HPLC on a Chiralpak® IC column (*n*-heptane/*i*-PrOH – 70:30, flow rate = 1.0 ml/min, λ = 190 nm, t = 25 °C): t_R = 11.0 min (major), t_R = 20.4 min (minor). $[\alpha]_D^{20}$ = -13.1 (c = 2.1, $CHCl_3$). ^1H NMR (400 MHz, chloroform-*d*): δ 8.05 (s, 2H), 7.74 – 7.61 (m, 8H), 7.51 (dd, J = 8.3, 6.9 Hz, 4H), 7.45 – 7.36 (m, 2H), 7.19 – 7.10 (m, 4H), 6.77 (s, 2H), 6.76 (s, 2H), 6.66 (s, 2H), 6.47 (s, 2H), 4.69 (d, J = 13.7 Hz, 2H), 4.12 – 3.75 (m, 8H), 3.70 (s, 6H), 3.60 (s, 6H), 3.49 (s, 6H), 3.29 (s, 6H) ppm. $^{13}C\{^1H\}$ NMR (101 MHz, chloroform-*d*): δ 166.4 (2C), 151.4 (2C), 151.2 (2C), 151.0 (2C), 150.9 (2C), 150.3 (2C), 140.5 (2C), 140.4 (2C), 139.3 (2C), 134.2 (2C), 132.1 (2C), 129.20 (2C), 129.16 (2C), 129.0 (4C), 128.3 (4C), 128.1 (2C), 127.6 (2C), 127.4 (2C), 127.3 (4C), 122.2 (4C), 114.6 (2C), 114.3 (2C), 114.0 (2C), 113.9 (2C), 56.3 (2C), 56.1 (2C), 56.0 (2C), 55.3 (2C), 33.9 (2C), 30.0 (2C), 29.2 (1C) ppm. IR (ATR): ν = 1736 (C=O, esters) cm^{-1}. HRMS (ESI+) *m/z*: calcd. for $C_{69}H_{62}O_{12}Na$ $[M + Na]^+$: 1105.4134, found: 1105.4138.

Dimethyl $3^2,3^5,5^2,5^5,7^2,7^5,9^2,9^5$-octamethoxy-1,3,5,7,9(1,4)-pentabenzenacyclodecaphane-$1^2,1^5$-dicarboxylate (4f)

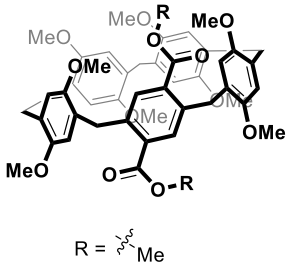

The title compound was synthesized according to the general procedure A (reaction time: 24 h), using pillar[5]arene **1a** (37.3 mg, 0.05 mmol) and methanol (4.5 γL, 0.11 mmol). The crude product was purified by column chromatography (hexane/EtOAc – 3:1), affording **4f** (28.6 mg, 71%) as a yellow foam.

Note: We analyzed *rac*-**4f** by chiral HPLC on chiral columns used in this study for enantiomer separations (IA, IC and IG), but we detected only one peak without any separation.

$[\alpha]_D^{20}$ = 0 (*c* = 2.7, $CHCl_3$). ^{1}H NMR (400 MHz, chloroform-*d*): *δ* 7.65 (s, 2H), 6.72 (s, 2H), 6.66 (s, 2H), 6.65 (s, 2H), 6.52 (s, 2H), 4.19 (s, 4H), 3.80 (d, *J* = 12.7 Hz, 6H), 3.73 (s, 6H), 3.64 (s, 6H), 3.61 (s, 6H), 3.59 (s, 6H), 3.33 (s, 6H) ppm. ^{13}C{^{1}H} NMR (101 MHz, chloroform-*d*): *δ* 168.4 (2C), 151.24 (2C), 151.19 (2C), 151.1 (2C), 151.0 (2C), 139.7 (2C), 133.4 (2C), 132.5 (2C), 129.1 (2C), 128.9 (2C), 128.3 (2C), 127.8 (2C), 114.4 (2C), 114.29 (2C), 114.25 (2C), 114.1 (2C), 56.2 (2C), 56.12 (2C), 56.06 (2C), 55.6 (2C), 51.9 (2C), 33.4 (2C), 29.9 (1C), 29.8 (2C) ppm. IR (ATR): *ν* = 1718 (C=O, esters) cm^{-1}. HRMS (ESI+) *m*/*z*: calcd. for $C_{47}H_{50}O_{12}Na$ [M + Na]$^+$: 829.3194, found: 829.3196.

Didodecyl $3^2,3^5,5^2,5^5,7^2,7^5,9^2,9^5$-octamethoxy-1,3,5,7,9(1,4)-pentabenzenacyclodecaphane-$1^2,1^5$-dicarboxylate (4i)

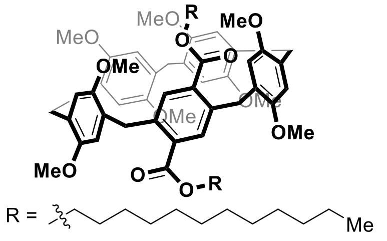

The title compound was synthesized according to the general procedure A (reaction time: 72 h, no full conversion of starting material or corresponding monoester was observed), using pillar[5]arene **1a** (37.3 mg, 0.05 mmol) and lauryl alcohol (20.5 mg, 0.11 mmol). The crude product was purified by column chromatography (hexane/EtOAc – 10:1), affording **4i** (10.3 mg, 18%) as a white foam.

Note: We analyzed *rac*-**4i** by chiral HPLC on chiral columns used in this study for enantiomer separations (IA, IC and IG), but we detected only one peak without any separation.

$[\alpha]_D^{20}$ = 0 (*c* = 1.0, $CHCl_3$). ^{1}H NMR (400 MHz, chloroform-*d*): *δ* 7.85 (s, 2H), 6.76 (s, 2H), 6.75 (s, 2H), 6.68 (s, 2H), 6.56 (s, 2H), 4.26 (t, *J* = 6.7 Hz, 4H), 3.78 (d, *J* = 10.0 Hz, 6H), 3.66 (s, 6H), 3.62 (d, *J* = 1.0 Hz, 12H), 3.44 (s, 6H), 1.74 (p, *J* = 6.9 Hz, 4H), 1.56 – 0.94 (m, 40H), 0.84 (t, *J* = 7.2 Hz, 6H) ppm. ^{13}C{^{1}H} NMR (101 MHz, chloroform-*d*): *δ* 168.0 (2C), 151.00 (4C), 150.97 (4C), 140.0 (2C), 134.1 (2C), 132.4 (2C), 128.9 (2C), 128.7 (2C), 128.1 (2C), 127.9 (2C), 114.2 (2C), 114.1 (2C), 114.0 (2C), 113.6 (2C), 65.2 (2C), 56.0 (2C), 55.92 (2C), 55.88 (2C), 55.5 (2C), 32.7 (2C), 32.0 (2C), 29.8 (1C), 29.7 (4C), 29.6 (4C), 29.54 (2C), 29.45 (2C), 29.3 (2C), 28.7 (2C), 26.1 (2C), 22.8 (2C), 14.3 (2C) ppm. IR (ATR): *ν* = 1711 (C=O, esters) cm^{-1}. HRMS (ESI+) *m*/*z*: calcd. for $C_{69}H_{94}O_{12}Na$ [M + Na]$^+$: 1137.6643, found: 1137.6645.

Bis(3-phenylpropyl) $3^2,3^5,5^2,5^5,7^2,7^5,9^2,9^5$-octamethoxy-1,3,5,7,9(1,4)-pentabenzenacyclodecaphane-$1^2,1^5$-dicarboxylate (4j)

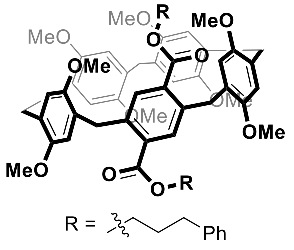

The title compound was synthesized according to the general procedure A (reaction time: 72 h, no full conversion of starting material or corresponding monoester was observed), using pillar[5]arene **1a** (37.3 mg, 0.05 mmol) and 3-phenylpropanol (15.0 mg, 0.11 mmol). The crude product was purified by column chromatography (hexane/EtOAc – 5:1), affording **4j** (13.3 mg, 26%) as a white foam.

Note: We analyzed *rac*-**4j** by chiral HPLC on chiral columns used in this study for enantiomer separations (IA, IC and IG), but we detected only one peak without any separation.
$[\alpha]_D^{20}$ = 0 (*c* = 1.2, $CHCl_3$). ^{1}H NMR (400 MHz, chloroform-*d*): δ 7.81 (s, 2H), 7.34 – 7.27 (m, 4H), 7.25 – 7.17 (m, 6H), 6.73 (s, 2H), 6.71 (s, 2H), 6.62 (s, 2H), 6.45 (s, 2H), 4.62 (d, *J* = 13.4 Hz, 2H), 4.28 (d, *J* = 7.1 Hz, 4H), 3.87 (d, *J* = 13.3 Hz, 2H), 3.79 (d, *J* = 13.2 Hz, 6H), 3.60 (s, 6H), 3.57 (s, 6H), 3.57 (s, 6H), 3.29 (s, 6H), 2.76 (dd, *J* = 8.6, 6.7 Hz, 4H), 2.06 (dq, *J* = 7.9, 6.7 Hz, 4H) ppm. $^{13}C\{^1H\}$ NMR (101 MHz, chloroform-*d*): δ 168.0 (2C), 151.2 (2C), 151.12 (2C), 151.08 (2C), 151.05 (2C), 141.2 (2C), 139.8 (2C), 133.9 (2C), 132.6 (2C), 129.0 (2C), 128.8 (2C), 128.7 (4C), 128.5 (4C), 128.0 (2C), 127.9 (2C), 126.3 (2C), 114.3 (4C), 114.1 (2C), 113.7 (2C), 64.4 (2C), 56.10 (2C), 56.05 (2C), 56.0 (2C), 55.5 (2C), 33.2 (2C), 32.4 (2C), 30.5 (2C), 30.0 (1C), 29.7 (2C) ppm. IR (ATR): ν = 1710 (C=O, esters) cm^{-1}. HRMS (ESI+) *m*/*z*: calcd. for $C_{63}H_{66}O_{12}Na$ $[M + Na]^+$: 1037.4446, found: 1037.4444.

Bis((8*R*,9*S*,13*S*,14*S*)-13-methyl-17-oxo-7,8,9,11,12,13,14,15,16,17-decahydro-6*H*-cyclopenta[*a*]phenanthren-3-yl) (*pS*)-3^2,3^5,5^2,5^5,7^2,7^5,9^2,9^5-octamethoxy-1,3,5,7,9(1,4)-pentabenzenacyclodecaphane-1^2,1^5-dicarboxylate (4k)

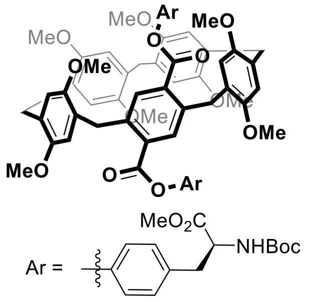

The title compound was synthesized according to the general procedure A (reaction time: 18 h), using pillar[5]arene **1a** (37.3 mg, 0.05 mmol) and Boc-L-Tyr-OMe (32.5 mg, 0.11 mmol). The crude product was purified by column chromatography (hexane/EtOAc – 2:1), affording **4k** (58.2 mg, 87%) as a white foam. The diastereomeric ratio of **4k**/**4k´** = 9:1 was determined by ^{1}H NMR of the crude reaction mixture (refer to the NMR spectra section).
$[\alpha]_D^{20}$ = +13.9 (*c* = 1.6, $CHCl_3$). ^{1}H NMR (400 MHz, chloroform-*d*, only major diastereomer): δ 7.97 (s, 2H), 7.20 (d, *J* = 8.1 Hz, 4H), 7.00 (t, *J* = 5.3 Hz, 4H), 6.72 (s, 2H), 6.69 (d, *J* = 3.3 Hz, 2H), 6.65 (d, *J* = 5.0 Hz, 2H), 6.40 (d, *J* = 4.0 Hz, 2H), 5.03 (d, *J* = 8.2 Hz, 2H), 4.69 – 4.55 (m, 4H), 3.94 (d, *J* = 13.7 Hz, 2H), 3.90 – 3.82 (m, 4H), 3.76 (s, 8H), 3.67 (s, 6H), 3.56 (s, 6H), 3.46 (s, 6H), 3.25 – 3.03 (m, 10H), 1.46 (s, 18H) ppm. $^{13}C\{^1H\}$ NMR (101 MHz, chloroform-*d*, only major diastereomer): δ 172.3 (2C), 166.2 (2C), 155.2 (2C), 151.4 (2C), 151.1 (2C), 151.0 (2C), 150.9 (2C), 149.9 (2C), 140.3 (2C), 134.2 (2C), 134.0 (2C), 132.1 (2C), 130.5 (4C), 129.20 (2C), 129.17 (2C), 128.0 (2C), 127.3 (2C), 121.9 (4C), 114.7 (2C), 114.3 (2C), 114.0 (2C), 113.8 (2C), 80.2 (2C), 56.3 (2C), 56.1 (2C), 56.0 (2C), 55.2 (2C), 54.6 (2C), 52.4 (2C), 37.8 (2C), 33.9 (2C), 29.9 (2C), 29.1 (1C), 28.4 (6C) ppm. IR (ATR): ν = 1738, 1714 (C=O, esters), 1607 (C=O, amides), 3371 (N-H) cm^{-1}. HRMS (ESI+) *m*/*z*: calcd. for $C_{75}H_{84}O_{20}N_2Na$ $[M + Na]^+$: 1355.5510, found: 1355.5513.

Bis((8*R*,9*S*,13*S*,14*S*)-13-methyl-17-oxo-7,8,9,11,12,13,14,15,16,17-decahydro-6*H*-cyclopenta[*a*]phenanthren-3-yl) (*pS*)-3^2,3^5,5^2,5^5,7^2,7^5,9^2,9^5-octamethoxy-1,3,5,7,9(1,4)-pentabenzenacyclodecaphane-1^2,1^5-dicarboxylate (4l)

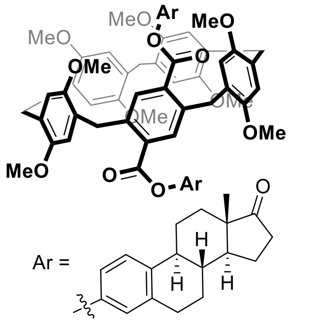

The title compound was synthesized according to the general procedure A (reaction time: 18 h), using pillar[5]arene **1a** (37.3 mg, 0.05 mmol) and estrone (29.7 mg, 0.11 mmol). The crude product was purified by column chromatography (hexane/EtOAc – 3:1 to 2:1), affording **4l** (59.6 mg, 93%) as a white foam. The diastereomeric ratio of **4l**/**4l´** = 10:1 was determined by ^{1}H NMR of the crude reaction mixture (refer to the NMR spectra section).
$[\alpha]_D^{20}$ = +50.0 (*c* = 0.9, $CHCl_3$). ^{1}H NMR (400 MHz, chloroform-*d*, only major diastereomer): δ 8.02 (s, 2H), 7.35 (d, *J* = 8.4 Hz, 2H), 6.89 – 6.81 (m, 4H), 6.75 (s, 2H), 6.70 (s, 2H), 6.67 (s, 2H), 6.38 (s, 2H), 4.66 (d, *J* = 13.6 Hz, 2H), 3.97 – 3.72 (m, 8H), 3.64 (s, 6H), 3.61 (s, 6H), 3.50 (s, 6H), 3.21 (s, 6H), 3.01 – 2.92 (m,

4H), 2.61 – 2.51 (m, 2H), 2.51 – 2.43 (m, 2H), 2.34 (td, *J* = 10.8, 4.1 Hz, 2H), 2.28 – 1.88 (m, 8H), 1.77 – 1.38 (m, 12H), 0.95 (s, 6H) ppm. ^{13}C{^{1}H} NMR (101 MHz, chloroform-*d*, only major diastereomer): δ 220.8 (2C), 166.6 (2C), 151.3 (2C), 151.2 (2C), 151.1 (2C), 150.9 (2C), 148.8 (2C), 140.3 (2C), 138.3 (2C), 137.7 (2C), 134.4 (2C), 132.1 (2C), 129.2 (2C), 129.1 (2C), 128.0 (2C), 127.5 (2C), 126.6 (2C), 121.9 (2C), 119.0 (2C), 114.6 (2C), 114.3 (2C), 114.1 (2C), 113.8 (2C), 56.22 (2C), 56.16 (2C), 56.0 (2C), 55.2 (2C), 50.6 (2C), 48.1 (2C), 44.3 (2C), 38.2 (2C), 36.0 (2C), 33.8 (2C), 31.7 (2C), 29.7 (2C), 29.6 (2C), 29.4 (1C), 26.5 (2C), 26.0 (2C), 21.7 (2C), 14.0 (2C) ppm. IR (ATR): ν = 1734 (C=O, esters) cm^{-1}. HRMS (ESI+) *m*/*z*: calcd. for $C_{81}H_{86}O_{14}Na$ [M + Na]$^{+}$: 1305.5910, found: 1305.5907.

Bis(4-((2*S*,3*R*)-3-((*S*)-3-((*tert*-butyldimethylsilyl)oxy)-3-(4-fluorophenyl)propyl)-1-(4-fluorophenyl)-4-oxoazetidin-2-yl)phenyl) (*pS*)-$3^2,3^5,5^2,5^5,7^2,7^5,9^2,9^5$-octamethoxy-1,3,5,7,9(1,4)-pentabenzenacyclodecaphane-$1^2,1^5$-dicarboxylate (4m)

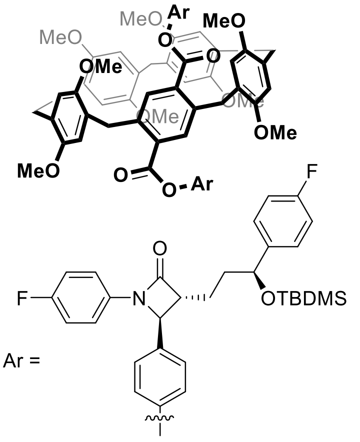

The title compound was synthesized according to the general procedure A (reaction time: 18 h), using pillar[5]arene **1a** (37.3 mg, 0.05 mmol) and TBDMS-ezetimibe (57.6 mg, 0.11 mmol). The crude product was purified by column chromatography (first column: hexane/EtOAc – 5:1, second column: hexane/DCM/EtOAc – 10:4:1), affording **4m** (68.2 mg, 76%) as a white foam. The diastereomeric ratio of **4m**/**4m´** = 10:1 was determined by ^{1}H NMR of the crude reaction mixture (refer to the NMR spectra section).

$[\alpha]_D^{20}$ = -21.3 (*c* = 1.4, $CHCl_3$). ^{1}H NMR (400 MHz, chloroform-*d*, only major diastereomer): δ 7.90 (s, 2H), 7.42 – 7.33 (m, 4H), 7.26 (td, *J* = 5.9, 2.5 Hz, 8H), 7.05 – 6.92 (m, 12H), 6.70 (s, 2H), 6.60 (s, 2H), 6.52 (s, 2H), 6.40 (s, 2H), 4.70 (t, *J* = 5.7 Hz, 2H), 4.63 (d, *J* = 2.4 Hz, 2H), 4.57 (d, *J* = 13.8 Hz, 2H), 3.92 (d, *J* = 13.8 Hz, 2H), 3.86 – 3.69 (m, 6H), 3.66 (s, 6H), 3.38 (s, 6H), 3.36 (s, 6H), 3.20 (s, 6H), 3.08 (td, *J* = 7.5, 2.4 Hz, 2H), 2.01 – 1.90 (m, 4H), 1.86 (td, *J* = 6.7, 2.9 Hz, 4H), 0.90 (s, 18H), 0.04 (s, 6H), -0.13 (s, 6H) ppm. ^{13}C{^{1}H} NMR (101 MHz, chloroform-*d*, only major diastereomer): δ 167.2 (2C), 166.1 (2C), 162.1 (d, *J* = 244.8 Hz, 2C), 159.2 (d, *J* = 243.8 Hz, 2C), 151.5 (2C), 151.1 (2C), 151.0 (2C), 150.92 (2C), 150.90 (2C), 140.8 (d, *J* = 3.0 Hz, 2C), 140.4 (2C), 135.6 (2C), 134.0 (2C), 133.9 (d, *J* = 2.6 Hz, 2C), 132.0 (2C), 129.3 (2C), 129.2 (2C), 128.1 (2C), 127.4 (d, *J* = 7.9 Hz, 4C), 127.2 (4C), 127.1 (2C), 122.8 (4C), 118.5 (d, *J* = 7.8 Hz, 4C), 116.0 (d, *J* = 22.7 Hz, 4C), 115.2 (d, *J* = 21.3 Hz 4C), 114.8 (2C), 114.3 (2C), 114.0 (2C), 113.9 (2C), 74.1 (2C), 61.0 (2C), 56.4 (2C), 55.94 (2C), 55.86 (2C), 55.2 (2C), 38.4 (2C), 34.1 (2C), 30.1 (2C), 29.1 (1C), 26.0 (6C), 24.9 (2C), 18.3 (2C), -4.50 (2C), -4.80 (2C) ppm. ^{19}F NMR (376 MHz, chloroform-*d*, only major diastereomer): δ -115.61 (tt, *J* = 8.8, 5.3 Hz, 2F), -117.80 (tq, *J* = 8.4, 4.3 Hz, 2F) ppm. IR (ATR): ν = 1747 (C=O, esters), 1604 (C=O, amides) cm^{-1}. HRMS (ESI+) *m*/*z*: calcd. for $C_{105}H_{112}O_{16}N_2NaSi_2$ [M + Na]$^{+}$: 1811.7379, found: 1811.7394.

Bis(4-((2*S*,3*R*)-1-(4-fluorophenyl)-3-((*S*)-3-(4-fluorophenyl)-3-hydroxypropyl)-4-oxoazetidin-2-yl)phenyl) (*pS*)-$3^2,3^5,5^2,5^5,7^2,7^5,9^2,9^5$-octamethoxy-1,3,5,7,9(1,4)-pentabenzenacyclodecaphane-$1^2,1^5$-dicarboxylate (4n)

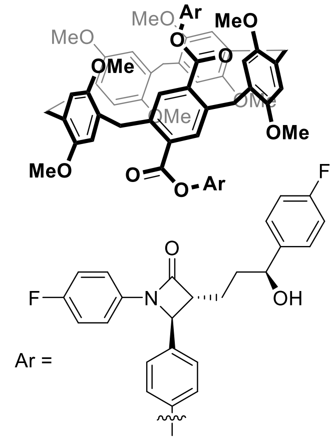

The title compound was synthesized according to the general procedure A (reaction time: 24 h), using pillar[5]arene **1a** (37.3 mg, 0.05 mmol) and ezetimibe (45.0 mg, 0.11 mmol). The crude product was purified by column chromatography (hexane/EtOAc – 2:1), affording **4n** (62.3 mg, 80%) as a white foam. The diastereomeric ratio of **4n**/**4n´** = 14:1 was determined by ^{1}H NMR of the crude reaction mixture (refer to the NMR spectra section).

$[\alpha]_D^{20}$ = -26.9 (*c* = 0.8, $CHCl_3$). ^{1}H NMR (400 MHz, chloroform-*d*, only major diastereomer): δ 7.90 (s, 2H), 7.42 – 7.36 (m, 4H), 7.34 – 7.24 (m, 8H), 7.07 – 6.94 (m, 12H), 6.69 (s, 2H), 6.60 (s, 2H), 6.52 (s, 2H), 6.40 (s, 2H), 4.75 (t, *J* = 6.1 Hz, 2H), 4.68 (d, *J* = 2.3 Hz, 2H), 4.57 (d, *J* = 13.7 Hz, 2H), 3.92 (d, *J* = 13.8 Hz, 2H), 3.86 – 3.69 (m, 6H), 3.64 (s, 6H), 3.39 (s, 6H), 3.36 (s, 6H), 3.20 (s, 6H), 3.14 (td, *J* = 7.4, 2.4 Hz, 2H), 2.26 – 2.19 (m, 2H), 2.02 – 1.90 (m, 6H), 1.58 (br s, 2H) ppm. $^{13}C\{^1H\}$ NMR (101 MHz, chloroform-*d*, only major diastereomer): δ 167.4 (2C), 166.1 (2C), 162.4 (d, *J* = 245.8 Hz, 2C), 159.2 (d, *J* = 243.9 Hz, 2c), 151.5 (2C), 151.1 (2C), 151.04 (2C), 150.99 (2C), 150.9 (2C), 140.5 (2C), 140.1 (d, *J* = 3.1 Hz, 2C), 135.5 (2C), 134.1 (2C), 133.9 (d, *J* = 2.5 Hz, 2C), 132.0 (2C), 129.3 (2C), 129.2 (2C), 128.1 (2C), 127.5 (d, *J* = 8.1 Hz, 4C), 127.21 (4C), 127.16 (2C), 122.9 (4C), 118.5 (d, *J* = 7.8 Hz, 4C), 116.1 (d, *J* = 22.7 Hz, 4C), 115.6 (d, *J* = 21.3 Hz, 4C), 114.8 (2C), 114.3 (2C), 114.0 (2C), 113.9 (2C), 73.4 (2C), 61.1 (2C), 60.8 (2C), 56.5 (2C), 56.0 (2C), 55.9 (2C), 55.3 (2C), 36.8 (2C), 34.1 (2C), 30.1 (2C), 29.1 (1C), 25.3 (2C) ppm. ^{19}F NMR (376 MHz, chloroform-*d*, only major diastereomer): δ -114.70 (tt, *J* = 8.6, 5.3 Hz, 2F), -117.61 (qt, *J* = 8.5, 4.2 Hz, 2F) ppm. IR (ATR): ν = 3444 (O-H), 1738 (C=O, esters), 1666 (C=O, amides) cm^{-1}. HRMS (ESI+) *m*/*z*: calcd. for $C_{93}H_{84}O_{16}N_2F_4Na$ $[M + Na]^+$: 1583.5649, found: 1583.5663.

Naphthalen-2-yl (*pS*)-1^5-([1,1'-biphenyl]-4-yl)-$3^2,3^5,5^2,5^5,7^2,7^5,9^2,9^5$-octamethoxy-1,3,5,7,9(1,4)-pentabenzenacyclodecaphane-1^2-carboxylate (5a)

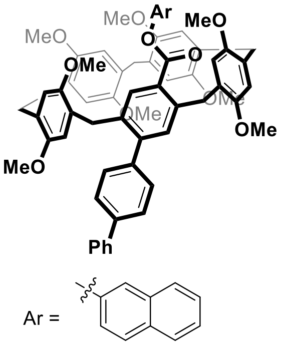

The title compound was synthesized according to the general procedure B (reaction time: 72 h, without full conversion of the aldehyde), using pillar[5]arene **1d** (43.6 mg, 0.05 mmol) and naphthalen-2-ol (8.7 mg, 0.06 mmol). The crude product was purified by column chromatography (hexane/EtOAc – 10:1-5:1), affording **5a** (32.0 mg, 62%) as a pale oil.

Er = 89:11 (*ee* = 77%), the enantiomeric excess of product **5a** was determined by HPLC on a Chiralpak® IA column (*n*-heptane/*i*-PrOH – 50:50, flow rate = 1.0 ml/min, λ = 286 nm, *t* = 25 °C): t_R = 6.5 min (major), t_R = 11.2 min (minor). $[\alpha]_D^{20}$ = +50.3 (*c* = 0.7, $CHCl_3$). ^{1}H NMR (400 MHz, chloroform-*d*): δ 8.15 (s, 1H), 7.96 – 7.84 (m, 3H), 7.70 – 7.63 (m, 2H), 7.58 – 7.48 (m, 7H), 7.46 – 7.39 (m, 1H), 7.28 – 7.25 (m, 1H), 7.15 (s, 1H), 7.07 (d, *J* = 7.8 Hz, 2H), 6.77 – 6.73 (m, 3H), 6.72 (s, 1H), 6.63 (s, 1H), 6.52 (s, 1H), 6.50 (s, 1H), 5.80 (s, 1H), 4.72 (d, *J* = 14.2 Hz, 1H), 3.98 (q, *J* = 6.9 Hz, 3H), 3.94 – 3.70 (m, 6H), 3.68 (s, 3H), 3.65 (s, 3H), 3.56 (s, 3H), 3.51 (s, 3H), 3.49 (s, 3H), 3.37 (s, 3H), 3.33 (s, 3H), 3.30 (s, 3H) ppm. $^{13}C\{^1H\}$ NMR (101 MHz, chloroform-*d*): δ 166.9, 151.4 (2C), 151.3 (2C), 151.09, 151.07, 150.8, 150.6, 148.9, 145.2, 141.2, 140.8, 140.7, 139.9, 136.8, 134.0, 133.8, 133.6, 131.6, 129.6 (2C), 129.5, 129.2, 129.1 (2C), 128.87, 128.85, 128.7, 128.4, 128.3, 128.1, 127.9, 127.8, 127.6, 127.5, 127.4, 127.1

(2C), 126.9 (2C), 126.7, 125.8, 121.6, 119.0, 114.7, 114.47, 114.45, 114.4, 114.24, 114.20, 114.15, 113.4, 56.22, 56.17, 56.11, 56.09, 56.0, 55.64, 55.55, 55.5, 33.94, 33.90, 30.2, 30.1, 29.5 ppm. IR (ATR): ν = 1732 (C=O, ester) cm^{-1}. HRMS (ESI+) *m*/*z*: calcd. for $C_{66}H_{60}O_{10}Na$ $[M + Na]^+$: 1035.4079, found: 1035.4081.

[1,1'-Biphenyl]-4-yl (*pS*)-1^5-([1,1'-biphenyl]-4-yl)-3^2,3^5,5^2,5^5,7^2,7^5,9^2,9^5-octamethoxy-1,3,5,7,9(1,4)-pentabenzenacyclodecaphane-1^2-carboxylate (5b)

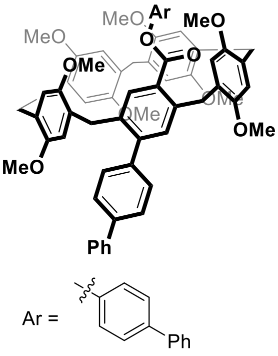

The title compound was synthesized according to the general procedure B (reaction time: 72 h, without full conversion of the aldehyde), using pillar[5]arene **1d** (43.6 mg, 0.05 mmol) and 4-phenylphenol (10.2 mg, 0.06 mmol). The crude product was purified by column chromatography (hexane/EtOAc – 10:1-5:1, second column: hexane/DCM/EtOAc – 10:4:1), affording **5b** (32.0mg, 62%) as a pale oil.

Er = 90:10 (*ee* = 80%), the enantiomeric excess of product **5b** was determined by HPLC on a Chiralpak® IA column (*n*-heptane/*i*-PrOH – 50:50, flow rate = 1.0 ml/min, λ = 282 nm, t = 25 °C): t_R = 6.5 min (major), t_R = 14.0 min (minor). $[\alpha]_D^{20}$ = +55.2 (c = 0.5, $CHCl_3$). ^1H NMR (400 MHz, chloroform-*d*): δ 8.08 (s, 1H), 7.65 (ddt, J = 9.3, 8.1, 1.6 Hz, 6H), 7.56 – 7.46 (m, 6H), 7.45 – 7.37 (m, 2H), 7.19 – 7.11 (m, 3H), 7.05 (d, J = 7.9 Hz, 2H), 6.77 – 6.70 (m, 4H), 6.61 (s, 1H), 6.56 (s, 1H), 6.52 (s, 1H), 5.81 (s, 1H), 4.70 (d, J = 14.2 Hz, 1H), 4.03 – 3.89 (m, 4H), 3.88 – 3.81 (m, 3H), 3.73 (d, J = 3.0 Hz, 2H), 3.68 (s, 3H), 3.67 (s, 3H), 3.58 (s, 3H), 3.50 (s, 6H), 3.38 (s, 3H), 3.36 (s, 3H), 3.33 (s, 3H) ppm. $^{13}C\{^1H\}$ NMR (101 MHz, chloroform-*d*): δ 166.8, 151.4, 151.31, 151.25, 151.2, 151.09, 151.05, 150.8, 150.7, 150.6, 145.2, 141.2, 140.8, 140.7, 140.6, 139.9, 139.0, 136.8, 133.7, 133.5, 129.6 (2C), 129.1 (2C), 129.0 (2C), 128.8, 128.7, 128.5, 128.4, 128.3 (2C), 128.0, 127.6, 127.52, 127.47, 127.4, 127.3 (2C), 127.1 (2C), 126.9 (2C), 122.3 (2C), 114.7, 114.43, 114.39, 114.3, 114.23, 114.20, 113.4, 56.2 (2C), 56.14, 56.08, 56.0, 55.64, 55.58, 55.55, 33.9, 33.8, 30.3, 30.2, 29.9, 29.5 ppm. IR (ATR): ν = 1732 (C=O, ester) cm^{-1}. HRMS (ESI+) *m*/*z*: calcd. for $C_{68}H_{62}O_{10}Na$ $[M + Na]^+$: 1061.4235, found: 1061.4240.

(8*R*,9*S*,13*S*,14*S*)-1^3-Methyl-17-oxo-7,8,9,11,12,13,14,15,16,17-decahydro-6*H*-cyclopenta[*a*]phenanthren-3-yl (*pS*)-1^5-([1,1'-biphenyl]-4-yl)-3^2,3^5,5^2,5^5,7^2,7^5,9^2,9^5-octamethoxy-1,3,5,7,9(1,4)-pentabenzenacyclodecaphane-1^2-carboxylate (5c)

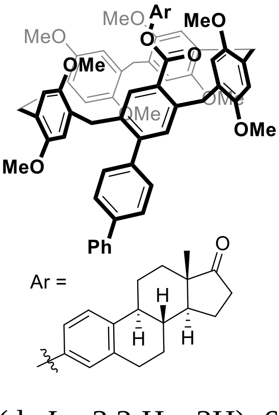

The title compound was synthesized according to the general procedure B (reaction time: 72 h, without full conversion of the aldehyde), using pillar[5]arene **1d** (43.6 mg, 0.05 mmol) and estrone (16.2 mg, 0.06 mmol). The crude product was purified by column chromatography (hexane/EtOAc – 10:1-3:1), affording **5c** (36.4 mg, 64%) as a white foam. The diastereomeric ratio of **5c**/**5c´** = 9:1 was determined by ^1H NMR of the crude reaction mixture (see NMR spectra section).

$[\alpha]_D^{20}$ = +57.6 (c = 0.8, $CHCl_3$). ^1H NMR (400 MHz, chloroform-*d*, only major diastereomer): δ 8.10 (s, 1H), 7.67 – 7.61 (m, 2H), 7.54 – 7.48 (m, 2H), 7.47 – 7.43 (m, 2H), 7.43 – 7.38 (m, 1H), 7.36 (d, J = 8.3 Hz, 1H), 7.07 (s, 1H), 7.02 – 6.96 (m, 2H), 6.92 – 6.86 (m, 2H), 6.74 (d, J = 3.2 Hz, 2H), 6.72 (d, J = 1.6 Hz, 2H), 6.62 (s, 1H), 6.52 (s, 1H), 6.49 (s, 1H), 5.74 (s, 1H), 4.70 (s, 1H), 4.00 – 3.69 (m, 9H), 3.67 (s, 3H), 3.63 (s, 3H), 3.61 (s, 3H), 3.53 (s, 3H), 3.50 (s, 3H), 3.35 (s, 3H), 3.32 (s, 3H), 3.29 (s, 3H), 2.98 (dd, J = 9.0, 4.2 Hz, 2H), 2.54 (dd, J = 18.7, 8.6 Hz, 1H), 2.50 – 2.43 (m, 1H), 2.41 – 1.97 (m, 5H), 1.72 – 1.49 (m, 6H), 0.95 (s, 3H) ppm. $^{13}C\{^1H\}$ NMR (101 MHz, chloroform-*d*, only major enantiomer): δ 220.9, 167.0, 151.4,

151.3, 151.24, 151.19, 151.1 (2C), 150.8, 150.6, 149.1, 145.2, 141.2, 140.8, 140.7, 139.8, 138.2, 137.5, 136.7, 133.6 (2C), 129.6 (2C), 129.11, 129.06 (2C), 128.80, 128.75, 128.6, 128.5, 128.4, 128.1, 127.6, 127.5, 127.4, 127.1 (2C), 126.8 (2C), 126.6, 122.0, 119.2, 114.7, 114.42, 114.40, 114.3, 114.21, 114.19, 114.17, 113.4, 56.20, 56.15, 56.14, 56.10, 56.0, 55.64, 55.55, 55.5, 50.6, 48.1, 44.4, 38.2, 36.0, 33.9, 33.6, 31.7, 30.1 (2C), 29.61, 29.56, 26.5, 26.0, 21.8, 14.0 ppm. IR (ATR): ν = 1734 (C=O, esters) cm^{-1}. HRMS (ESI+) *m*/*z*: calcd. for $C_{74}H_{74}O_{11}Na$ $[M + Na]^+$: 1161.5123, found: 1161.5126.

1^2-([1,1'-Biphenyl]-4-yl) 1^5-(naphthalen-2-yl) (*pS*)-3^2,3^5,5^2,5^5,7^2,7^5,9^2,9^5-octamethoxy-1,3,5,7,9(1,4)-pentabenzenacyclodecaphane-1^2,1^5-dicarboxylate (5d)

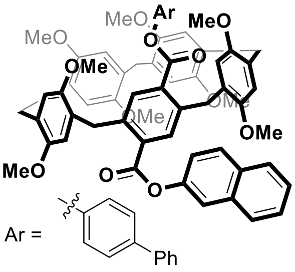

The title compound was synthesized according to the general procedure B (reaction time: 18 h), using pillar[5]arene **1e** (44.5 mg, 0.05 mmol) and 4-phenylphenol (10.2 mg, 0.06 mmol). The crude product was purified by column chromatography (first column: hexane/EtOAc – 5:1, second column: hexane/DCM/EtOAc – 10:4:1), affording **5d** (52.0 mg, 98%) as a white foam.

Er = 93:7 (*ee* = 79%), the enantiomeric excess of product **5d** was determined by HPLC on a Chiralpak® IC column (*n*-heptane/*i*-PrOH – 70:30, flow rate = 1.0 ml/min, λ = 190 nm, *t* = 25 °C): t_R = 9.9 min (major), t_R = 16.3 min (minor). $[\alpha]_D^{20}$ = -6.1 (*c* = 1.5, $CHCl_3$). ^1H NMR (400 MHz, chloroform-*d*): δ 8.11 (t, *J* = 1.1 Hz, 1H), 8.04 (t, *J* = 1.2 Hz, 1H), 7.97 – 7.87 (m, 3H), 7.66 (ddd, *J* = 13.7, 8.0, 1.3 Hz, 4H), 7.60 – 7.46 (m, 5H), 7.45 – 7.37 (m, 1H), 7.26 – 7.22 (m, 1H), 7.14 (dt, *J* = 8.7, 1.2 Hz, 2H), 6.76 (dq, *J* = 2.5, 1.2 Hz, 4H), 6.66 (dt, *J* = 2.5, 1.2 Hz, 2H), 6.41 (s, 1H), 6.39 (s, 1H), 4.68 (d, *J* = 13.7 Hz, 2H), 4.07 – 3.84 (m, 6H), 3.76 (t, *J* = 13.3 Hz, 2H), 3.69 (s, 3H), 3.68 (s, 3H), 3.58 (s, 3H), 3.54 (s, 3H), 3.49 (s, 3H), 3.48 (s, 3H), 3.25 (s, 3H), 3.20 (s, 3H) ppm. $^{13}C\{^1H\}$ NMR (101 MHz, chloroform-*d*): δ 166.6, 166.4, 151.43, 151.41, 151.3, 151.2, 151.1, 151.0, 150.94, 150.90, 150.3, 148.6, 140.5, 140.39, 140.36, 139.3, 134.4, 134.3, 134.0, 132.2, 132.1, 131.7, 129.6 (2C), 129.32, 129.28 (2C), 129.2, 129.0 (2C), 128.3 (2C), 127.99, 127.98, 127.95, 127.8, 127.6, 127.4, 127.3 (2C), 126.9, 126.0, 122.2 (2C), 121.4, 118.9, 114.7 (2C), 114.32, 114.29, 114.1, 114.0, 113.81, 113.77, 56.31, 56.28, 56.1 (2C), 56.03, 55.96, 55.2 (2C), 34.1, 34.0, 29.9 (2C), 29.1 ppm. IR (ATR): ν = 1734 (C=O, esters) cm^{-1}. HRMS (ESI+) *m*/*z*: calcd. for $C_{67}H_{60}O_{12}Na$ $[M + Na]^+$: 1079.3977, found: 1079.3979.

1^2-((8*R*,9*S*,13*S*,14*S*)-13-methyl-17-oxo-7,8,9,11,12,13,14,15,16,17-decahydro-6*H*-cyclopenta[*a*]phenanthren-3-yl) 1^5-(naphthalen-2-yl) (*pS*)-3^2,3^5,5^2,5^5,7^2,7^5,9^2,9^5-octamethoxy-1,3,5,7,9(1,4)-pentabenzenacyclodecaphane-1^2,1^5-dicarboxylate (5e)

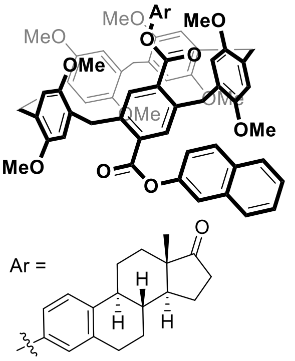

The title compound was synthesized according to the general procedure B (reaction time: 18 h), using pillar[5]arene **1e** (44.5 mg, 0.05 mmol) and estrone (16.2 mg, 0.06 mmol). The crude product was purified by column chromatography (hexane/EtOAc – 4:1), affording **5e** (46.6 mg, 98%) as a white foam. The diastereomeric ratio of **5e**/**5e´** = 12:1 was determined by ^1H NMR of the crude reaction mixture (refer to the NMR spectra section).

$[\alpha]_D^{20}$ = +29.2 (*c* = 1.6, $CHCl_3$). ^1H NMR (400 MHz, chloroform-*d*, only major diastereomer): δ 8.07 (d, *J* = 6.5 Hz, 2H), 7.96 – 7.85 (m, 3H), 7.60 – 7.51 (m, 2H), 7.50 (d, *J* = 2.4 Hz, 1H), 7.37 (d, *J* = 8.5 Hz, 1H), 7.22 (dd, *J* = 8.8, 2.3 Hz, 1H), 6.89 (dd, *J* = 8.3, 2.6 Hz, 1H), 6.85 (d, *J* = 2.6 Hz, 1H), 6.77 (s, 1H), 6.74 (s, 2H), 6.71 (s, 1H), 6.67 (s, 2H), 6.37 (d, *J* = 5.9 Hz, 2H), 4.67 (d, *J* = 13.6 Hz, 2H), 4.03 – 3.83 (m, 6H), 3.75 (dd, *J* = 14.3, 5.4 Hz, 2H), 3.67 (s, 3H), 3.66 (s, 3H), 3.63 (s, 3H), 3.52 (s, 3H), 3.49 (s, 3H), 3.48 (s, 3H), 3.22 (s, 3H), 3.17 (s, 3H), 3.03 – 2.95 (m, 2H),

2.55 (dd, J = 18.7, 8.6 Hz, 1H), 2.50 – 2.44 (m, 1H), 2.34 (qd, J = 11.3, 3.3 Hz, 1H), 2.27 – 2.15 (m, 1H), 2.15 – 1.96 (m, 3H), 1.73 – 1.45 (m, 6H), 0.96 (s, 3H) ppm. $^{13}C\{^1H\}$ NMR (101 MHz, chloroform-*d*, only major diastereomer): δ 220.8, 166.7, 166.5, 151.4 (2C), 151.3, 151.2, 151.1, 151.0, 150.9, 148.8, 148.5, 140.4, 140.2, 138.3, 137.7, 134.5, 134.4, 134.0, 132.13, 132.06, 131.7, 129.6 (2C), 129.3 (2C), 129.24, 129.21, 128.0, 127.9 (2C), 127.8, 127.5, 127.4, 126.8, 126.6, 126.0, 121.9, 121.3, 119.0, 118.9, 114.7, 114.6, 114.29, 114.27, 114.12, 114.05, 113.8, 113.7, 56.3, 56.2, 56.1, 56.03, 56.00, 55.98, 55.2 (2C), 50.6, 48.1, 44.3, 38.2, 36.0, 34.1, 33.8, 31.7, 29.8 (2C), 29.6, 29.2, 26.5, 26.0, 21.7, 14.0 ppm. IR (ATR): ν = 1734 (C=O, esters) cm^{-1}. HRMS (ESI+) *m/z*: calcd. for $C_{73}H_{72}O_{13}Na$ $[M + Na]^+$: 1179.4865, found: 1179.4876.

Follow-up transformations

Di(naphthalen-2-yl) (*pS*)-3²,3⁵,5²,5⁵,7²,7⁵,9²,9⁵-octamethoxy-1,3,5,7,9(1,4)-pentabenzenacyclodecaphane-1²,1⁵-dicarboxylate (3a)

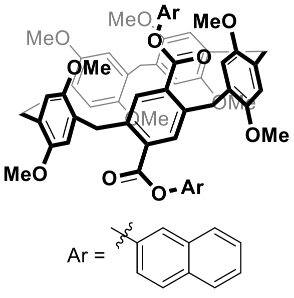

A round-bottom flask (100 ml) was charged with pillar[5]arene **1a** (1000 mg, 1.34 mmol, 1.0 equiv.), *pre*-**C4** (111 mg, 0.27 mmol, 0.2 equiv.), DQ (1205 mg, 2.95 mmol, 2.2 equiv.), AcONa (220 mg, 2.68 mmol, 2.0 equiv.) and naphthalen-2-ol (425 mg, 2.95 mmol, 2.2 equiv.) followed by DCM (27 ml) addition. The reaction mixture was heated to 40 °C (oil bath) and left to stir at this temperature for 18 hours. Once the staring pillar[5]arene or the corresponding monoester was no longer detected by TLC, the heterogeneous mixture was filtered through a short pad of Celite and washed with DCM (2 × 20 ml). The filtrate was partly evaporated on a rotavap and directly loaded onto a column. The product was purified by column chromatography (elution with hexane/EtOAc – 5:1), affording product **3a** (1300 mg, 96%, *er* = 92:8) as a white foam.
All analytical data agree with data on this compound prepared on a 0.1-mmol scale.

Bis(6-formylnaphthalen-2-yl) (*pS*)-3²,3⁵,5²,5⁵,7²,7⁵,9²,9⁵-octamethoxy-1,3,5,7,9(1,4)-pentabenzenacyclodecaphane-1²,1⁵-dicarboxylate (6)

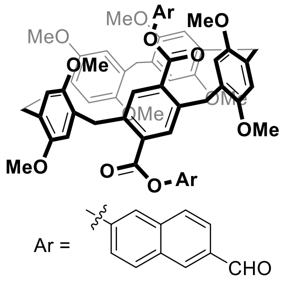

A round-bottom flask (25 ml) was charged with pillar[5]arene **3o** (133 mg, 0.11 mmol, 1.0 equiv., 92:8 *er*) and dissolved in THF (5.0 ml). Diluted hydrochloric acid (1.0M, 2.5 ml) was added in one portion at room temperature. At this temperature, the reaction mixture was left to stir for 1 hour. Once the starting material was no longer detected by TLC, the mixture was poured to a water/EtOAc mixture (20/20 ml). The organic phase was separated, and the water phase was extracted with EtOAc (3 × 10 ml). The organic phases were collected, washed with brine (1 × 10 ml) and dried under anhydrous $MgSO_4$. After filtration of the solid, the filtrate was concentrated under reduced pressure. The crude product was purified by filtration through a short pad of silica gel (eluting by hexane/EtOAc – 2:1-1:1), affording product **6** (117 mg, 95%) as a white foam.
Er = 92:8 (*ee* = 85%), the enantiomeric excess of product **6** was determined by HPLC on a Chiralpak® IA column (*n*-heptane/*i*-PrOH – 50:50, flow rate = 1.0 ml/min, λ = 190 nm, t = 25 °C): t_R = 12.6 min (major), t_R = 35.3 min (minor). $[\alpha]_D^{20}$ = -11.4 (c = 0.7, $CHCl_3$). ^1H NMR (400 MHz, chloroform-*d*): δ 10.20 (s, 2H), 8.41 (d, J = 1.5 Hz, 2H), 8.11 (d, J = 8.9 Hz, 2H), 8.09 – 7.98 (m, 6H), 7.56 (d, J = 2.3 Hz, 2H), 7.34 (dd, J = 8.8, 2.3 Hz, 2H), 6.74 (s, 2H), 6.70 (s, 2H), 6.64 (s, 2H), 6.28 (s, 2H), 4.65 (d, J = 13.7 Hz, 2H), 4.00 (t, J = 14.2 Hz, 4H), 3.90 (s, 2H), 3.69 (s, 8H), 3.48 (s, 6H), 3.45 (s, 6H), 3.10 (s, 6H) ppm. $^{13}C\{^1H\}$ NMR (101 MHz, chloroform-*d*):

δ 192.1 (2C), 166.2 (2C), 151.5 (2C), 151.3 (2C), 151.1 (2C), 151.0 (2C), 150.9 (2C), 140.5 (2C), 137.3 (2C), 134.5 (2C), 134.3 (2C), 134.2 (2C), 132.0 (2C), 131.3 (2C), 130.9 (2C), 129.7 (2C), 129.5 (2C), 129.0 (2C), 127.7 (2C), 127.2 (2C), 124.0 (2C), 122.8 (2C), 119.5 (2C), 115.0 (2C), 114.2 (2C), 114.1 (2C), 113.7 (2C), 56.5 (2C), 56.0 (2C), 55.9 (2C), 55.1 (2C), 34.5 (2C), 29.9 (2C), 28.8 (1C) ppm. IR (ATR): ν = 1738 (C=O, esters), 1691 (C=O, aldehydes) cm^{-1}. HRMS (ESI+) *m*/*z*: calcd. for $C_{67}H_{58}O_{14}Na$ $[M + Na]^+$: 1109.3719, found: 1109.3720.

Bis(6-((*E*)-3-ethoxy-3-oxoprop-1-en-1-yl)naphthalen-2-yl) (*pS*)-$3^2,3^5,5^2,5^5,7^2,7^5,9^2,9^5$-octamethoxy-1,3,5,7,9(1,4)-pentabenzenacyclodecaphane-$1^2,1^5$-dicarboxylate (7)

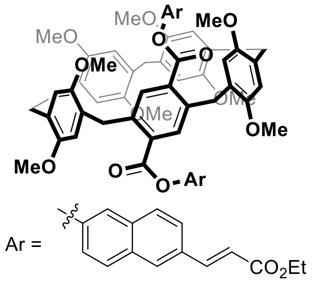

A vial (4 ml) was charged with pillar[5]arene **6** (27.2 mg, 0.025 mmol, 1.0 equiv., 92:8 *er*) and ethyl 2-(triphenyl-λ^5-phosphaneylidene)acetate (43.5 mg, 0.125 mmol, 5.0 equiv.), followed by DCM (1.0 ml) addition at room temperature. The mixture was heated to 40 °C (heating block) and stirred at this temperature overnight (12 hours). Once the aldehyde was no longer detected by TLC, the reaction mixture was cooled to room temperature, and the solvent was evaporated. The crude product was purified by column chromatography (eluting with hexane/EtOAc – 2:1), affording product **7** (24.3 mg, 79%) as a white foam. The ratio of *E*/*Z* isomers of **7** = 18:1 was determined by ^{1}H NMR of the crude reaction mixture.

Er = 91:9 (*ee* = 82%), the enantiomeric excess of product **7** was determined by HPLC on a Chiralpak® IA column (*n*-heptane/*i*-PrOH – 50:50, flow rate = 1.0 ml/min, λ = 190 nm, t = 25 °C): t_R = 20.0 min (major), t_R = 24.4 min (minor). $[\alpha]_D^{20}$ = -21.5 (c = 0.5, $CHCl_3$). ^{1}H NMR (400 MHz, chloroform-*d*, only *E* isomer): δ 8.09 (s, 2H), 8.03 – 8.00 (m, 2H), 7.97 (d, J = 8.9 Hz, 2H), 7.95 – 7.85 (m, 4H), 7.77 (dd, J = 8.7, 1.7 Hz, 2H), 7.50 (d, J = 2.3 Hz, 2H), 7.30 – 7.25 (m, 2H), 6.76 (s, 2H), 6.74 (s, 2H), 6.67 (s, 2H), 6.61 (d, J = 16.0 Hz, 2H), 6.31 (s, 2H), 4.67 (d, J = 13.7 Hz, 2H), 4.34 (q, J = 7.1 Hz, 4H), 4.02 (dd, J = 14.1, 12.2 Hz, 4H), 3.91 (s, 2H), 3.74 (d, J = 14.5 Hz, 2H), 3.71 (s, 6H), 3.51 (s, 6H), 3.48 (s, 6H), 3.13 (s, 6H), 1.40 (t, J = 7.1 Hz, 6H) ppm. $^{13}C\{^1H\}$ NMR (101 MHz, chloroform-*d*, only *E* isomer): δ 167.1 (2C), 166.4 (2C), 151.5 (2C), 151.3 (2C), 151.0 (2C), 150.9 (2C), 149.6 (2C), 144.4 (2C), 140.4 (2C), 134.8 (2C), 134.5 (2C), 132.2 (2C), 132.1 (2C), 131.6 (2C), 130.2 (2C), 129.7 (2C), 129.6 (2C), 129.4 (2C), 128.6 (2C), 127.8 (2C), 127.3 (2C), 124.6 (2C), 122.3 (2C), 119.1 (2C), 118.9 (2C), 114.9 (2C), 114.2 (2C), 114.1 (2C), 113.7 (2C), 60.8 (2C), 56.4 (2C), 56.0 (2C), 55.9 (2C), 55.1 (2C), 34.4 (2C), 29.9 (2C), 28.9 (1C), 14.5 (2C) ppm. IR (ATR): ν = 1738, 1704 (C=O, esters) cm^{-1}. HRMS (ESI+) *m*/*z*: calcd. for $C_{75}H_{70}O_{16}Na$ $[M + Na]^+$: 1249.4556, found: 1249.4560.

Bis(6-(methoxycarbonyl)naphthalen-2-yl) (*pS*)-$3^2,3^5,5^2,5^5,7^2,7^5,9^2,9^5$-octamethoxy-1,3,5,7,9(1,4)-pentabenzenacyclodecaphane-$1^2,1^5$-dicarboxylate (3n)

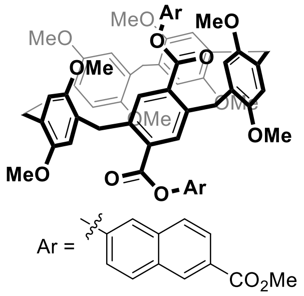

A vial (4 ml) was charged with pillar[5]arene **6** (27.2 mg, 0.025 mmol, 1.0 equiv., 92:8 *er*), *pre*-**C6** (1.8 mg, 0.005 mmol, 0.2 equiv.), DQ (22.5 mg, 0.055mmol, 2.2 equiv.) and Cs_2CO_3 (22.5 mg, 0.05 mmol, 2.0 equiv.), followed by DCM (1.0 ml) and methanol (5.1 µl, 0.125 mmol, 5.0 equiv.) addition at room temperature. The reaction was stirred at room temperature (~20 °C) for 6 hours. Once the aldehyde was no longer detected (TLC), the reaction mixture was directly loaded onto a silica gel column. The product was purified by column chromatography (eluting with hexane/EtOAc- 12:1), affording product **3n** (23.0 mg, 80%, 92:8 *er*) as a white foam.

All analytical data agree with data on this compound (**3n**) *prepared by organocatalytic esterification of diformylpillar[5]arene* **1a**.

Rotaxane (8)

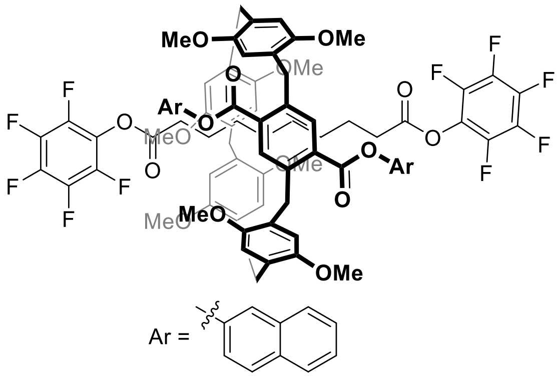

The Schlenk tube (2 ml) was charged with pillar[5]arene **3a** (206 mg, 0.2 mmol, 2.5 equiv., 92:8 *er*) and evacuated/refilled with Ar. Then, the staring material was dissolved in anhydrous chloroform (0.4 ml). This solution was cooled to -15 °C (cryocooler). At this temperature sebacoyl chloride (17.0 µl, 0.08 mmol, 1.0 equiv.) was added in one portion. The reaction mixture was left to stir for 1 hour before adding a solution of pentafluorophenol (32.4 mg, 0.176 mmol, 2.2 equiv.) and TEA (27.9 µl, 0.2 mmol, 2.5 equiv.) in anhydrous chloroform (0.2 ml). Subsequently, the reaction mixture was slowly heated to room temperature and left to stir overnight. Then, the reaction mixture was directly loaded onto a silica gel column and purified by filtration through a short pad of silica gel (eluting by hexane/EtOAc – 7:1-5:1), affording virtually pure product **8** and recovering the starting material **3a** (160.2 mg). Analytically pure product was purified by column chromatography (elution with hexane/DCM/EtOAc – 2:1:0-7:0:1), affording product **8** (30.2 mg, 24%) as pale oil.

Note: The carbon signals of the pentafluorophenyl groups in the $^{13}C\{^{1}H\}$ NMR spectra were not detected due to coupling with fluorine atoms, which reduced the signal intensity. The presence of pentafluorophenyl groups was confirmed by ^{19}F NMR and HMRS.

Er = 92:8 (*ee* = 85%), the enantiomeric excess of product **8** was determined by HPLC on a Chiralpak® IA column (*n*-heptane/*i*-PrOH – 80:20, flow rate = 1.0 ml/min, λ = 190 nm, t = 25 °C): t_R = 4.9 min (major), t_R = 6.2 min (minor). $[\alpha]_D^{20}$ = -60.8 (c = 1.1, $CHCl_3$). ^{1}H NMR (400 MHz, chloroform-*d*): δ 8.62 (s, 2H), 7.86 (dd, J = 9.4, 6.8 Hz, 4H), 7.76 (d, J = 2.3 Hz, 2H), 7.75 – 7.71 (m, 2H), 7.52 – 7.46 (m, 4H), 7.43 (dd, J = 8.8, 2.3 Hz, 2H), 7.17 (s, 2H), 6.96 (s, 2H), 6.94 (s, 2H), 6.92 (s, 2H), 4.70 (d, J = 12.7 Hz, 2H), 4.26 (d, J = 12.7 Hz, 2H), 3.86 (s, 6H), 3.81 (s, 4H), 3.79 (s, 8H), 3.76 (s, 6H), 3.75 (s, 6H), 2.06 – 1.96 (m, 4H), 0.53 (p, J = 7.9 Hz, 4H), -0.82 (d, J = 13.3 Hz, 4H), -1.11 (h, J = 8.0 Hz, 4H) ppm. $^{13}C\{^{1}H\}$ NMR (101 MHz, chloroform-*d*): δ 169.7 (2C), 165.8 (2C), 150.8 (2C), 150.7 (2C), 150.53 (2C), 150.49 (2C), 148.6 (2C), 142.3 (2C), 135.5 (2C), 133.8 (2C), 131.5 (2C), 131.2 (2C), 129.48 (2C), 129.46 (2C), 128.7 (2C), 128.4 (2C), 127.9 (2C), 127.5 (2C), 127.4 (2C), 126.7 (2C), 126.0 (2C), 121.1 (2C), 118.6 (2C), 113.7 (2C), 113.5 (2C), 113.4 (2C), 55.83 (2C), 55.76 (2C), 55.61 (2C), 55.58 (2C), 33.1 (2C), 31.7 (2C), 29.2 (2C), 29.0 (1C), 28.3 (2C), 27.8 (2C), 24.6 (2C) ppm. ^{19}F NMR (376 MHz, chloroform-*d*): δ -152.89 – -153.78 (m, 4F), -158.27 (t, J = 21.7 Hz, 2F), -162.43 (td, J = 22.3, 5.1 Hz, 4F) ppm. IR (ATR): ν = 1789, 1732 (C=O, esters) cm^{-1}. HRMS (ESI+) *m*/*z*: calcd. for $C_{87}H_{74}O_{16}F_{10}Na$ $[M + Na]^{+}$: 1587.4709, found: 1587.4712.

Proposed reaction mechanism

Briefly (Supplementary Figure 2), a chiral carbene is formed by base deprotonation of the corresponding azolium salt (*pre*-**C4**). Then, the nucleophilic carbene attacks (step A) the aldehyde carbon, yielding a tetrahedral intermediate **II**. This intermediate undergoes a 1,2-proton shift (step B), generating a Breslow intermediate (**III**). In the presence of an oxidant (DQ), the Breslow intermediate is irreversibly oxidized (step C) to an acyl azolium intermediate (**IV**). The resulting acyl azolium is electrophilic at the carbonyl carbon and, thus, undergoes acyl substitution (step D) with alcohol (or alkoxide). The final acyl substitution regenerates the carbene back to the catalytic cycle, yielding ester **IV**. Because this intermediate is prochiral, we postulated that the second catalytic cycle of organocatalytic oxidative esterification is enantio-divergent. In general, the proposed reaction mechanism is the same as the first cycle, but chirality derives from steric hindrance of the chiral NHC carbene.

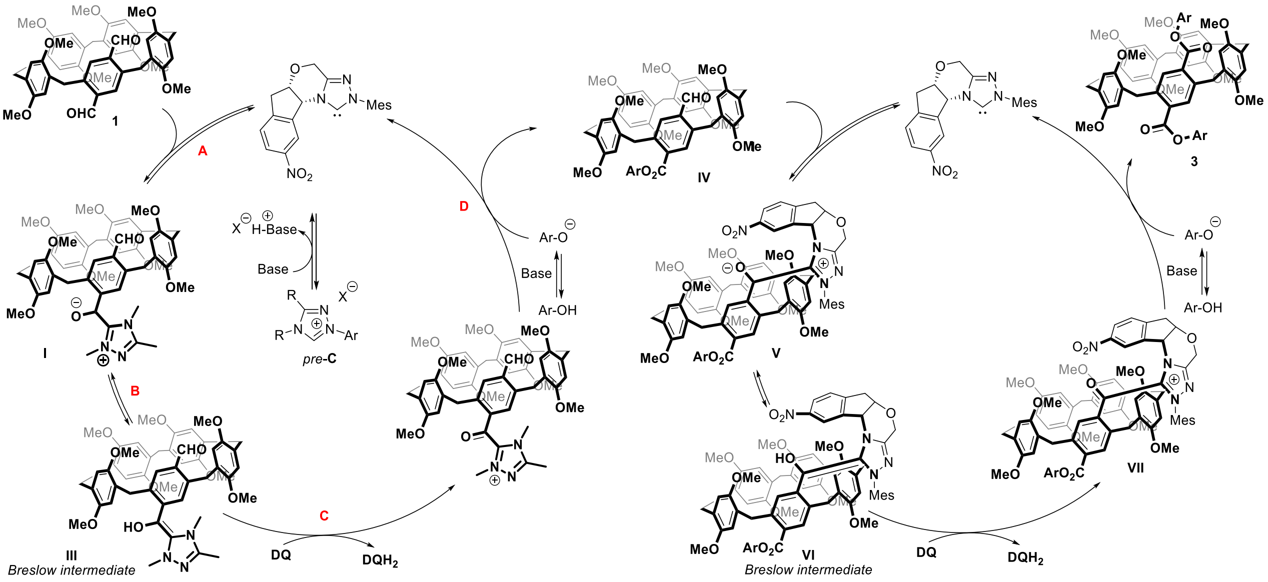

Supplementary Figure 2. Proposed reaction mechanism

Configuration stability

Variation of enantiomeric purity as a function of temperature

Compound **3a** (2.5 mg) was dissolved in mesitylene (2.5 ml). This solution was left to stir at 40 °C (heating block) for 1 hour. Then, a sample was collected from the heated solution (0.1 ml) to determine the enantioselectivity (0.1 ml). Then, the temperature was increased by 10 °C, and the same procedure was repeated up to 120 °C.

Supplementary Table 9. Enantiomeric purity of **3a** at various temperatures

Entry	Temperature (°C)	Time (h)	Major enan. (%)[a]	Minor enan. (%)[a]
1	20 (r.t.)	0	97.3	2.07
2	40	1	97.33	2.65
3	50	2	97.55	2.45
4	60	3	96.72	3.28
5	70	4	95.28	4.72
6	80	5	92.19	7.81
7	90	6	84.85	15.15
8	100	7	70.69	29.31
9	110	8	57.06	42.94
10	120	9	51.02	48.98

[a] Determined by chiral HPLC.

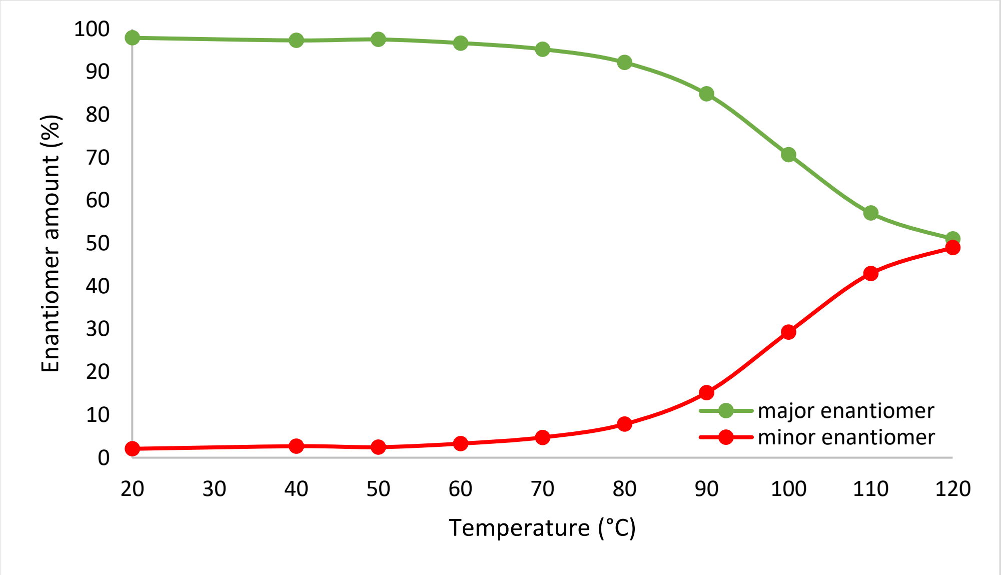

Supplementary Figure 2. Variation of enantiomer amount as a function of temperature

Experimental determination of the rotational barrier

Compound 3a (2.8 mg) was dissolved in pre-heated (90 °C) mesitylene (2.8 ml). This solution was left to stir at 90°C (heating block) for 30 minutes. Then, a sample was collected from the heated solution (0.1 ml) to determine the enantioselectivity (0.1 ml). The same procedure was repeated in 30-minute intervals for 5 hours.

Supplementary Table 10. Enantiomeric purity of **3a** in various temperatures

Entry	Time (s)	Major enan. (M, %)[a]	Minor enan. (m, %)[a]	ln(M+m)/(M-m)
1	0	98.04	1.96	0.039989
2	1800	94.49	5.51	0.116789
3	3600	89.52	10.48	0.235216
4	5400	85.75	14.25	0.335473
5	7200	82.95	17.05	0.417032
6	9000	79.14	20.86	0.539911
7	10800	77.31	22.68	0.604487
8	12600	74.50	25.50	0.713350
9	14400	72.10	27.90	0.816445
10	16200	68.31	31.69	1.004576
11	18000	66.63	33.37	1.100815

[a] Determined by chiral HPLC.

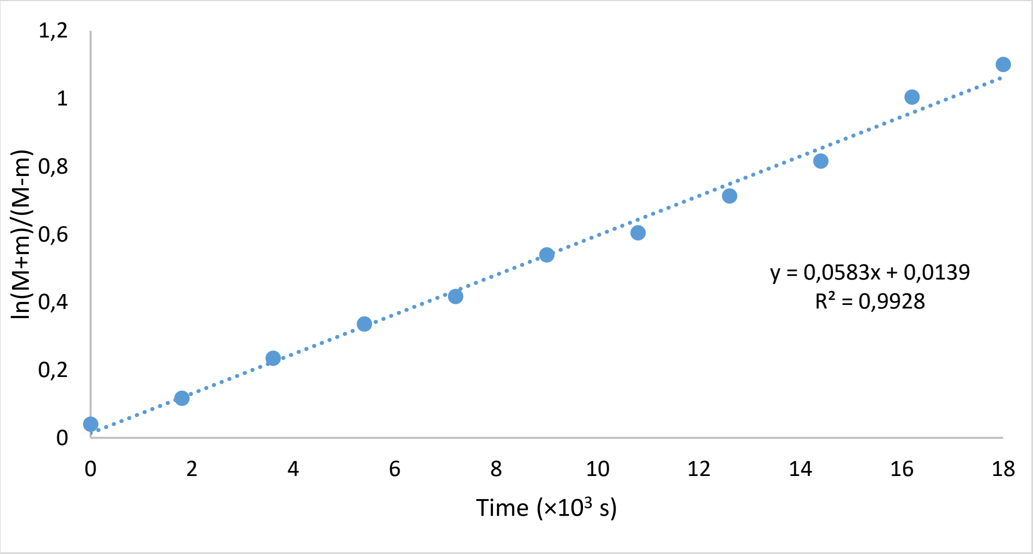

Supplementary Figure 3. Variation of ln(M+m)/(M-m) as a function of time

Following the previously reported rotational barrier calculation,[20] k_B = 1.381×10^{-23} J/K - Boltzmann's constant, h = 6.626 × 10^{-34} J·s - Planck's constant, and R = 8.3145 J/mol·K - universal gas constant.

$$\ln\frac{M+m}{M-m} = k_{rac}t + c = k_{rot}t + c \quad (1)$$

$$k_{rot} = \frac{slope}{2} \quad (2)$$

$$k_{rac} = 2k_{rot} = \frac{2k_{\mathrm{B}}T}{h}e^{(-\frac{\Delta G^{\ddagger}_{\mathrm{rot}}}{RT})} c = k_{rot}t + c \quad (3)$$

$$K^{\ddagger}_{\mathrm{rot}} = \frac{k_{\mathrm{rot}}h}{k_{\mathrm{B}}T} \quad (4)$$

$$\Delta G^{\ddagger}_{\mathrm{rot}} = -RTlnK^{\ddagger}_{\mathrm{rot}} \quad (5)$$

Calcutions for **3a** at T = 363.15 K.

$$k_{rot} = \frac{5.8294 \times 10^{-5} s^{-1}}{2} = 2.915 \times 10^{-5} s^{-1}$$

$$K_{\mathrm{rot}}^{\ddagger} = \frac{2.915 \times 10^{-5} s^{-1} \times 6.626 \times 10^{-34} \mathrm{J \cdot s}}{1.381 \times 10^{-23} \mathrm{J \cdot s^{-1}} \times 363.15\ \mathrm{K}} = \ 3.85110^{-18}$$

$$\Delta G_{\mathrm{rot}}^{\ddagger} = -8.314\ \mathrm{J \cdot s^{-1}\ \cdot K^{-1}} \times 363.15\ \mathrm{K}\ \times \ln(3.85110^{-18}) = 121065.7\ \mathrm{J \cdot mol^{-1}}$$

$$121065.7\ \mathrm{J \cdot mol^{-1}} = 28.94\ \mathrm{kcal \cdot mol^{-1}}$$

Crystallographic data

X-ray single-crystal data of *rac*-**3a, 3a** and **3q** were collected on a Bruker D8 VENTURE Kappa Duo PHOTONIII by IμS micro-focus sealed tube. The samples were kept at low temperature during the measurements with a Cryostream Cooler 1000. The structures were solved using direct methods XT[21] and refined by full matrix least squares based on F^2 SHELXL2019.[22] The hydrogen atoms on carbon were fixed into idealized positions (riding model) and assigned temperature factors either $H_{iso}(H) = 1.2\ U_{eq}$(pivot atom) or $H_{iso}(H) = 1.5\ U_{eq}$ (pivot atom) for a methyl moiety. The absolute structure[23] determinations were based on anomalous dispersion of oxygen and Cl atoms.

All samples contained chiral molecules, and *rac*-**3a** crystals were racemic from the centrosymmetric space group *P*-1. The samples of **3a** were formed by one enantiomer (space group $P2_1$). Regardless of naphthalene disorder and methoxy moieties, the absolute structure of measured crystal was determined reliably.

The presence of a chlorine atom with high anomalous dispersion in **3q** and the Sohncke space group $P2_12_12_1$ of the crystal were promising factors for determining the absolute structure of this molecule. However, the refined Flack parameter suggested racemic twinning, and the ratio of twin domains was 0.673:0.327 for one enantiomer and its centrosymmetric counterpart.

Supplementary Table 11. Crystal data, data collection, and refinement parameters for *rac*-**3a**, **3a**, and **3q**

**Compound**	***Rac*-3a**	**3a**	**3q**
CCDC	2498606	2498607	2498608
Formula	$C_{65}H_{58}O_{12}$	$C_{65}H_{58}O_{12}$	$C_{65}H_{56}Cl_2O_{12}$
M.w.	1031.11	1031.11	1099.99
Crystal system	Triclinic	Monoclinic	Orthorhombic
Space group	*P* -1 (No.2)	$P2_1$ (4)	$P2_1\,2_1\,2_1$ (No.19)
a [Å]	10.4977 (4)	8.0867 (3)	7.6850 (3)
b [Å]	12.2202 (4)	21.9071 (8)	15.0492 (6)
c [Å]	20.7690 (8)	14.7521 (5)	45.8912 (19)
α [°]	94.242 (1)		
β [°]	100.724 (1)	92.577 (1)	
γ [°]	93.576 (1)		
Z	2	2	4
$V[Å^3]$	2602.73 (17)	2610.78 (16)	5307.5 (4)
Temperature	100	100	100
D_x [g cm^{-3}]	1.316	1.312	1.377
Wavelength, Å	0.71073	1.54178	1.54178
Crystal size [mm]	0.39 × 0.24 × 0.19	0.24 × 0.17 × 0.06	0.30 × 0.03 × 0.03
Crystal color, shape	Prism, colourless	Prism, colourless	Needle, colourless
μ [mm^{-1}]	0.09	0.73	1.66
T_{min},T_{max}	0.966, 0.983	0.845, 0.958	0.64, 0.955
Measured reflections	59358	87947	57804

Independent diffractions (R_{int}[a])	12771, (0.023)	10915, (0.023)	11153, (0.049)
Observed diffract. [I>2σ(I)]	11956	10826	10747
No. of parameters	702	795	721
R[b]	0.040	0.032	0.037
$wR(F^2)$ for all data	0.102	0.087	0.092
GOF[c]	1.02	1.03	1.07
Residual electron density [e/Å^3]	0.35, -0.23	0.31, -0.24	0.29, −0.31
Absolute structure parameter		-0.05 (2)	0.327 (13)

[a]$R_{int} = \Sigma|F_o^2 - F_{o,mean}^2|/\Sigma F_o^2$; [b]$R(F) = \Sigma||F_o| - |F_c||/\Sigma|F_o|$; $wR(F^2) = [\Sigma(w(F_o^2 - F_c^2)^2)/(\Sigma w(F_o^2)^2)]^{½}$; [c] GOF = $[\Sigma(w(F_o^2 - F_c^2)^2)/(N_{diffrs} - N_{params})]^{½}$

X-ray crystallographic data have been deposited with the Cambridge Crystallographic Data Centre under deposition numbers 2498606, 2498607, 2498608, and can be obtained free of charge from the Centre via its website (https://www.ccdc.cam.ac.uk/structures/).

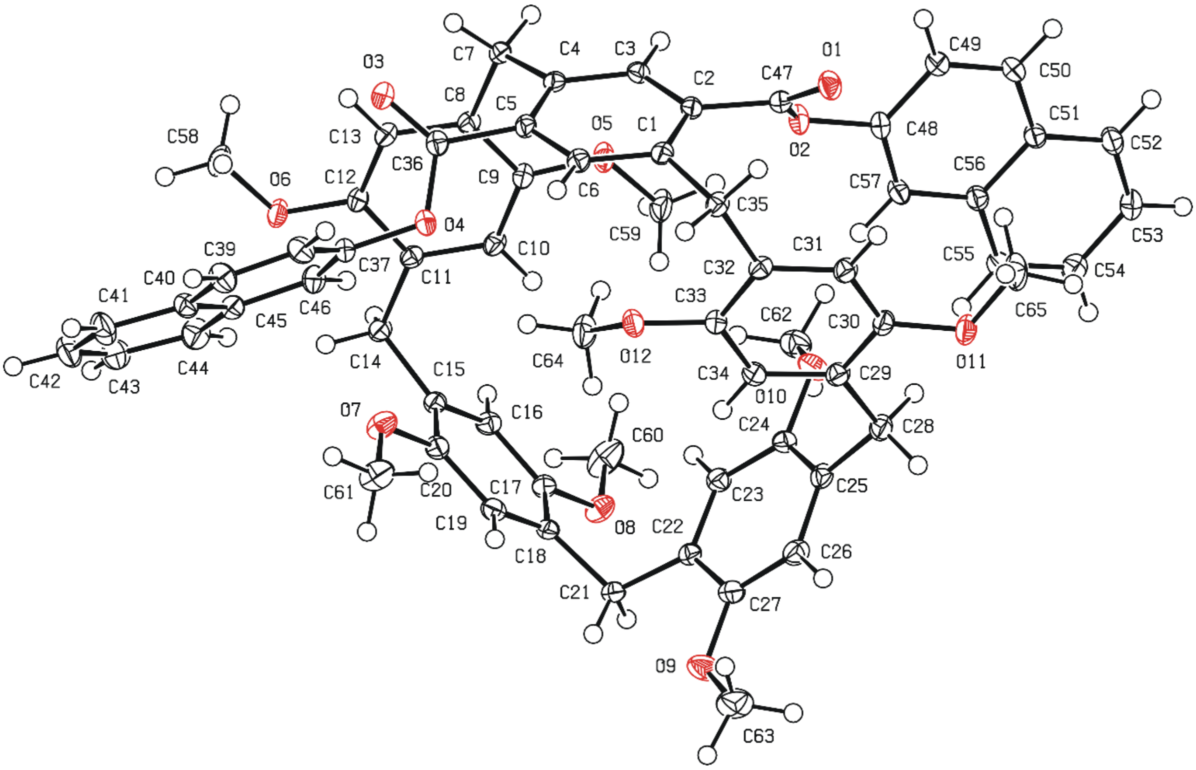

Supplementary Figure 4. View of the *rac*-**3a** molecule with atom numbering schema; the displacement ellipsoids are drawn on a 30% probability level (CCDC: 2498606).

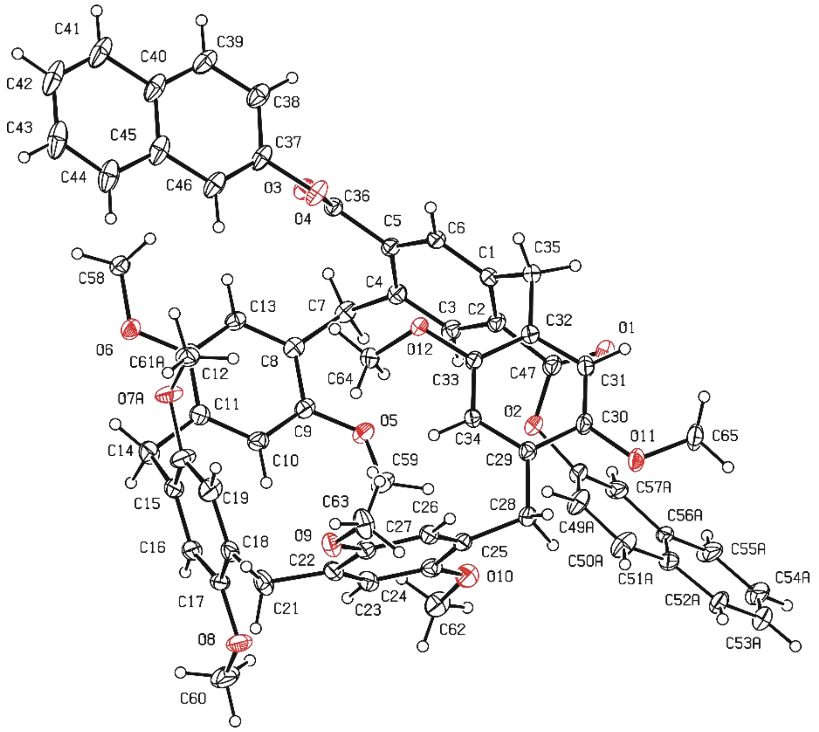

Supplementary Figure 5. View of the **3a** molecule with atom numbering schema; the displacement ellipsoids are drawn on a 30% probability level. The second positions of the disordered moieties are omitted for clarity (CCDC: 2498607).

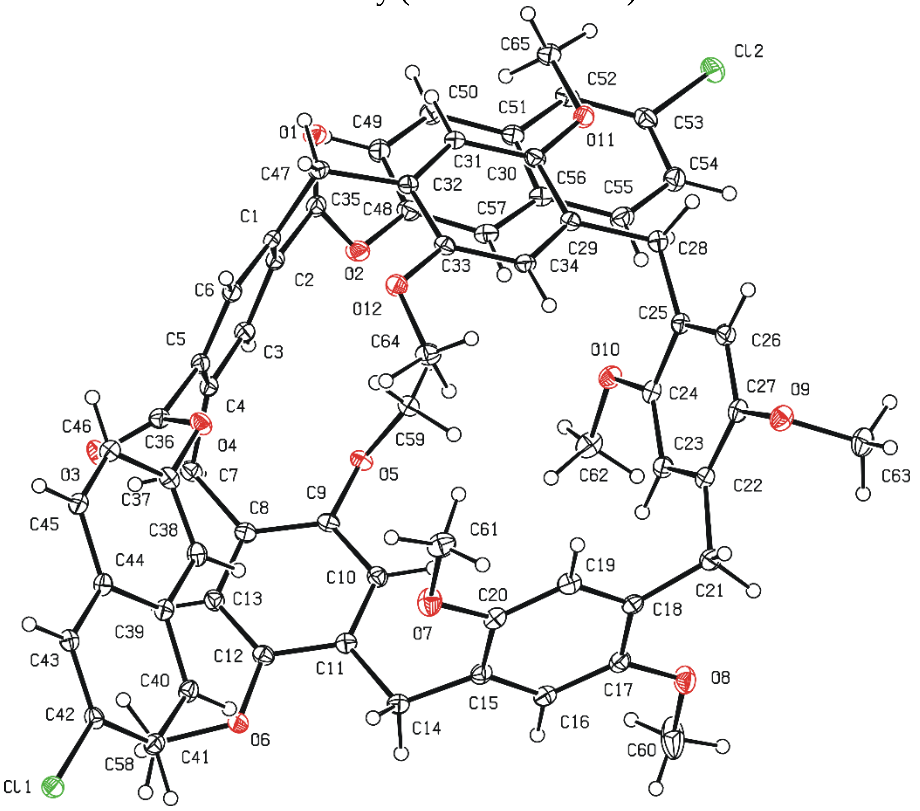

Supplementary Figure 6. View of the **3q** molecule with atom numbering schema; the displacement ellipsoids are drawn on a 30% probability level. The depicted enantiomer is forming 67% of the measured crystal (CCDC: 2498608).

NMR spectra

$3^2,3^5,5^2,5^5,7^2,7^5,9^2,9^5$-Octamethoxy-1,3,5,7,9(1,4)-pentabenzenacyclodecaphane-$1^2,1^5$-dicarbaldehyde (1a)

^{1}H NMR of **1a** (400 MHz, chloroform-*d*)

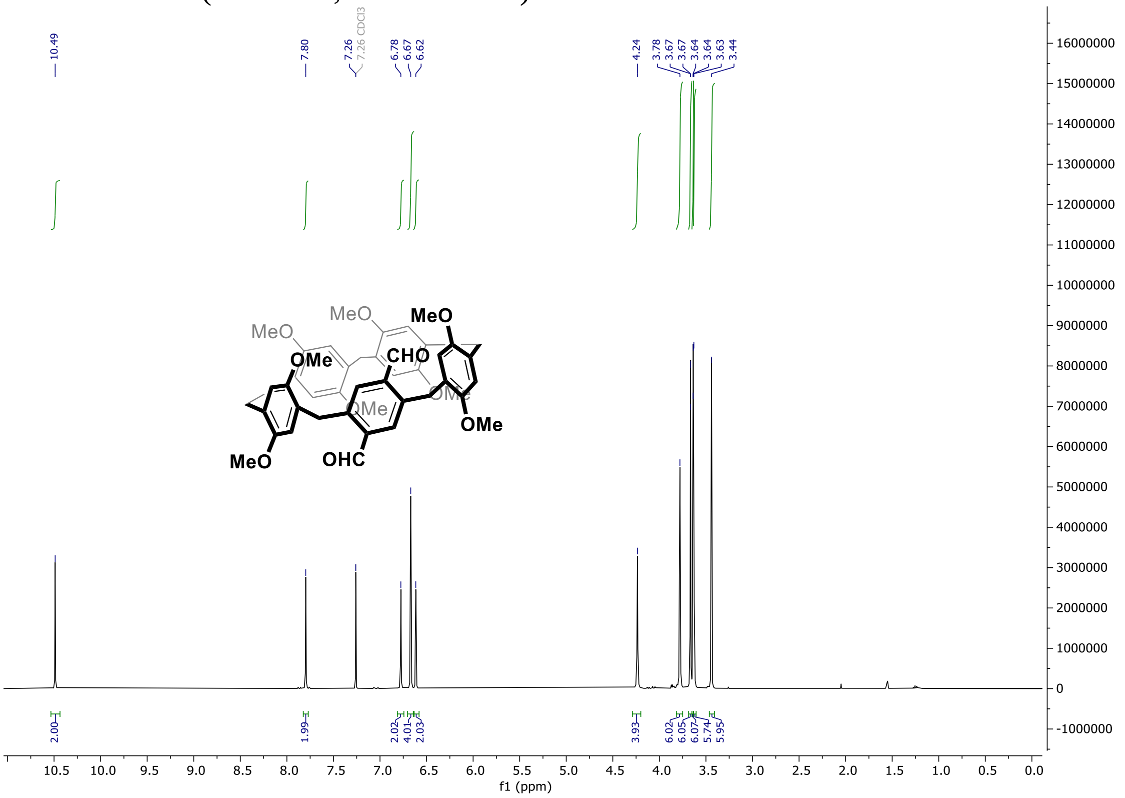

^{13}C{^{1}H} NMR of **1a** (101 MHz, chloroform-*d*)

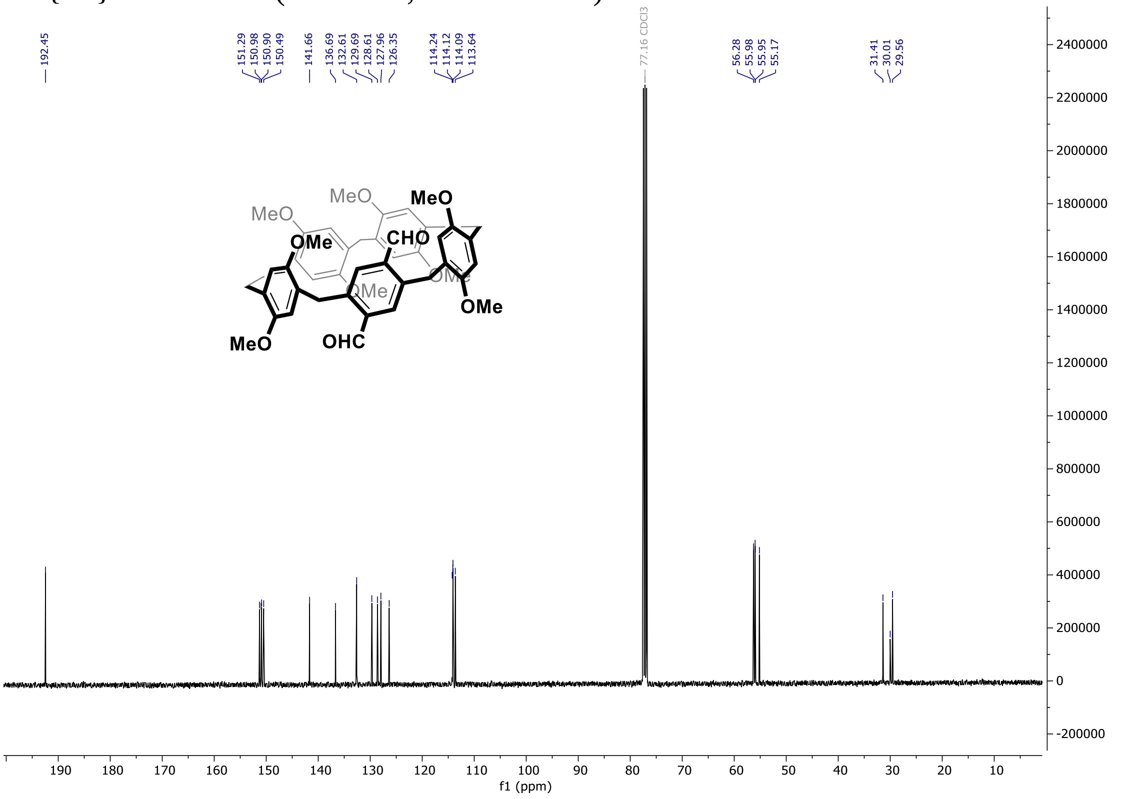

3[2],3[5],5[2],5[5],7[2],7[5],9[2],9[5]-Octaethoxy-1,3,5,7,9(1,4)-pentabenzenacyclodecaphane-1[2],1[5]-dicarbaldehyde (1b)

^{1}H NMR of **1b** (400 MHz, chloroform-*d*)

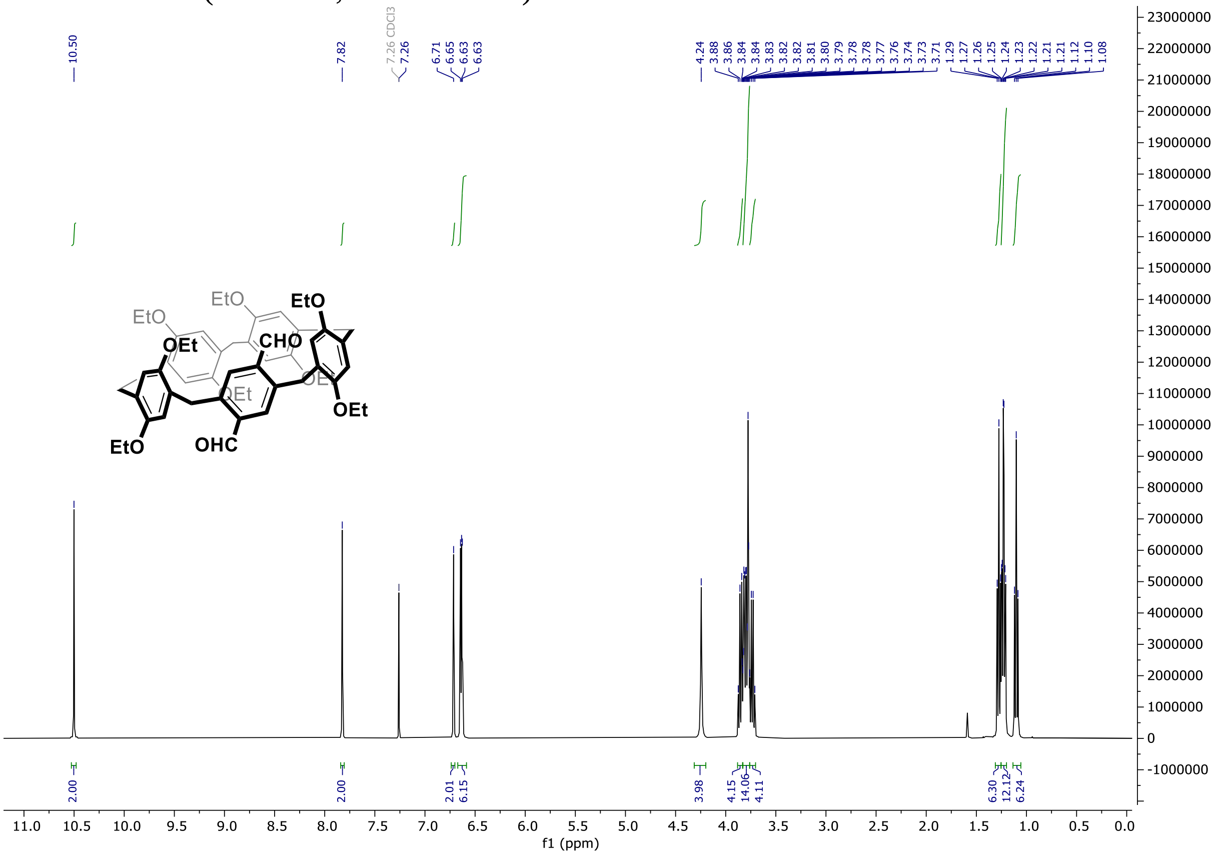

^{13}C{^{1}H} NMR of **1b** (101 MHz, chloroform-*d*)

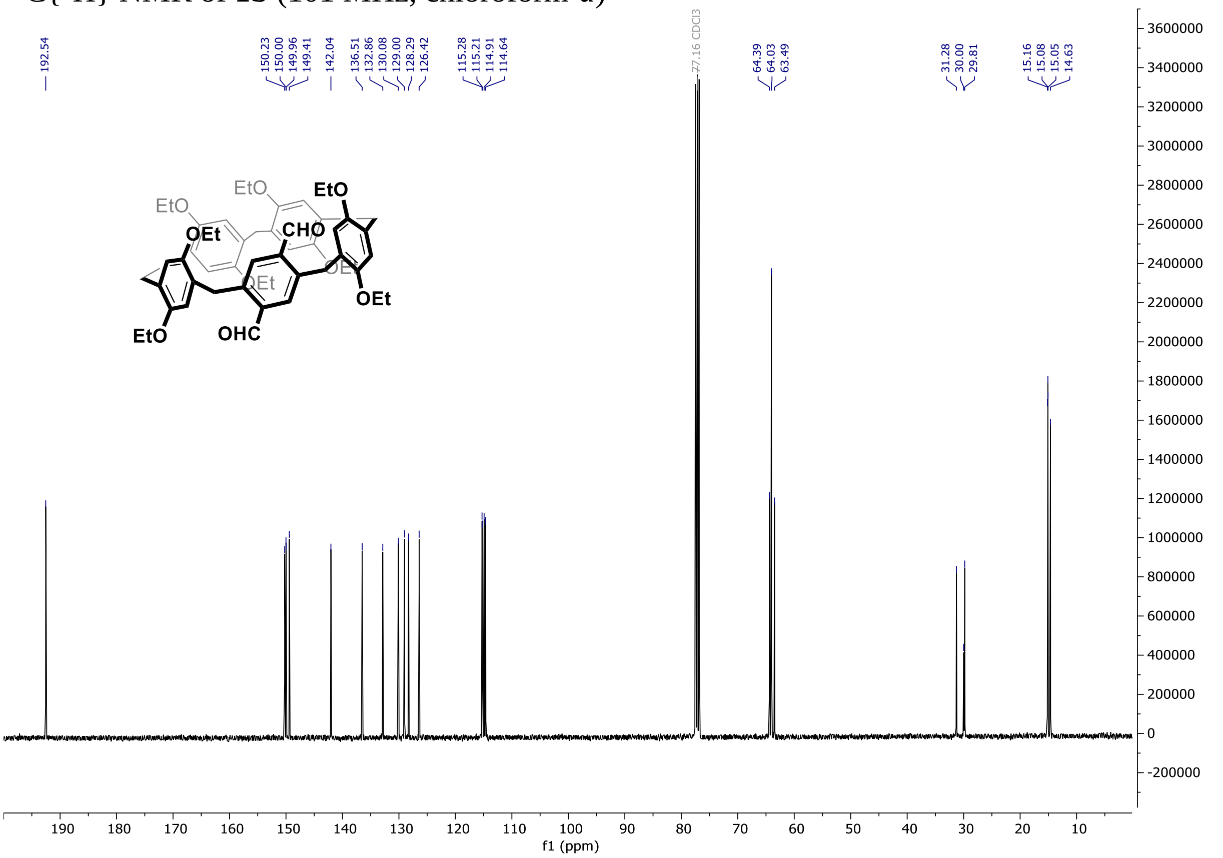

(2*E*,2'*E*)-3,3'-($3^2,3^5,5^2,5^5,7^2,7^5,9^2,9^5$-Octamethoxy-1,3,5,7,9(1,4)-pentabenzenacyclodecaphane-$1^2,1^5$-diyl)diacrylaldehyde (1c)

^{1}H NMR of **1c** (400 MHz, chloroform-*d*)

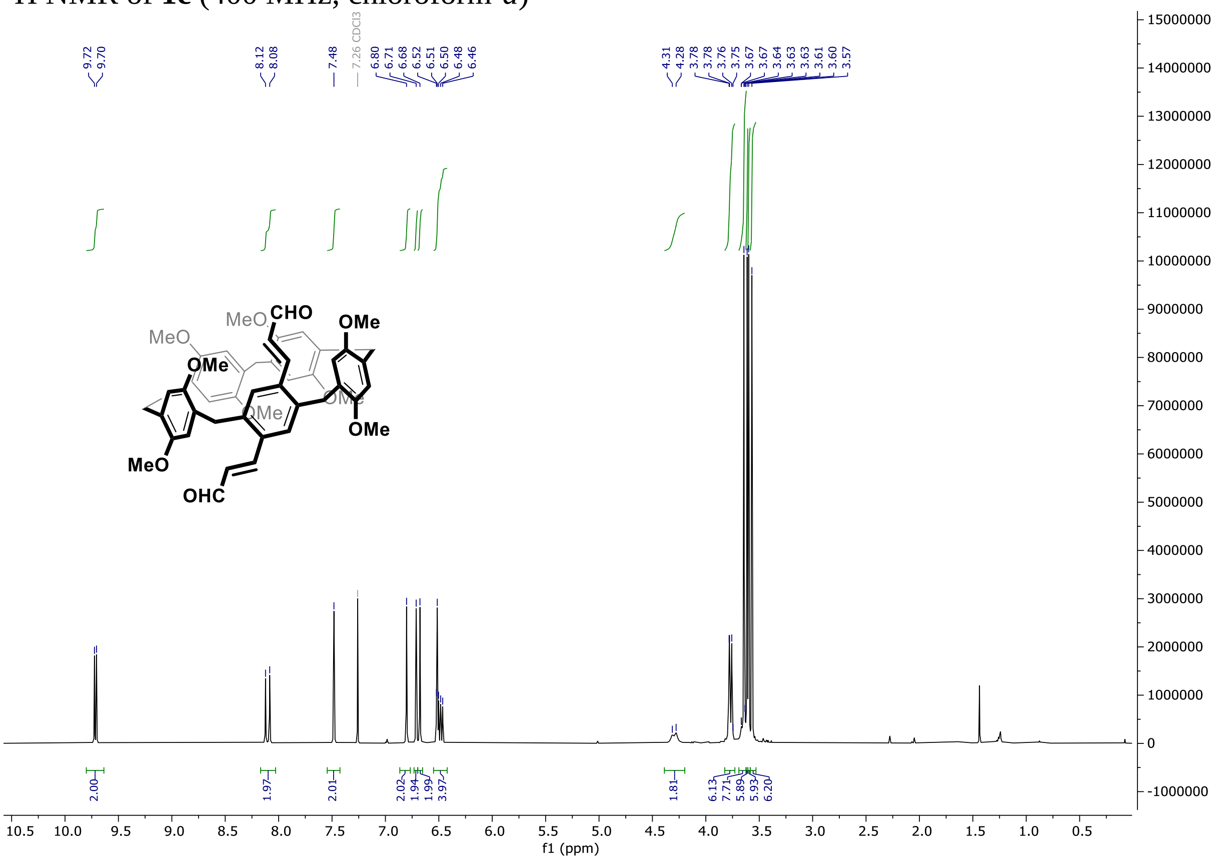

^{13}C{^{1}H} NMR of **1c** (101 MHz, chloroform-*d*)

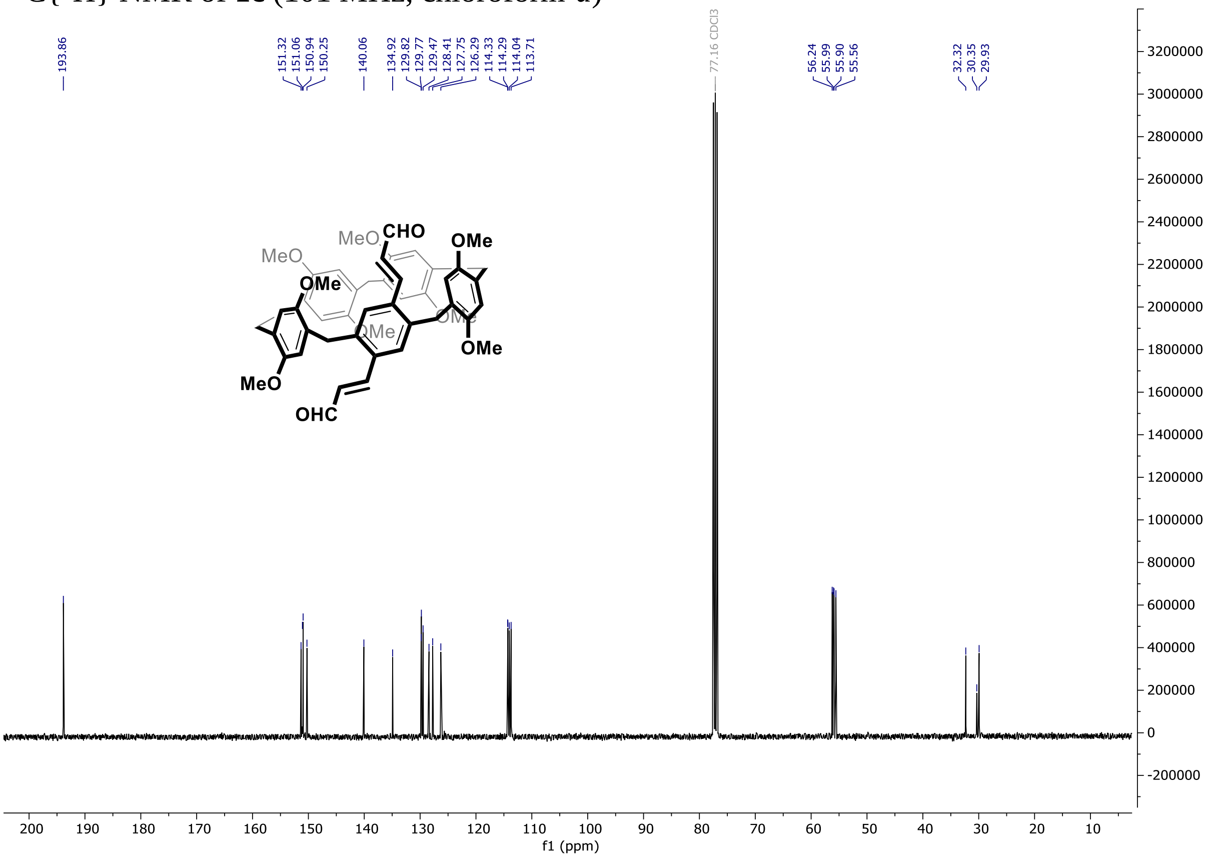

1⁵-([1,1'-Biphenyl]-4-yl)-3²,3⁵,5²,5⁵,7²,7⁵,9²,9⁵-octamethoxy-1,3,5,7,9(1,4)-pentabenzenacyclodecaphane-1²-carbaldehyde (1d)

^{1}H NMR of **1d** (400 MHz, chloroform-*d*)

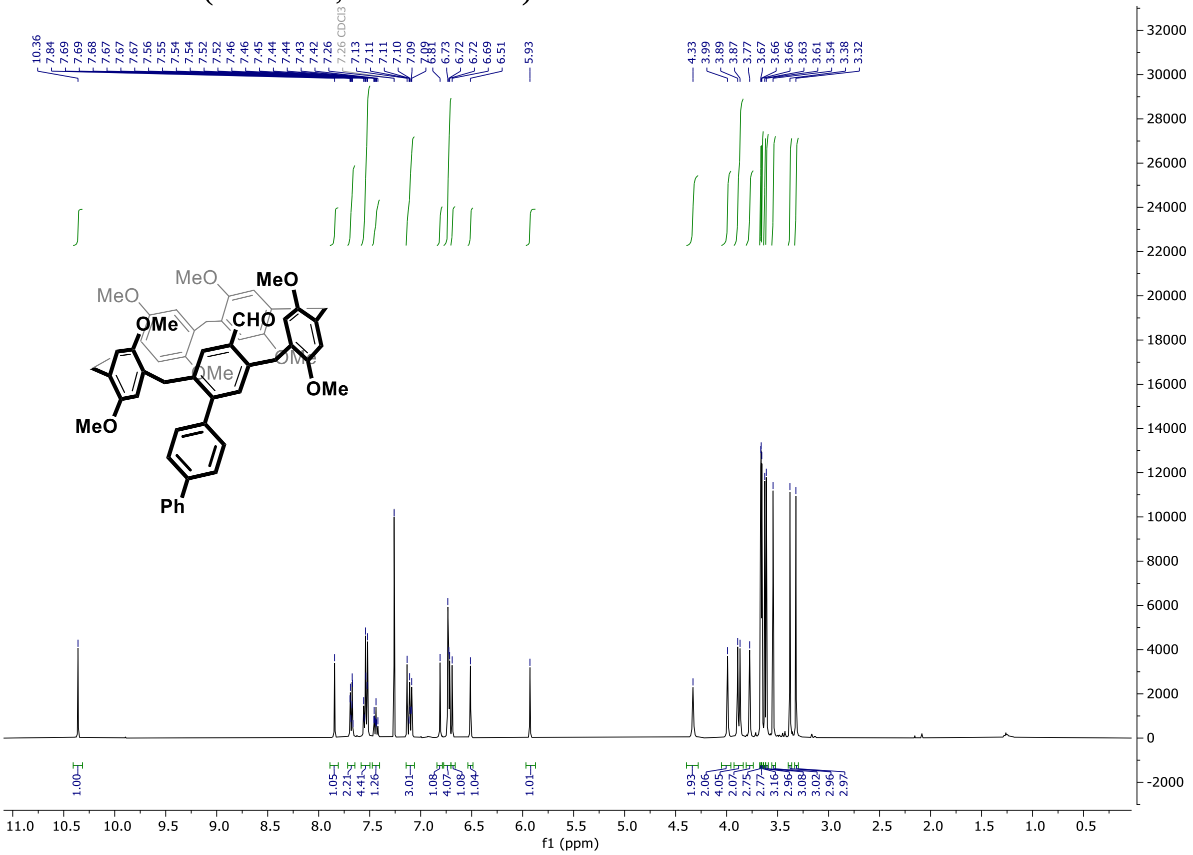

^{13}C{^{1}H} NMR of **1d** (101 MHz, chloroform-*d*)

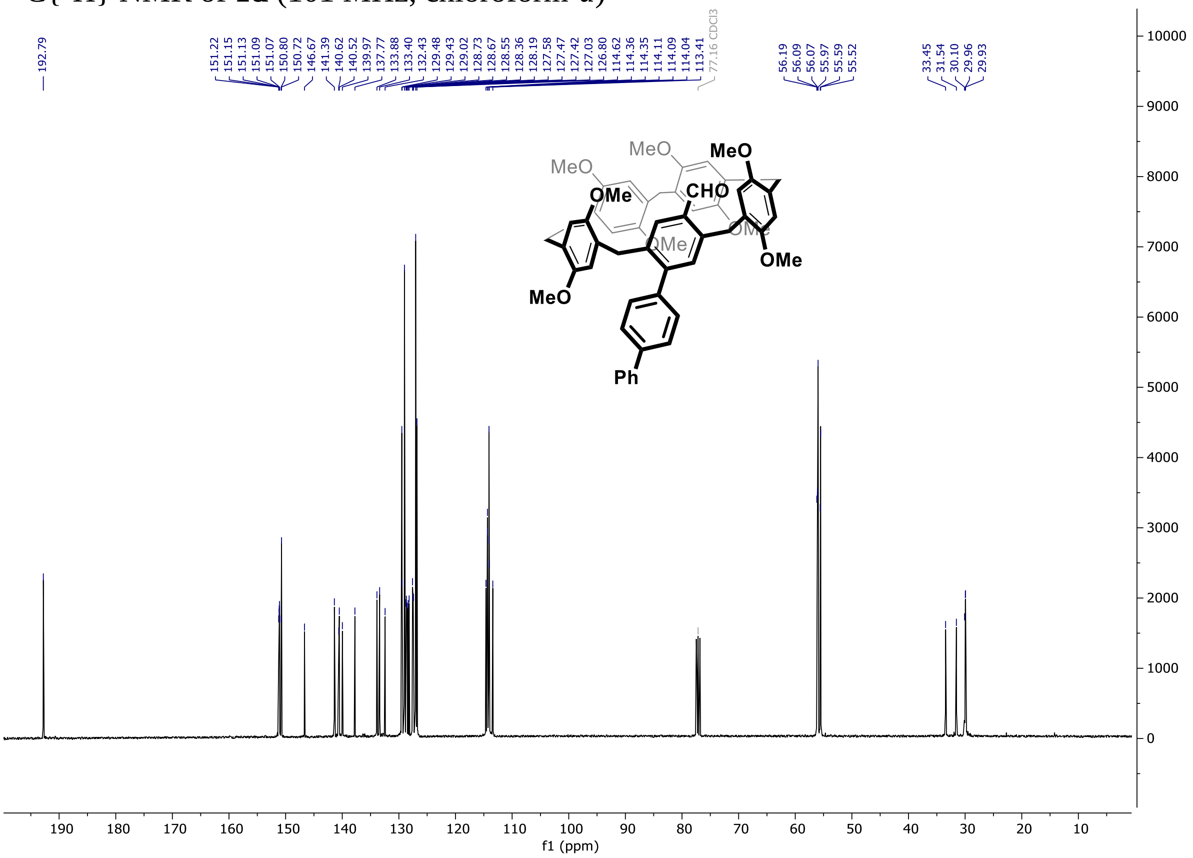

Naphthalen-2-yl 1^5-formyl-3^2,3^5,5^2,5^5,7^2,7^5,9^2,9^5-octamethoxy-1,3,5,7,9(1,4)-pentabenzenacyclodecaphane-1^2-carboxylate (1e)

^{1}H NMR of **1e** (400 MHz, chloroform-*d*)

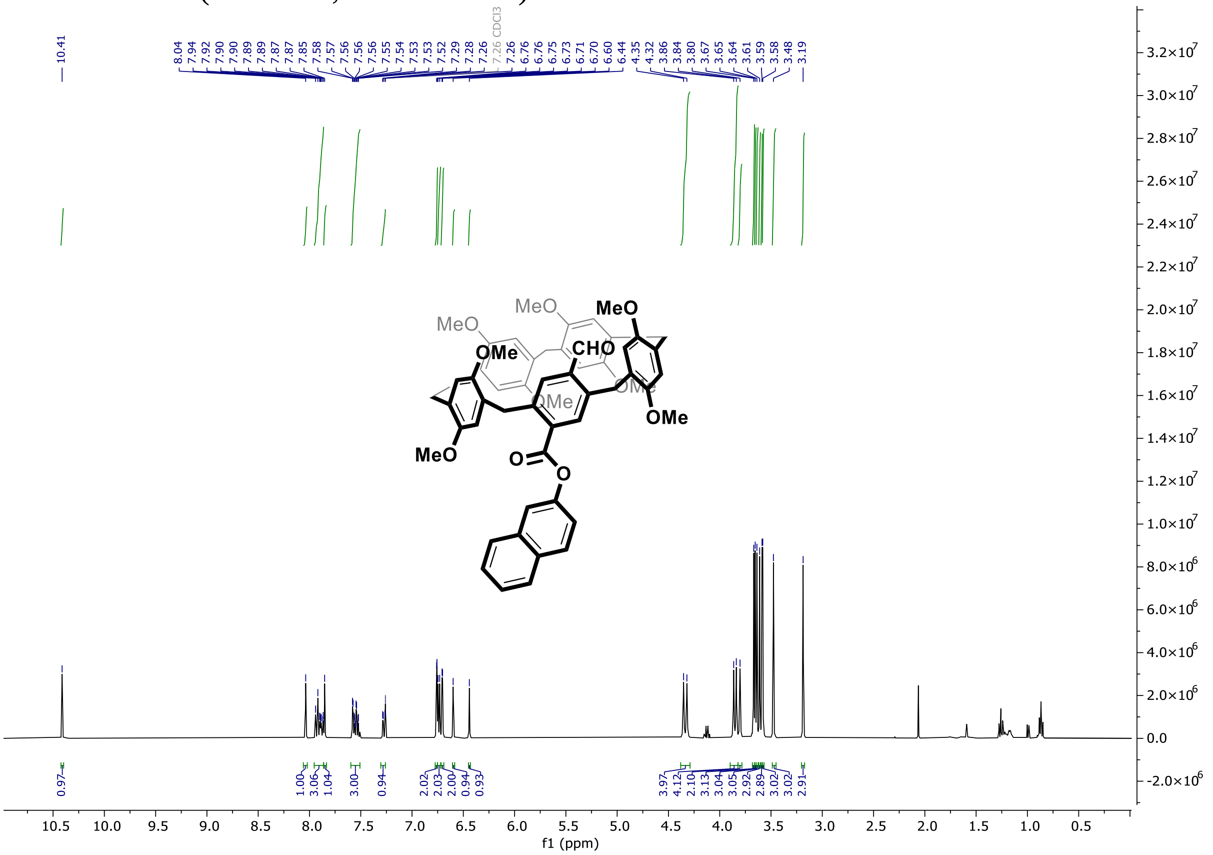

^{13}C{^{1}H} NMR of **1e** (101 MHz, chloroform-*d*)

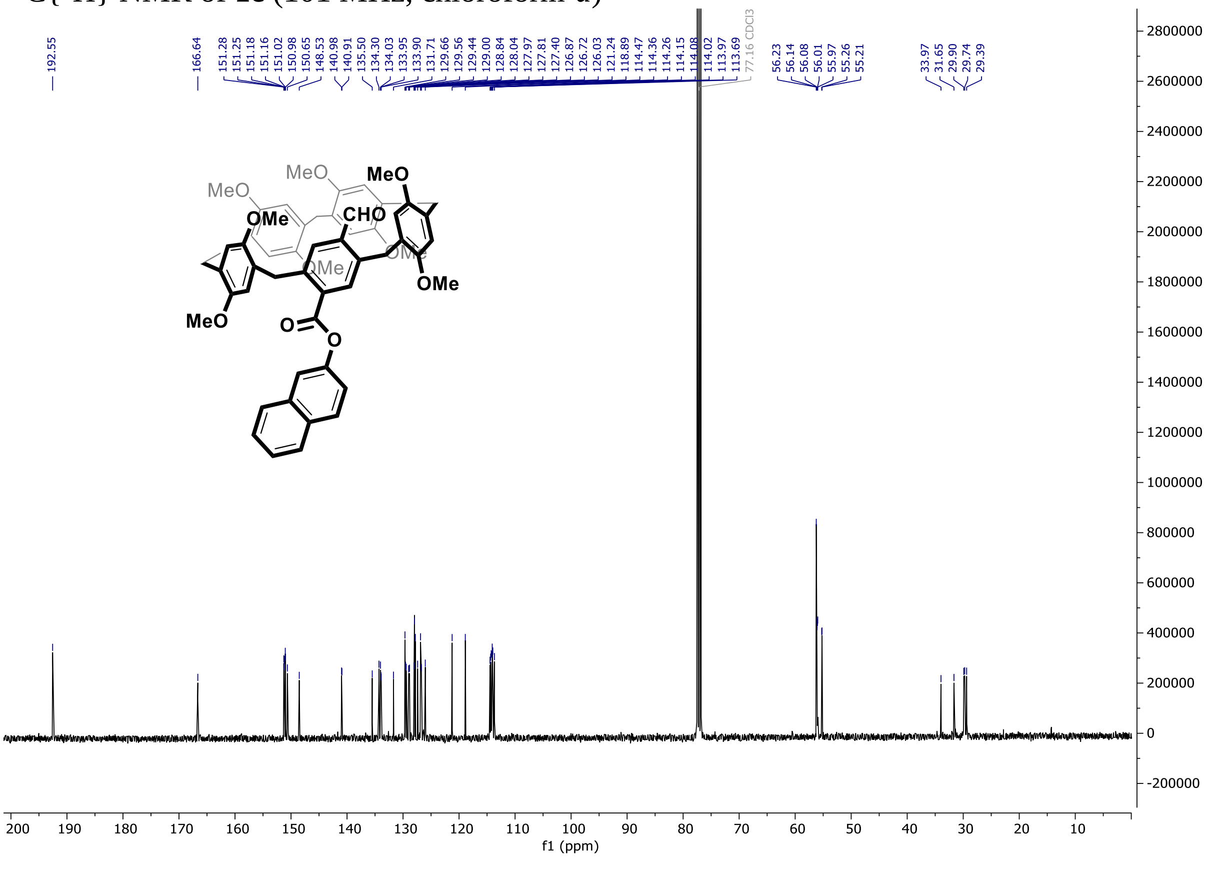

Di(naphthalen-2-yl) (*pS*)-$3^2,3^5,5^2,5^5,7^2,7^5,9^2,9^5$-octamethoxy-1,3,5,7,9(1,4)-pentabenzenacyclodecaphane-$1^2,1^5$-dicarboxylate (3a)

^{1}H NMR of **3a** (400 MHz, chloroform-*d*)

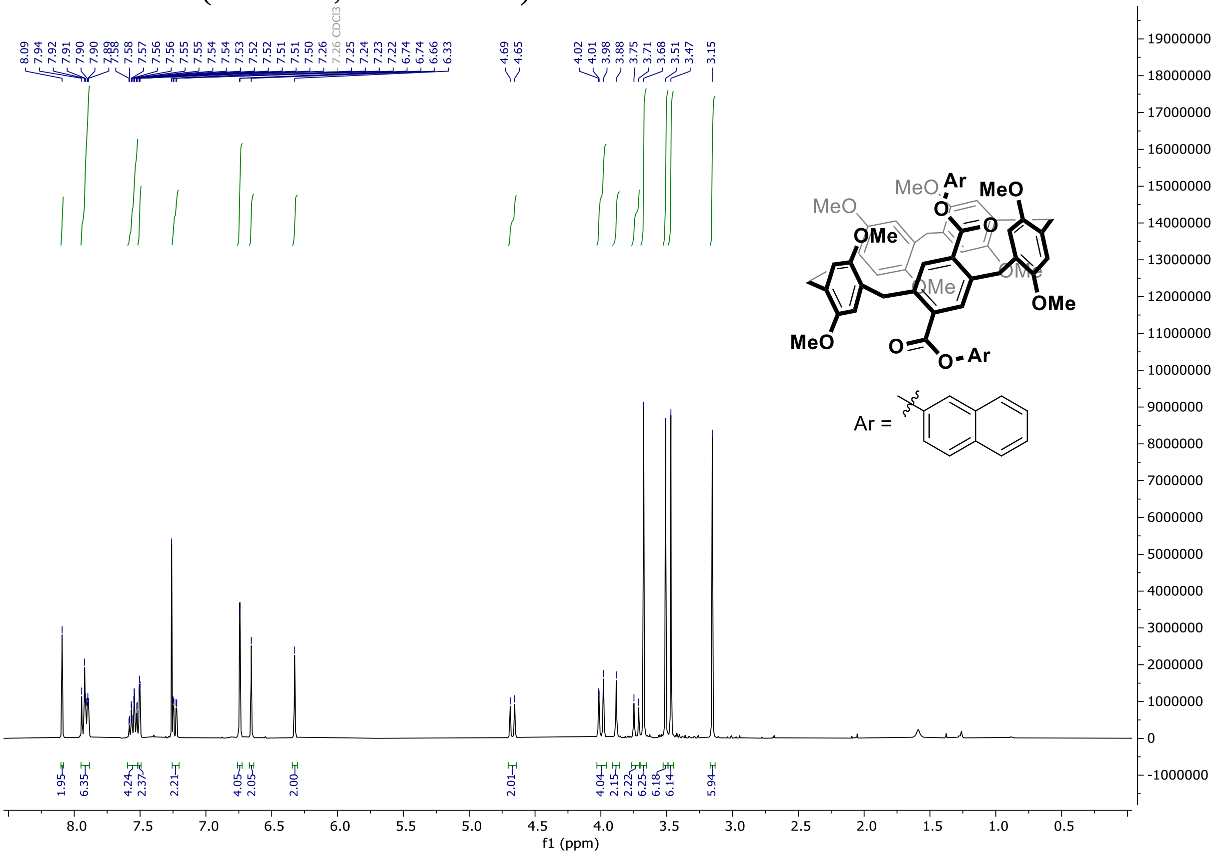

^{13}C{^{1}H} NMR of **3a** (101 MHz, chloroform-*d*)

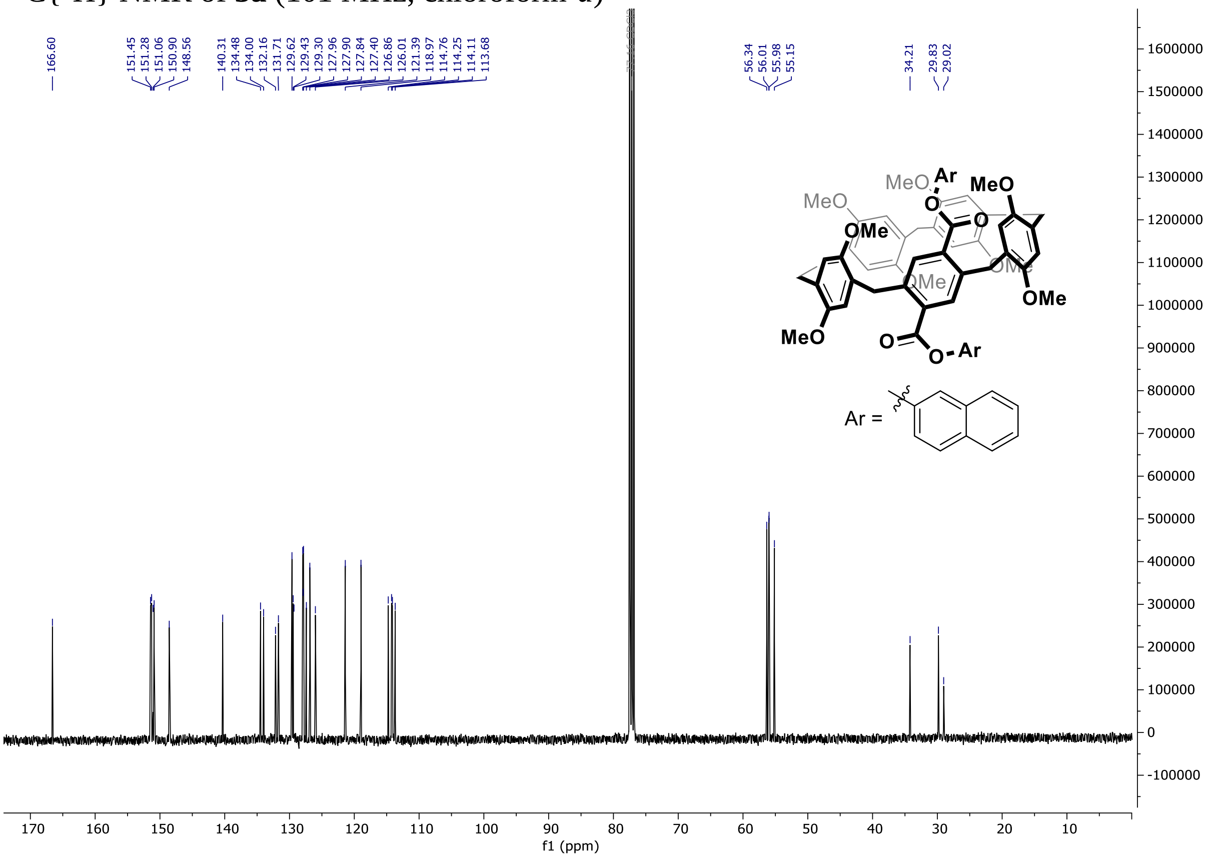

Di(naphthalen-2-yl) (*pS*)-3²,3⁵,5²,5⁵,7²,7⁵,9²,9⁵-octaethoxy-1,3,5,7,9(1,4)-pentabenzenacyclodecaphane-1²,1⁵-dicarboxylate (3b)

^{1}H NMR of **3b** (400 MHz, chloroform-*d*)

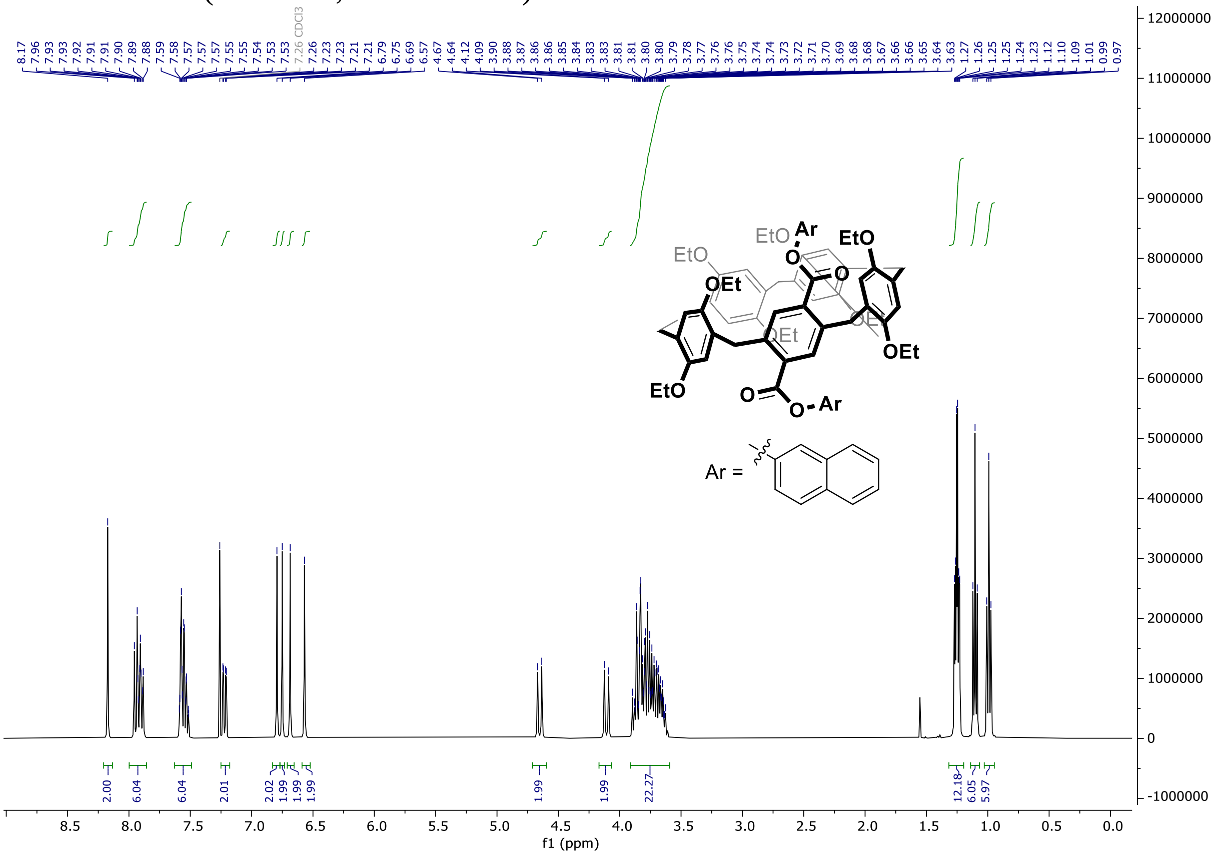

^{13}C{^{1}H} NMR of **3b** (101 MHz, chloroform-*d*)

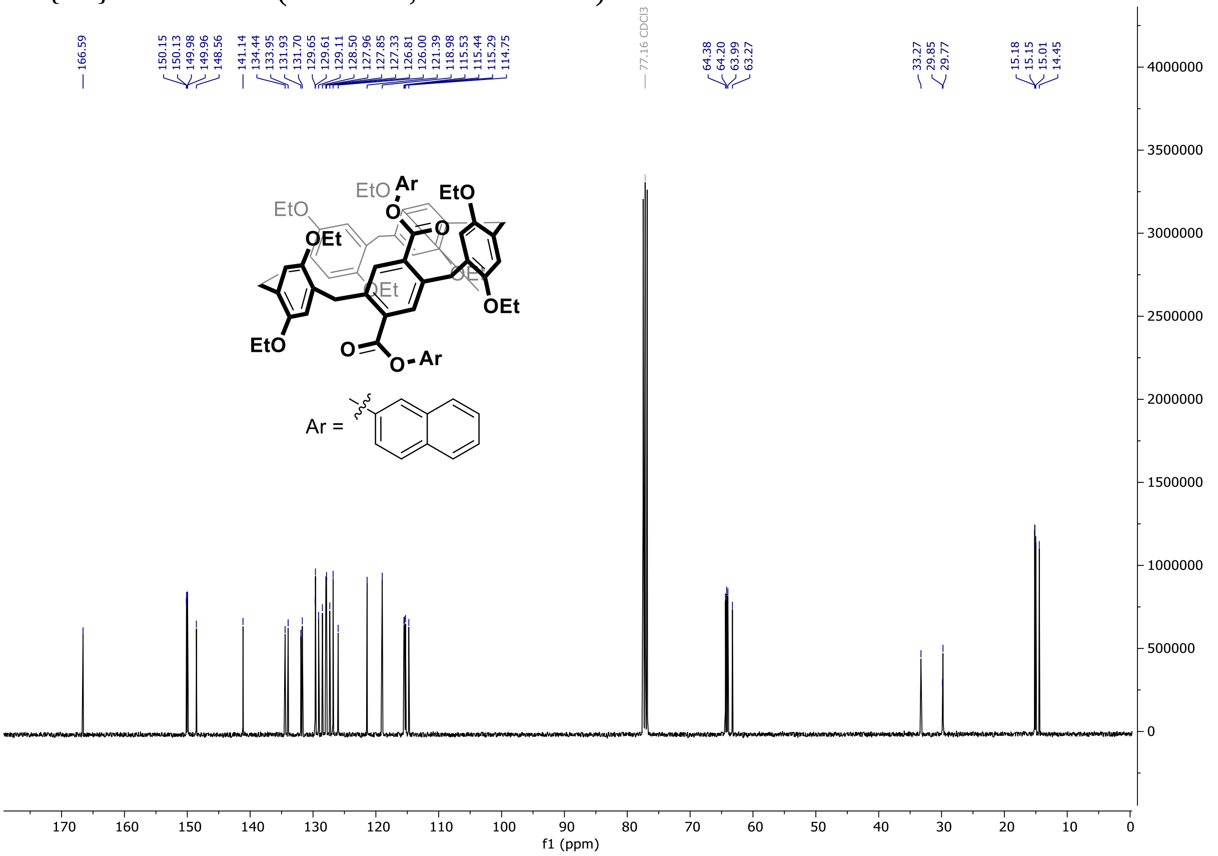

Di(naphthalen-2-yl) (*pS*)-3,3'-($3^2,3^5,5^2,5^5,7^2,7^5,9^2,9^5$-octamethoxy-1,3,5,7,9(1,4)-pentabenzenacyclodecaphane-$1^2,1^5$-diyl)(2*E*,2'*E*)-diacrylate (3c)

^{1}H NMR of **3c** (400 MHz, chloroform-*d*)

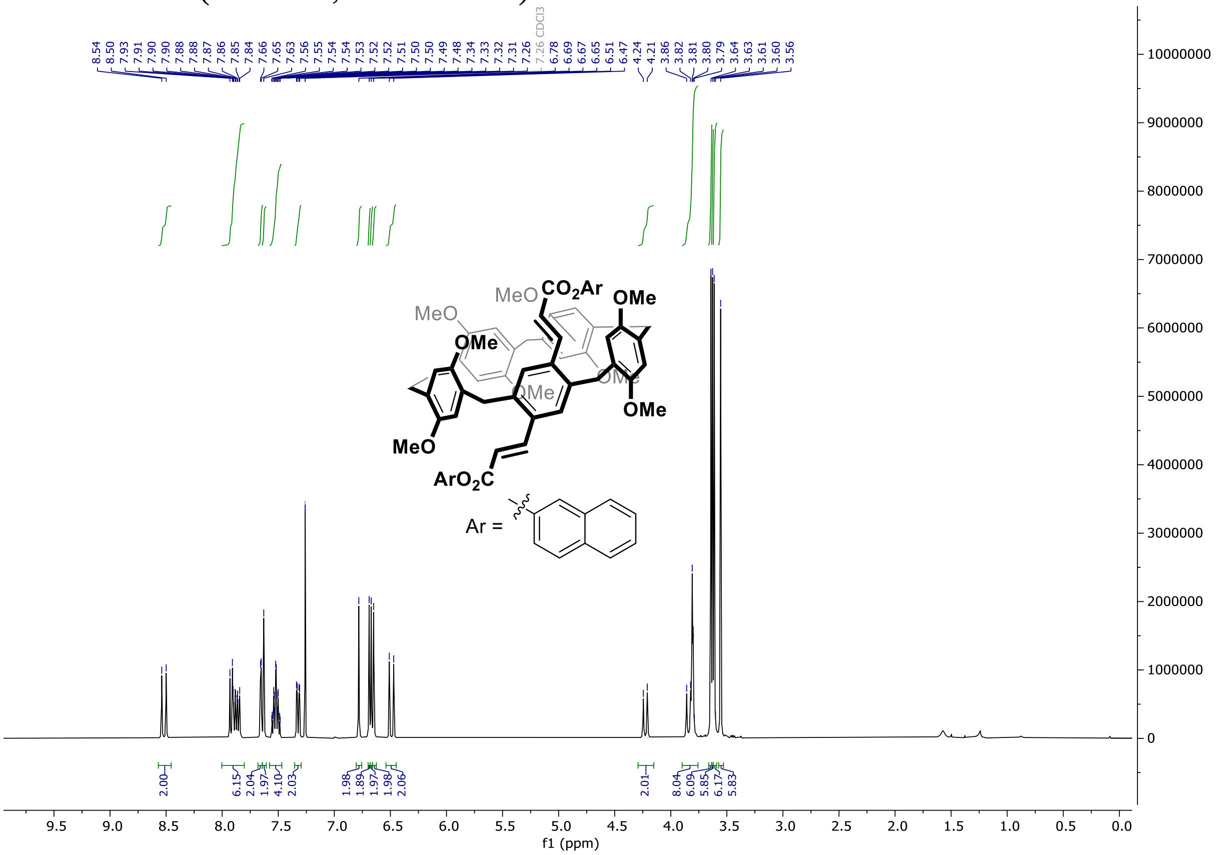

^{13}C{^{1}H} NMR of **3c** (101 MHz, chloroform-*d*)

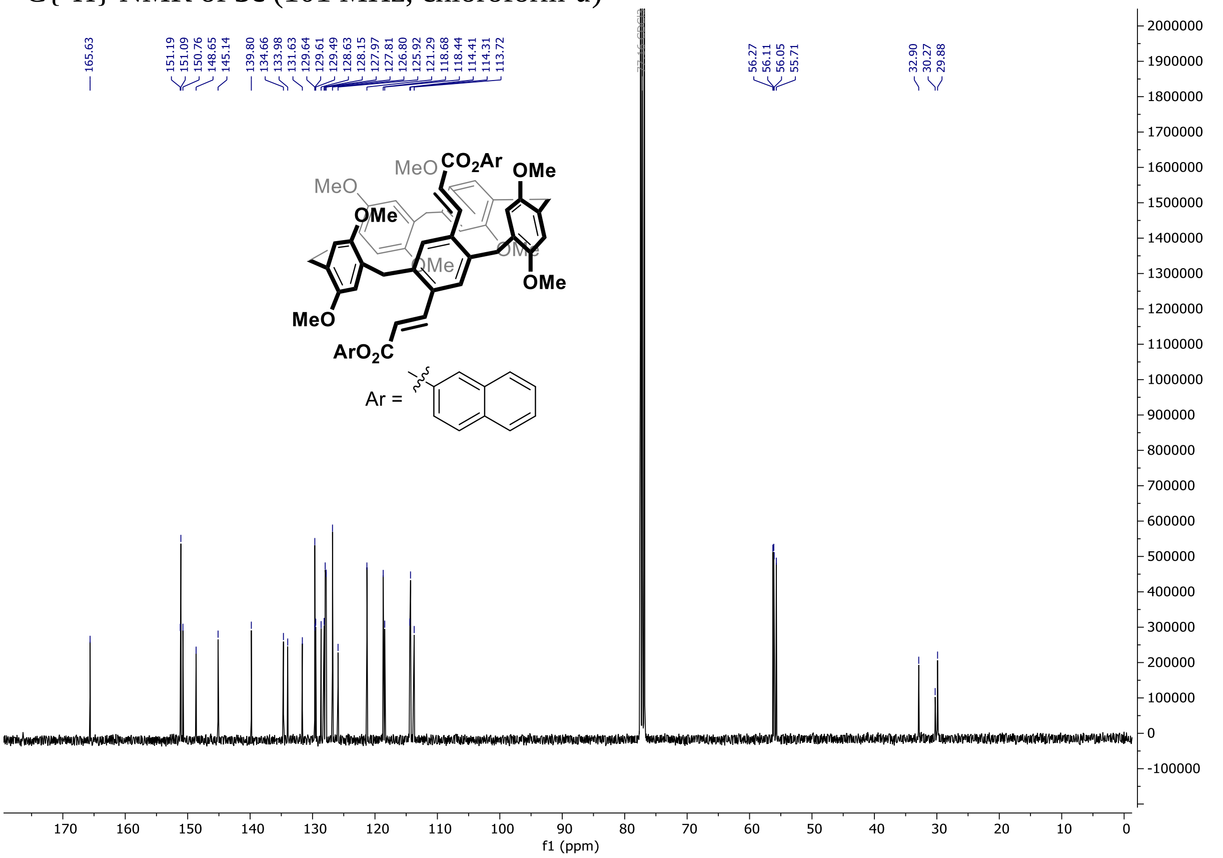

Di(anthracen-2-yl) (*pS*)-$3^2,3^5,5^2,5^5,7^2,7^5,9^2,9^5$-octamethoxy-1,3,5,7,9(1,4)-pentabenzenacyclodecaphane-$1^2,1^5$-dicarboxylate (3d)

^{1}H NMR of **3d** (400 MHz, chloroform-*d*)

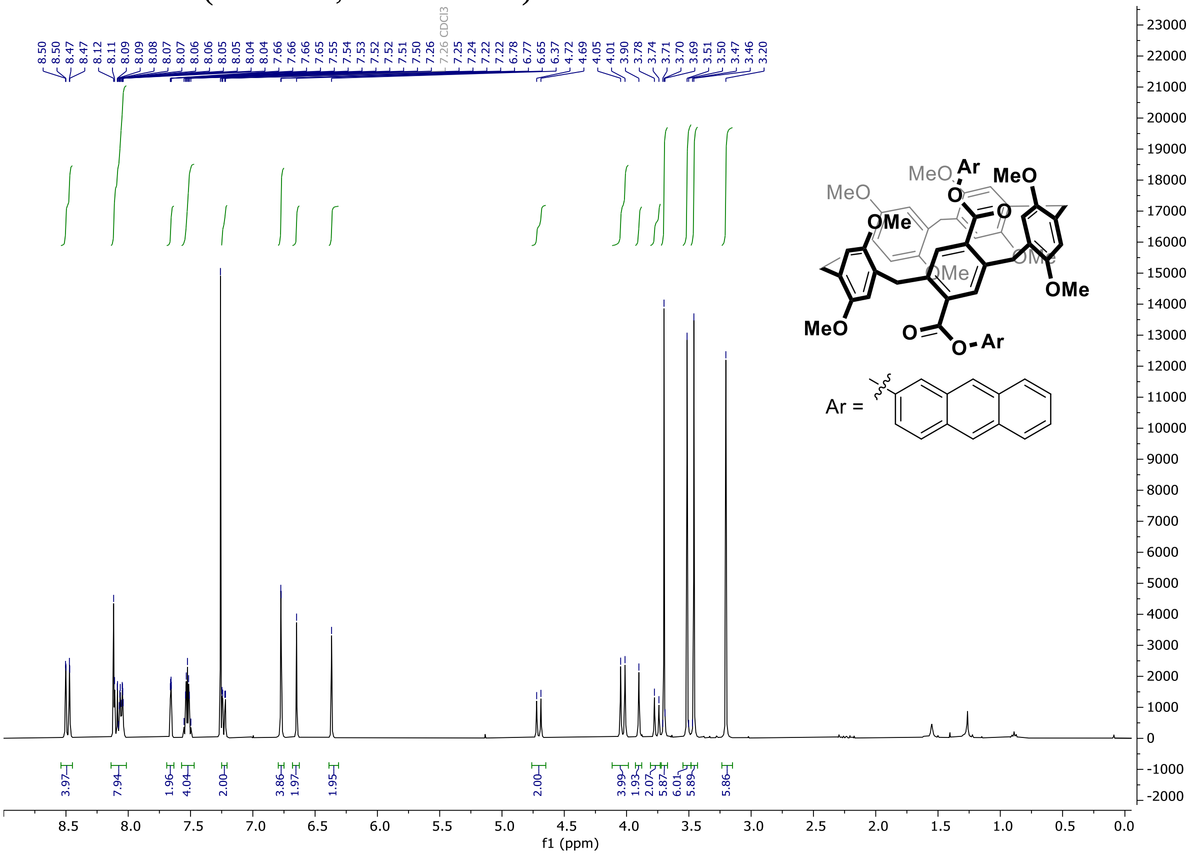

^{13}C{^{1}H} NMR of **3d** (101 MHz, chloroform-*d*)

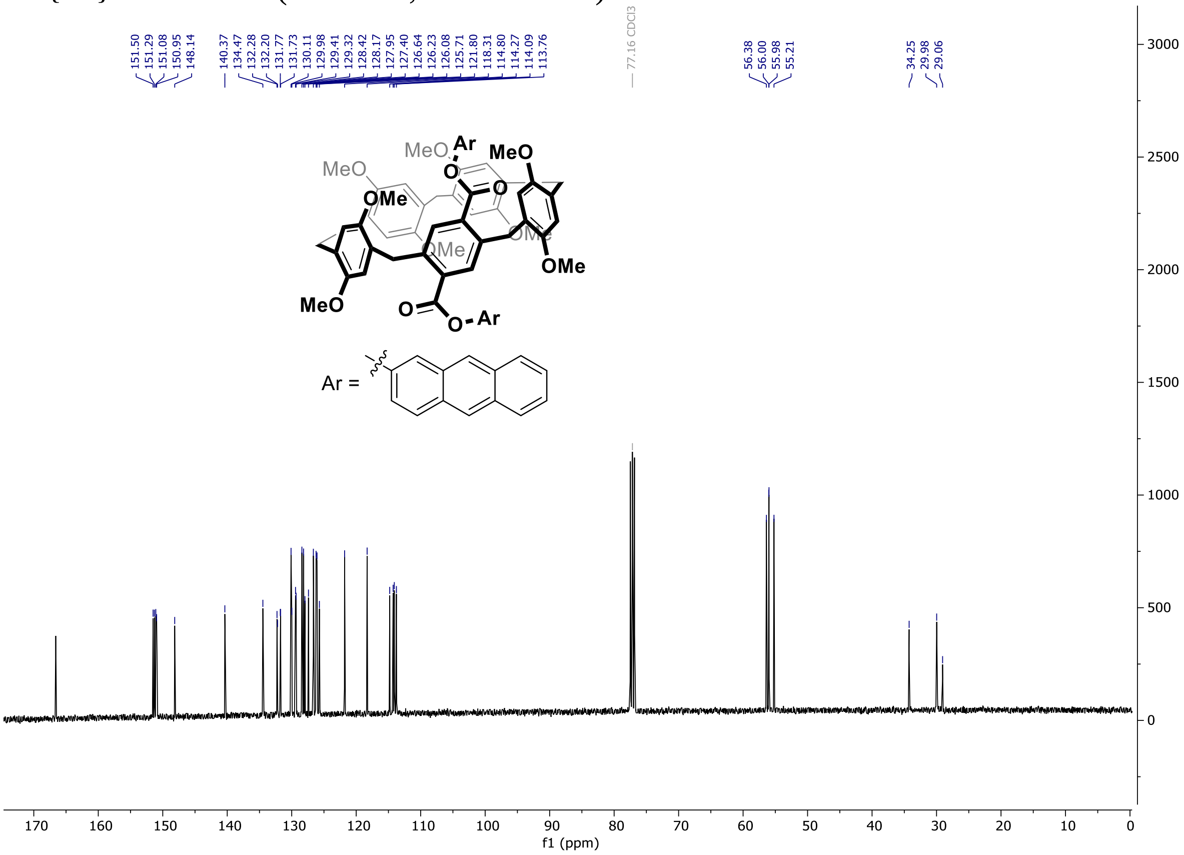

Bis(7-methylnaphthalen-2-yl) (*pS*)-$3^2,3^5,5^2,5^5,7^2,7^5,9^2,9^5$-octamethoxy-1,3,5,7,9(1,4)-pentabenzenacyclodecaphane-$1^2,1^5$-dicarboxylate (3e)

^{1}H NMR of **3e** (400 MHz, chloroform-*d*)

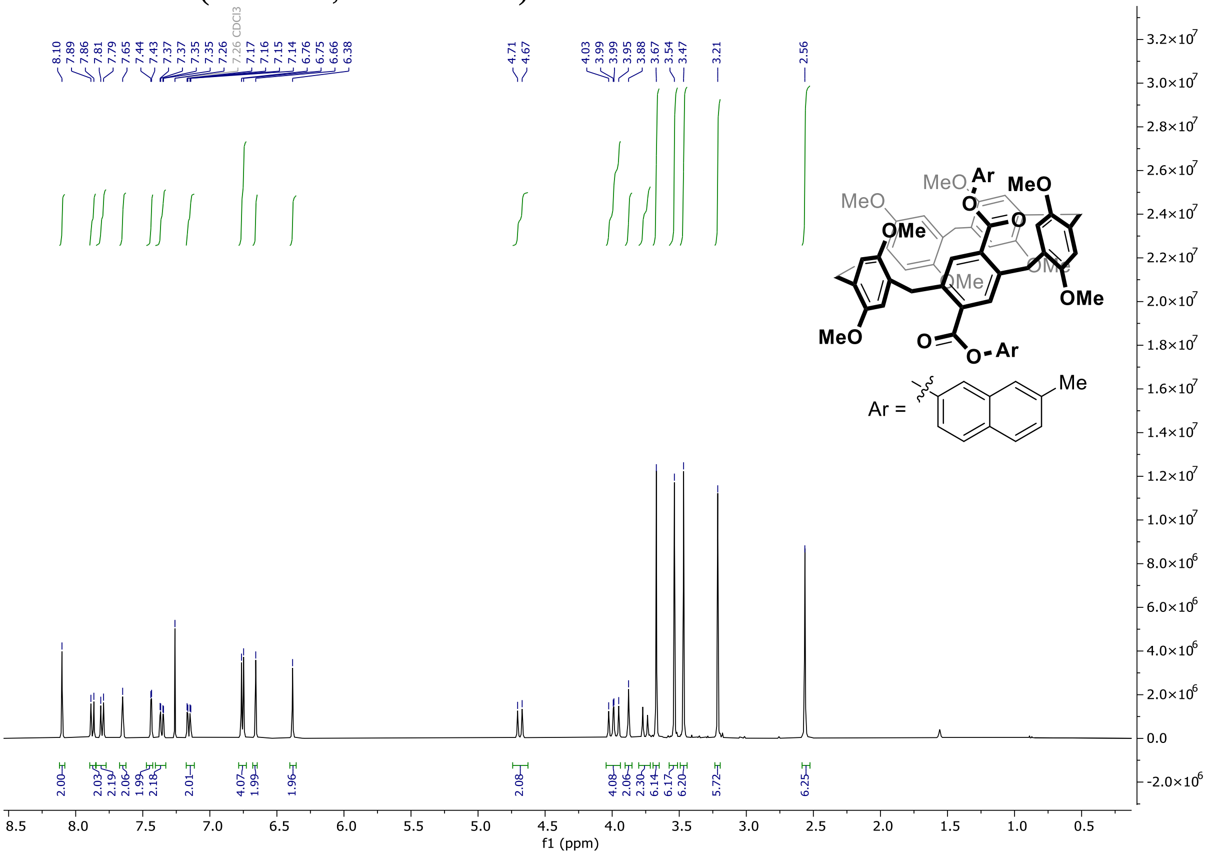

^{13}C{^{1}H} NMR of **3e** (101 MHz, chloroform-*d*)

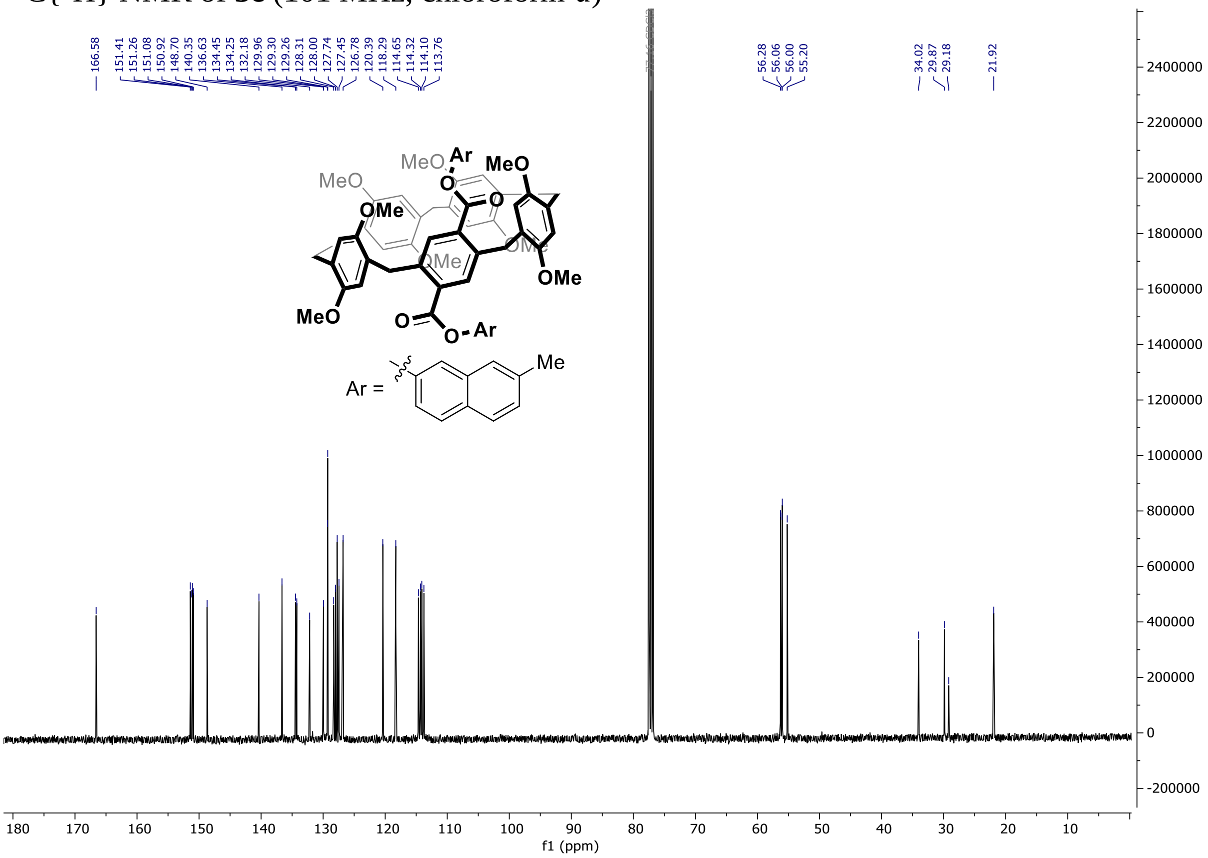

Bis(7-methoxynaphthalen-2-yl) (*pS*)-3^2,3^5,5^2,5^5,7^2,7^5,9^2,9^5-octamethoxy-1,3,5,7,9(1,4)-pentabenzenacyclodecaphane-1^2,1^5-dicarboxylate (3f)

^{1}H NMR of **3f** (400 MHz, chloroform-*d*)

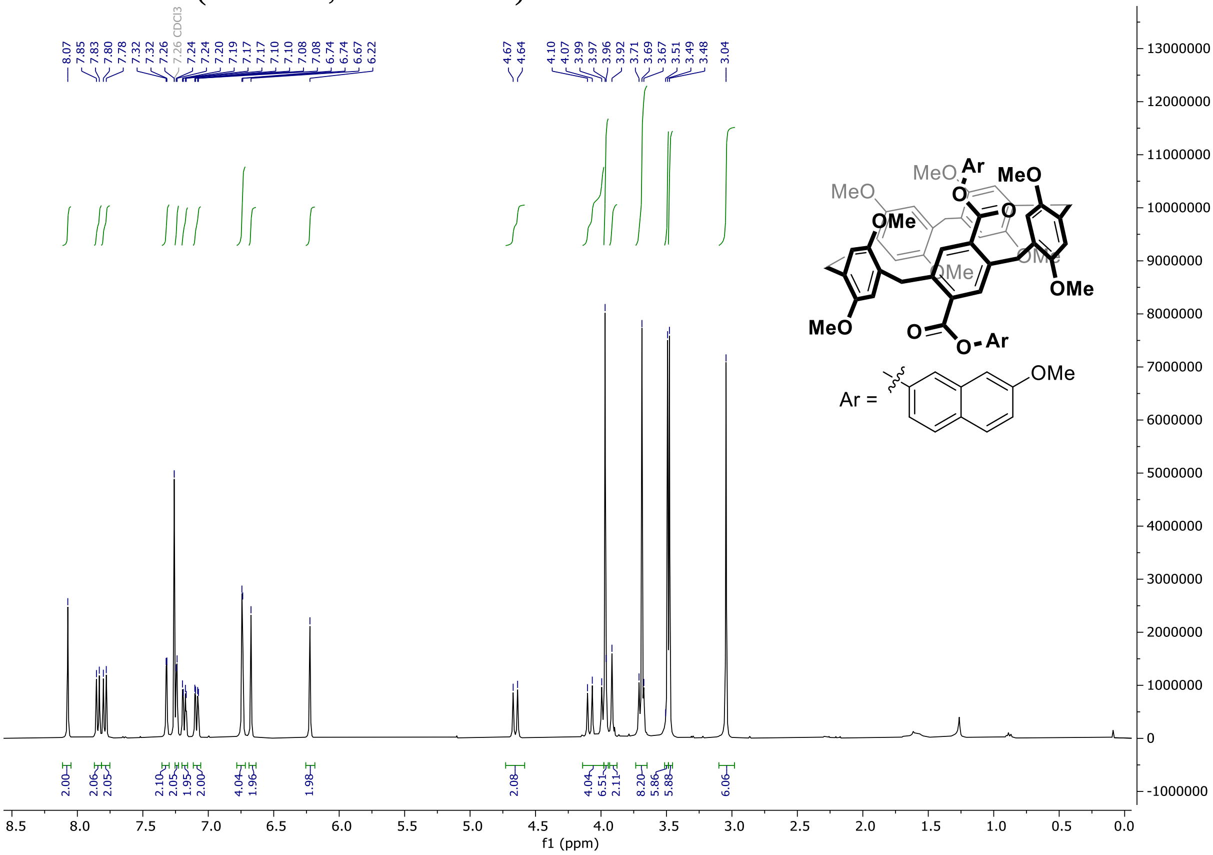

^{13}C{^{1}H} NMR of **3f** (101 MHz, chloroform-*d*)

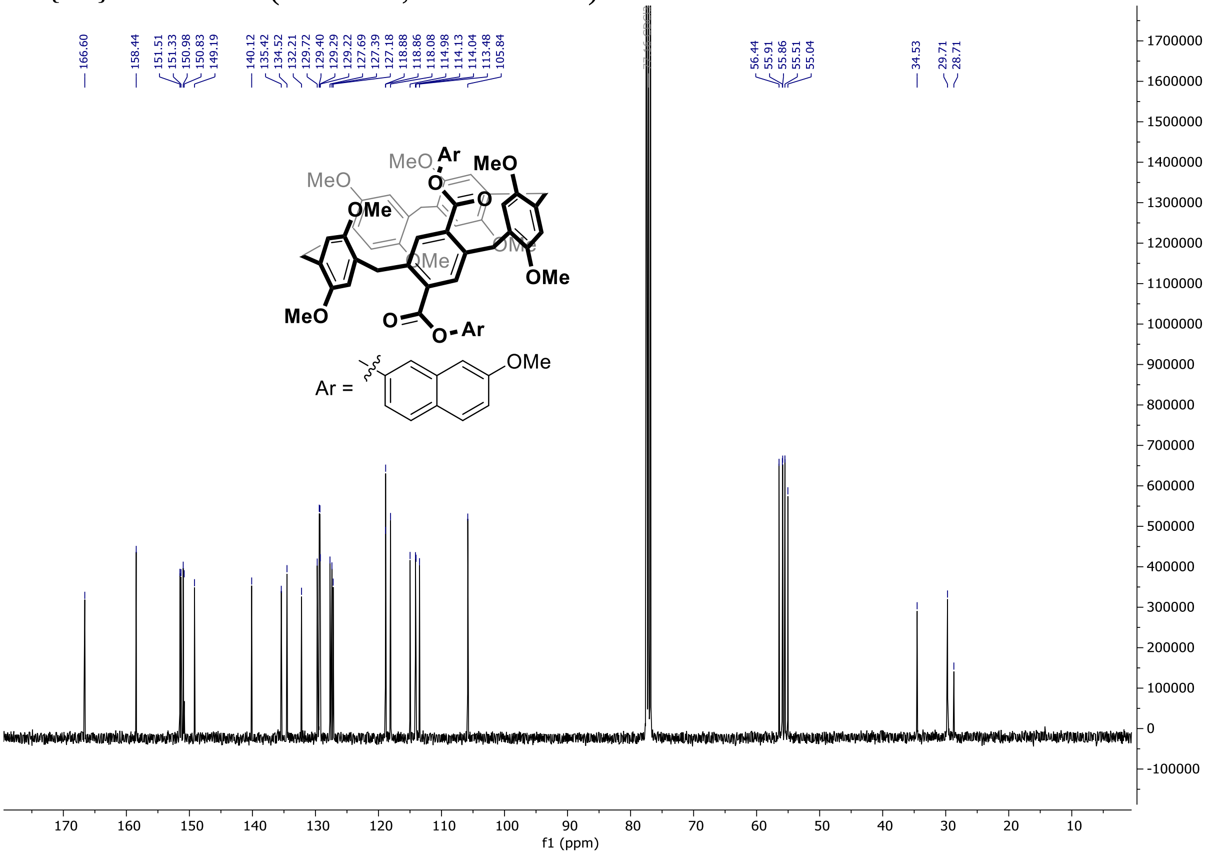

Bis(7-(methoxycarbonyl)naphthalen-2-yl) (*pS*)-$3^2,3^5,5^2,5^5,7^2,7^5,9^2,9^5$-octamethoxy-1,3,5,7,9(1,4)-pentabenzenacyclodecaphane-$1^2,1^5$-dicarboxylate (3g)

^{1}H NMR of **3g** (400 MHz, chloroform-*d*)

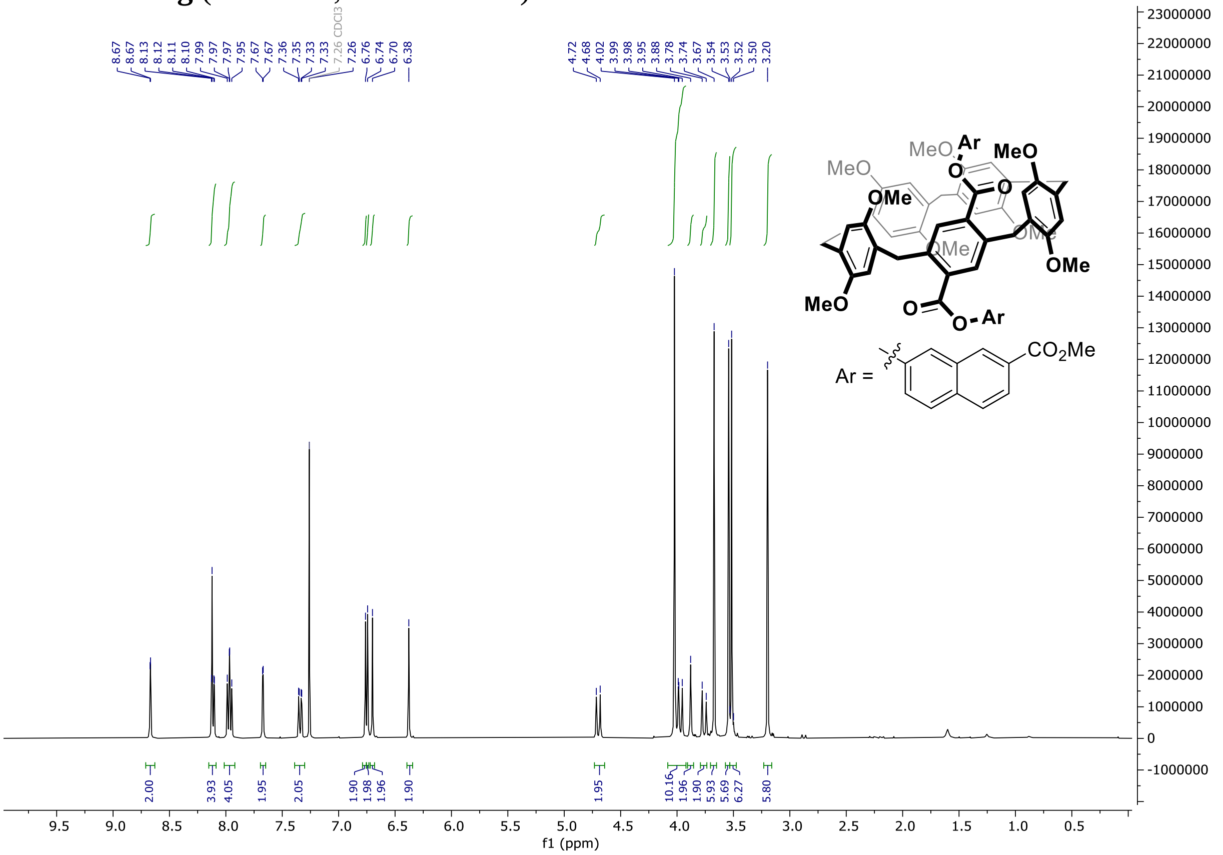

^{13}C{^{1}H} NMR of **3g** (101 MHz, chloroform-*d*)

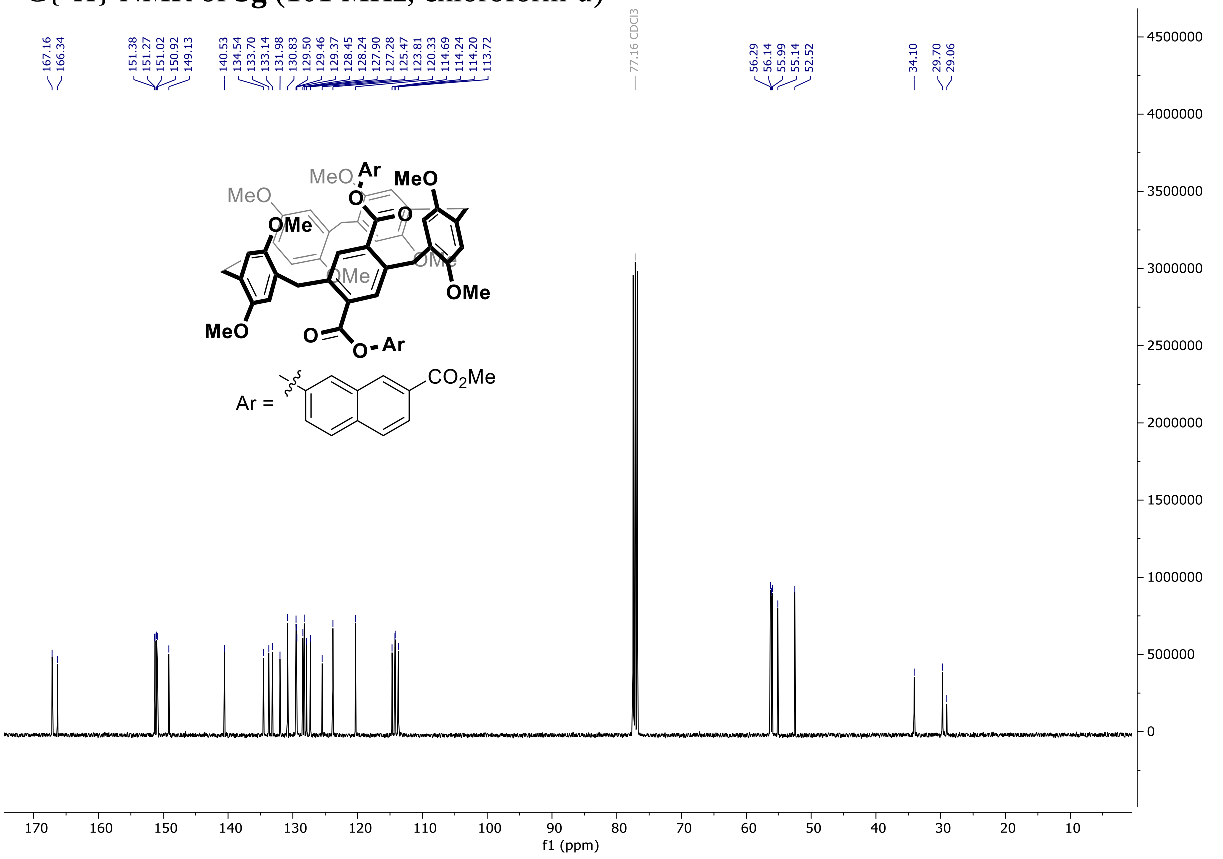

Bis(7-cyanonaphthalen-2-yl) (*pS*)-3²,3⁵,5²,5⁵,7²,7⁵,9²,9⁵-octamethoxy-1,3,5,7,9(1,4)-pentabenzenacyclodecaphane-1²,1⁵-dicarboxylate (3h)

^{1}H NMR of **3h** (400 MHz, chloroform-*d*)

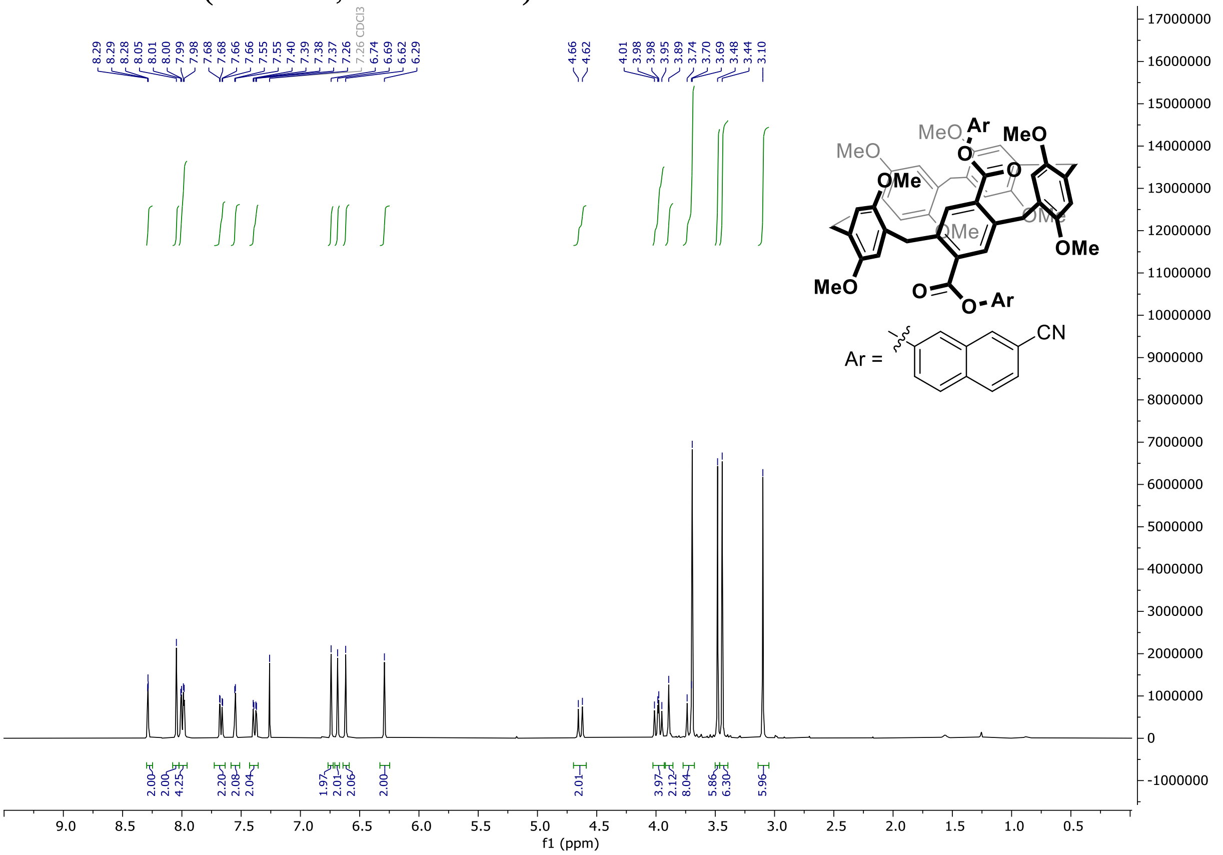

^{13}C{^{1}H} NMR of **3h** (101 MHz, chloroform-*d*)

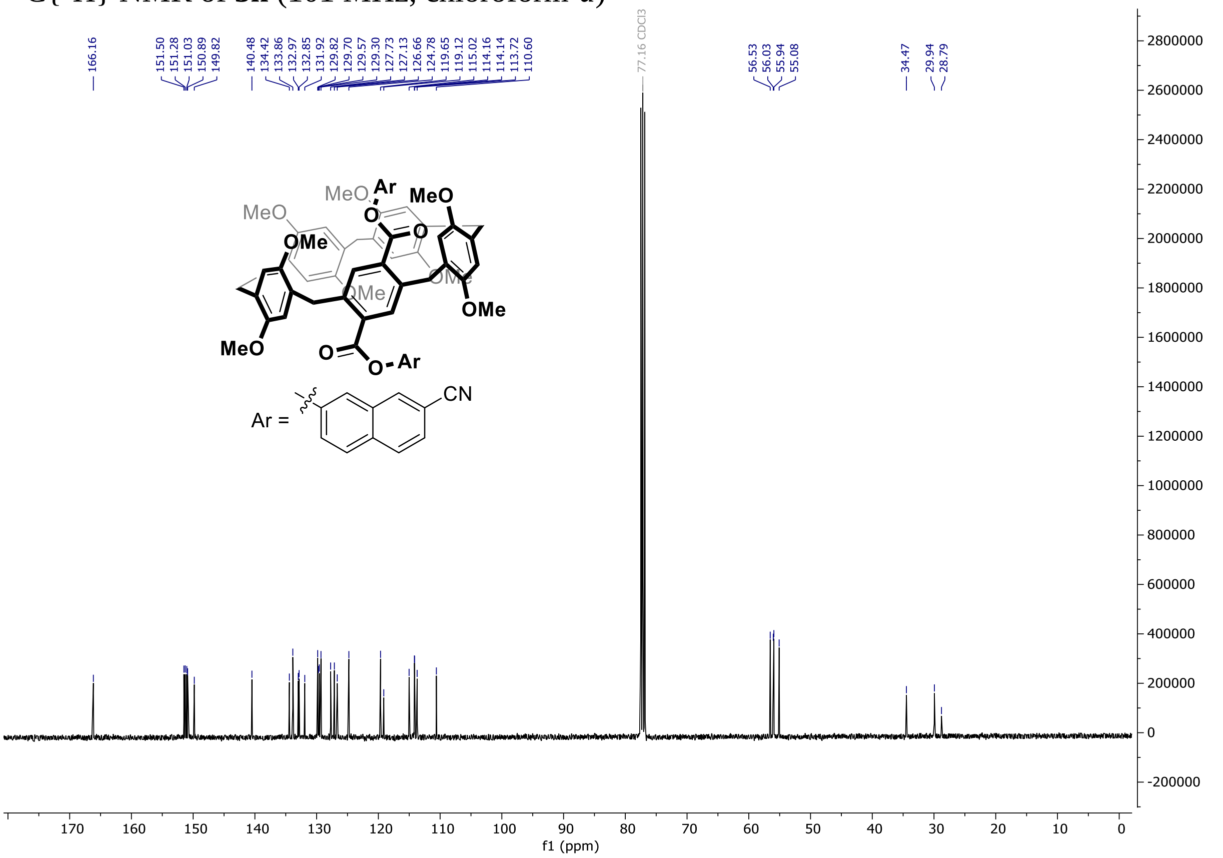

Bis(7-fluoronaphthalen-2-yl) (*pS*)-$3^2,3^5,5^2,5^5,7^2,7^5,9^2,9^5$-octamethoxy-1,3,5,7,9(1,4)-pentabenzenacyclodecaphane-$1^2,1^5$-dicarboxylate (3i)

^{1}H NMR of **3i** (400 MHz, chloroform-*d*)

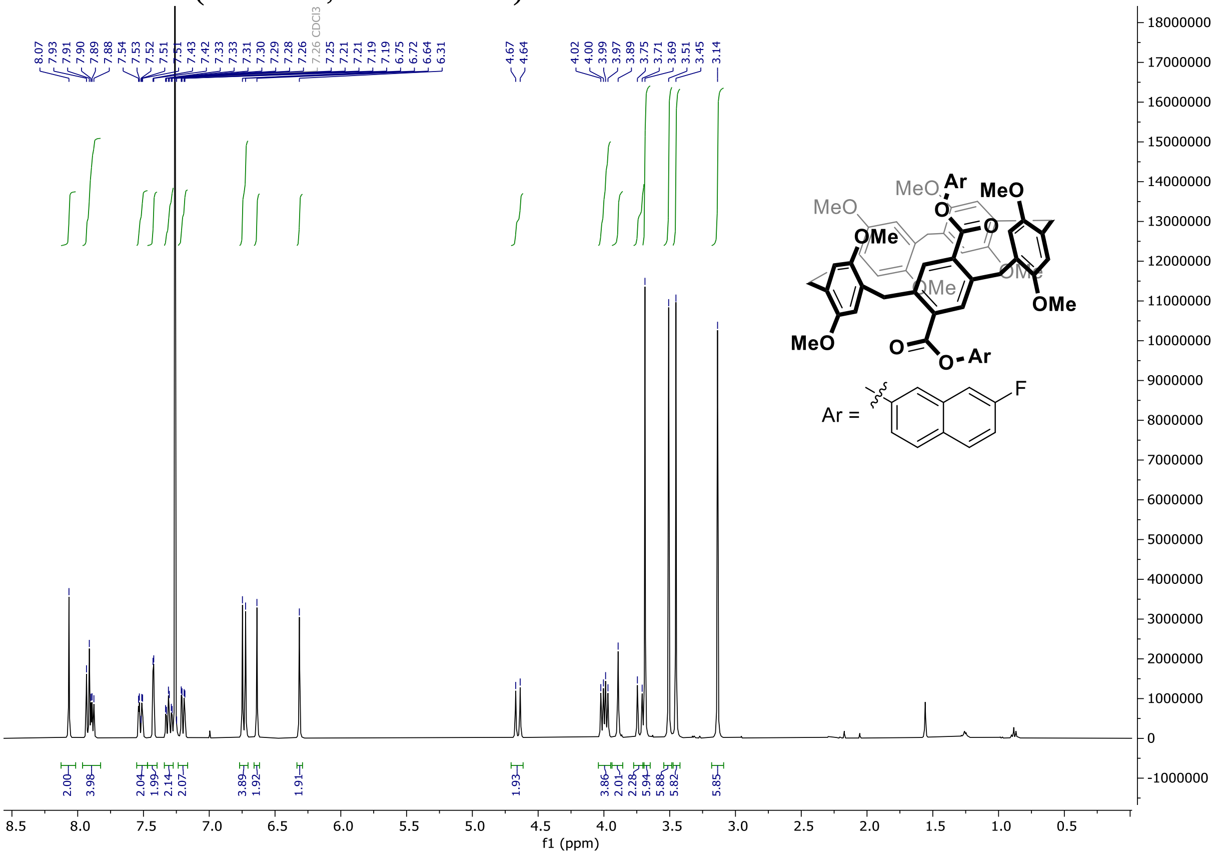

^{13}C{^{1}H} NMR of **3i** (101 MHz, chloroform-*d*)

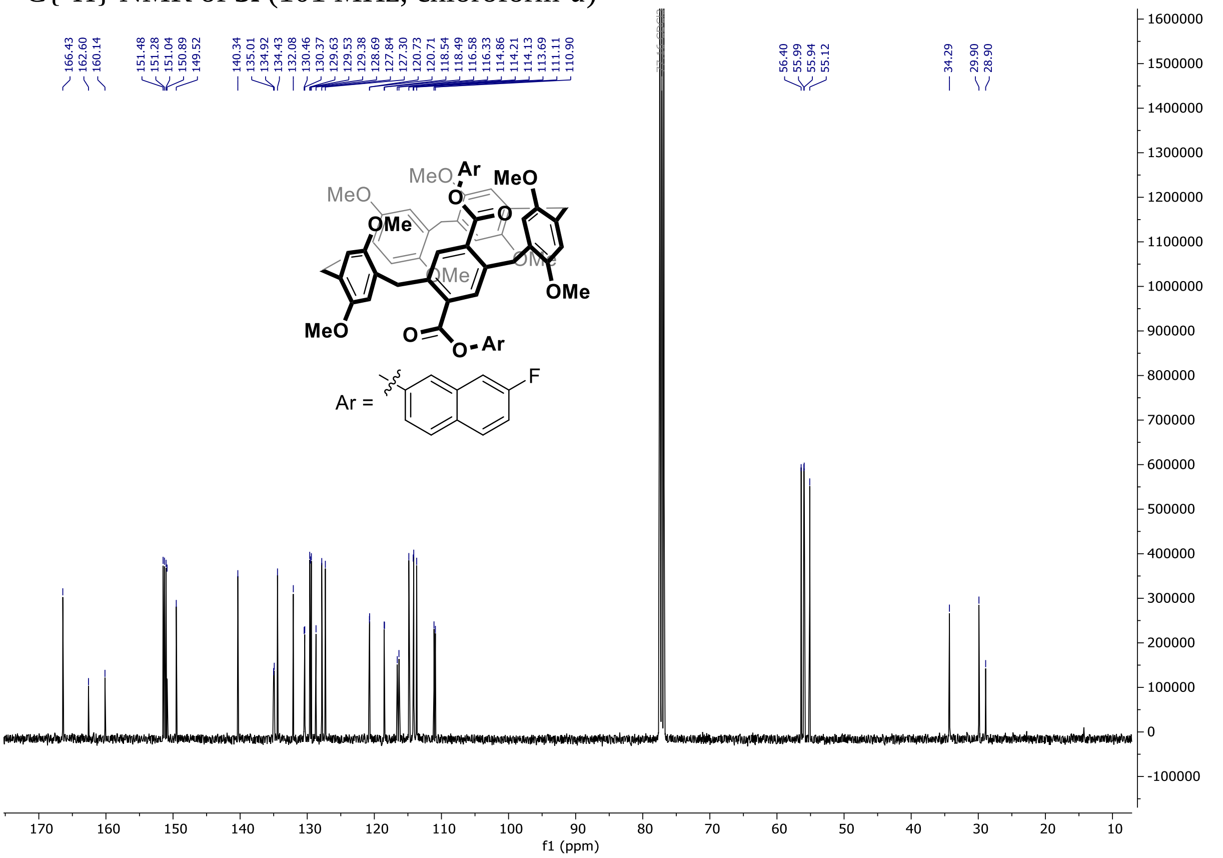

^{19}F NMR of **3i** (376 MHz, chloroform-*d*)

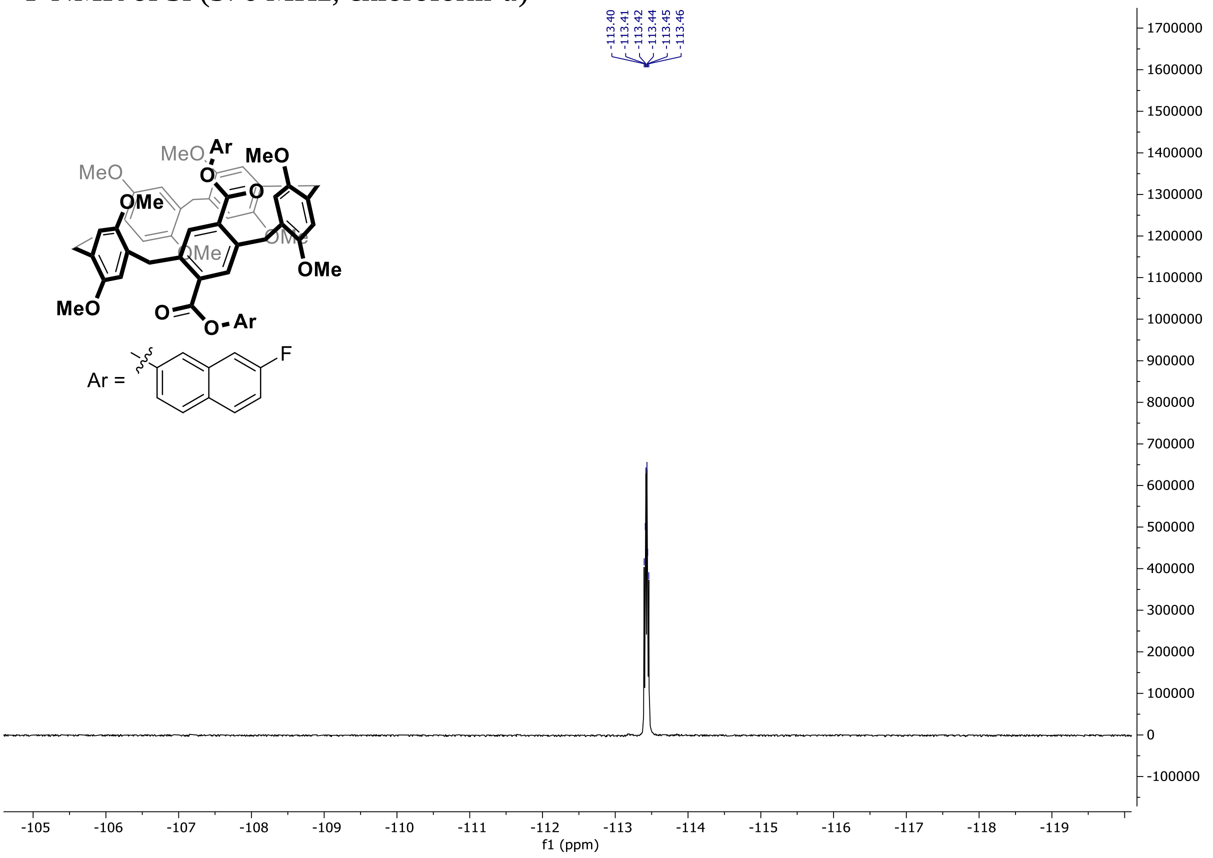

Bis(7-chloronaphthalen-2-yl) (*pS*)-$3^2,3^5,5^2,5^5,7^2,7^5,9^2,9^5$-octamethoxy-1,3,5,7,9(1,4)-pentabenzenacyclodecaphane-$1^2,1^5$-dicarboxylate (3j)

^{1}H NMR of **3j** (400 MHz, chloroform-*d*)

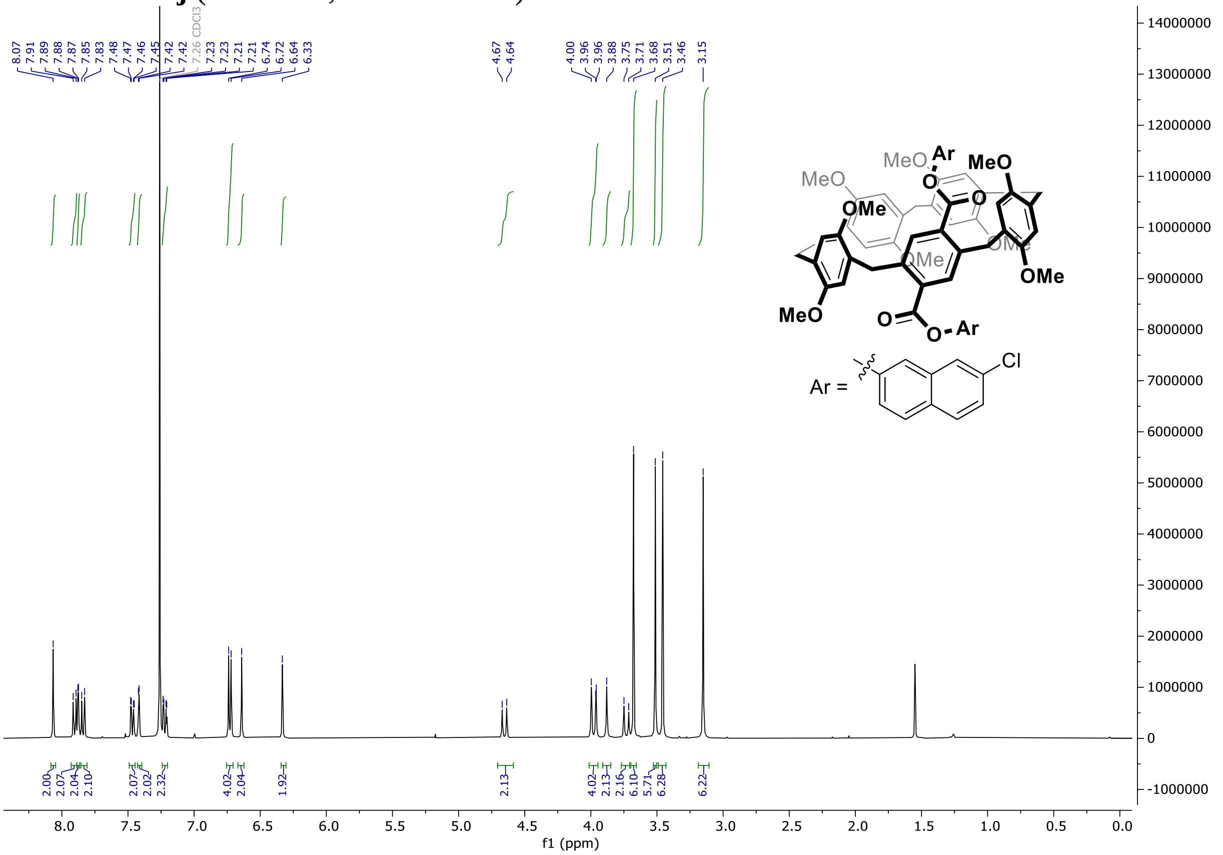

^{13}C{^{1}H} NMR of **3j** (101 MHz, chloroform-*d*)

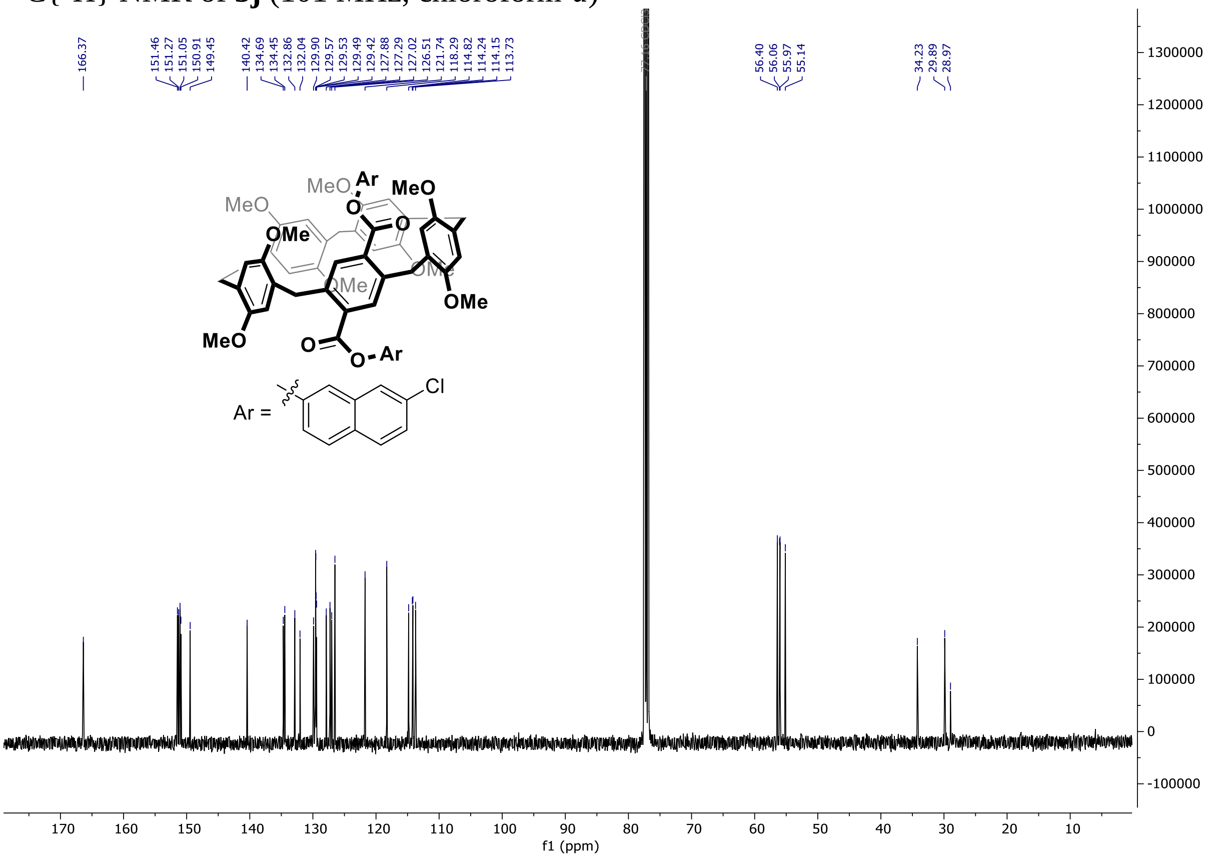

Bis(7-bromonaphthalen-2-yl) (*pS*)-3^2,3^5,5^2,5^5,7^2,7^5,9^2,9^5-octamethoxy-1,3,5,7,9(1,4)-pentabenzenacyclodecaphane-1^2,1^5-dicarboxylate (3k)

^{1}H NMR of **3k** (400 MHz, chloroform-*d*)

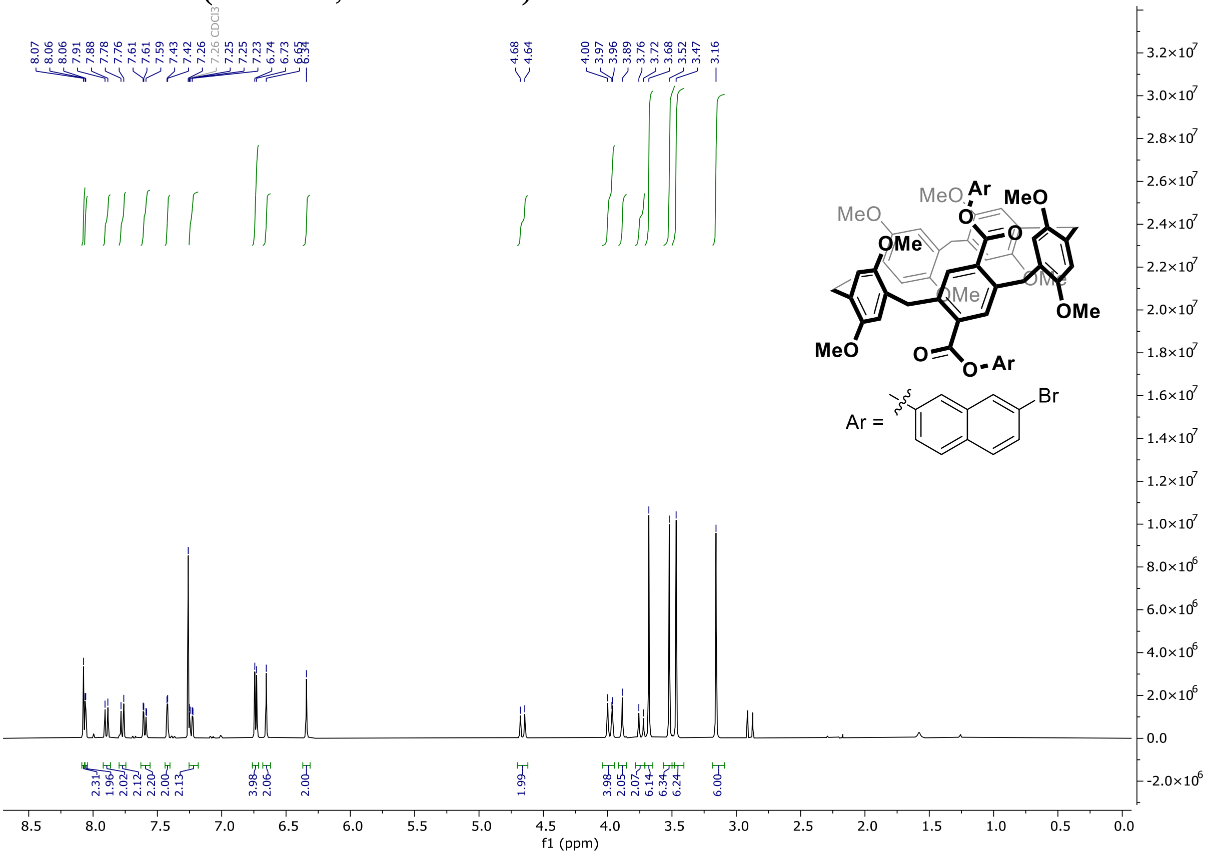

^{13}C{^{1}H} NMR of **3k** (101 MHz, chloroform-*d*)

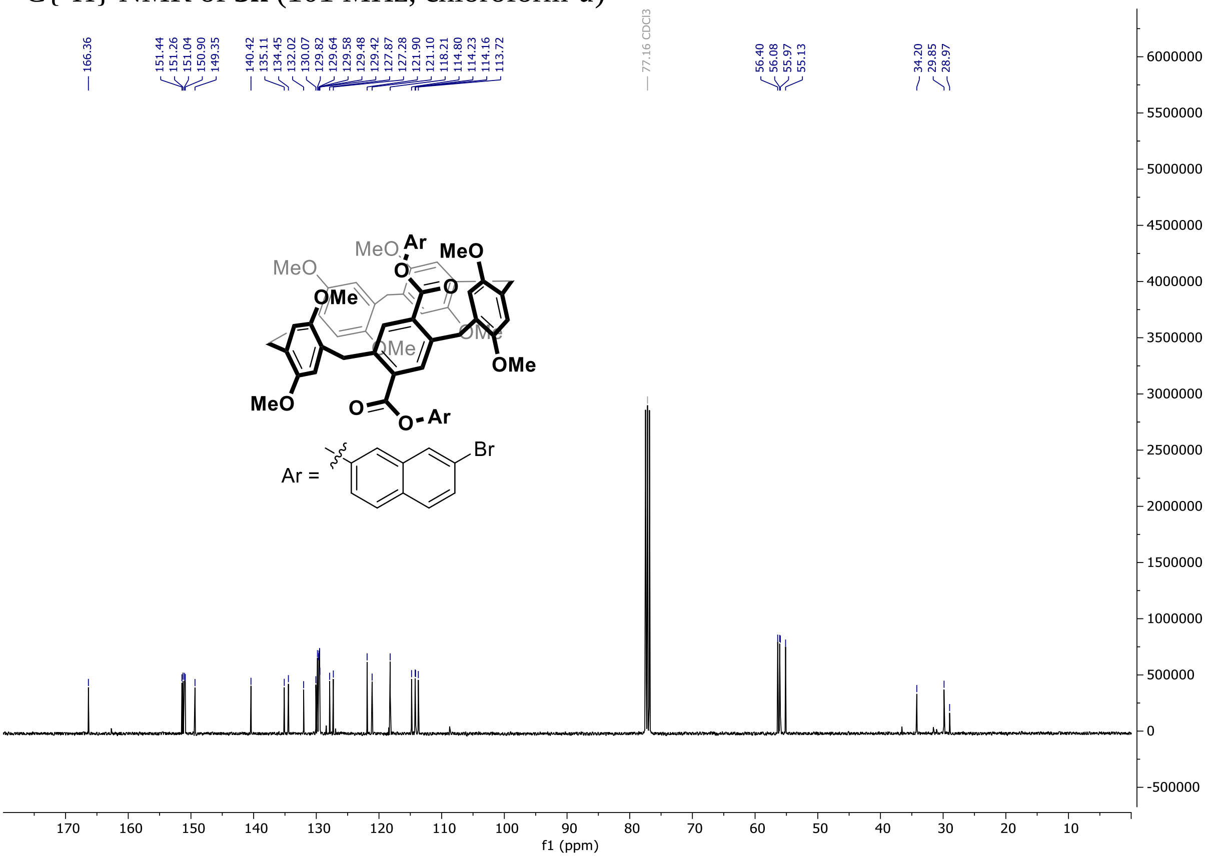

Bis(6-methylnaphthalen-2-yl) (*pS*)-$3^2,3^5,5^2,5^5,7^2,7^5,9^2,9^5$-octamethoxy-1,3,5,7,9(1,4)-pentabenzenacyclodecaphane-$1^2,1^5$-dicarboxylate (3l)

^{1}H NMR of **3l** (400 MHz, chloroform-*d*)

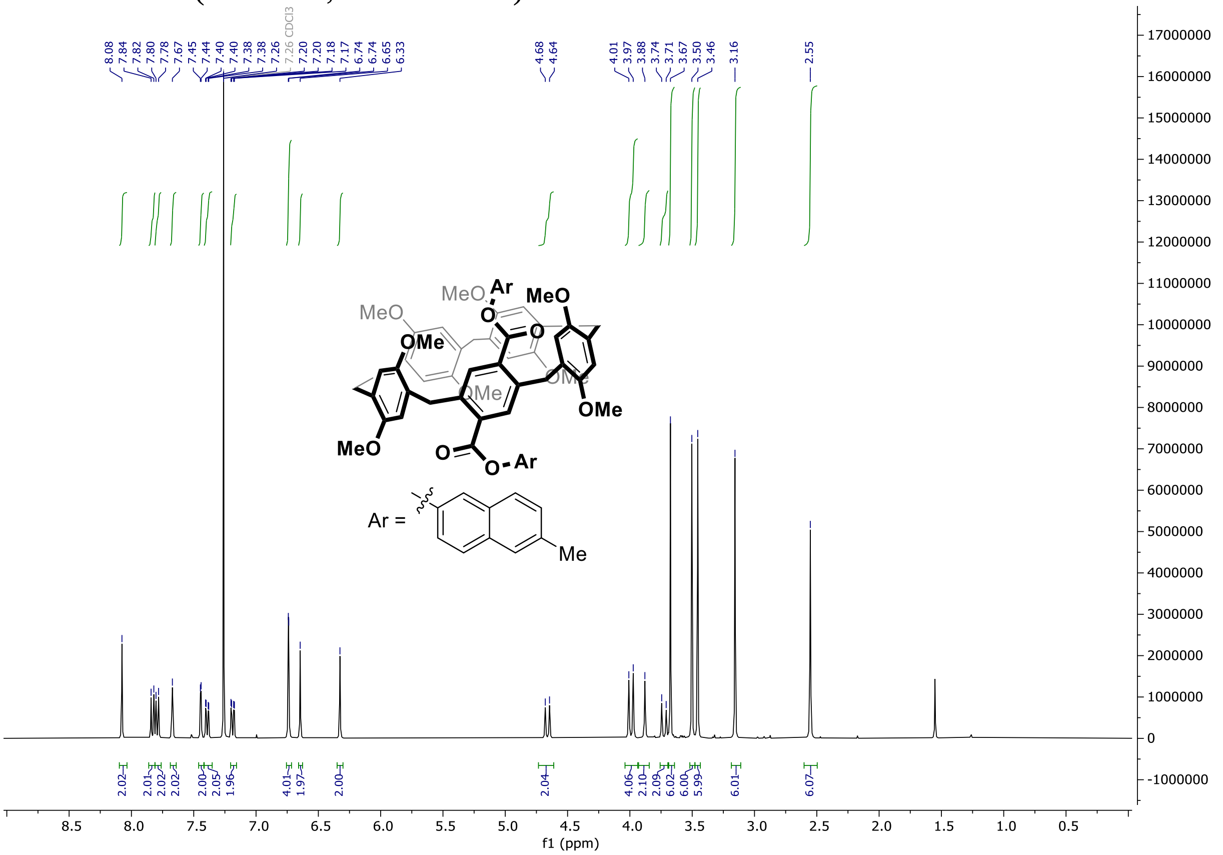

^{13}C{^{1}H} NMR of **3l** (101 MHz, chloroform-*d*)

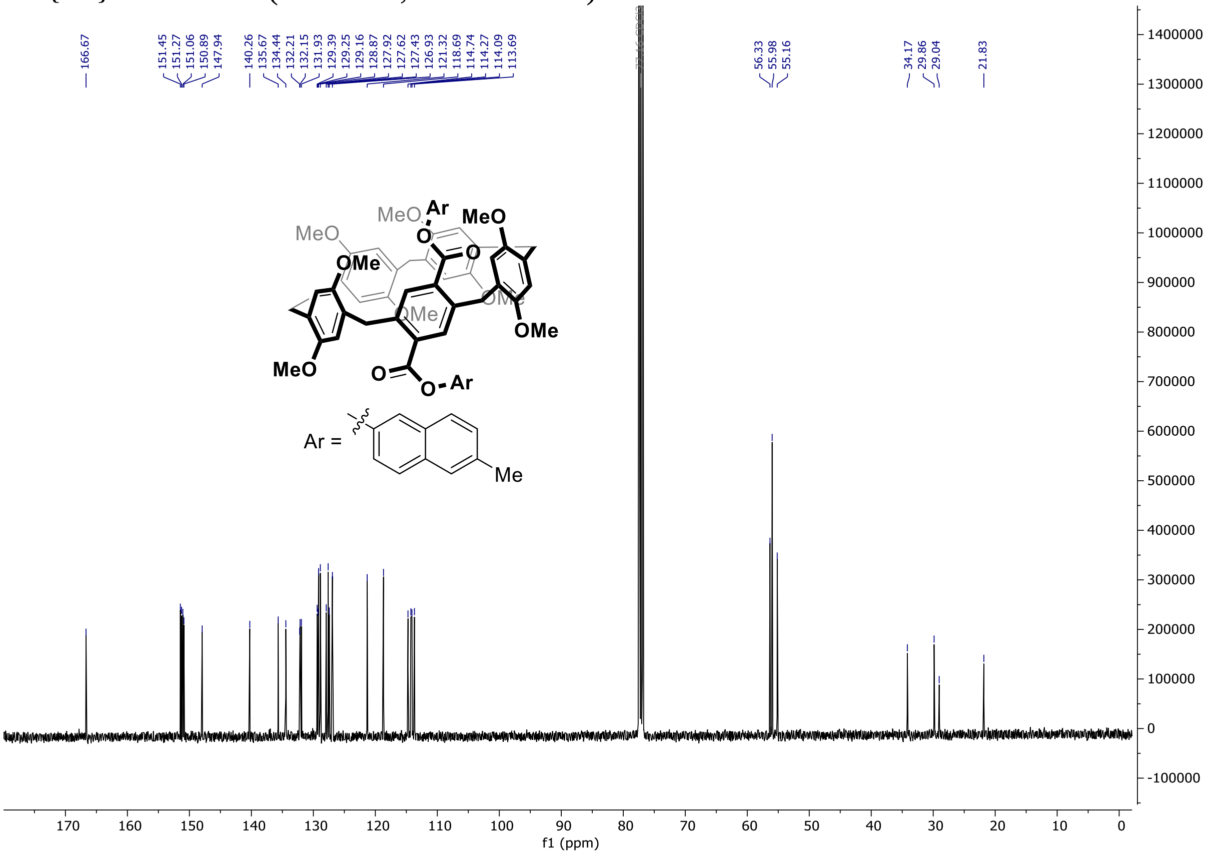

Bis(6-methoxynaphthalen-2-yl) (*pS*)-3²,3⁵,5²,5⁵,7²,7⁵,9²,9⁵-octamethoxy-1,3,5,7,9(1,4)-pentabenzenacyclodecaphane-1²,1⁵-dicarboxylate (3m)

^{1}H NMR of **3m** (400 MHz, chloroform-*d*)

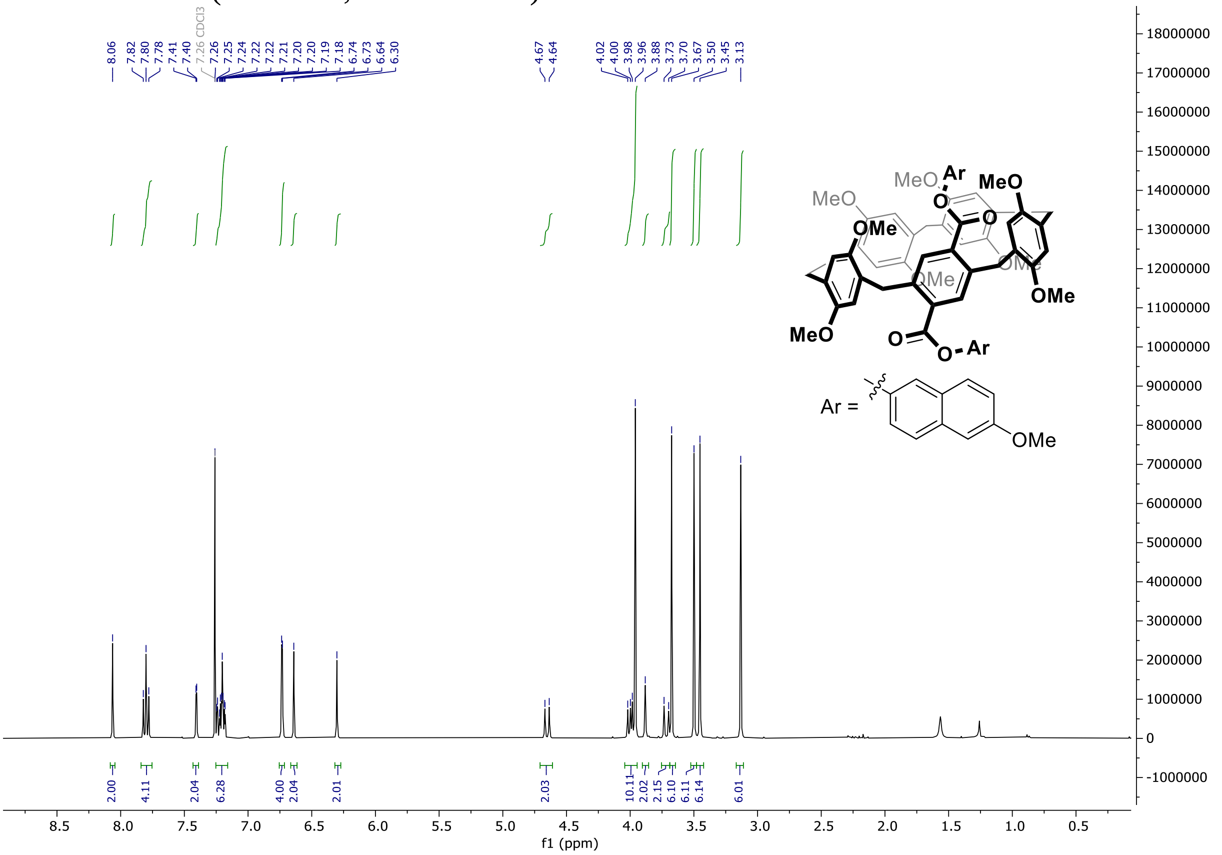

^{13}C{^{1}H} NMR of **3m** (101 MHz, chloroform-*d*)

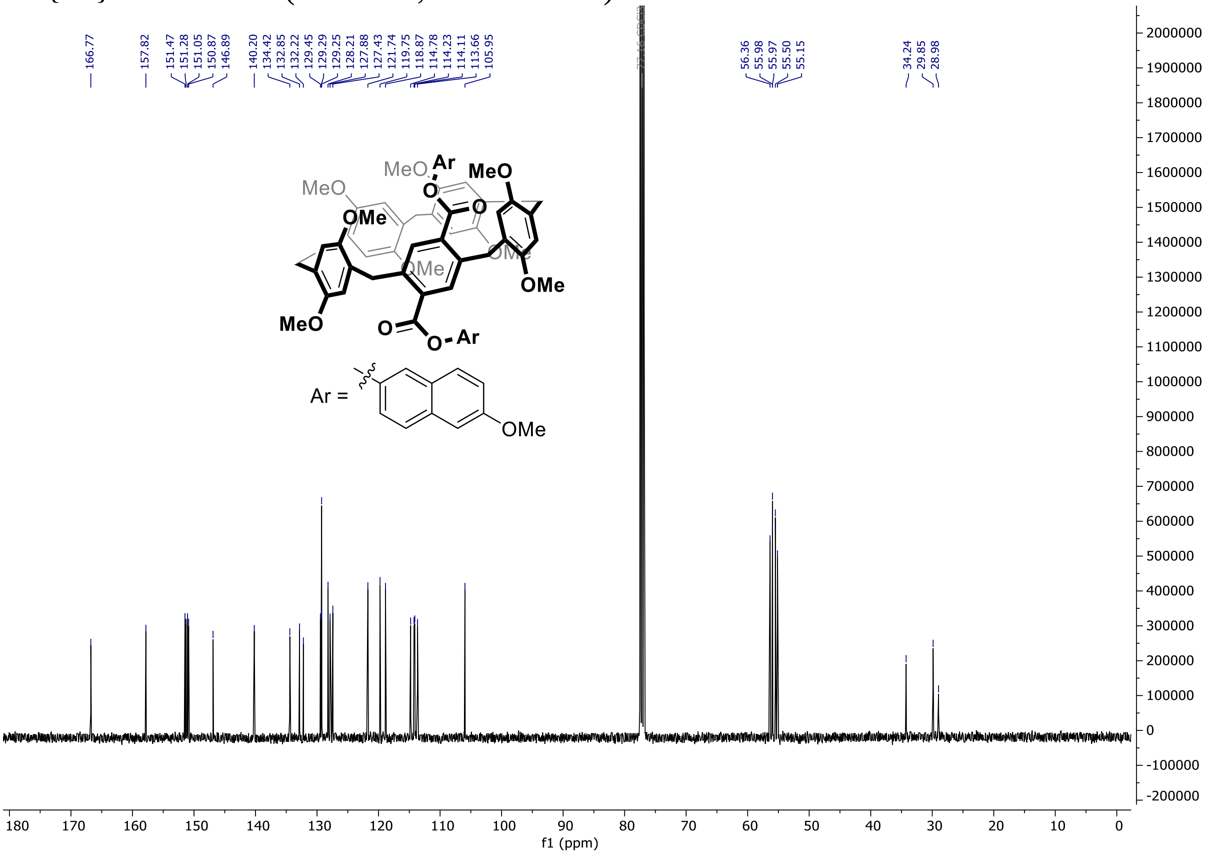

Bis(6-(methoxycarbonyl)naphthalen-2-yl) (*pS*)-$3^2,3^5,5^2,5^5,7^2,7^5,9^2,9^5$-octamethoxy-1,3,5,7,9(1,4)-pentabenzenacyclodecaphane-$1^2,1^5$-dicarboxylate (3n)

^{1}H NMR of **3n** (400 MHz, chloroform-*d*)

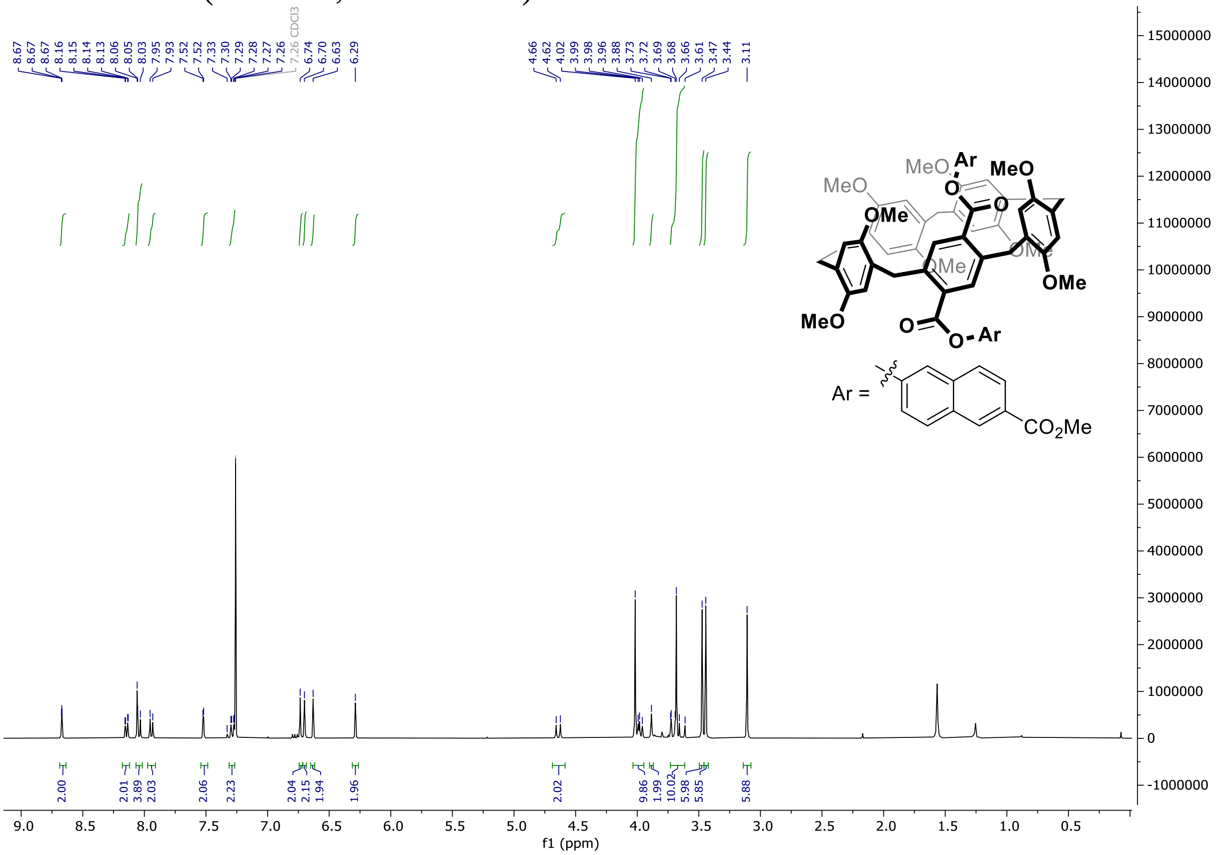

^{13}C{^{1}H} NMR of **3n** (101 MHz, chloroform-*d*)

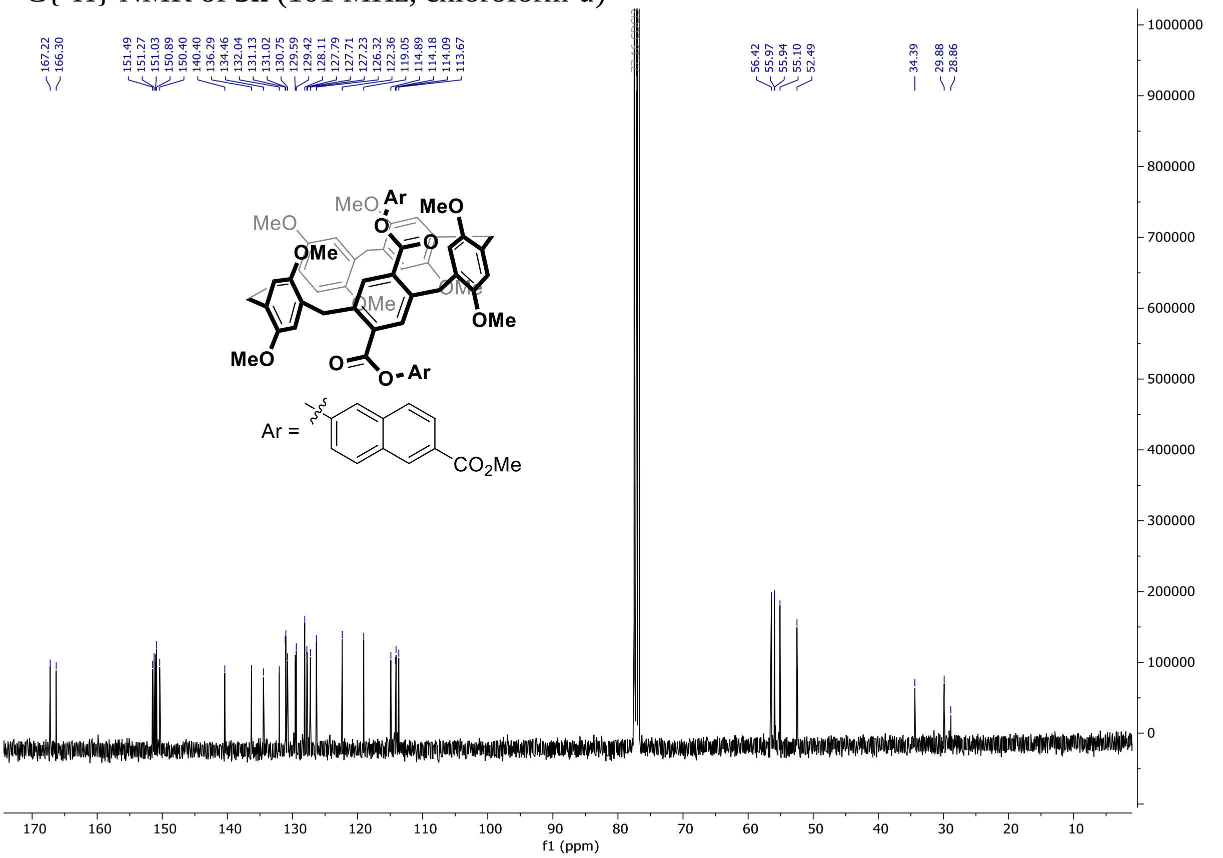

Bis(6-(1,3-dioxolan-2-yl)naphthalen-2-yl) (*pS*)-$3^2,3^5,5^2,5^5,7^2,7^5,9^2,9^5$-octamethoxy-1,3,5,7,9(1,4)-pentabenzenacyclodecaphane-$1^2,1^5$-dicarboxylate (3o)

^{1}H NMR of **3o** (400 MHz, chloroform-*d*)

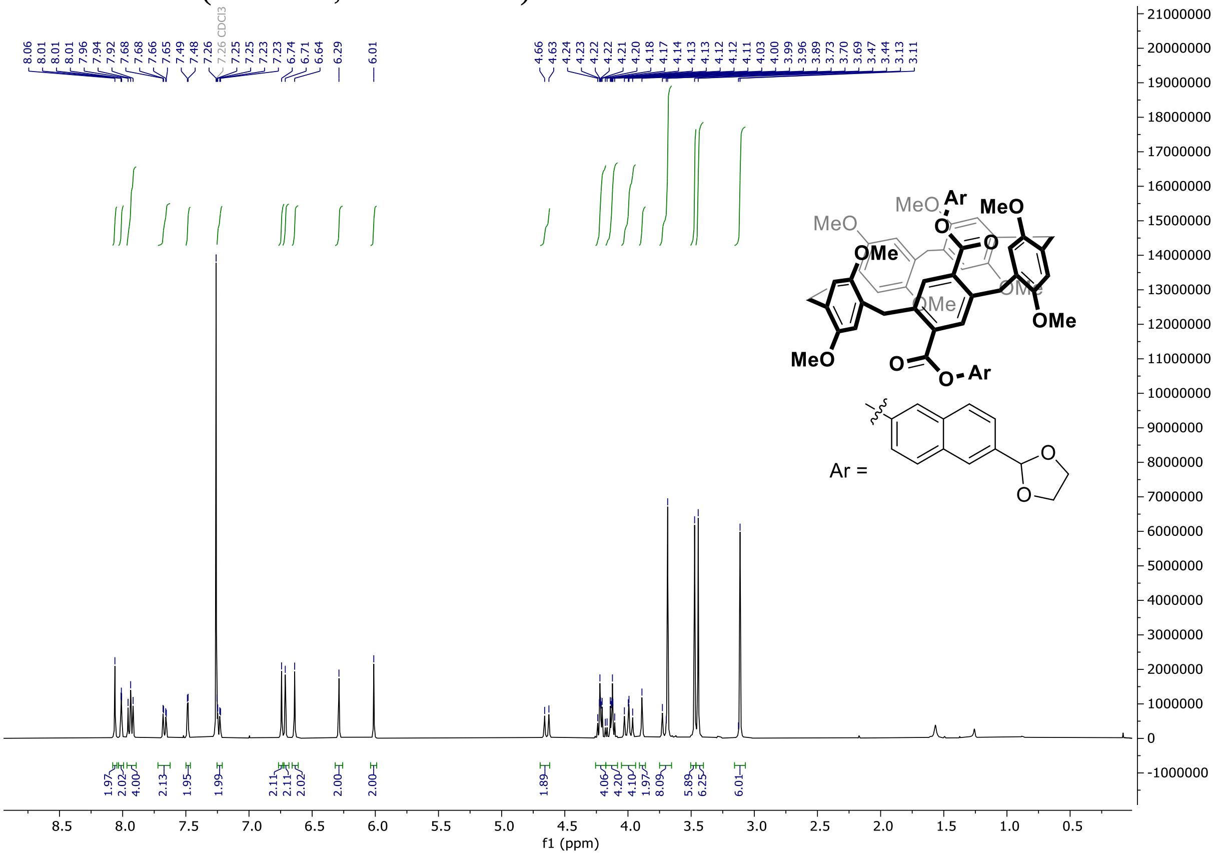

^{13}C{^{1}H} NMR of **3o** (101 MHz, chloroform-*d*)

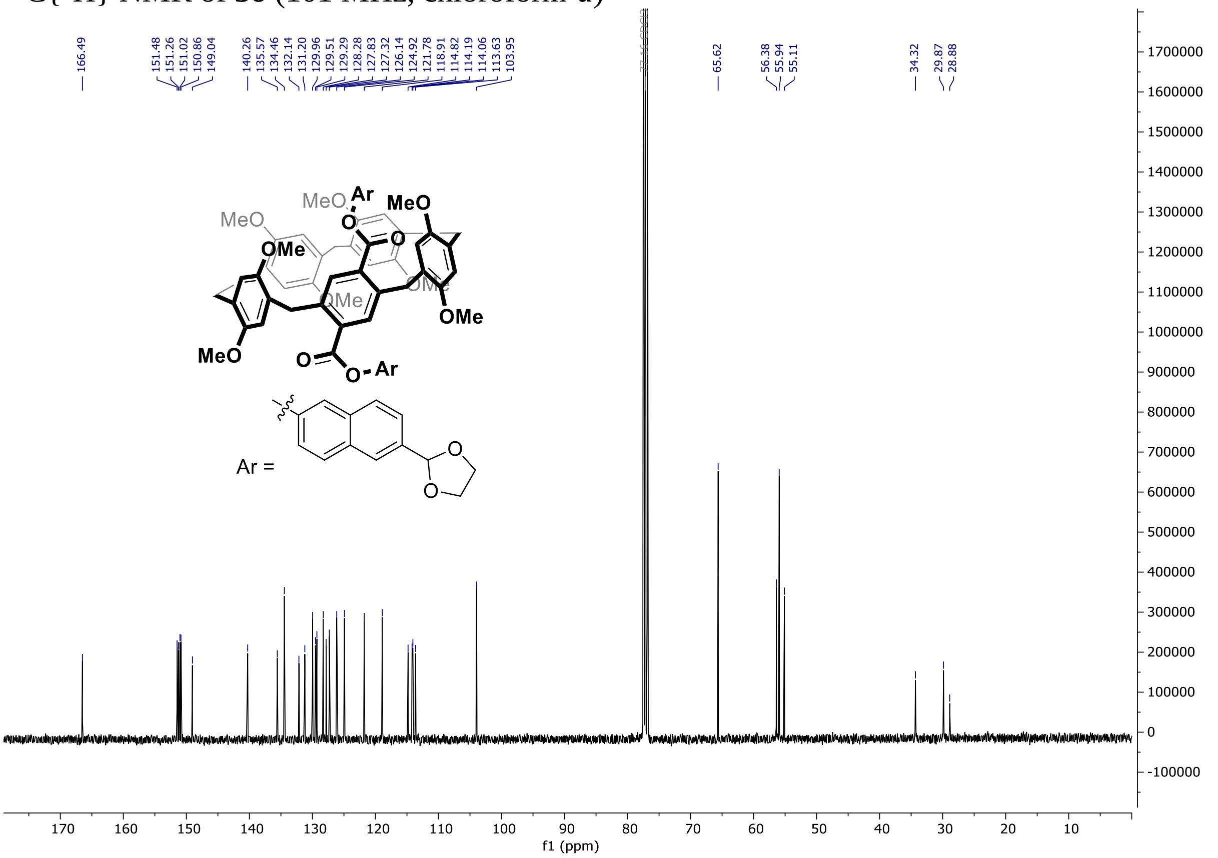

Bis(6-fluoronaphthalen-2-yl) (*pS*)-3^2,3^5,5^2,5^5,7^2,7^5,9^2,9^5-octamethoxy-1,3,5,7,9(1,4)-pentabenzenacyclodecaphane-1^2,1^5-dicarboxylate (3p)

^{1}H NMR of **3p** (400 MHz, chloroform-*d*)

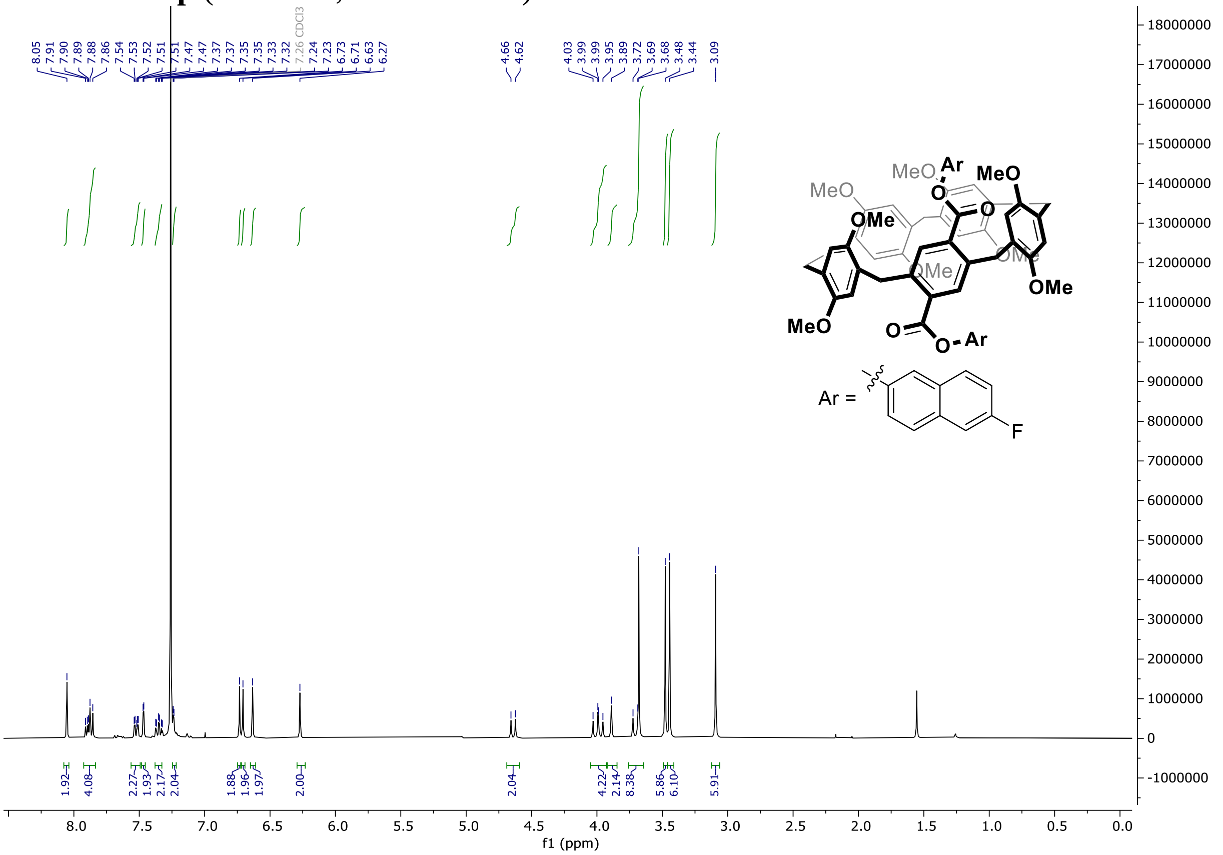

^{13}C{^{1}H} NMR of **3p** (101 MHz, chloroform-*d*)

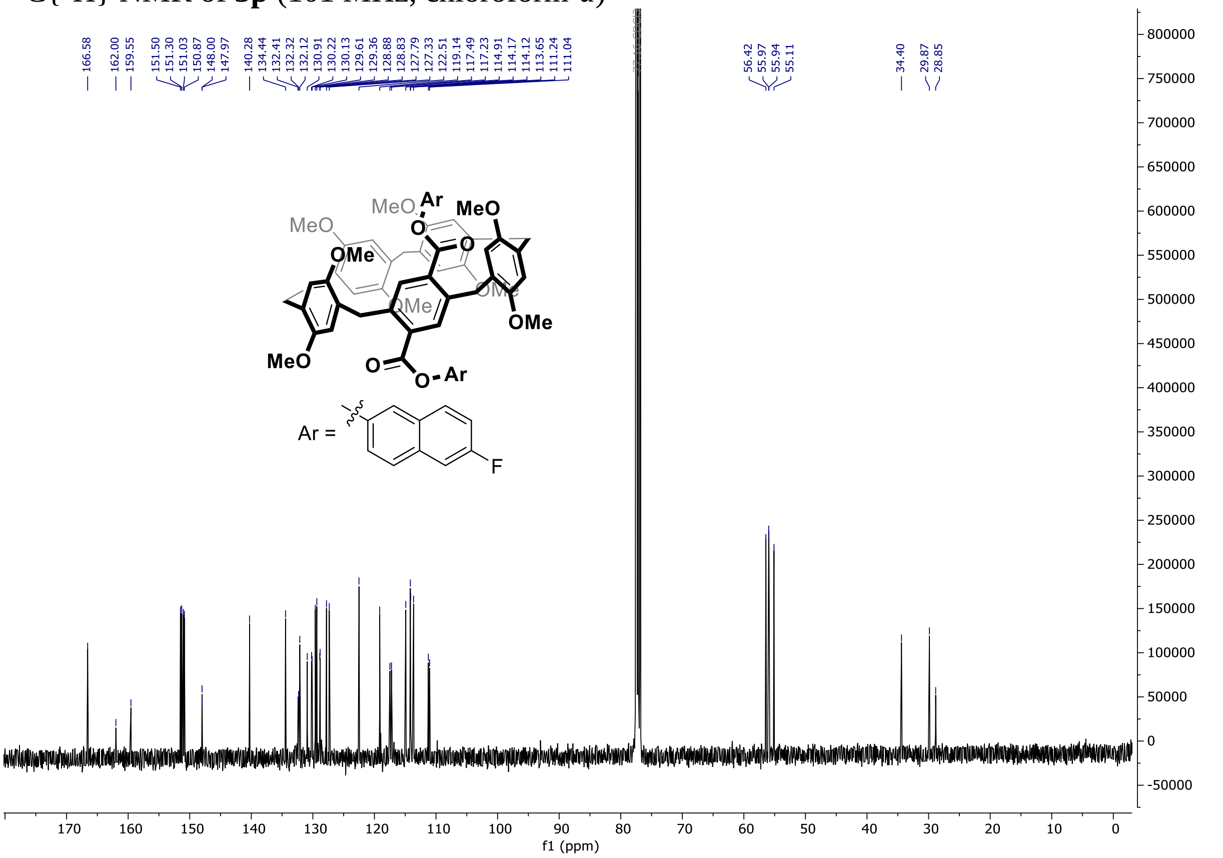

^{19}F NMR of **3p** (376 MHz, chloroform-*d*)

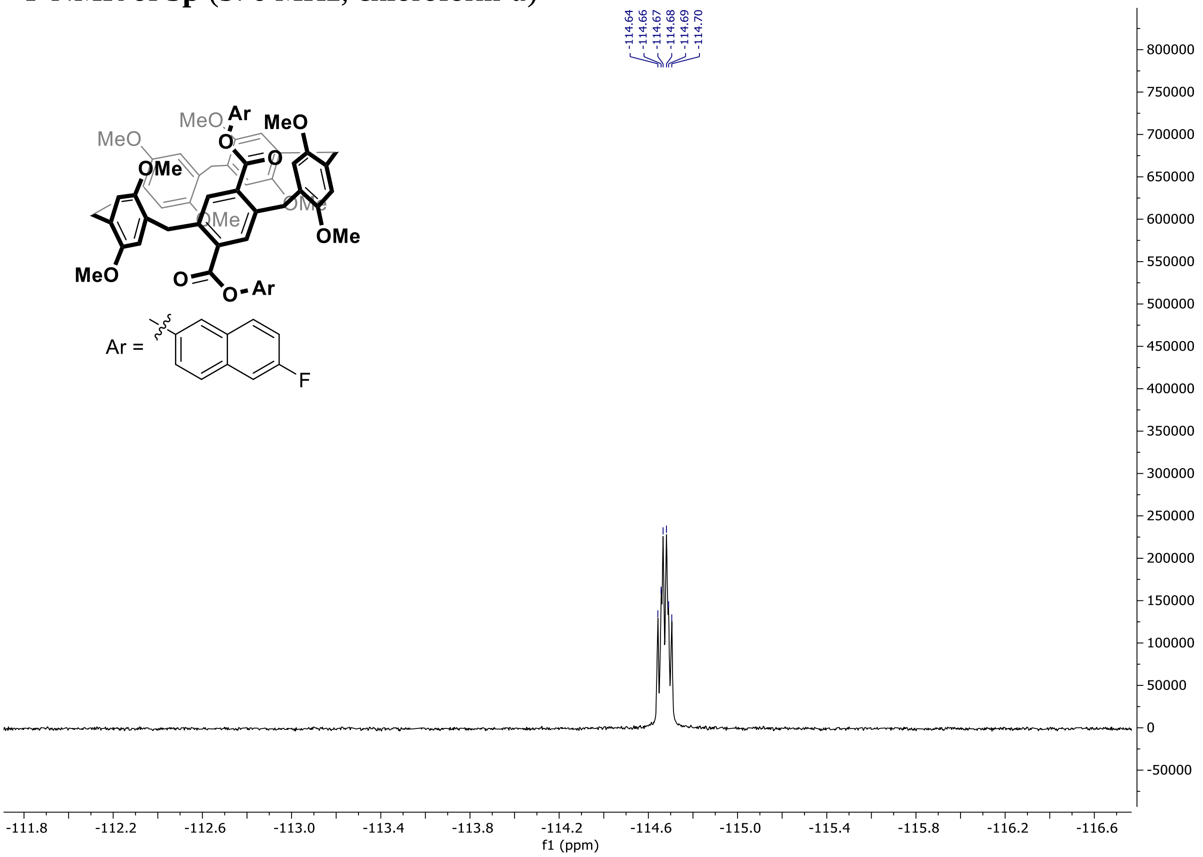

Bis(6-chloronaphthalen-2-yl) (*pS*)-3[2],3[5],5[2],5[5],7[2],7[5],9[2],9[5]-octamethoxy-1,3,5,7,9(1,4)-pentabenzenacyclodecaphane-1[2],1[5]-dicarboxylate (3q)

^{1}H NMR of **3q** (400 MHz, chloroform-*d*)

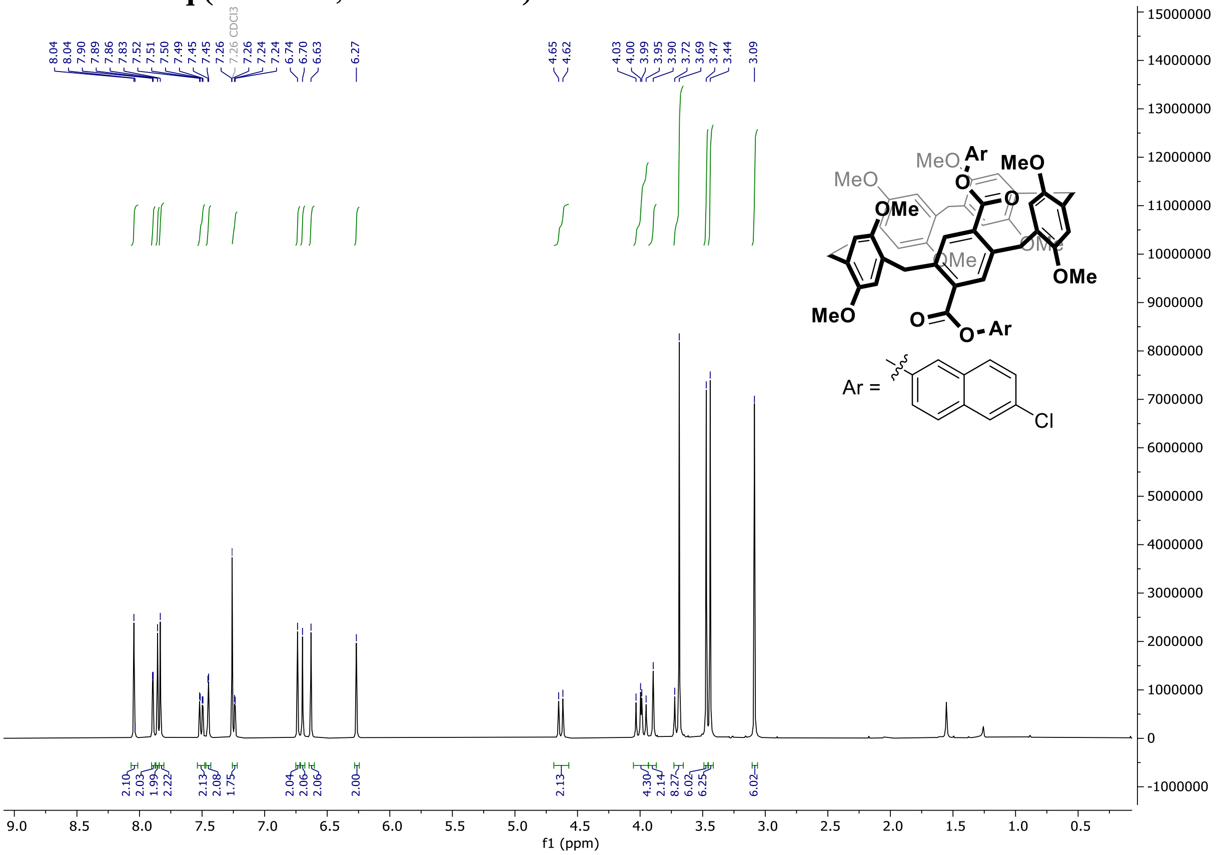

^{13}C{^{1}H} NMR of **3q** (101 MHz, chloroform-*d*)

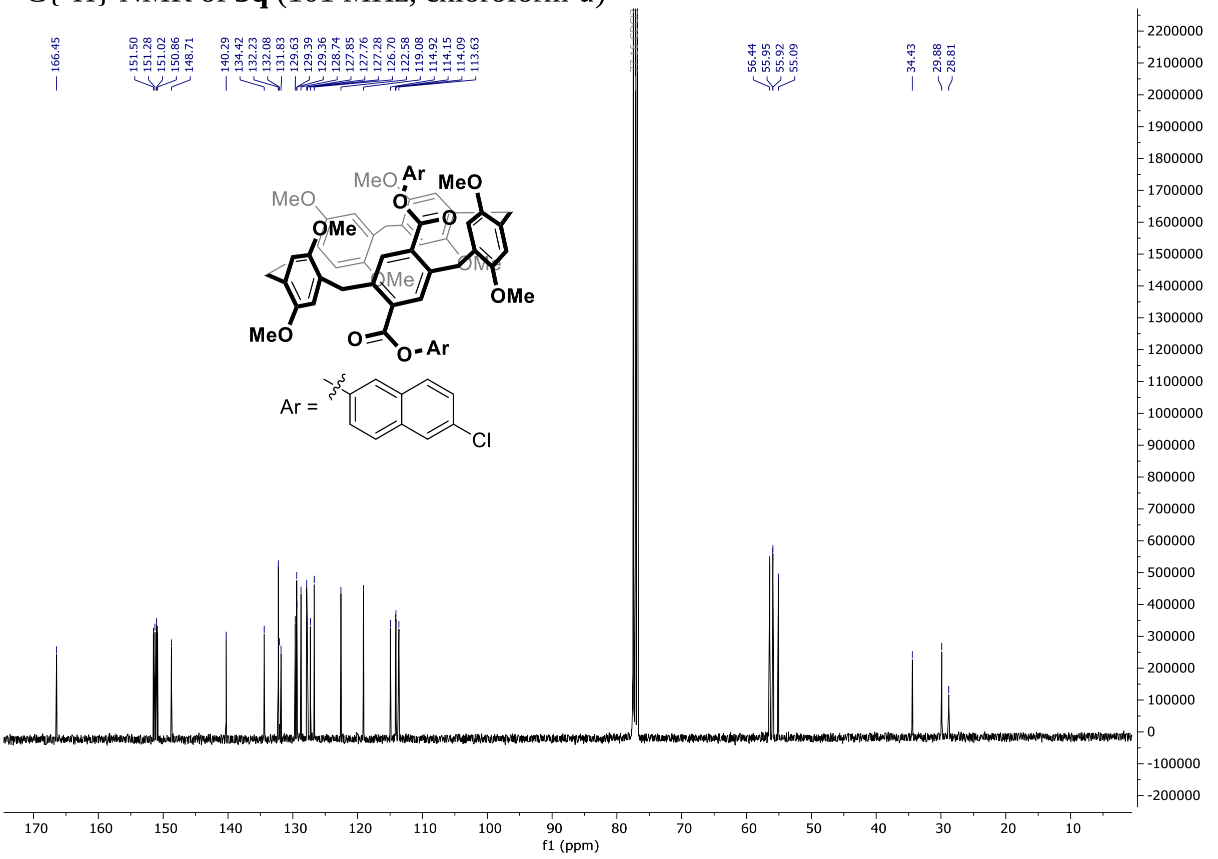

Bis(6-bromonaphthalen-2-yl) (*pS*)-3^2,3^5,5^2,5^5,7^2,7^5,9^2,9^5-octamethoxy-1,3,5,7,9(1,4)-pentabenzenacyclodecaphane-1^2,1^5-dicarboxylate (3r)

^{1}H NMR of **3r** (400 MHz, chloroform-*d*)

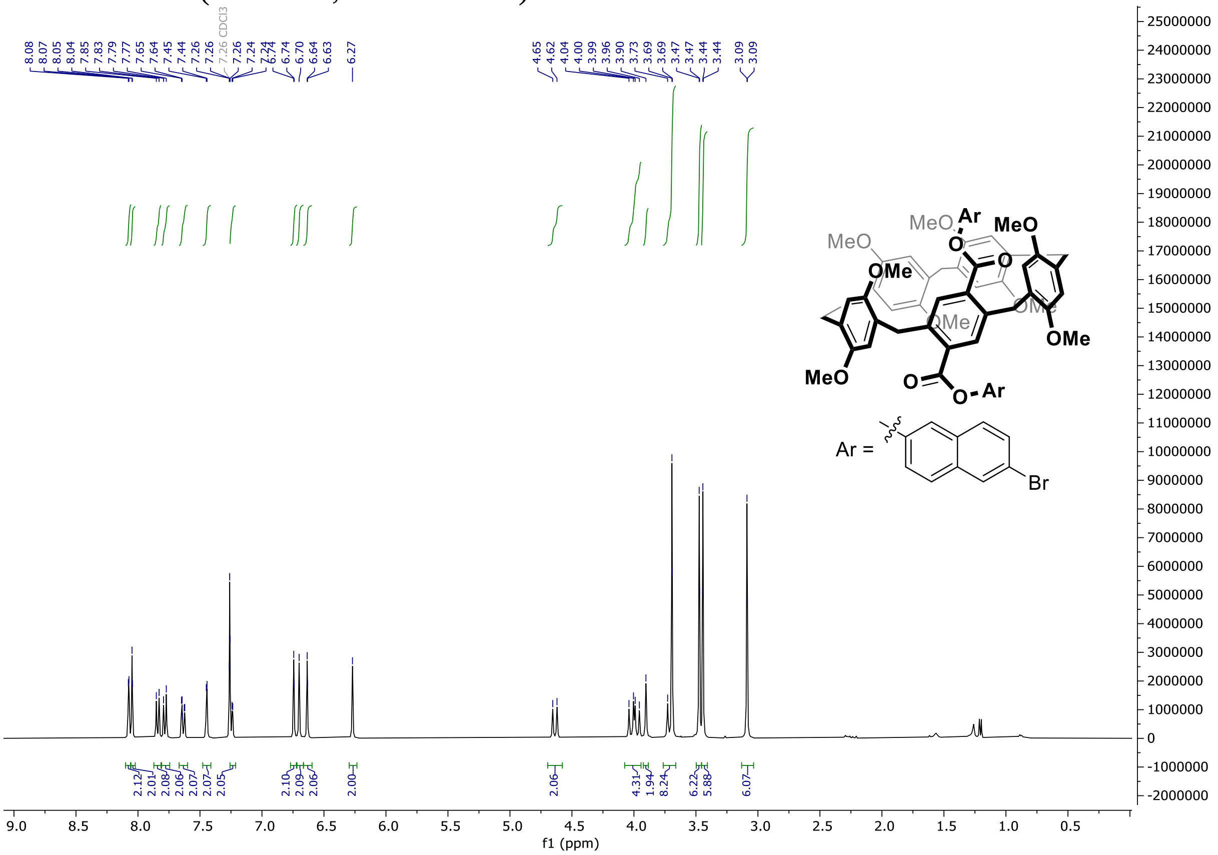

^{13}C{^{1}H} NMR of **3r** (101 MHz, chloroform-*d*)

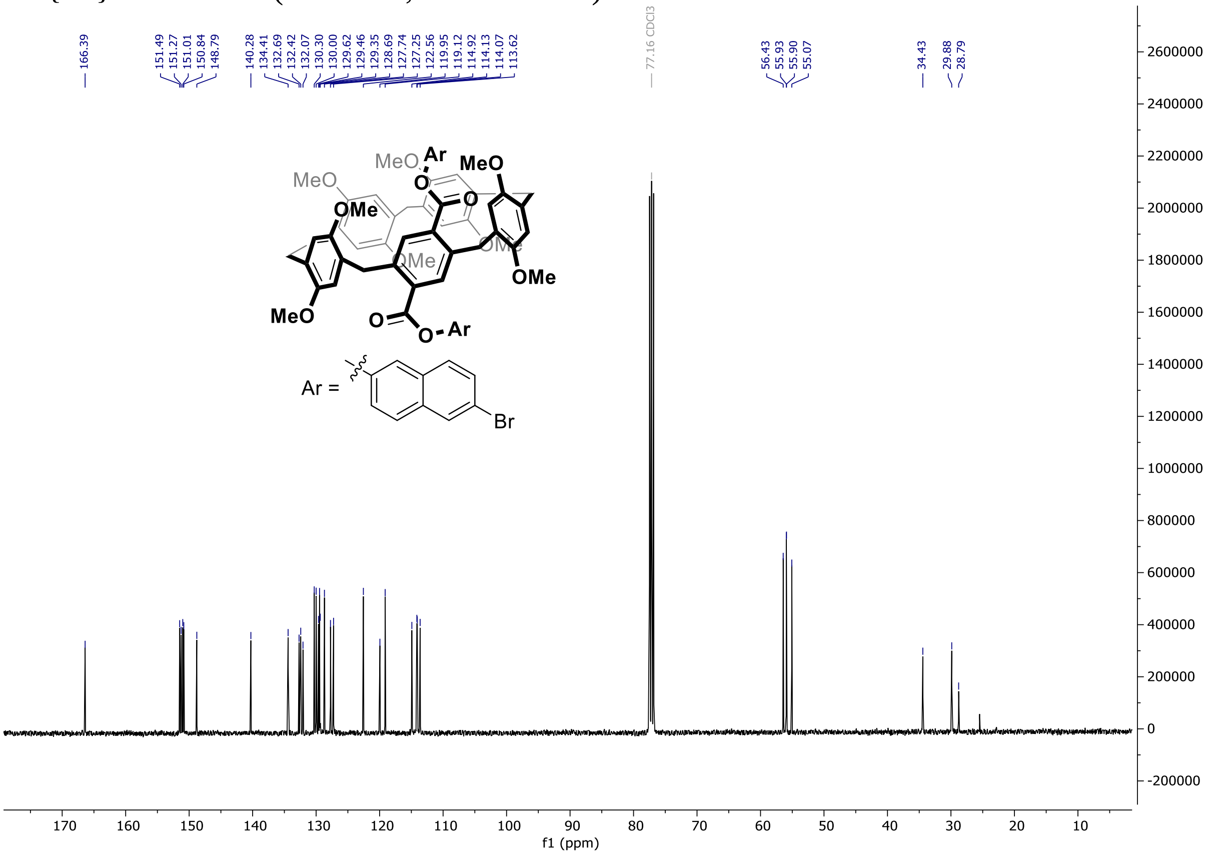

Bis(5,6,7,8-tetrahydronaphthalen-2-yl) (*pS*)-$3^2,3^5,5^2,5^5,7^2,7^5,9^2,9^5$-octamethoxy-1,3,5,7,9(1,4)-pentabenzenacyclodecaphane-$1^2,1^5$-dicarboxylate (3s)

^{1}H NMR of **3s** (400 MHz, chloroform-*d*)

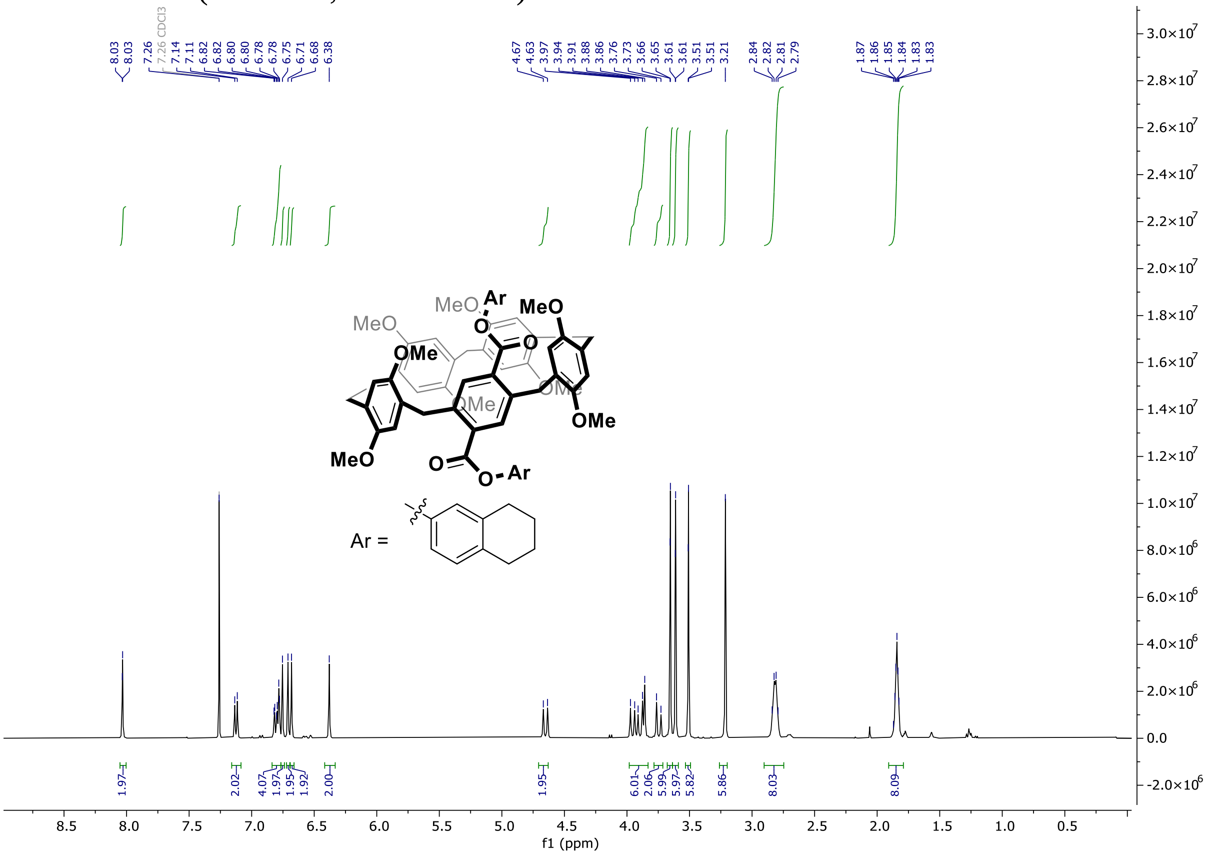

^{13}C{^{1}H} NMR of **3s** (101 MHz, chloroform-*d*)

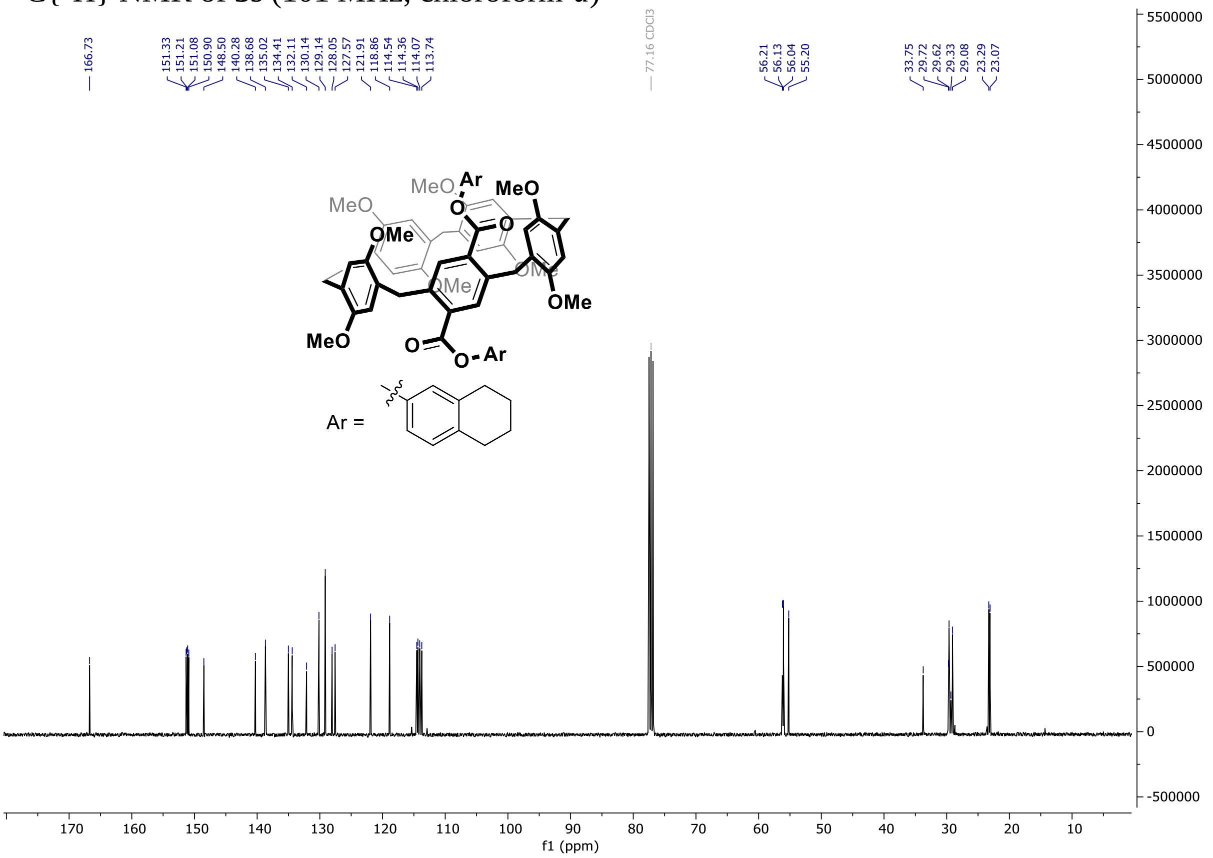

Di(naphthalen-1-yl) (*pS*)-3²,3⁵,5²,5⁵,7²,7⁵,9²,9⁵-octamethoxy-1,3,5,7,9(1,4)-pentabenzenacyclodecaphane-1²,1⁵-dicarboxylate (3t)

^{1}H NMR of **3t** (400 MHz, chloroform-*d*)

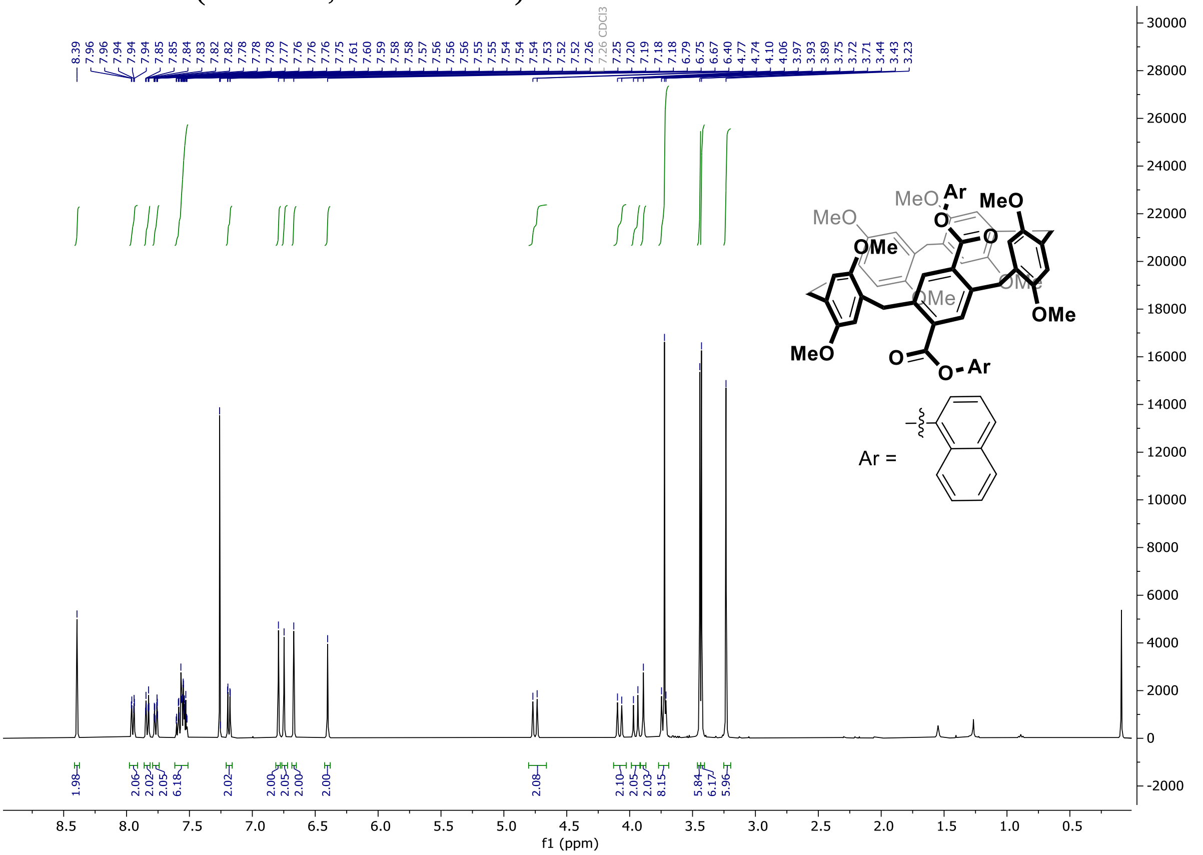

^{13}C{^{1}H} NMR of **3t** (101 MHz, chloroform-*d*)

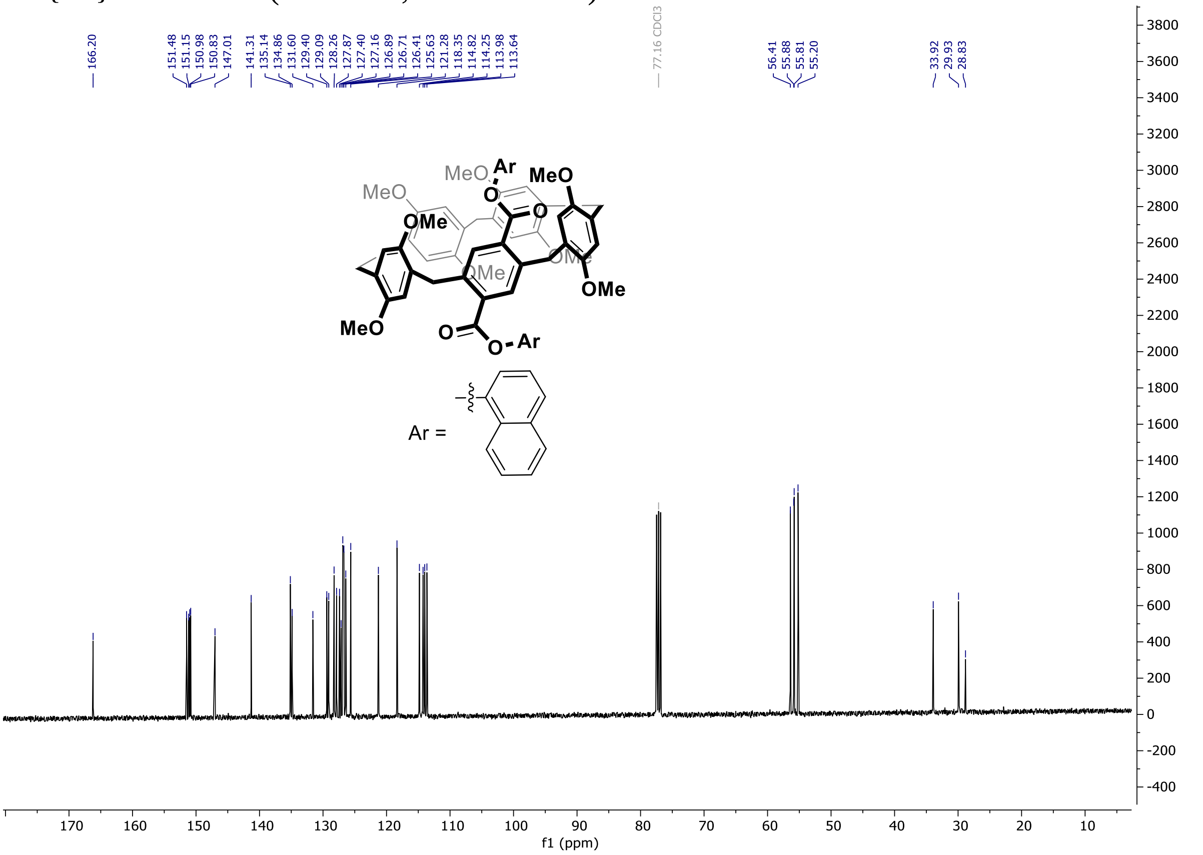

Bis(5,6,7,8-tetrahydronaphthalen-1-yl) (*pS*)-3^{2},3^{5},5^{2},5^{5},7^{2},7^{5},9^{2},9^{5}-octamethoxy-1,3,5,7,9(1,4)-pentabenzenacyclodecaphane-1^{2},1^{5}-dicarboxylate (3u)

^{1}H NMR of **3u** (400 MHz, chloroform-*d*)

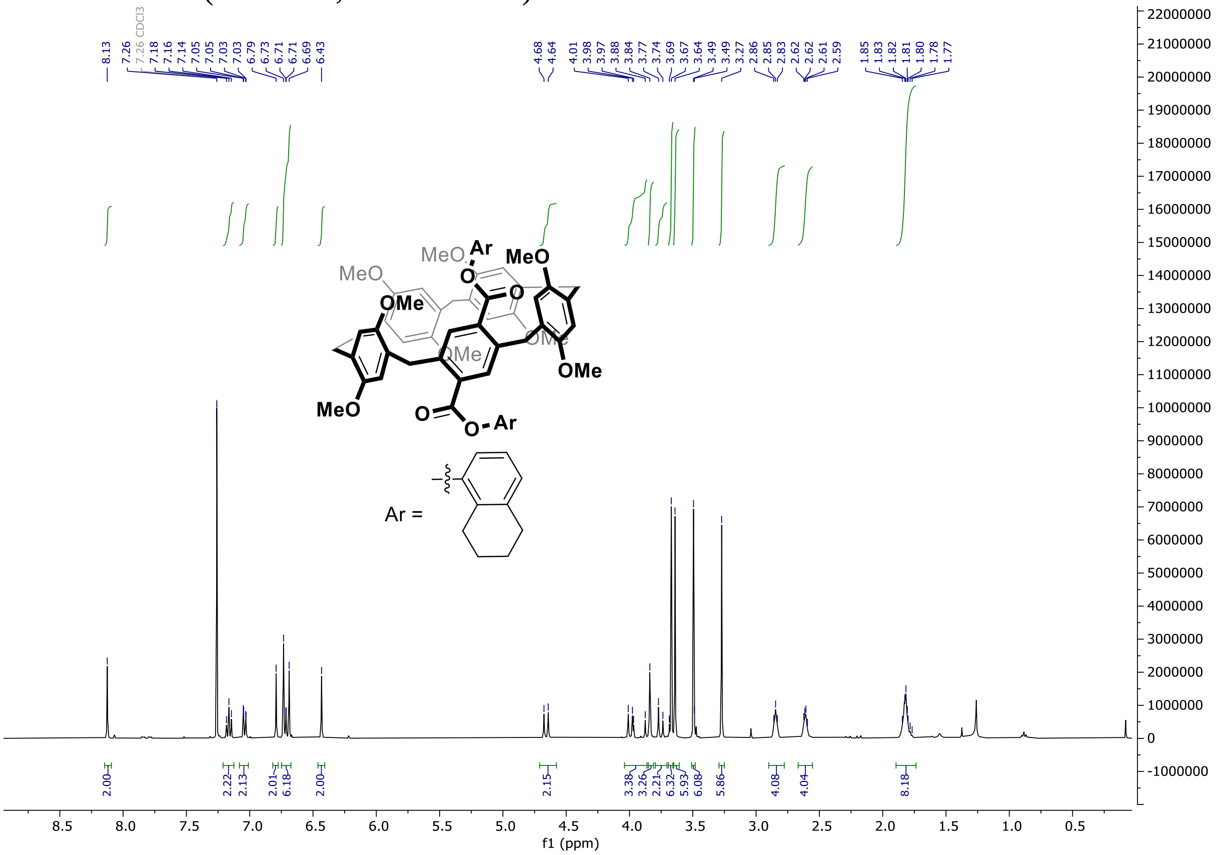

^{13}C{^{1}H} NMR of **3u** (101 MHz, chloroform-*d*)

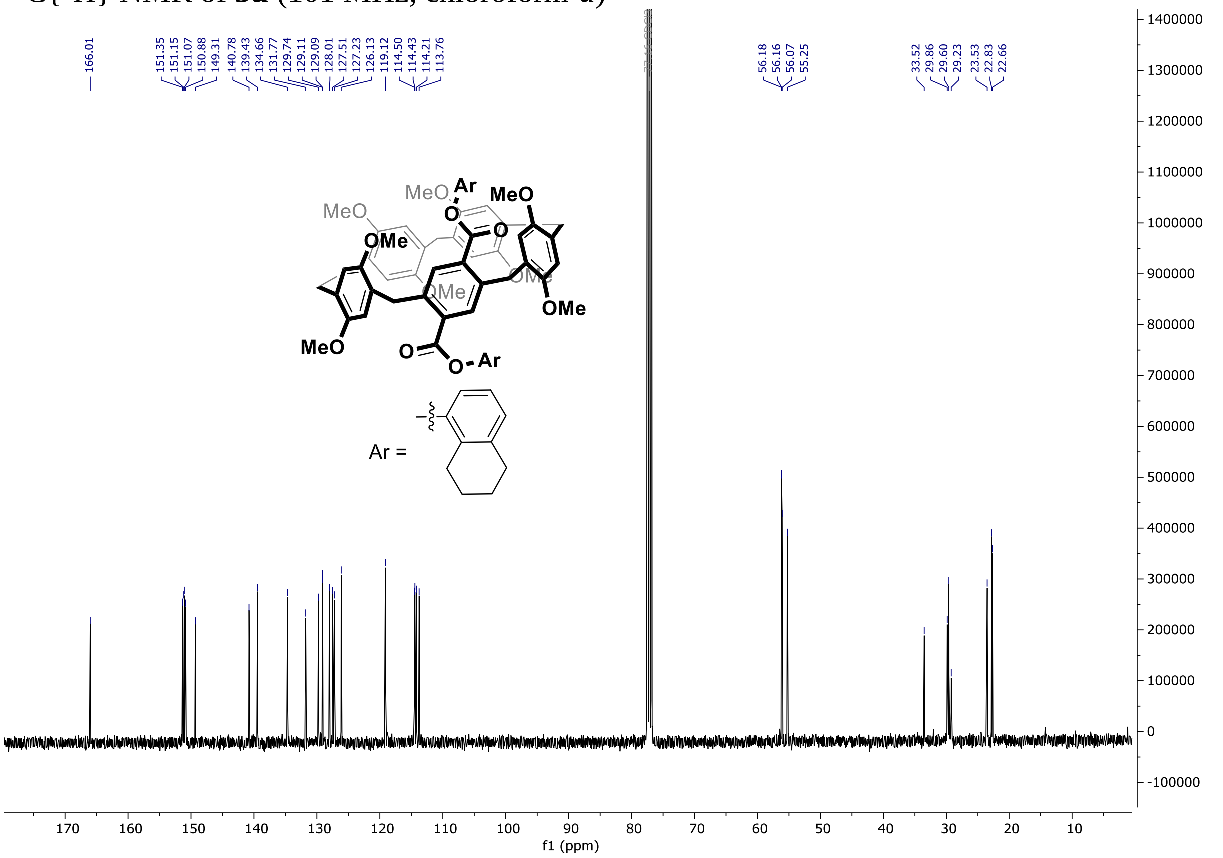

Diphenyl 3^2,3^5,5^2,5^5,7^2,7^5,9^2,9^5-octamethoxy-1,3,5,7,9(1,4)-pentabenzenacyclodecaphane-1^2,1^5-dicarboxylate (4a)

^{1}H NMR of **4a** (400 MHz, chloroform-*d*)

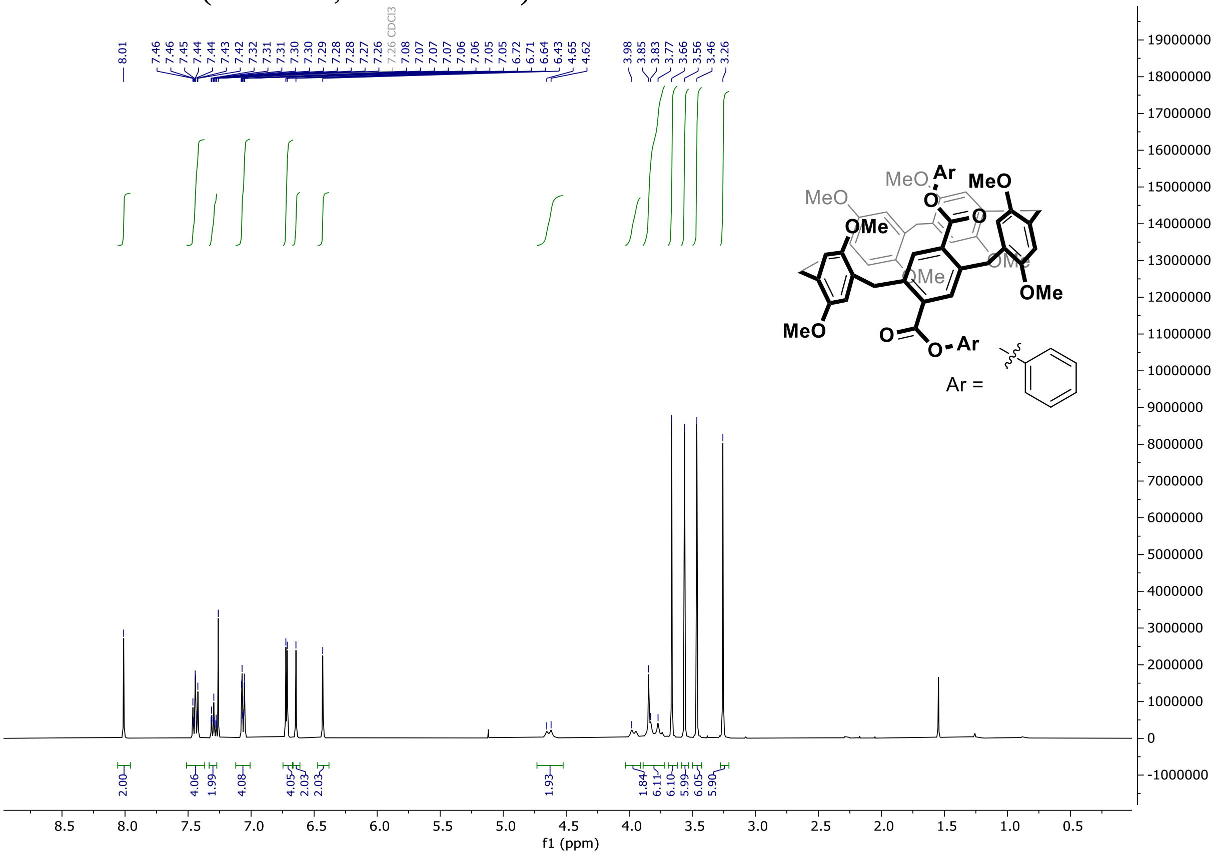

^{13}C{^{1}H} NMR of **4a** (101 MHz, chloroform-*d*)

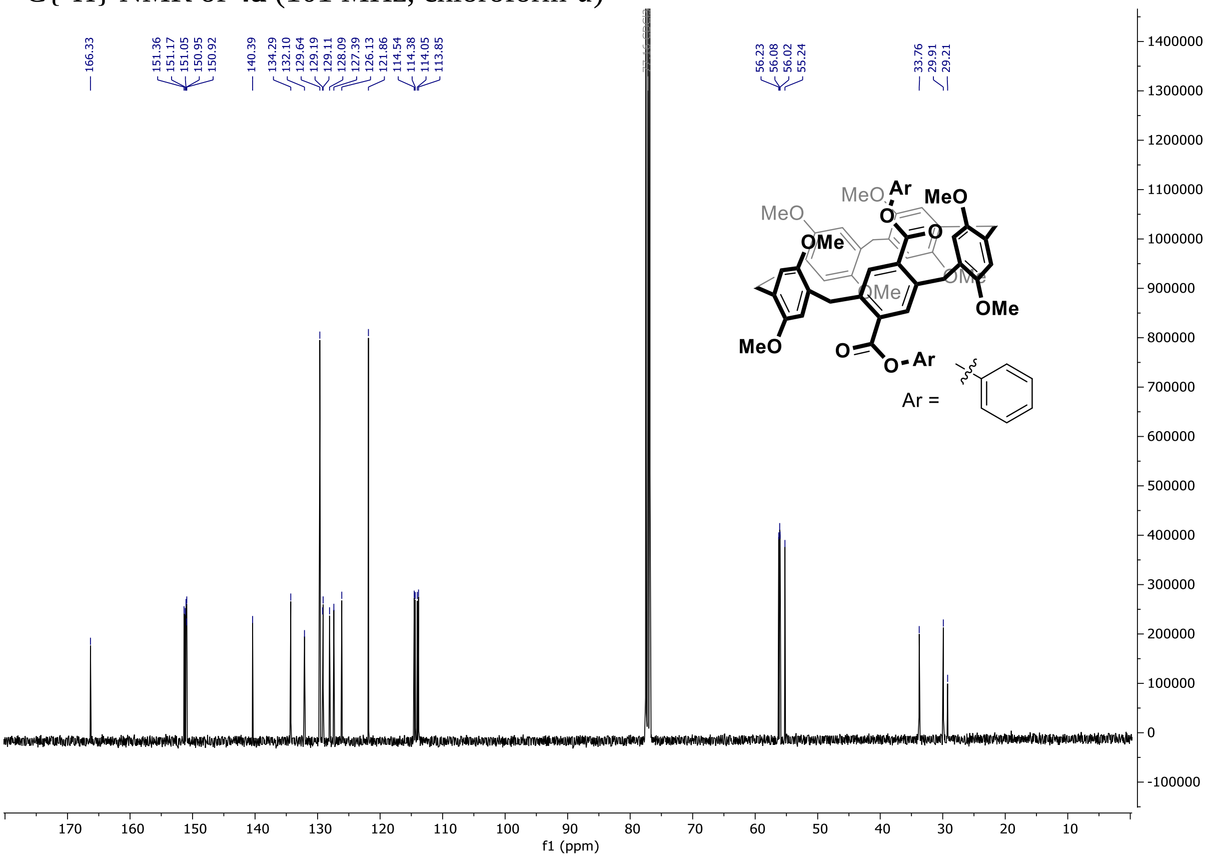

Bis(4-bromophenyl) $3^2,3^5,5^2,5^5,7^2,7^5,9^2,9^5$-octamethoxy-1,3,5,7,9(1,4)-pentabenzenacyclodecaphane-$1^2,1^5$-dicarboxylate (4b)

^{1}H NMR of **4b** (400 MHz, chloroform-*d*)

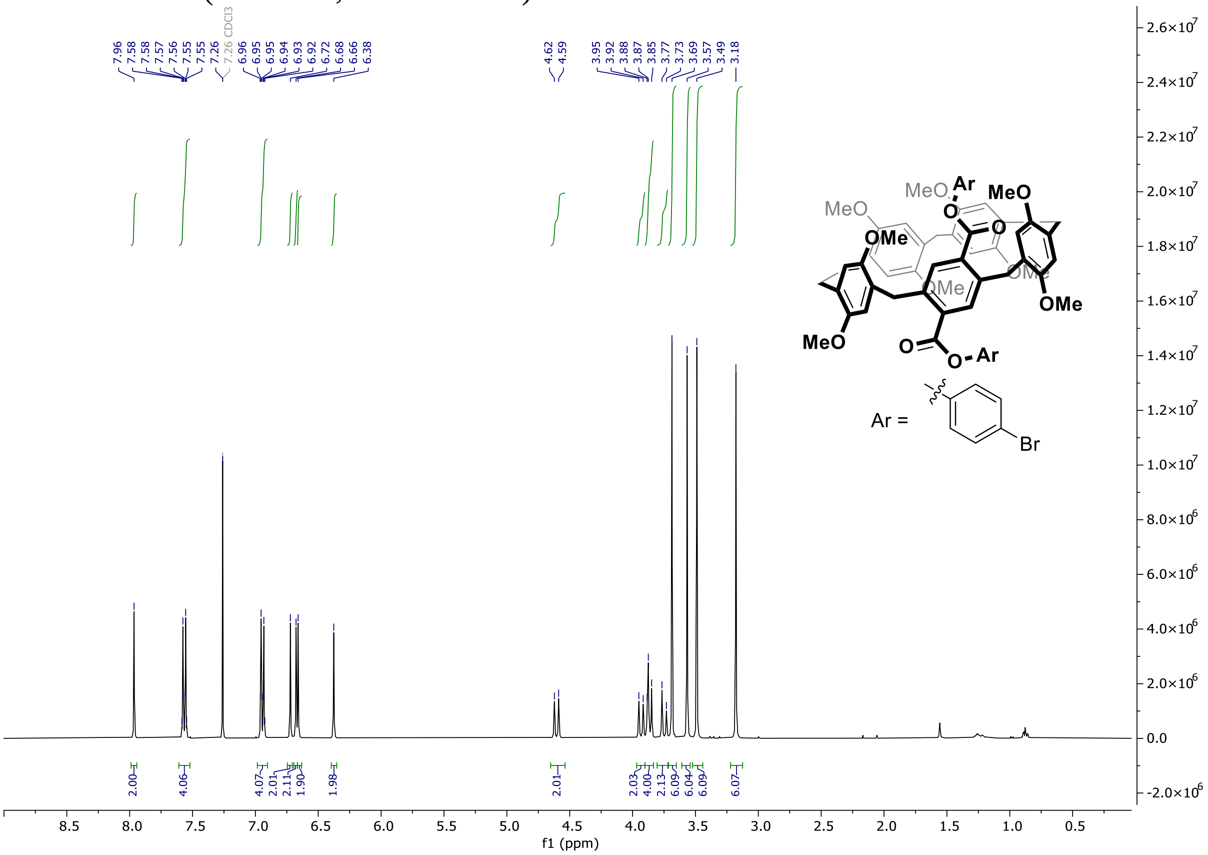

^{13}C{^{1}H} NMR of **4b** (101 MHz, chloroform-*d*)

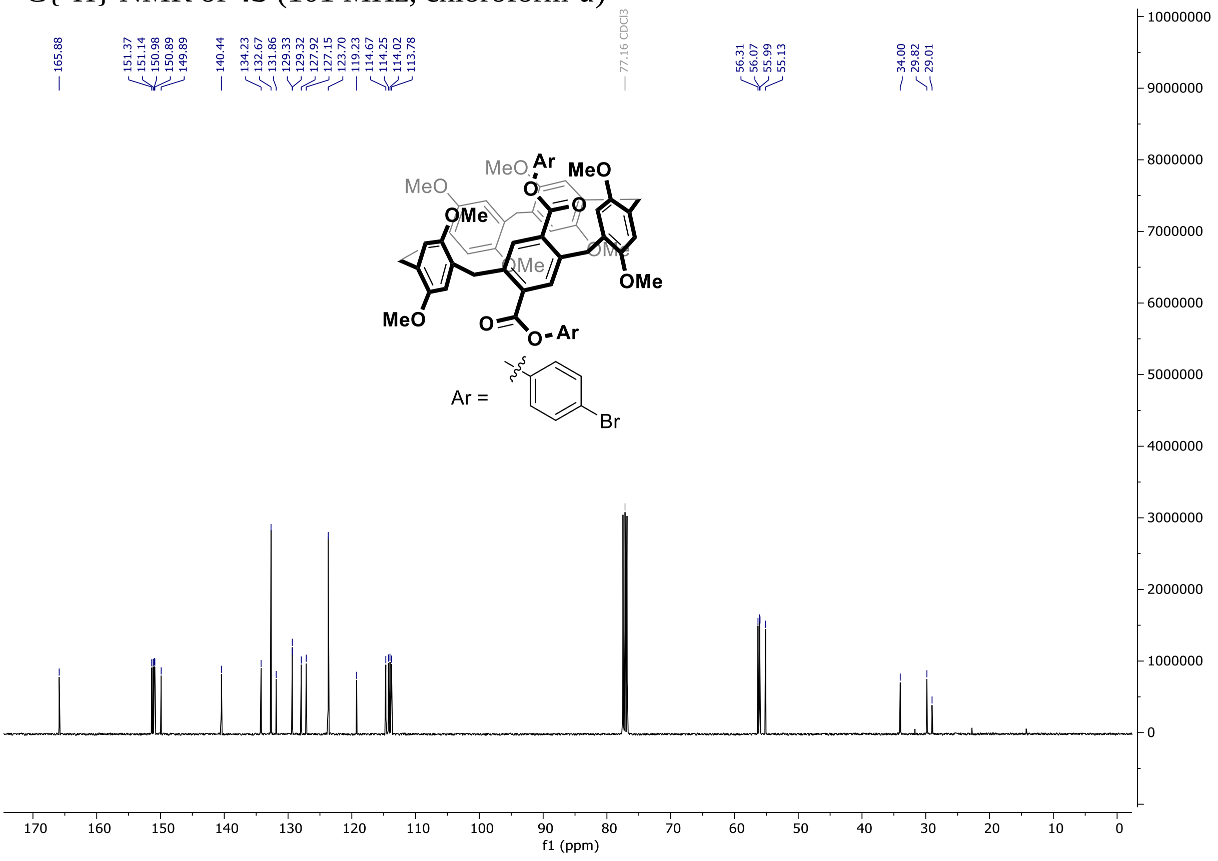

Di-*p*-tolyl 3^2,3^5,5^2,5^5,7^2,7^5,9^2,9^5-octamethoxy-1,3,5,7,9(1,4)-pentabenzenacyclodecaphane-1^2,1^5-dicarboxylate (4c)

^{1}H NMR of **4c** (400 MHz, chloroform-*d*)

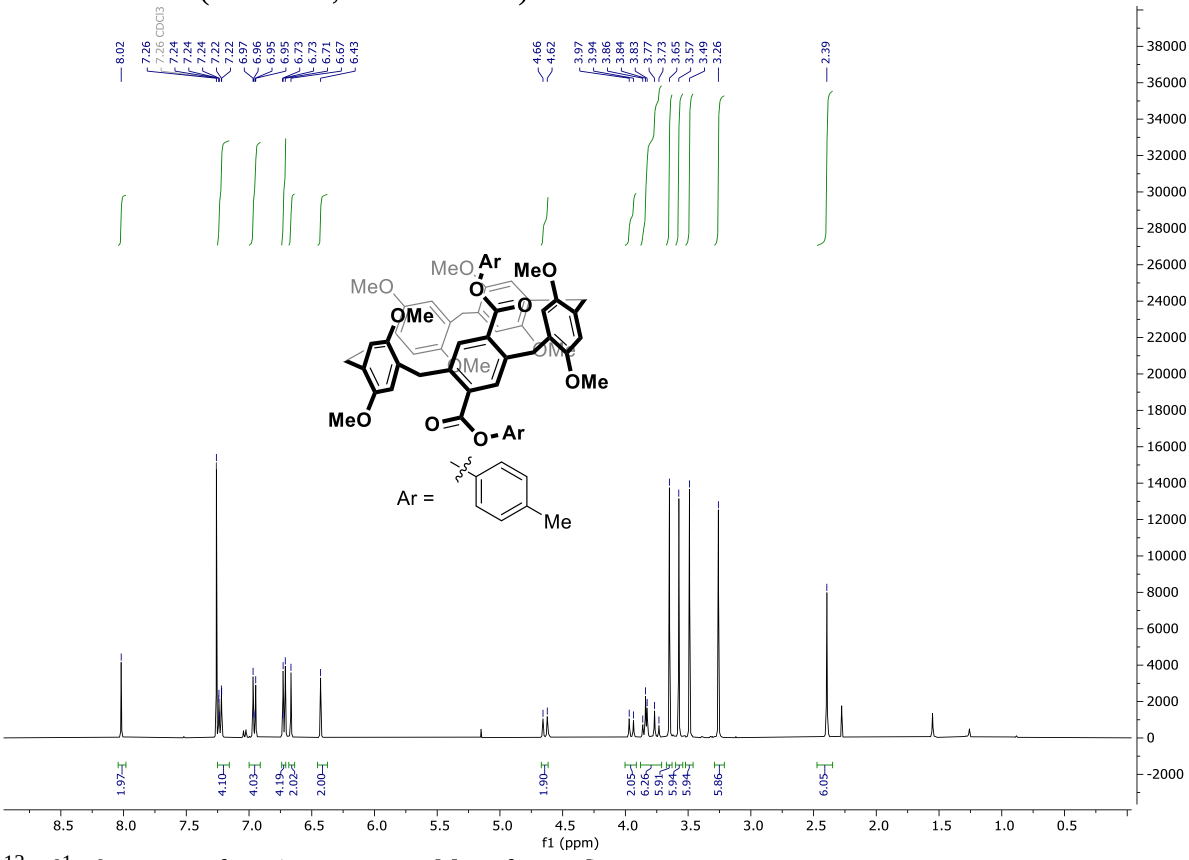

^{13}C{^{1}H} NMR of **4c** (101 MHz, chloroform-*d*)

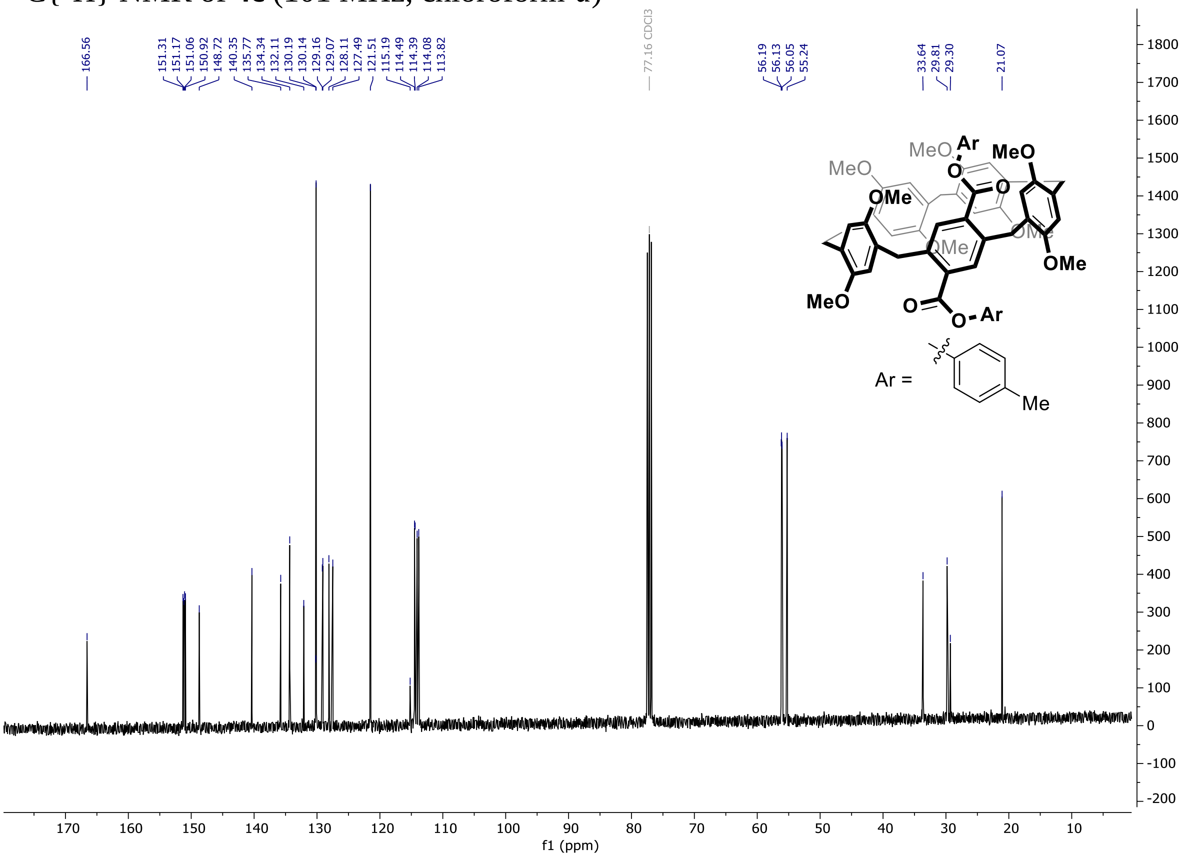

Bis(4-(*tert*-butyl)phenyl) (*pS*)-3²,3⁵,5²,5⁵,7²,7⁵,9²,9⁵-octamethoxy-1,3,5,7,9(1,4)-pentabenzenacyclodecaphane-1²,1⁵-dicarboxylate (4d)

^{1}H NMR of **4d** (400 MHz, chloroform-*d*)

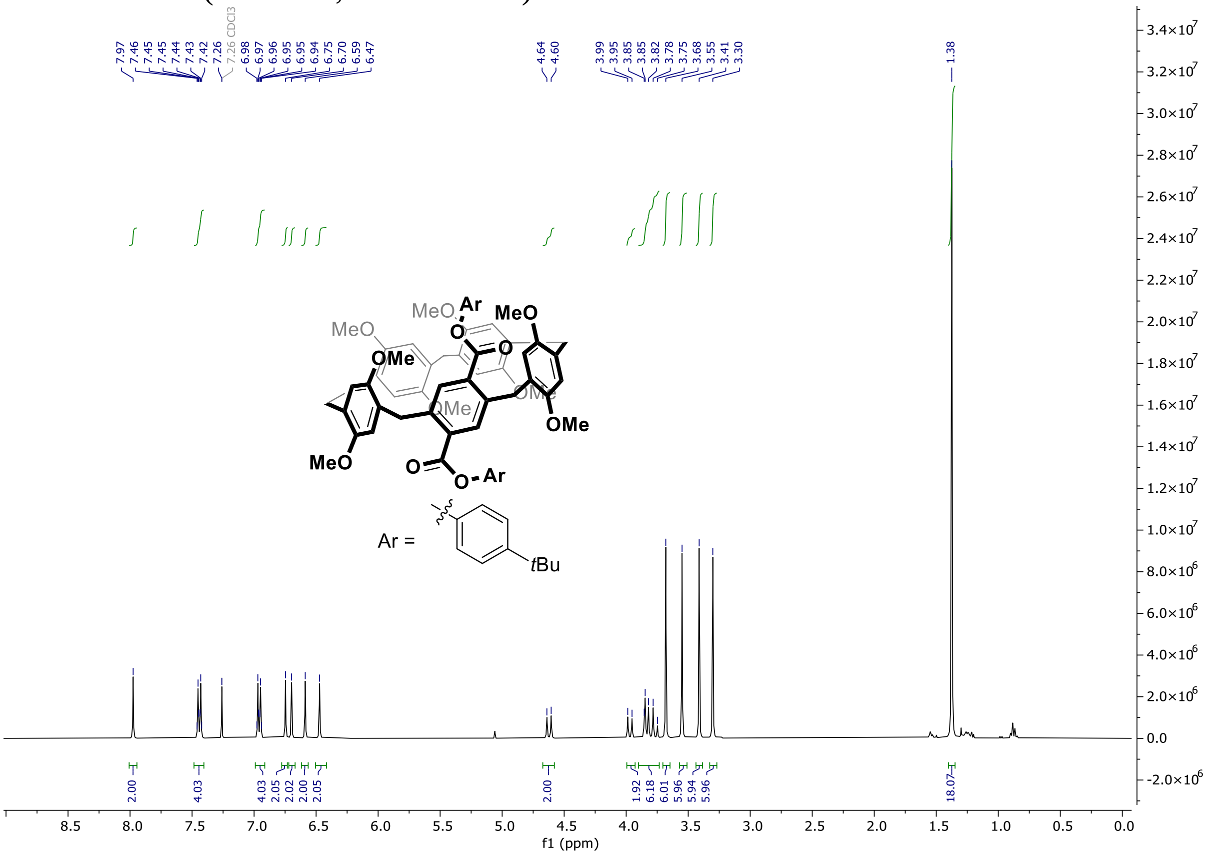

^{13}C{^{1}H} NMR of **4d** (101 MHz, chloroform-*d*)

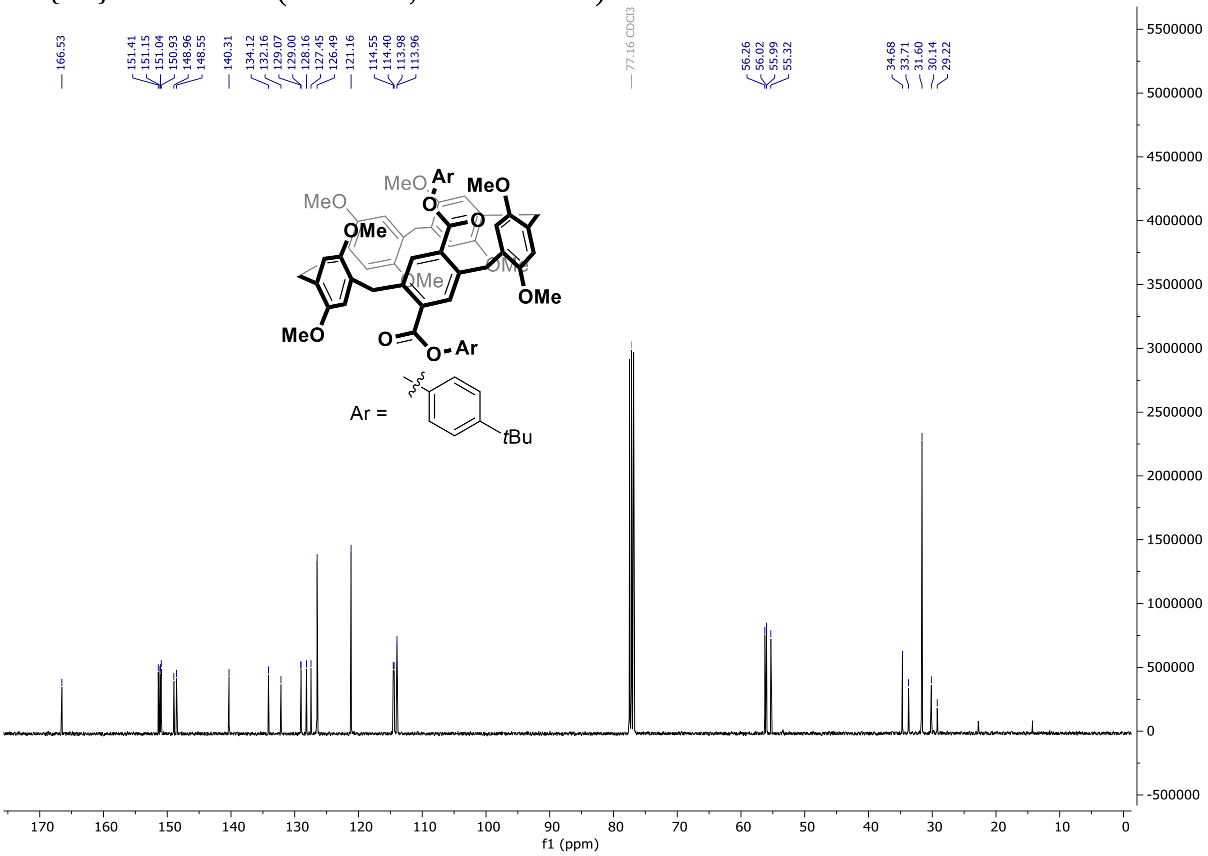

Di([1,1'-biphenyl]-4-yl) (*pS*)-3[2],3[5],5[2],5[5],7[2],7[5],9[2],9[5]-octamethoxy-1,3,5,7,9(1,4)-pentabenzenacyclodecaphane-1[2],1[5]-dicarboxylate (4e)

^{1}H NMR of **4e** (400 MHz, chloroform-*d*)

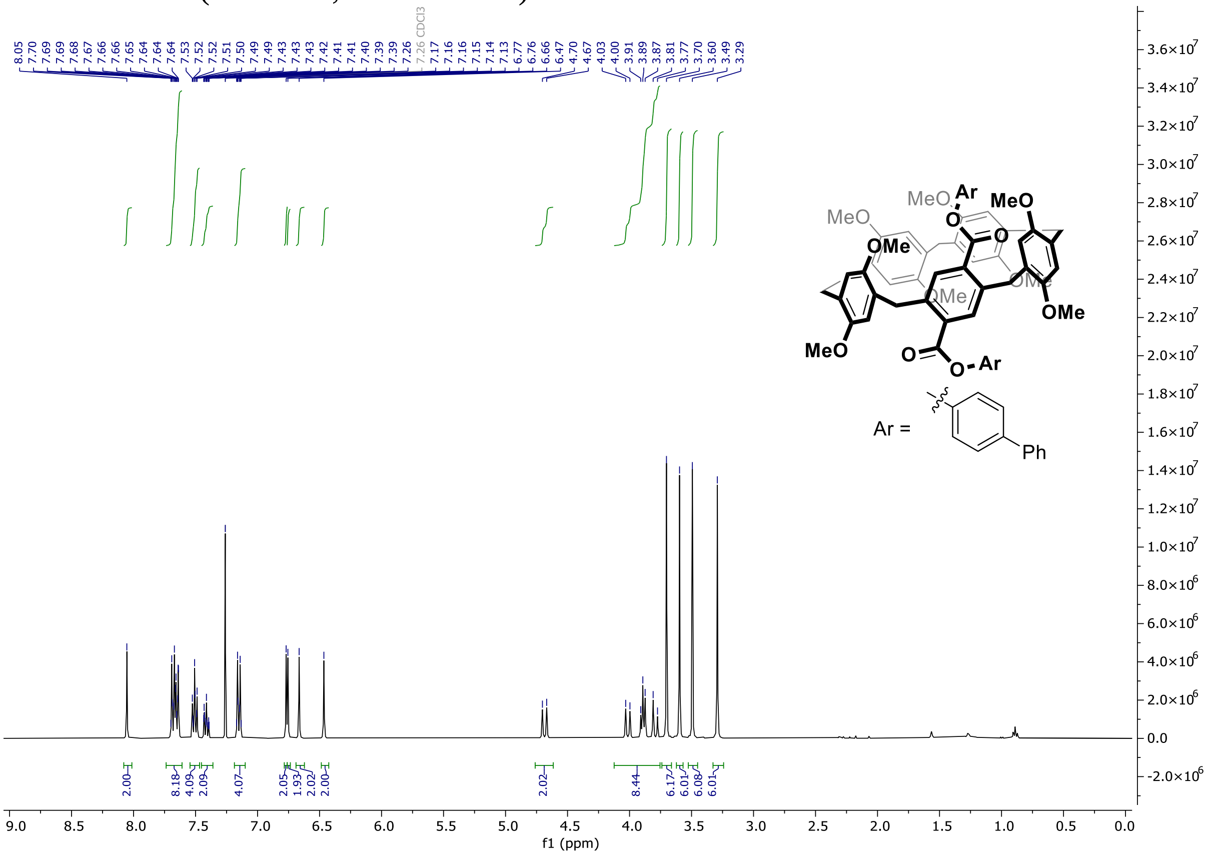

^{13}C{^{1}H} NMR of **4e** (101 MHz, chloroform-*d*)

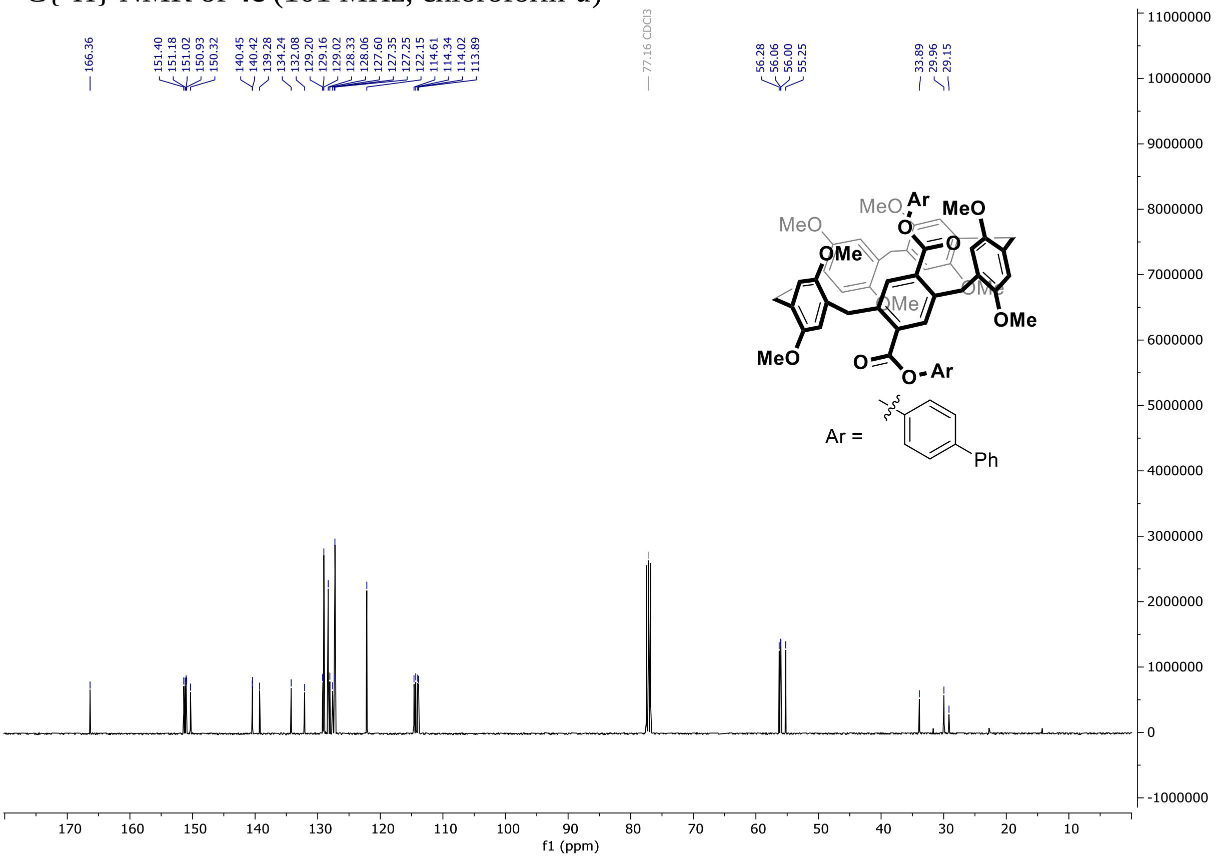

Dimethyl $3^2,3^5,5^2,5^5,7^2,7^5,9^2,9^5$-octamethoxy-1,3,5,7,9(1,4)-pentabenzenacyclodecaphane-$1^2,1^5$-dicarboxylate (4f)

^{1}H NMR of **4f** (400 MHz, chloroform-d)

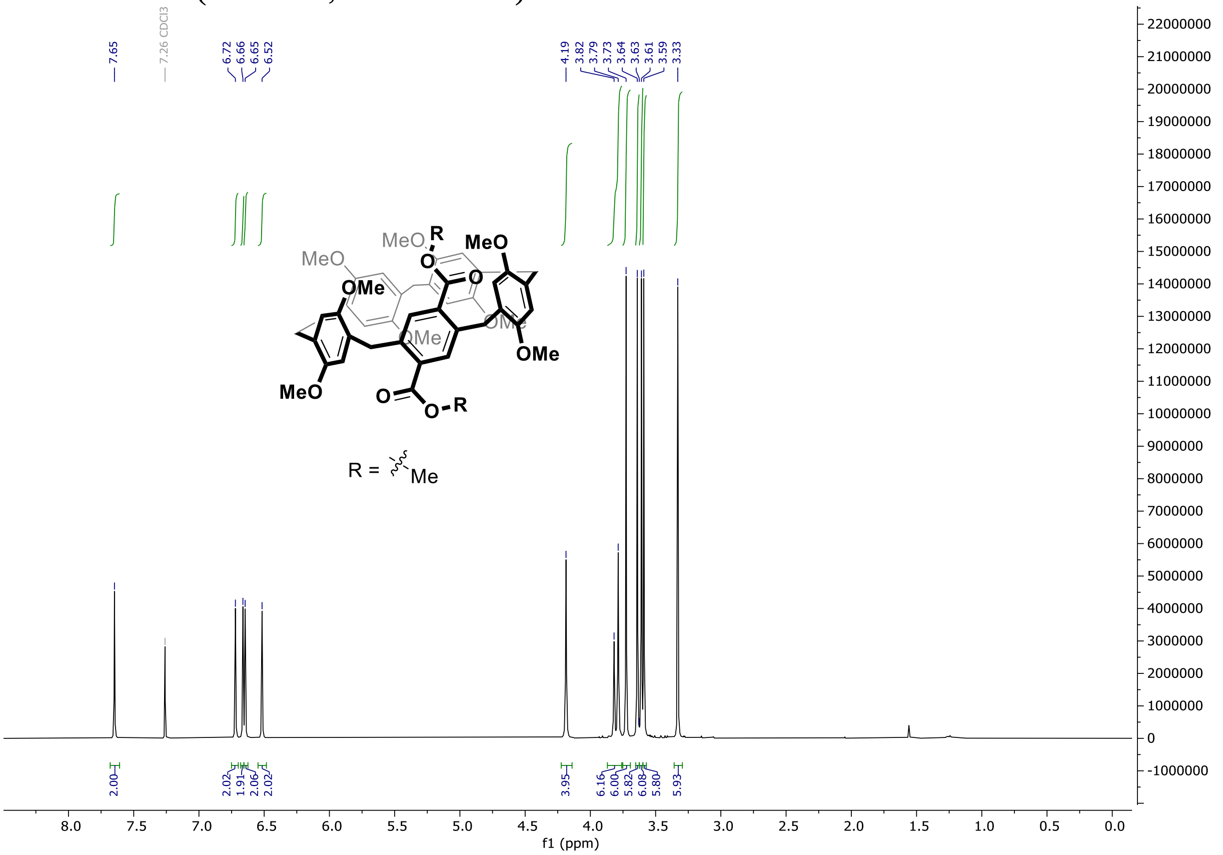

^{13}C{^{1}H} NMR of **4f** (101 MHz, chloroform-d)

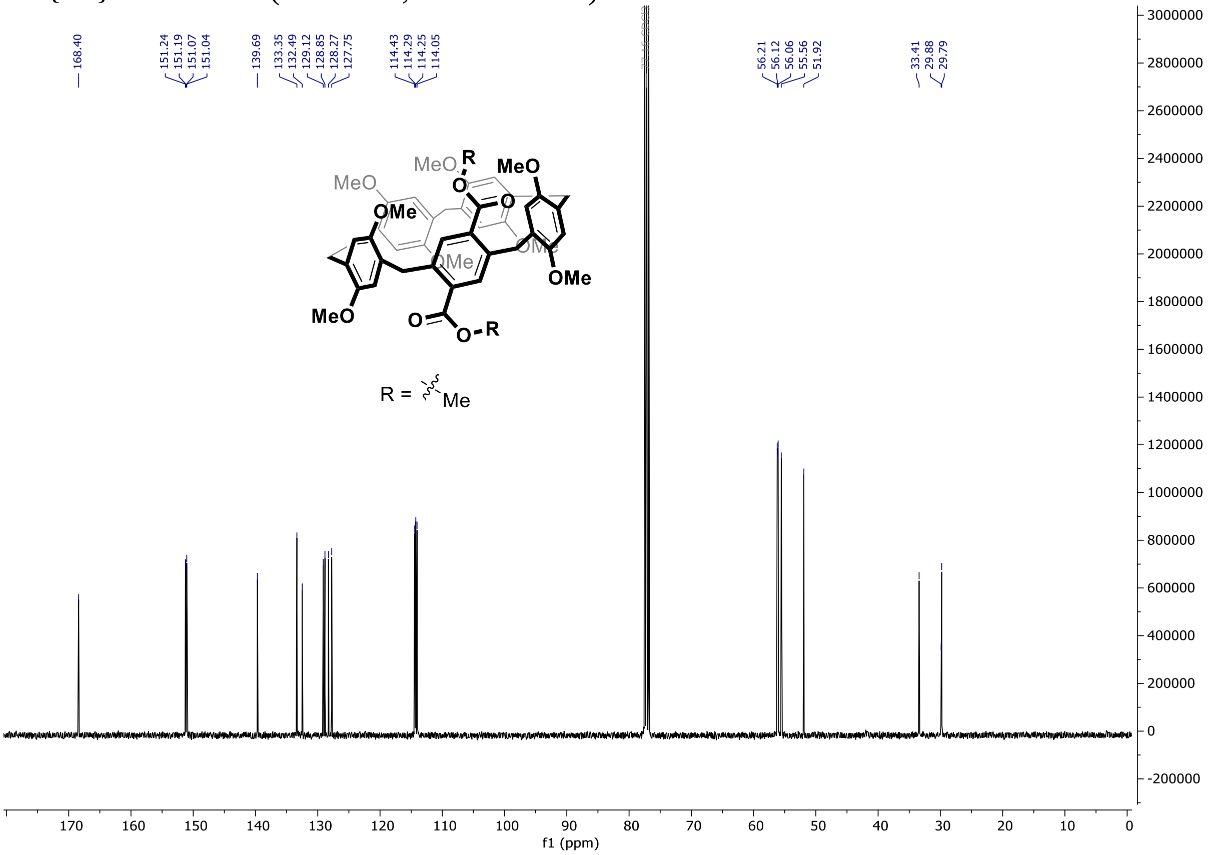

Didodecyl 3²,3⁵,5²,5⁵,7²,7⁵,9²,9⁵-octamethoxy-1,3,5,7,9(1,4)-pentabenzenacyclodecaphane-1²,1⁵-dicarboxylate (4i)

^{1}H NMR of **4i** (400 MHz, chloroform-*d*)

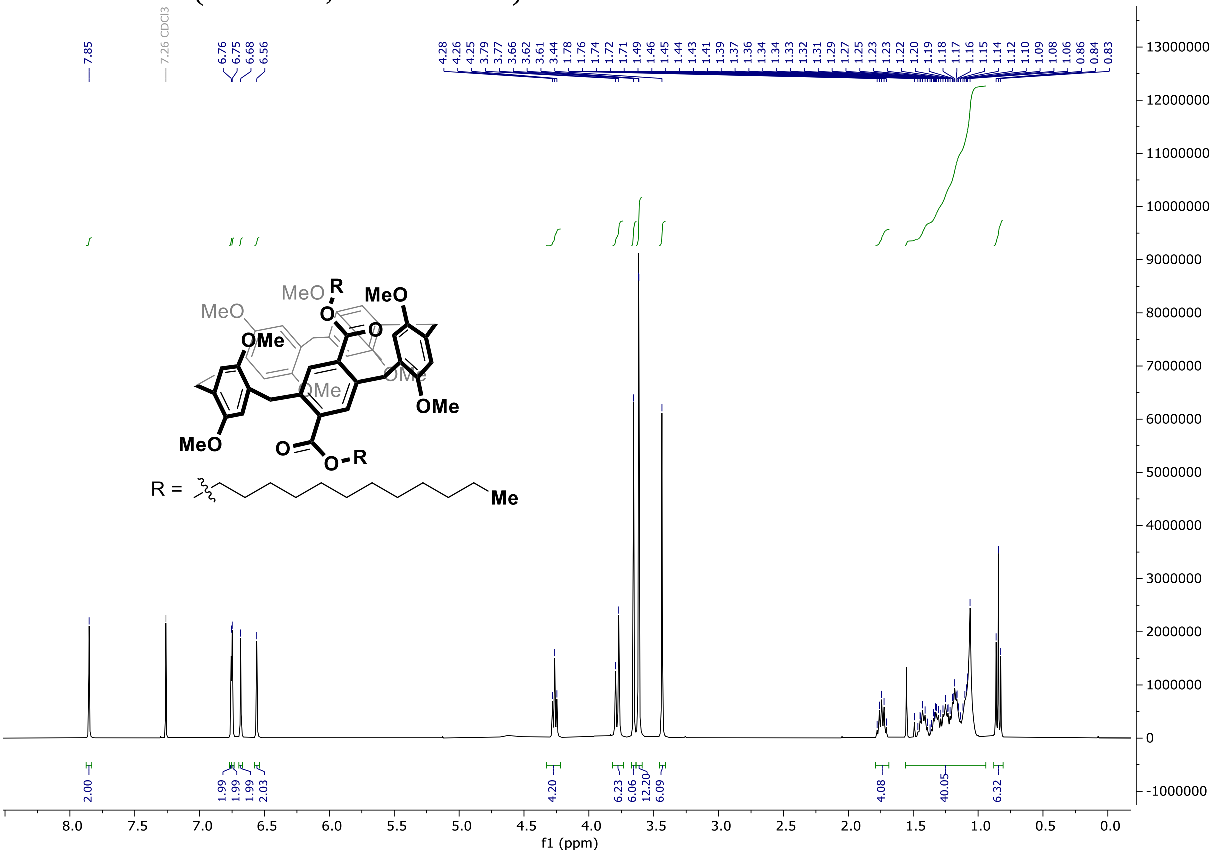

^{13}C{^{1}H} NMR of **4i** (101 MHz, chloroform-*d*)

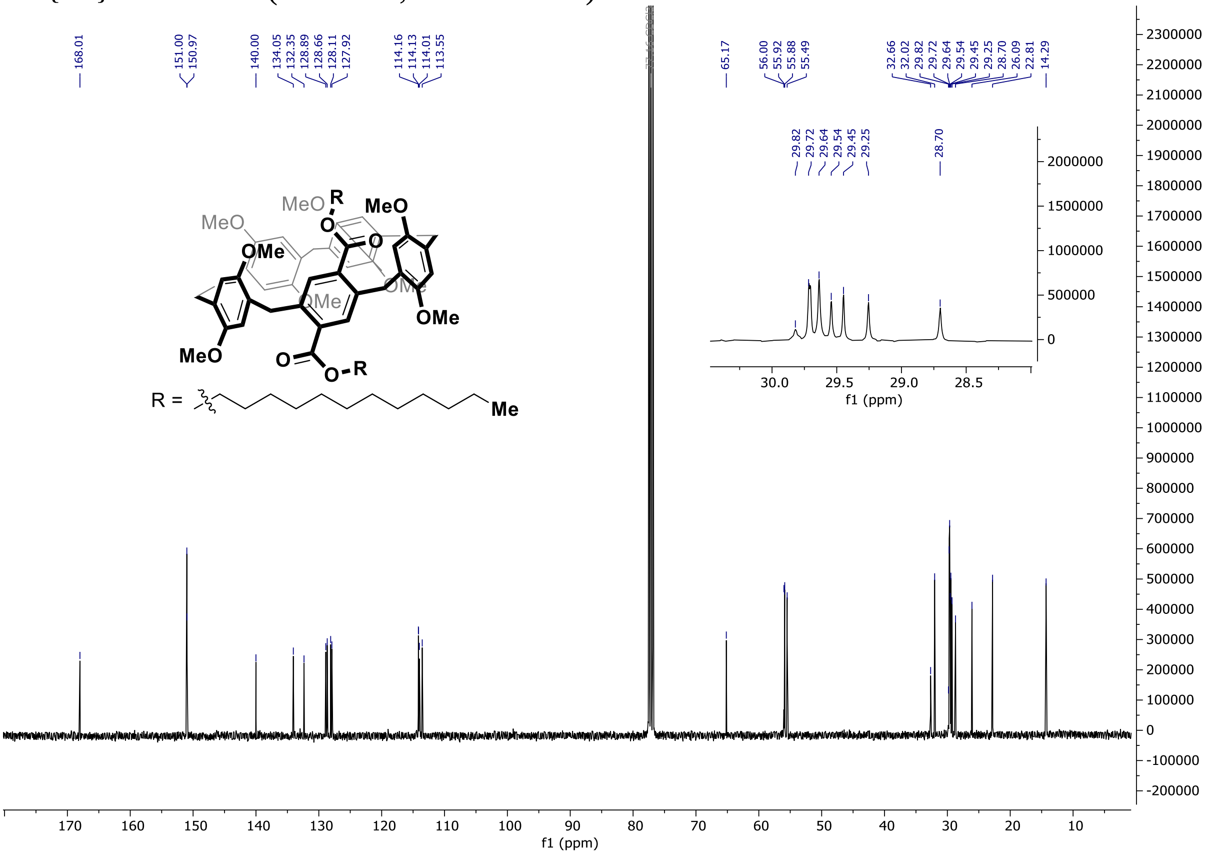

Bis(3-phenylpropyl) 3[2],3[5],5[2],5[5],7[2],7[5],9[2],9[5]-octamethoxy-1,3,5,7,9(1,4)-pentabenzenacyclodecaphane-1[2],1[5]-dicarboxylate (4j)

^{1}H NMR of **4j** (400 MHz, chloroform-*d*)

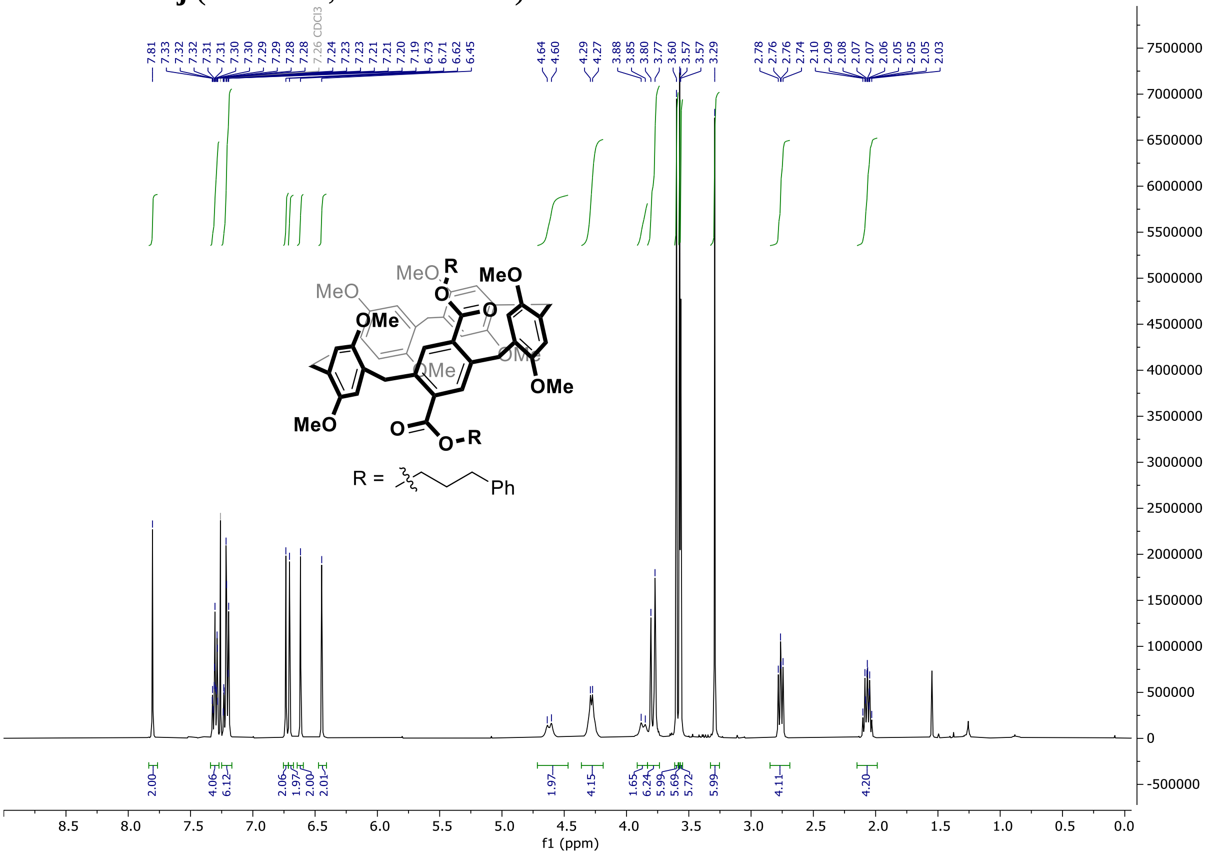

^{13}C{^{1}H} NMR of **4j** (101 MHz, chloroform-*d*)

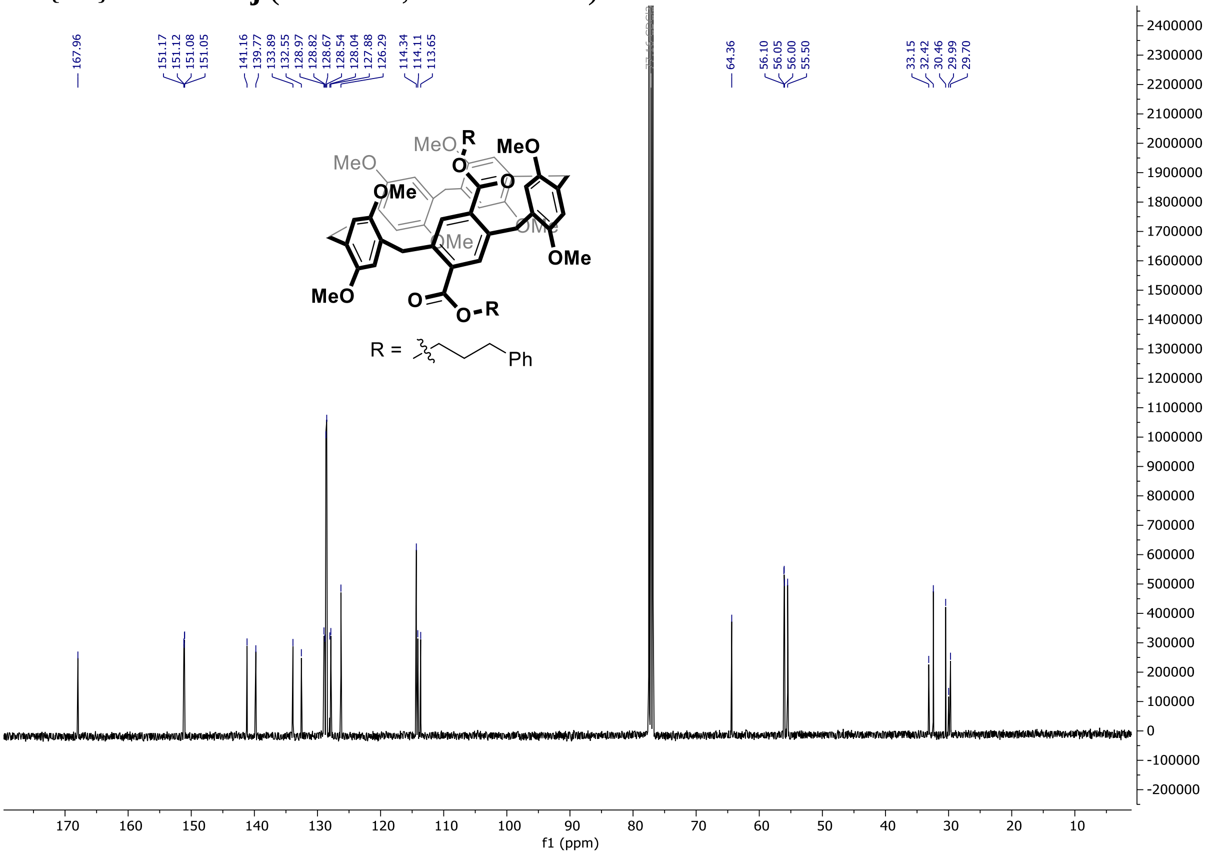

Bis((8*R*,9*S*,13*S*,14*S*)-13-methyl-17-oxo-7,8,9,11,12,13,14,15,16,17-decahydro-6*H*-cyclopenta[*a*]phenanthren-3-yl) (*pS*)-3[2],3[5],5[2],5[5],7[2],7[5],9[2],9[5]-octamethoxy-1,3,5,7,9(1,4)-pentabenzenacyclodecaphane-1[2],1[5]-dicarboxylate (4k)

^{1}H NMR of **4k** (400 MHz, chloroform-*d*)

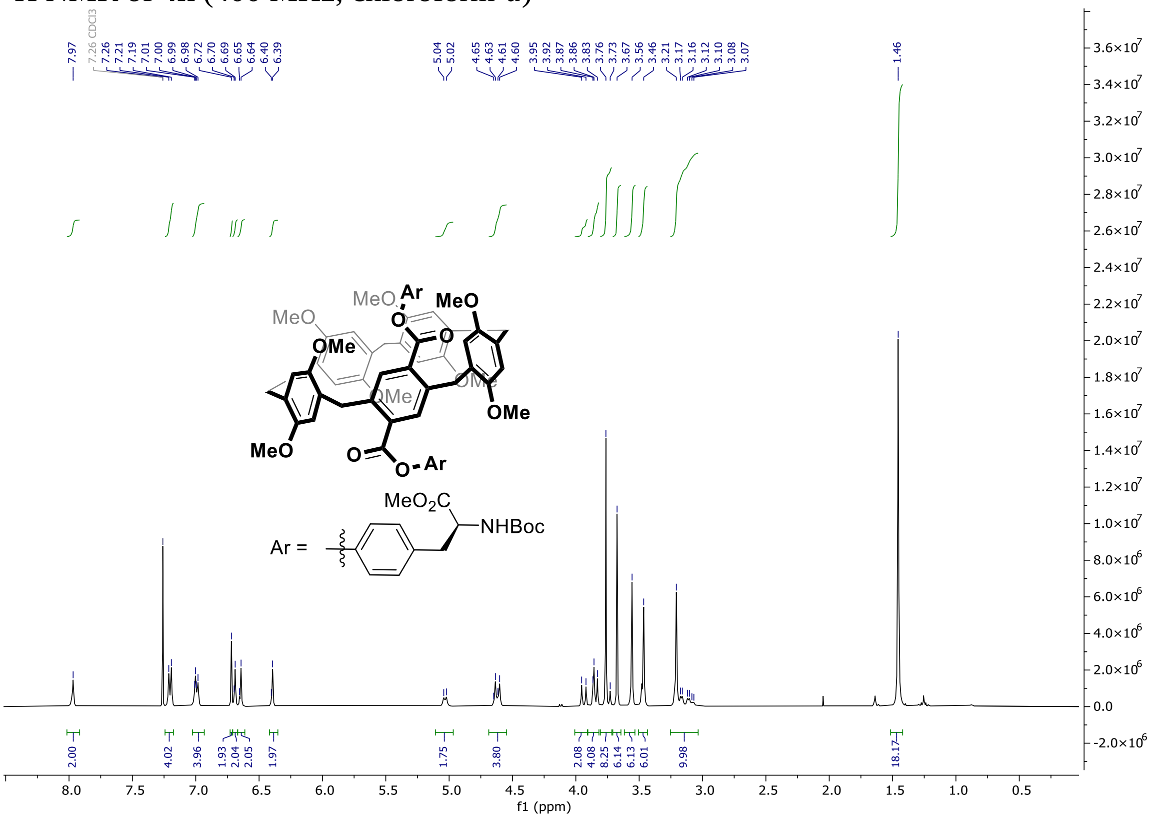

^{13}C{^{1}H} NMR of **4k** (101 MHz, chloroform-*d*)

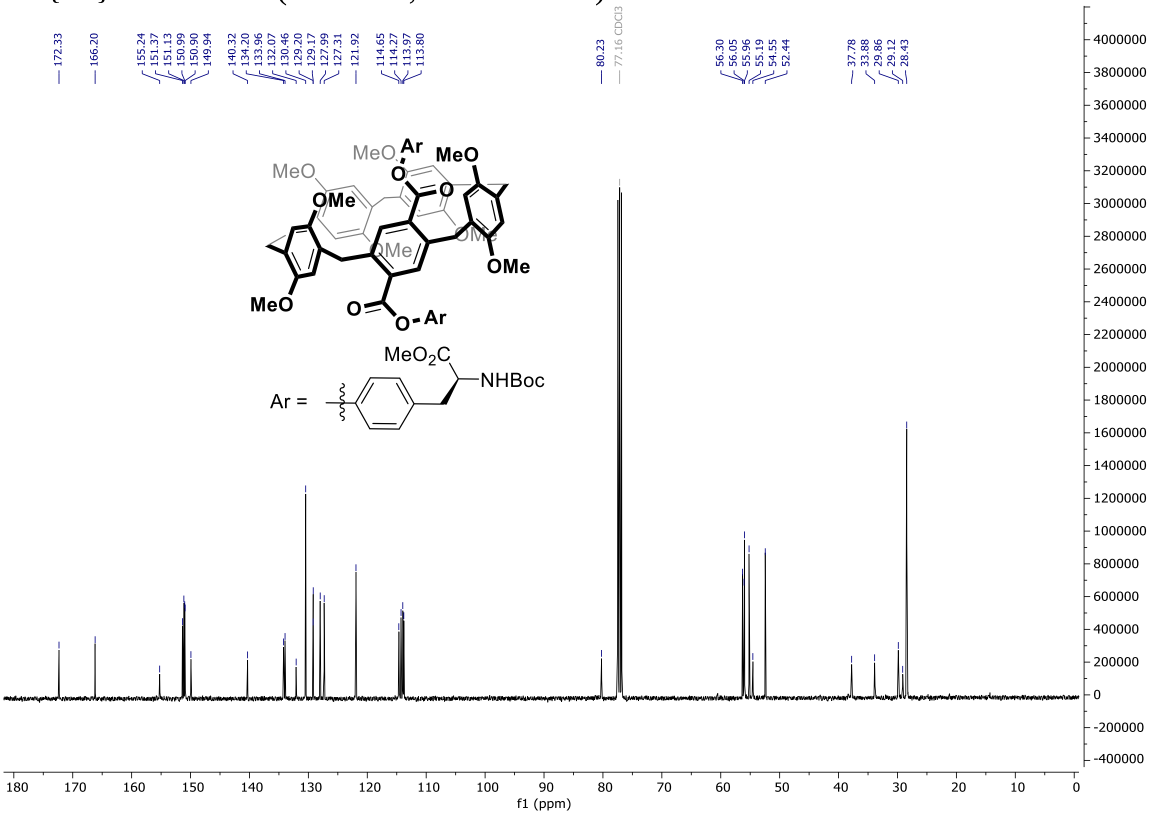

^{1}H NMR of crude reaction mixture for **4k** (400 MHz, chloroform-*d*) with highlighted diastereomeric ratio determination

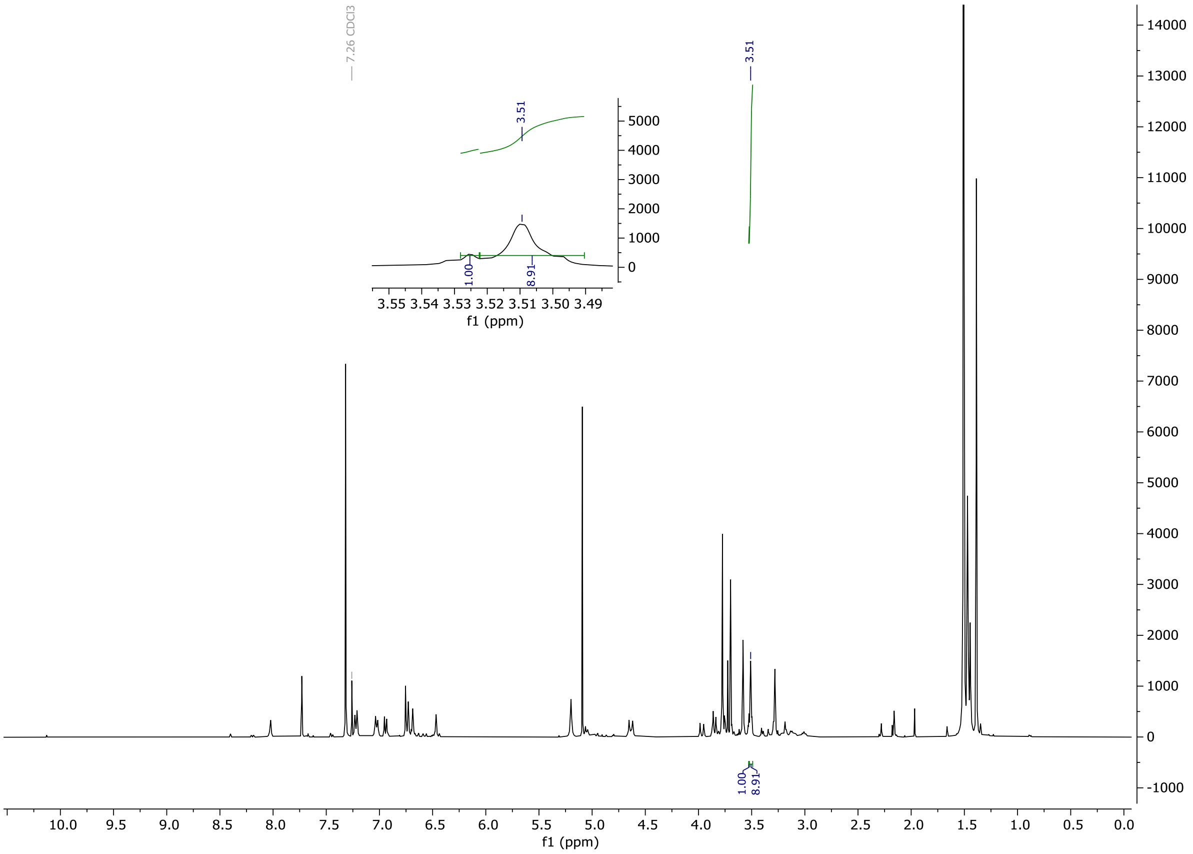

Bis((8*R*,9*S*,13*S*,14*S*)-13-methyl-17-oxo-7,8,9,11,12,13,14,15,16,17-decahydro-6*H*-cyclopenta[*a*]phenanthren-3-yl) (*pS*)-$3^2,3^5,5^2,5^5,7^2,7^5,9^2,9^5$-octamethoxy-1,3,5,7,9(1,4)-pentabenzenacyclodecaphane-$1^2,1^5$-dicarboxylate (4l)

^{1}H NMR of **4l** (400 MHz, chloroform-*d*)

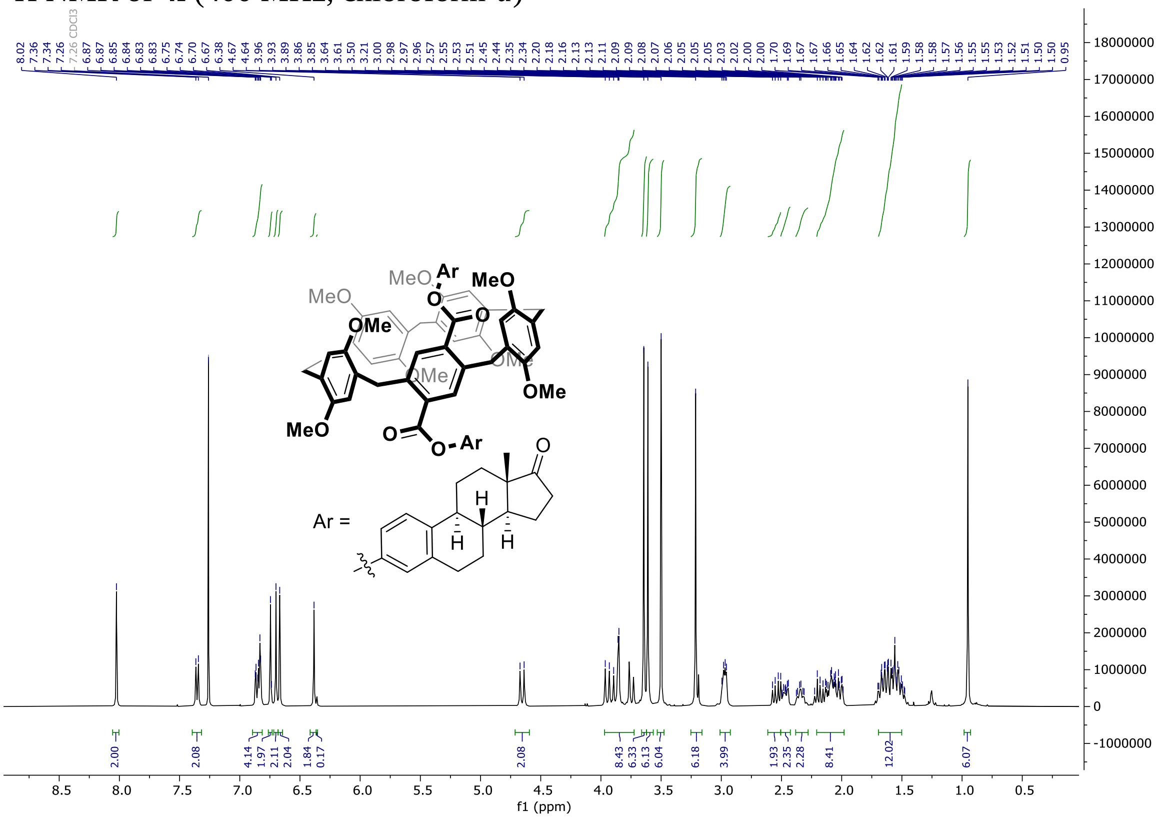

^{13}C{^{1}H} NMR of **4l** (101 MHz, chloroform-*d*)

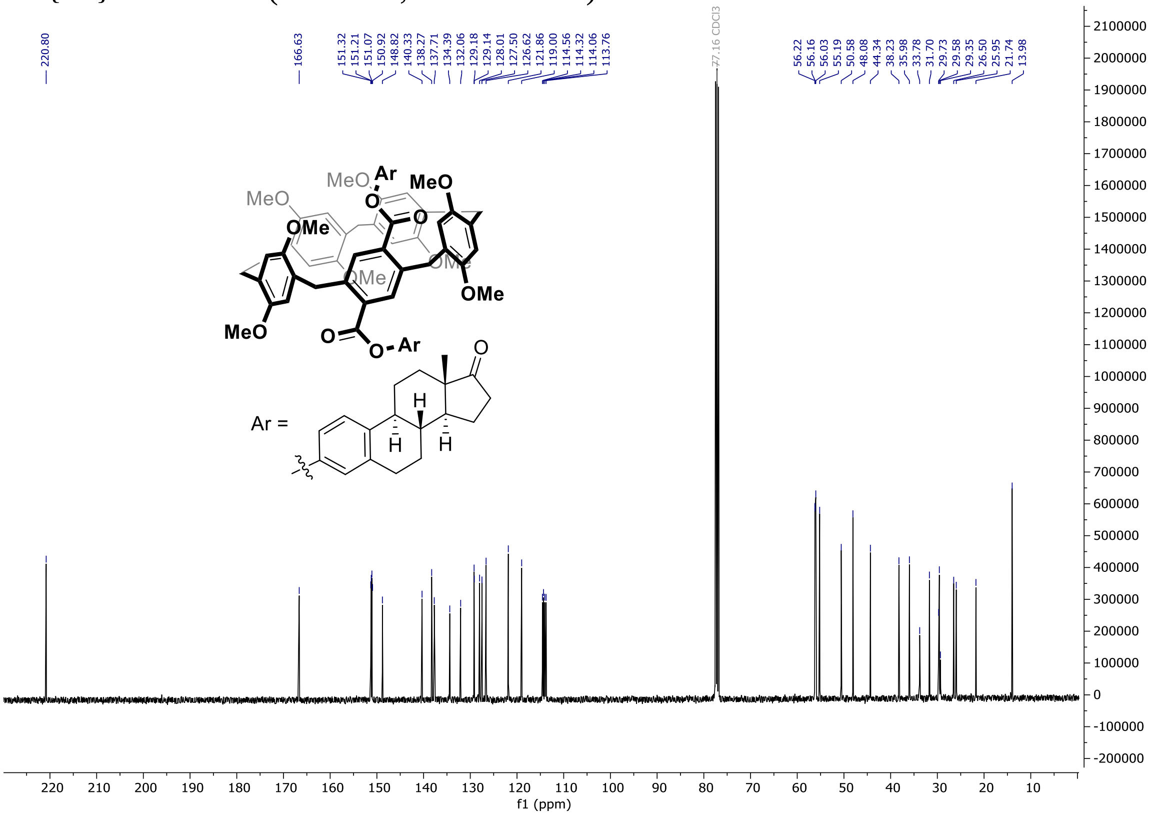

^{1}H NMR of crude reaction mixture for **4l** (400 MHz, chloroform-*d*) with highlighted diastereomeric ratio determination

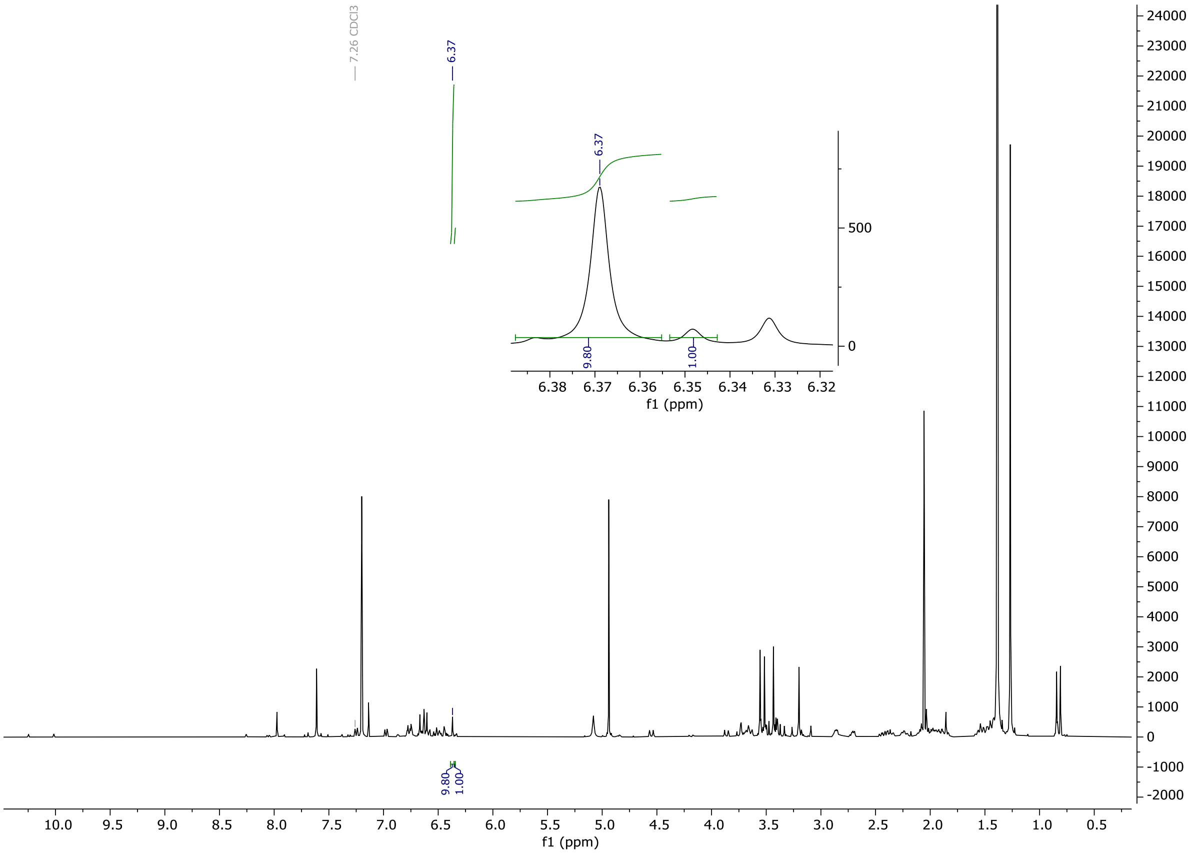

Bis(4-((2*S*,3*R*)-3-((*S*)-3-((*tert*-butyldimethylsilyl)oxy)-3-(4-fluorophenyl)propyl)-1-(4-fluorophenyl)-4-oxoazetidin-2-yl)phenyl) (*pS*)-$3^2,3^5,5^2,5^5,7^2,7^5,9^2,9^5$-octamethoxy-1,3,5,7,9(1,4)-pentabenzenacyclodecaphane-$1^2,1^5$-dicarboxylate (4m)

^{1}H NMR of **4m** (400 MHz, chloroform-*d*)

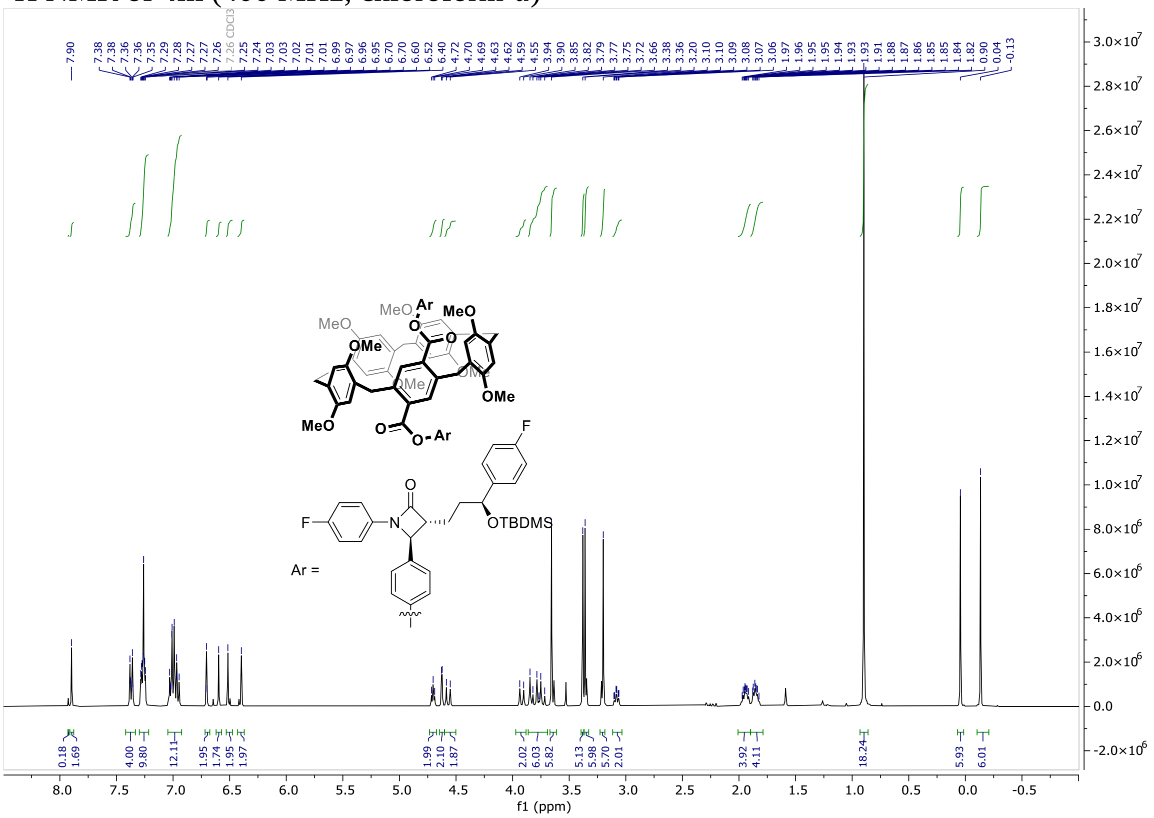

^{13}C{^{1}H} NMR of **4m** (101 MHz, chloroform-*d*)

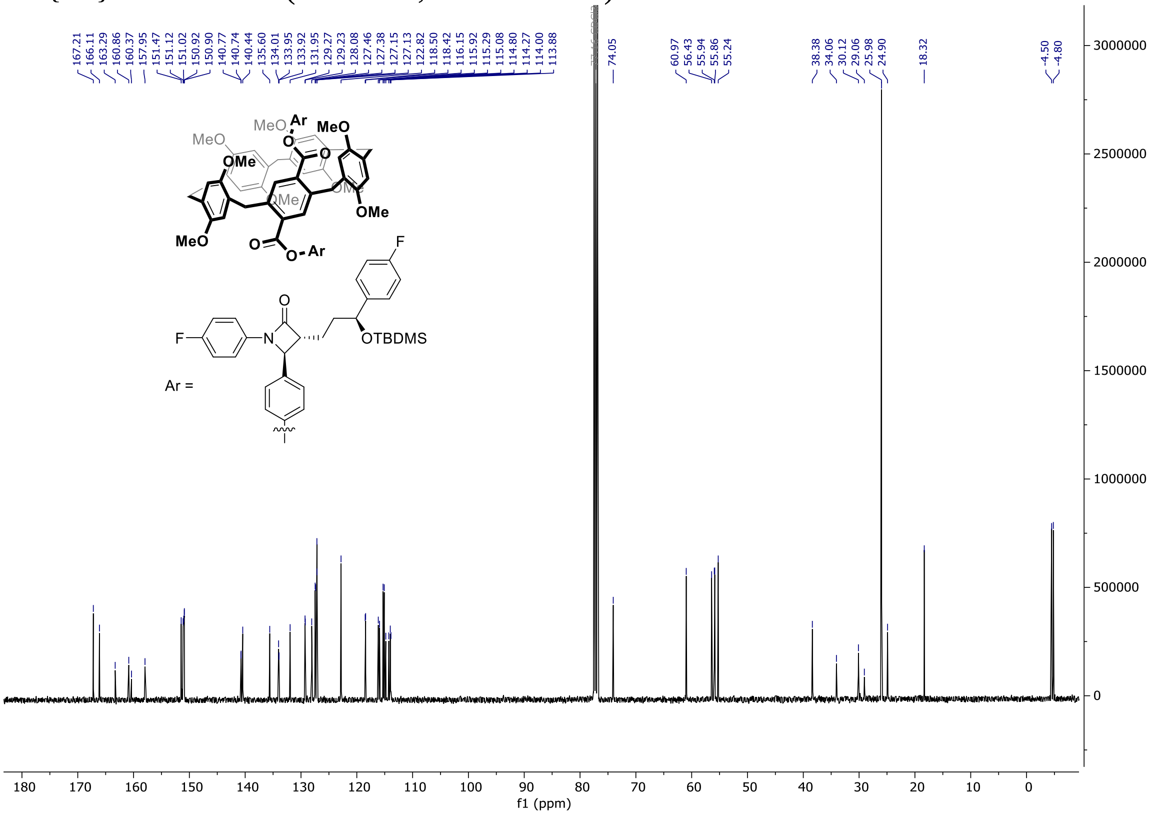

^{19}F NMR of **4m** (376 MHz, chloroform-*d*)

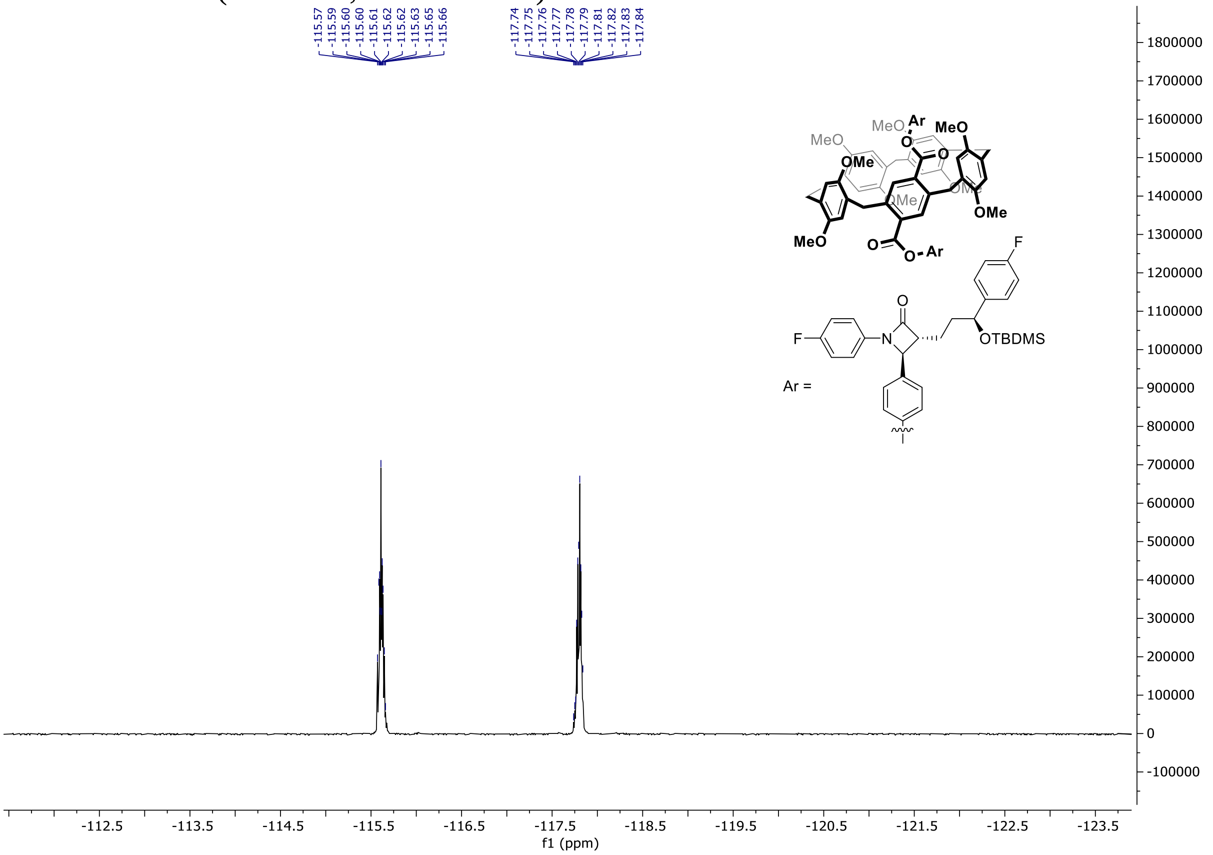

^{1}H NMR of crude reaction mixture for **4m** (400 MHz, chloroform-*d*) with highlighted diastereomeric ratio determination

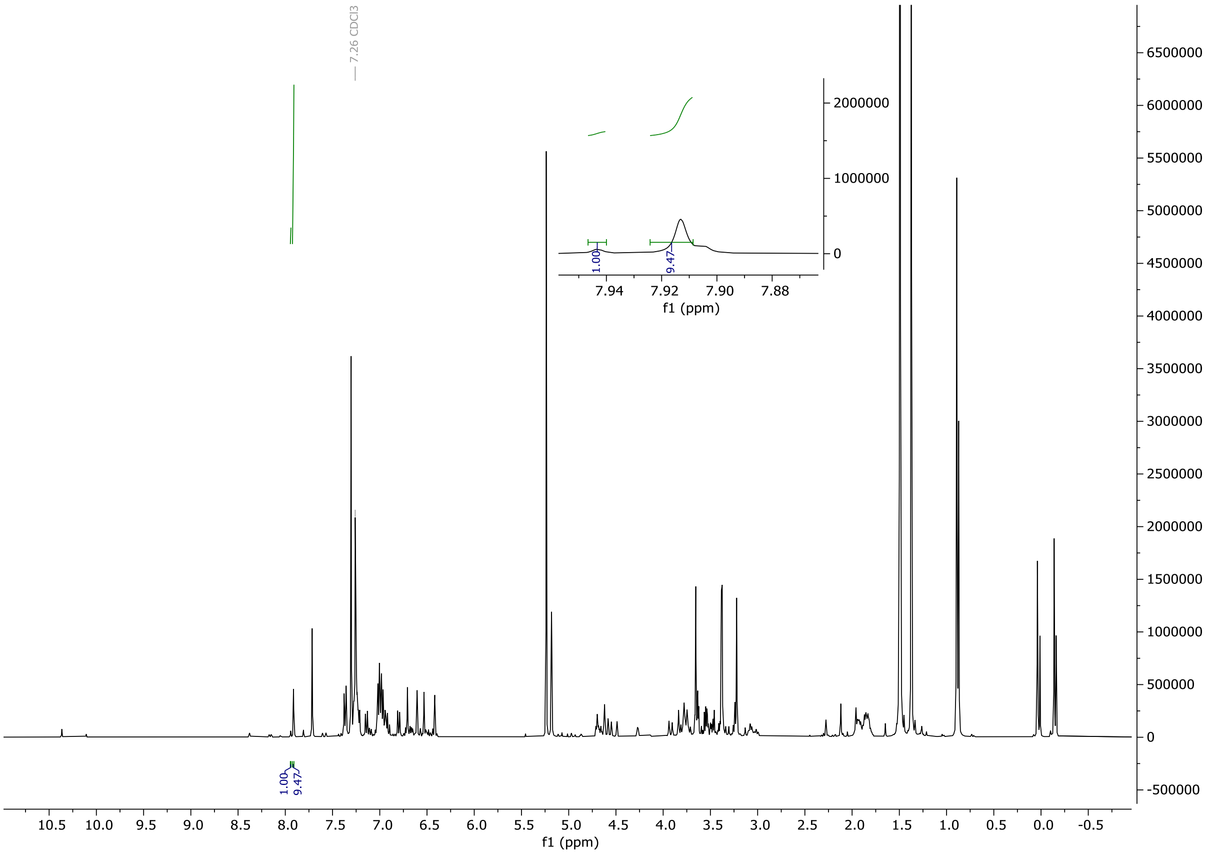

Bis(4-((2*S*,3*R*)-1-(4-fluorophenyl)-3-((*S*)-3-(4-fluorophenyl)-3-hydroxypropyl)-4-oxoazetidin-2-yl)phenyl) (*pS*)-3²,3⁵,5²,5⁵,7²,7⁵,9²,9⁵-octamethoxy-1,3,5,7,9(1,4)-pentabenzenacyclodecaphane-1²,1⁵-dicarboxylate (4n)

^{1}H NMR of **4n** (400 MHz, chloroform-*d*)

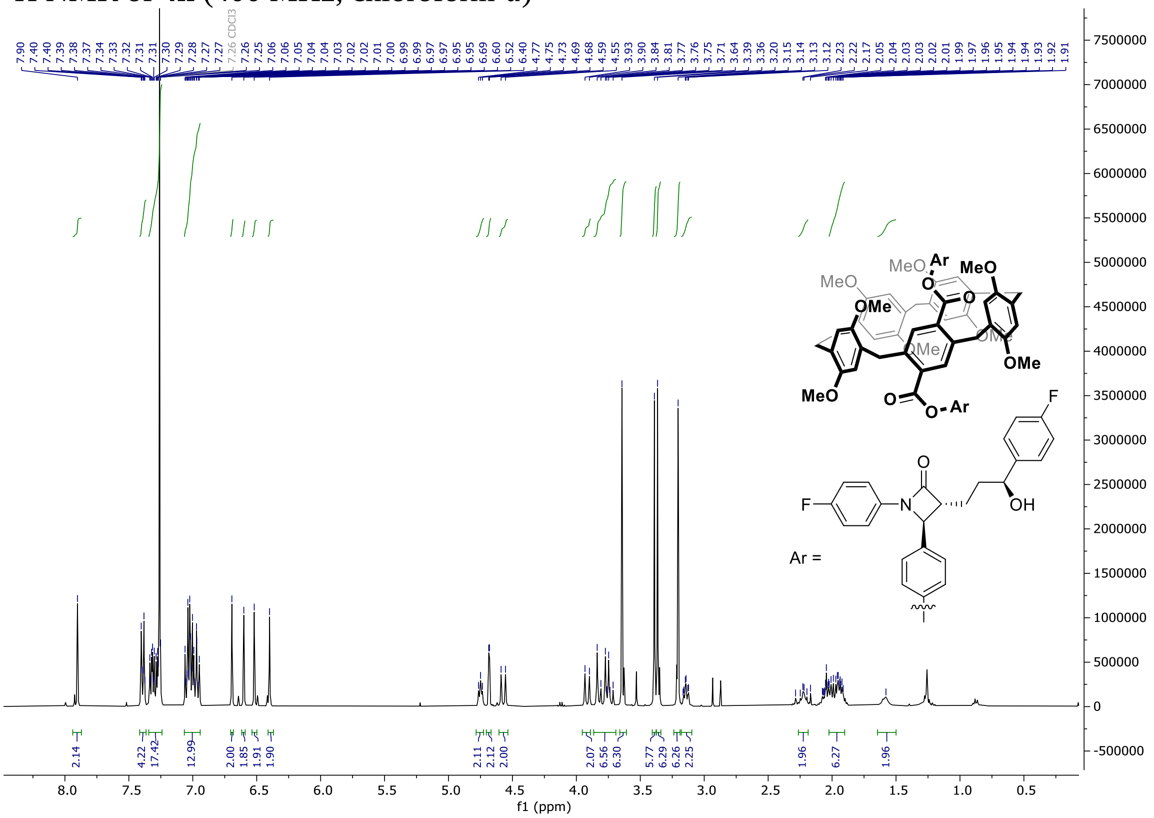

^{13}C{^{1}H} NMR of **4n** (101 MHz, chloroform-*d*)

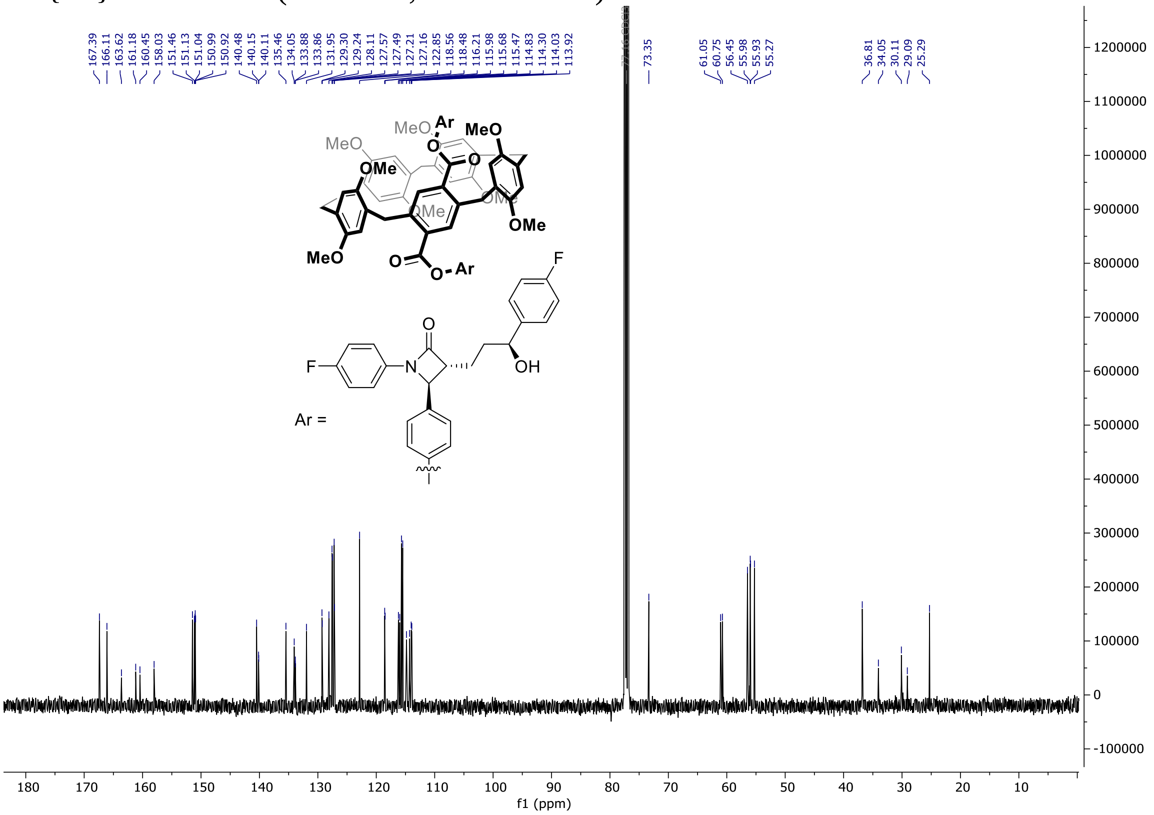

^{19}F NMR of **4n** (376 MHz, chloroform-*d*)

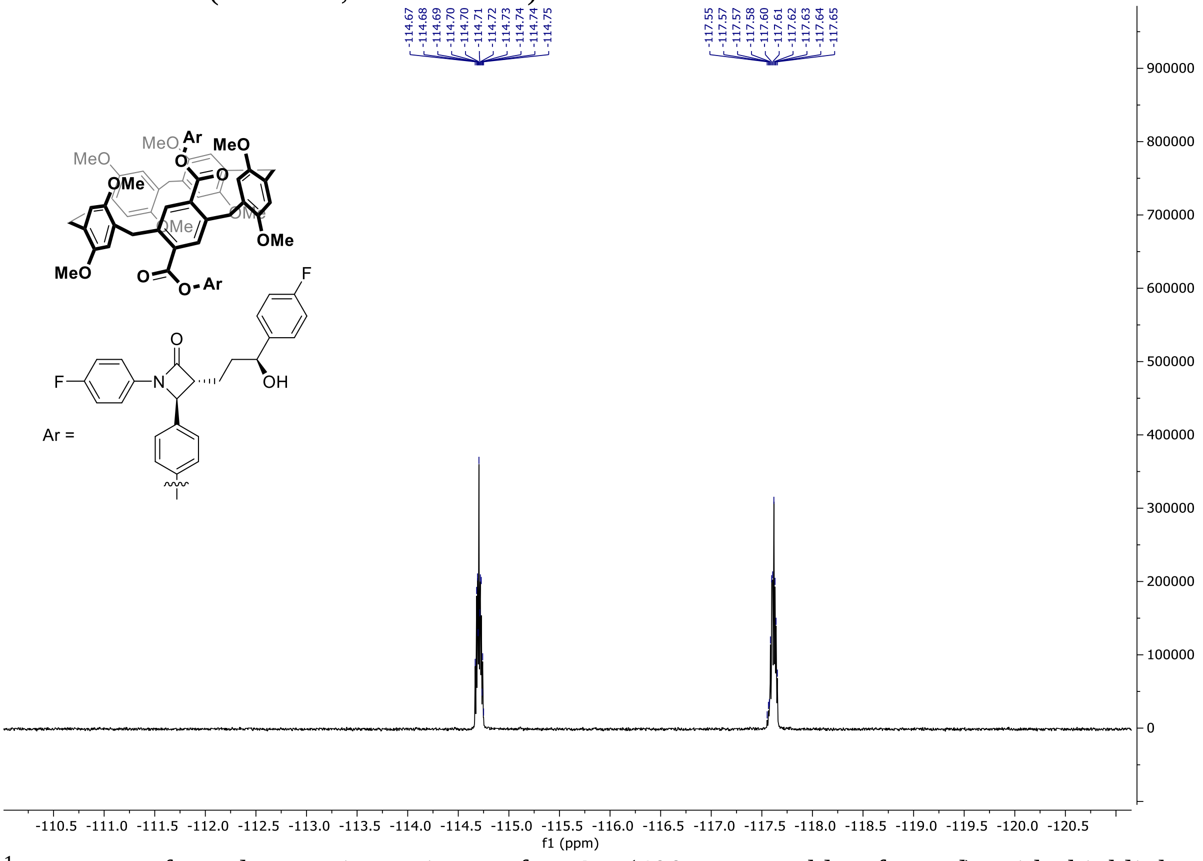

^{1}H NMR of crude reaction mixture for **4n** (400 MHz, chloroform-*d*) with highlighted diastereomeric ratio determination

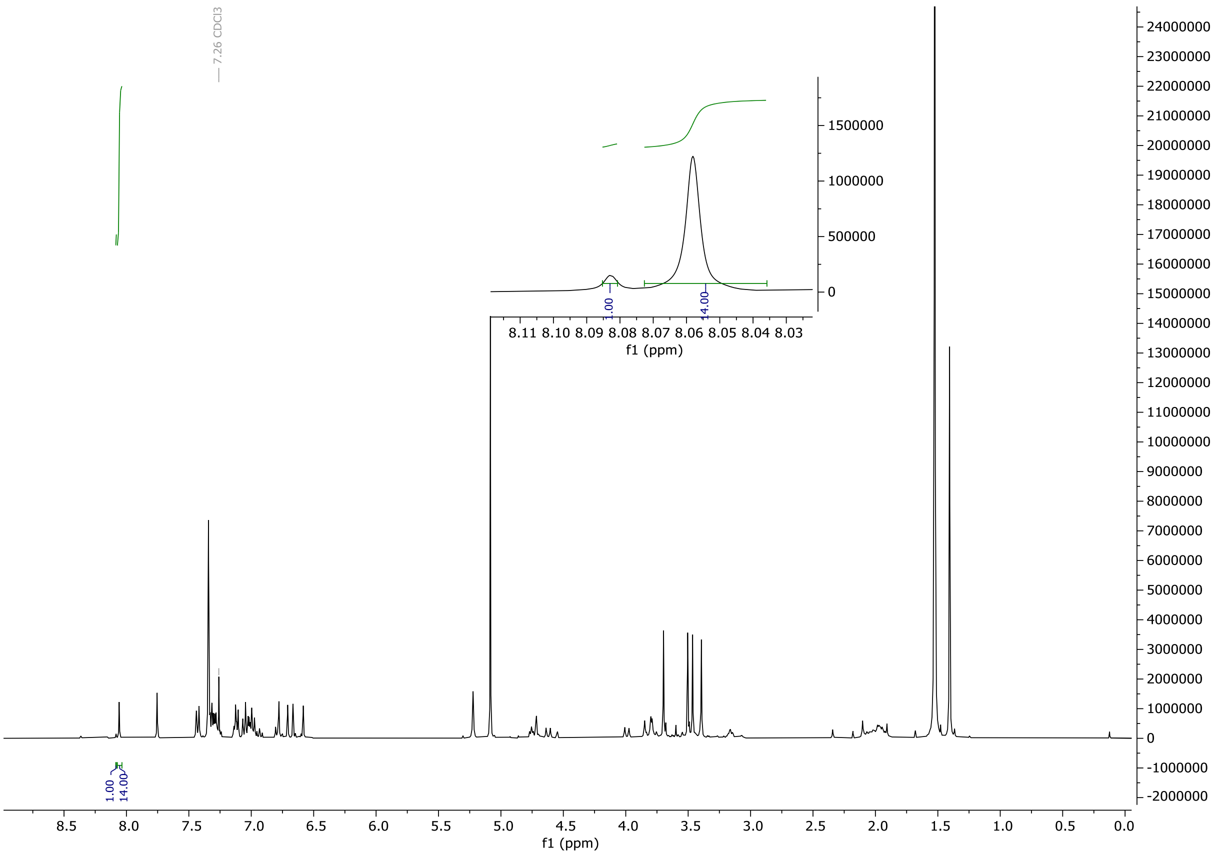

Naphthalen-2-yl (*pS*)-1^5-([1,1'-biphenyl]-4-yl)-3^2,3^5,5^2,5^5,7^2,7^5,9^2,9^5-octamethoxy-1,3,5,7,9(1,4)-pentabenzenacyclodecaphane-1^2-carboxylate (5a)

^{1}H NMR of **5a** (400 MHz, chloroform-*d*)

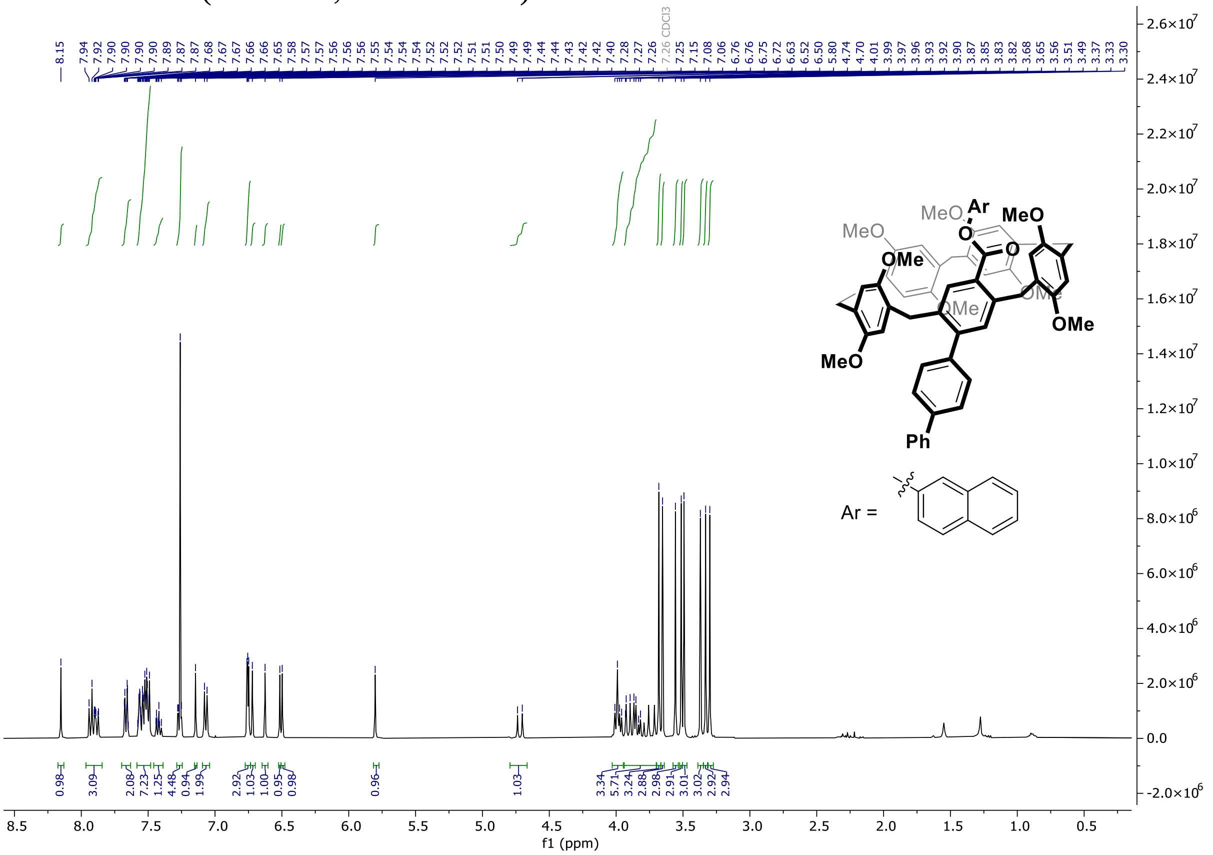

^{13}C{^{1}H} NMR of **5a** (101 MHz, chloroform-*d*)

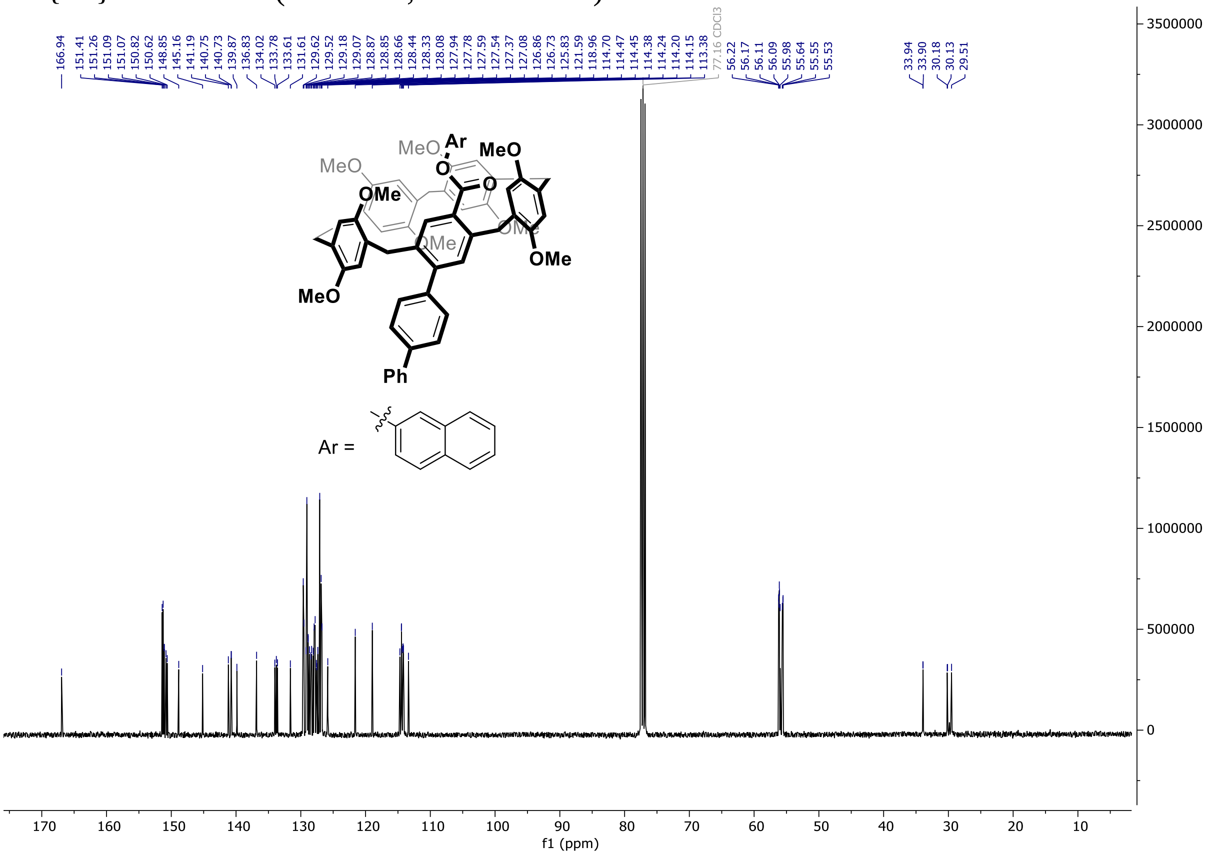

[1,1'-Biphenyl]-4-yl (*pS*)-1^5-([1,1'-biphenyl]-4-yl)-3^2,3^5,5^2,5^5,7^2,7^5,9^2,9^5-octamethoxy-1,3,5,7,9(1,4)-pentabenzenacyclodecaphane-1^2-carboxylate (5b)

^{1}H NMR of **5b** (400 MHz, chloroform-*d*)

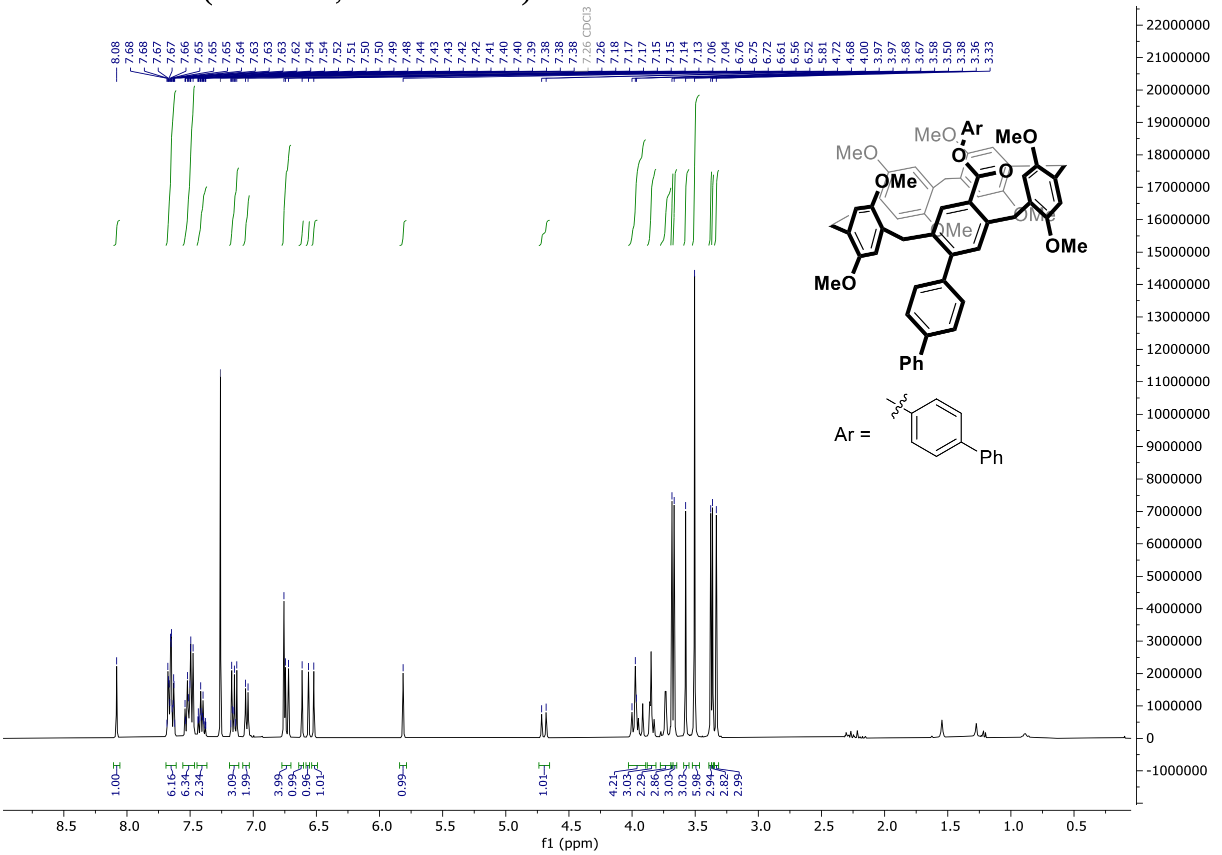

^{13}C{^{1}H} NMR of **5b** (101 MHz, chloroform-*d*)

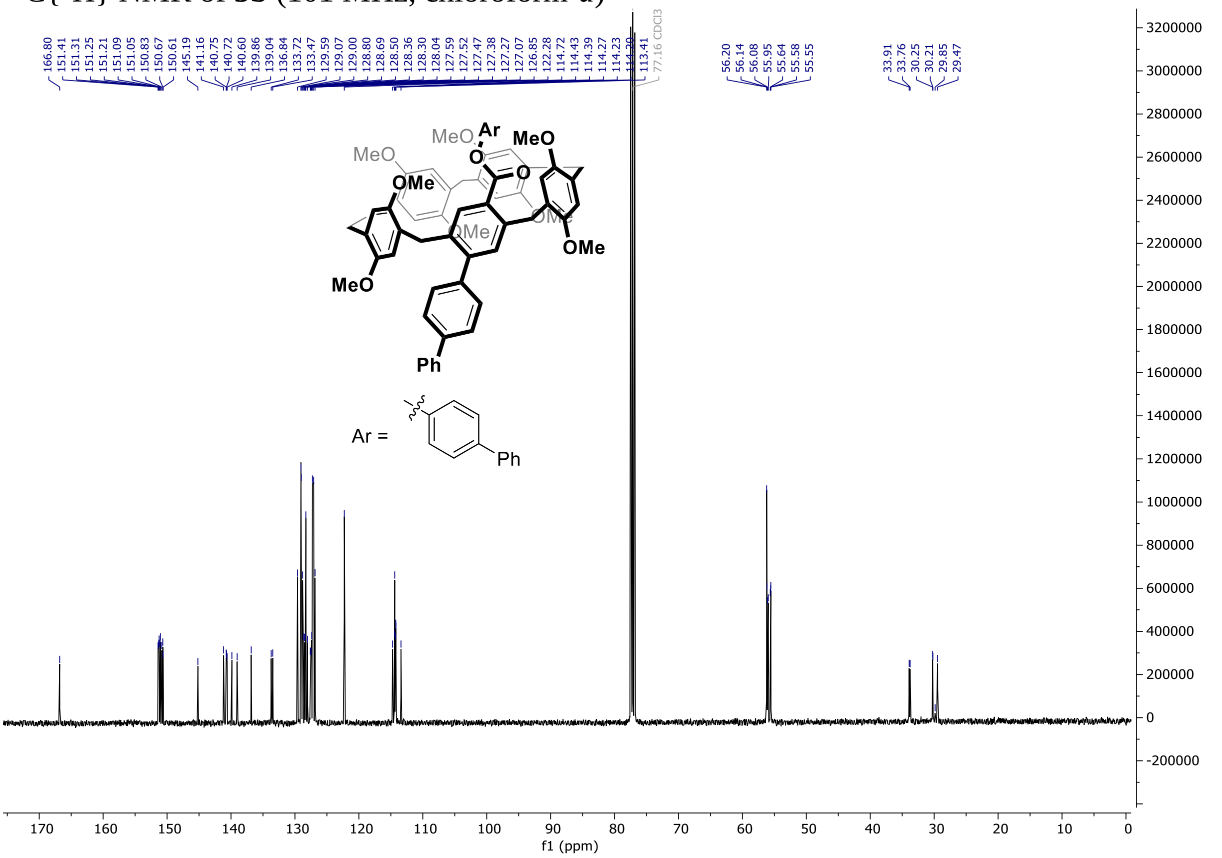

(8*R*,9*S*,13*S*,14*S*)-1^3-Methyl-17-oxo-7,8,9,11,12,13,14,15,16,17-decahydro-6*H*-cyclopenta[*a*]phenanthren-3-yl (*pS*)-1^5-([1,1'-biphenyl]-4-yl)-3^2,3^5,5^2,5^5,7^2,7^5,9^2,9^5-octamethoxy-1,3,5,7,9(1,4)-pentabenzenacyclodecaphane-1^2-carboxylate (5c)

^{1}H NMR of **5c** (400 MHz, chloroform-*d*)

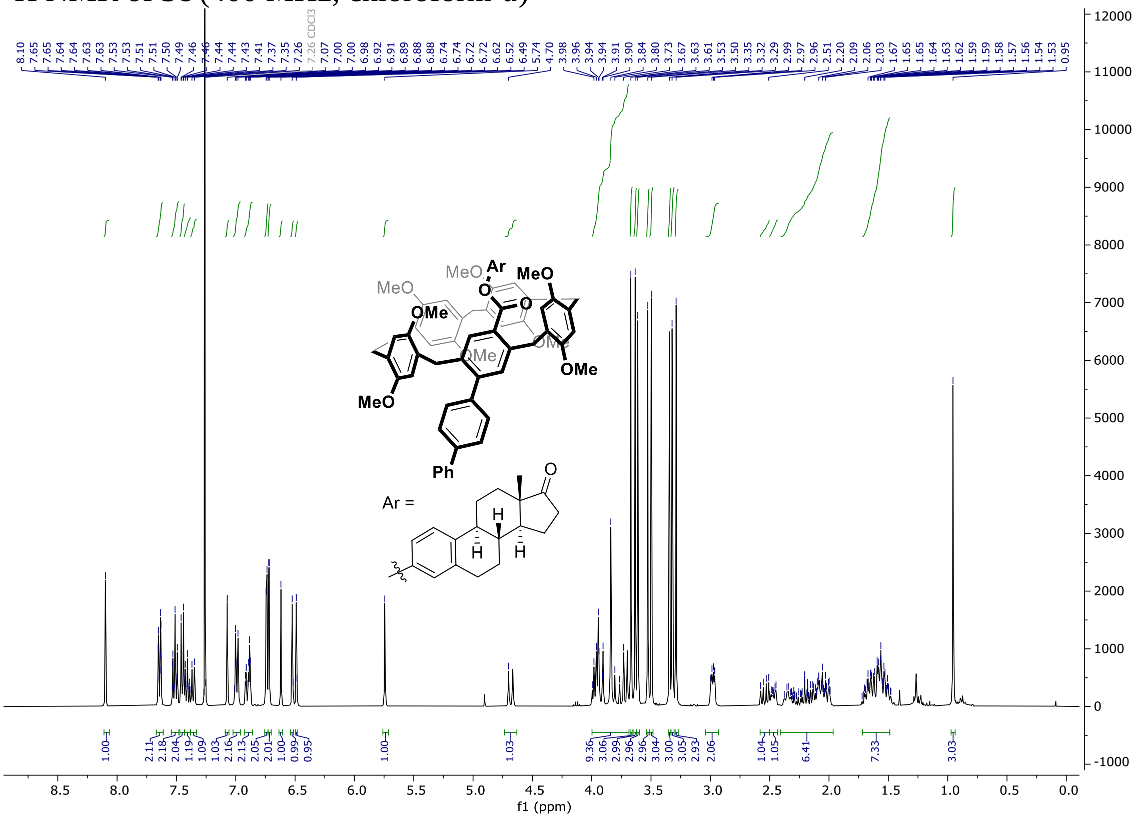

^{13}C{^{1}H} NMR of **5c** (101 MHz, chloroform-*d*)

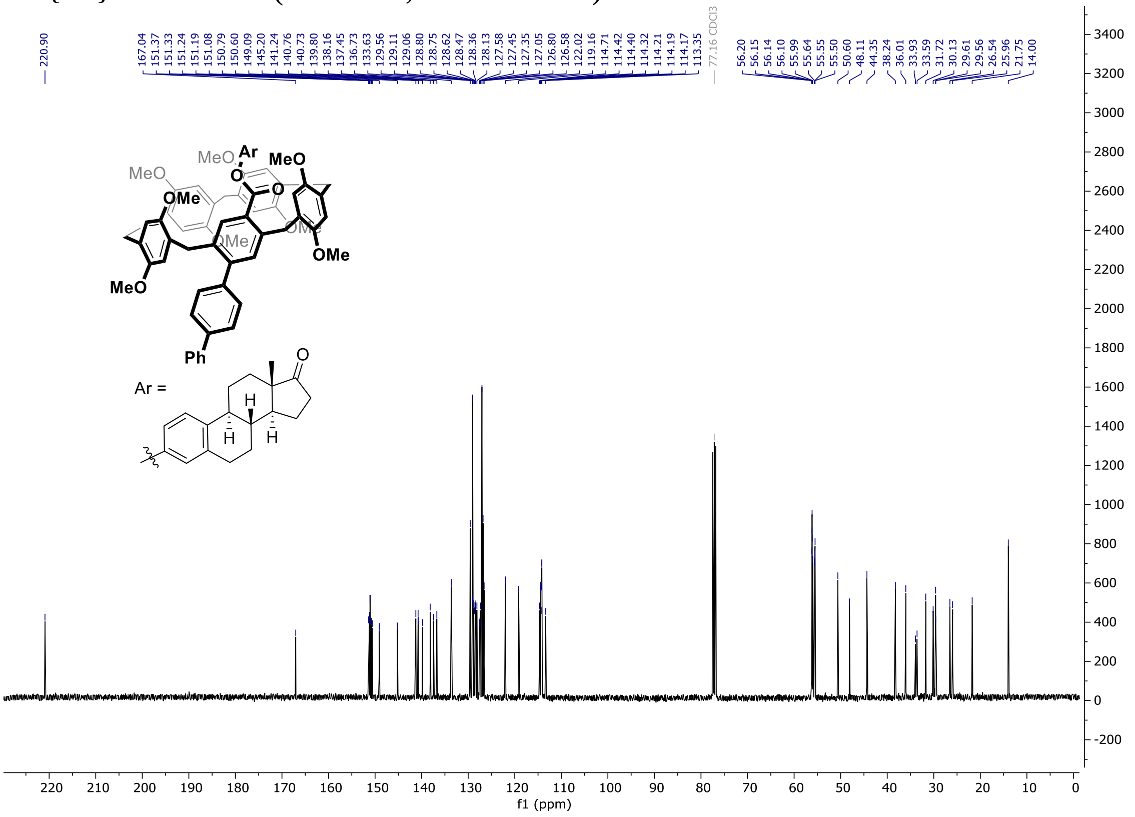

^{1}H NMR of crude reaction mixture for **5c** (400 MHz, chloroform-*d*) with highlighted diastereomeric ratio determination

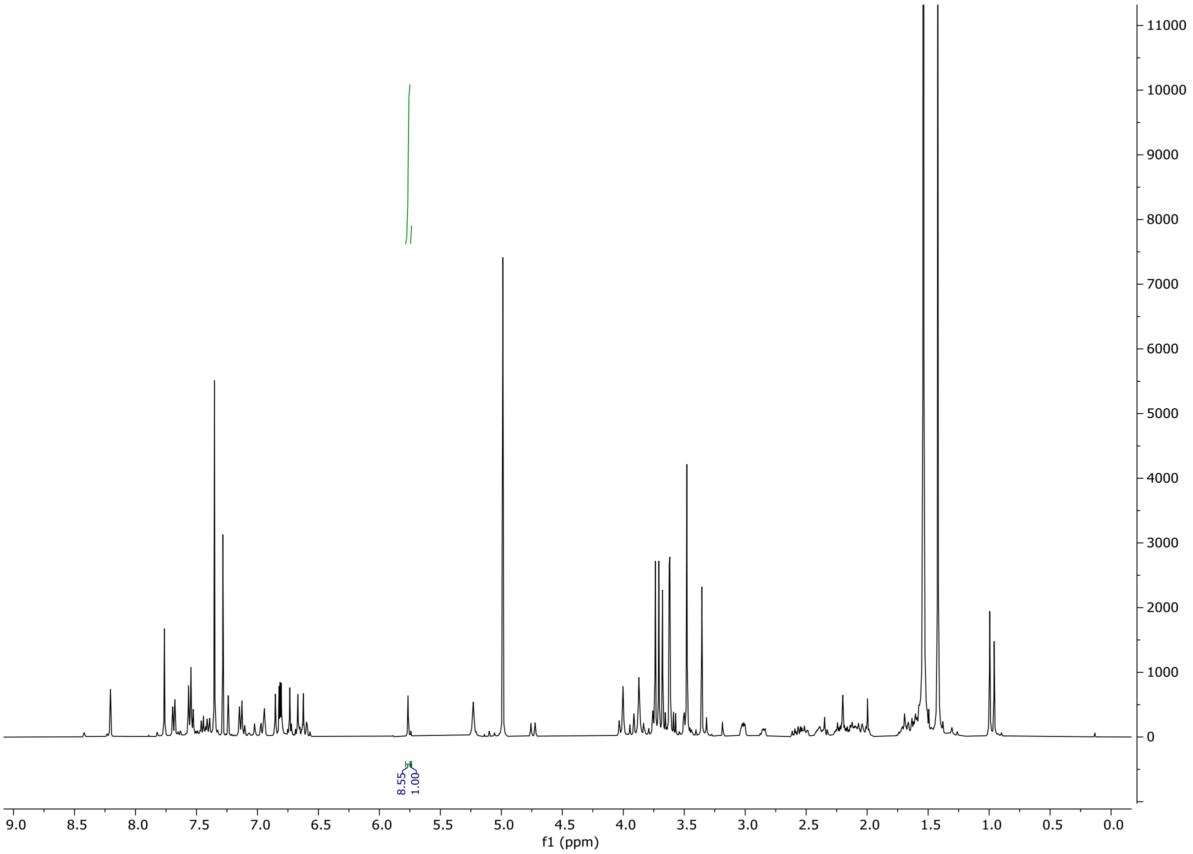

1²-([1,1'-Biphenyl]-4-yl) 1⁵-(naphthalen-2-yl) (*pS*)-3²,3⁵,5²,5⁵,7²,7⁵,9²,9⁵-octamethoxy-1,3,5,7,9(1,4)-pentabenzenacyclodecaphane-1²,1⁵-dicarboxylate (5d)

^{1}H NMR of **5d** (400 MHz, chloroform-*d*)

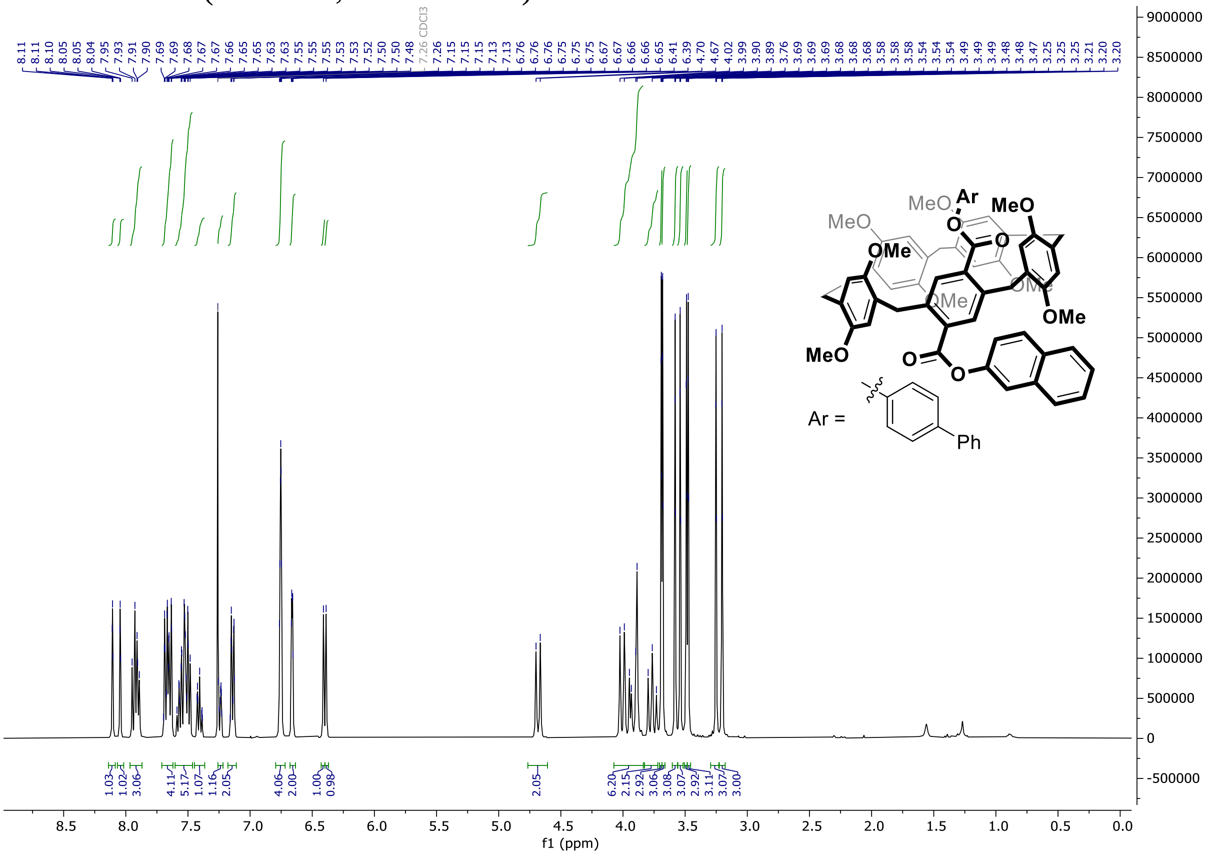

^{13}C{^{1}H} NMR of **5d** (101 MHz, chloroform-*d*)

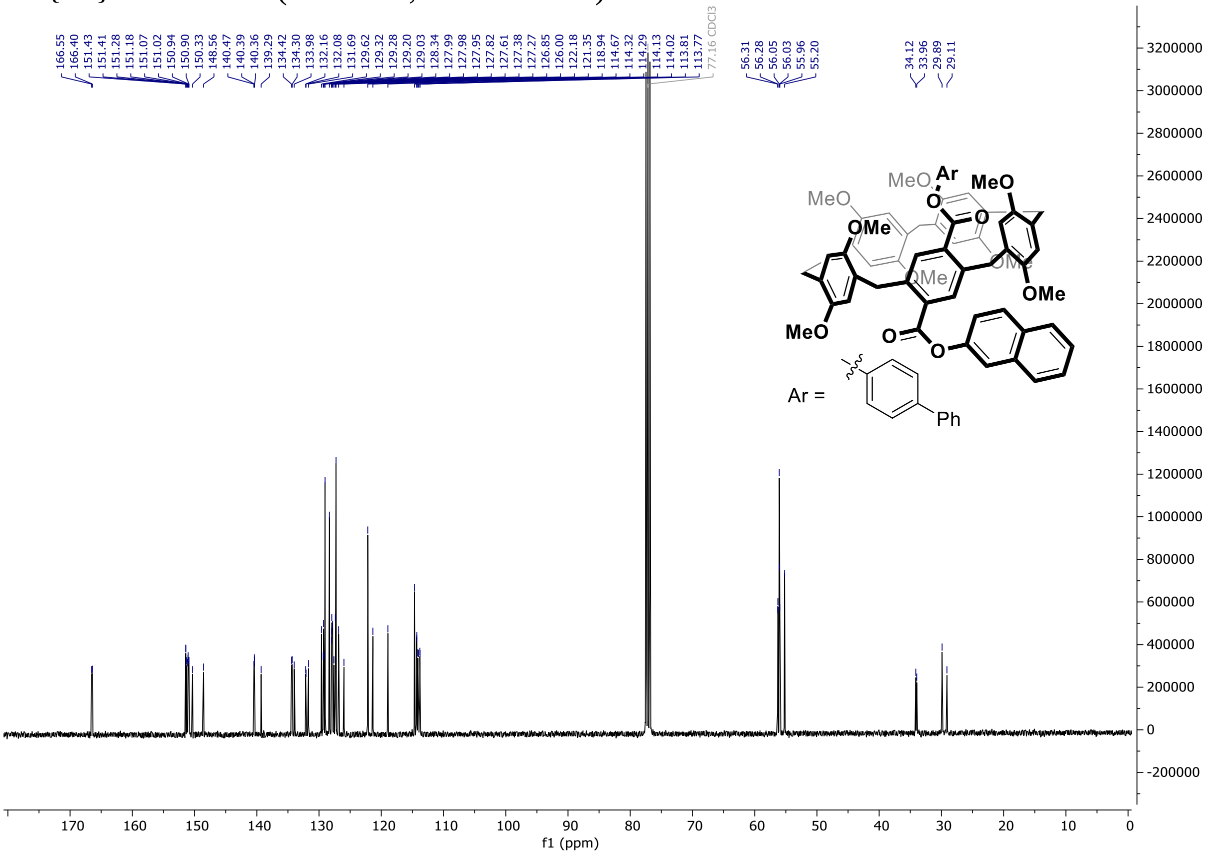

1[2]-((8*R*,9*S*,13*S*,14*S*)-13-methyl-17-oxo-7,8,9,11,12,13,14,15,16,17-decahydro-6*H*-cyclopenta[*a*]phenanthren-3-yl) 1[5]-(naphthalen-2-yl) (*pS*)-3[2],3[5],5[2],5[5],7[2],7[5],9[2],9[5]-octamethoxy-1,3,5,7,9(1,4)-pentabenzenacyclodecaphane-1[2],1[5]-dicarboxylate (5e)

^{1}H NMR of **5e** (400 MHz, chloroform-*d*)

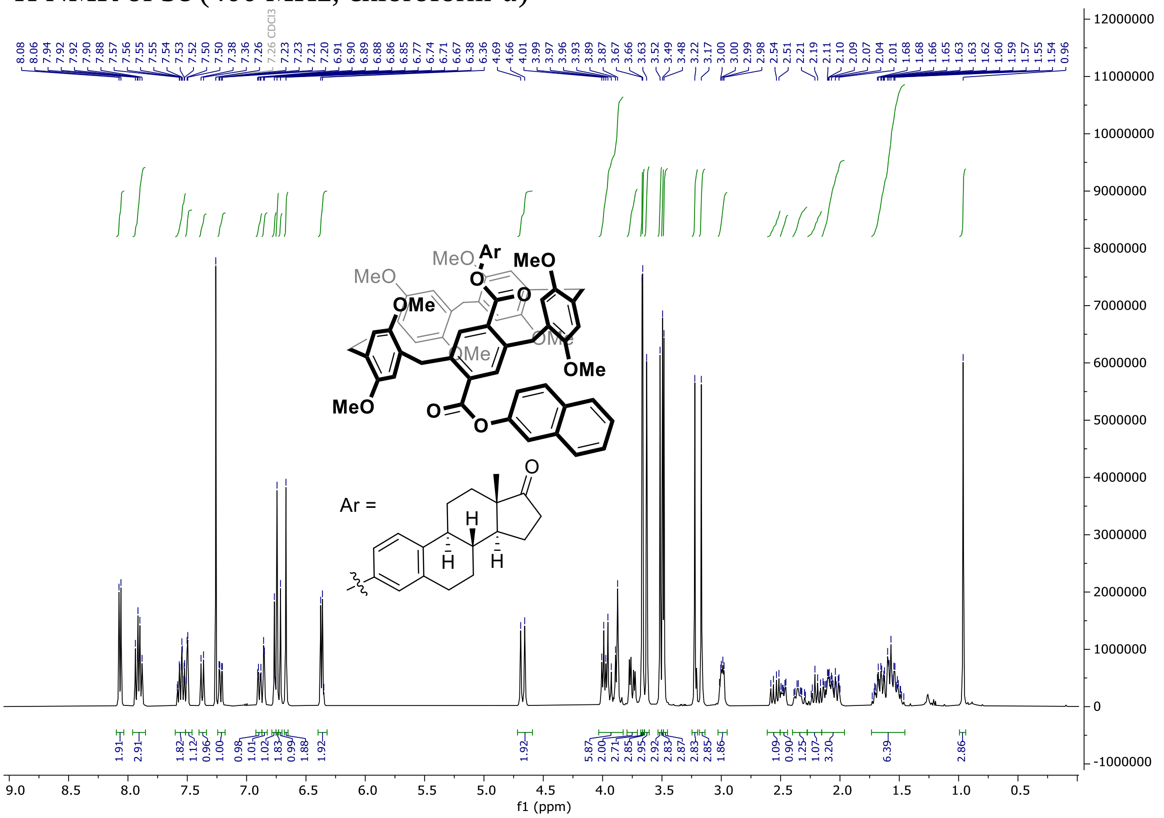

^{13}C{^{1}H} NMR of **5e** (101 MHz, chloroform-*d*)

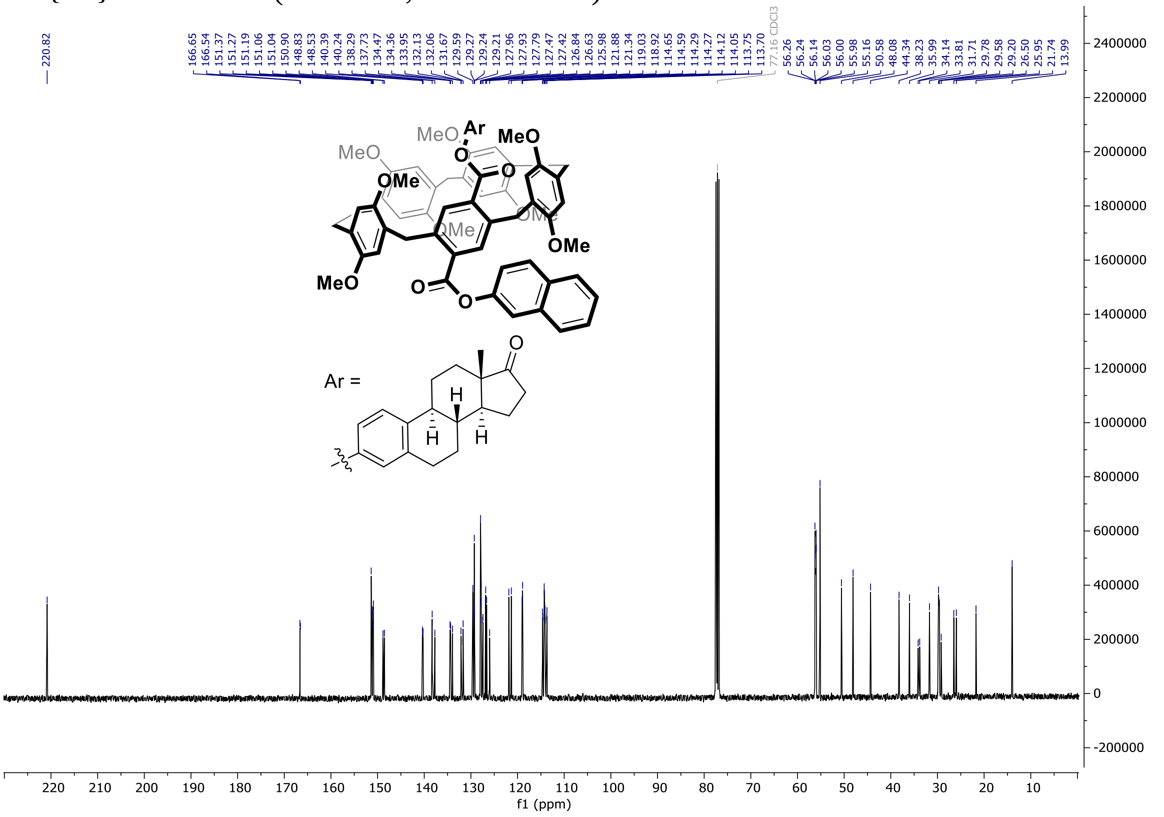

^{1}H NMR of crude reaction mixture for **5e** (400 MHz, chloroform-*d*) with highlighted diastereomeric ratio determination

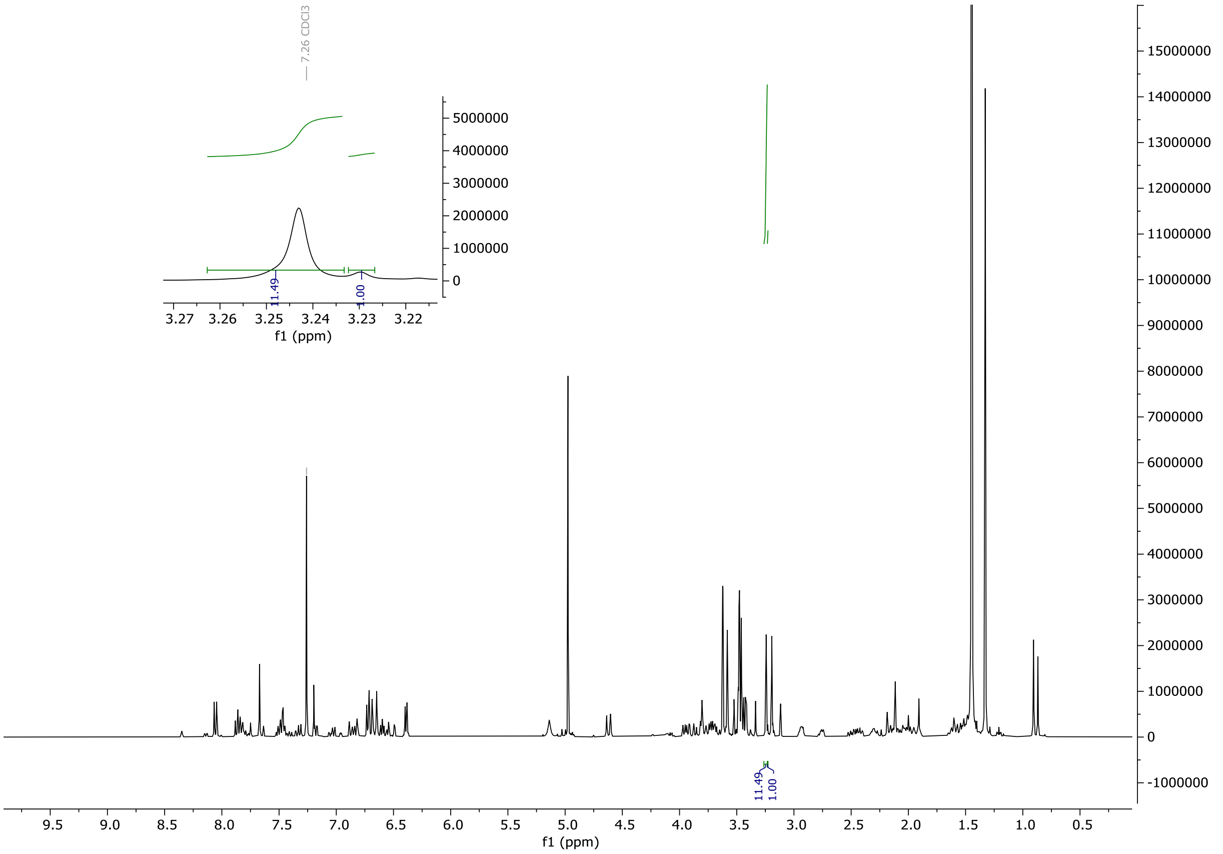

Bis(6-formylnaphthalen-2-yl) (*pS*)-3^2,3^5,5^2,5^5,7^2,7^5,9^2,9^5-octamethoxy-1,3,5,7,9(1,4)-pentabenzenacyclodecaphane-1^2,1^5-dicarboxylate (6)

^{1}H NMR of **6** (400 MHz, chloroform-*d*)

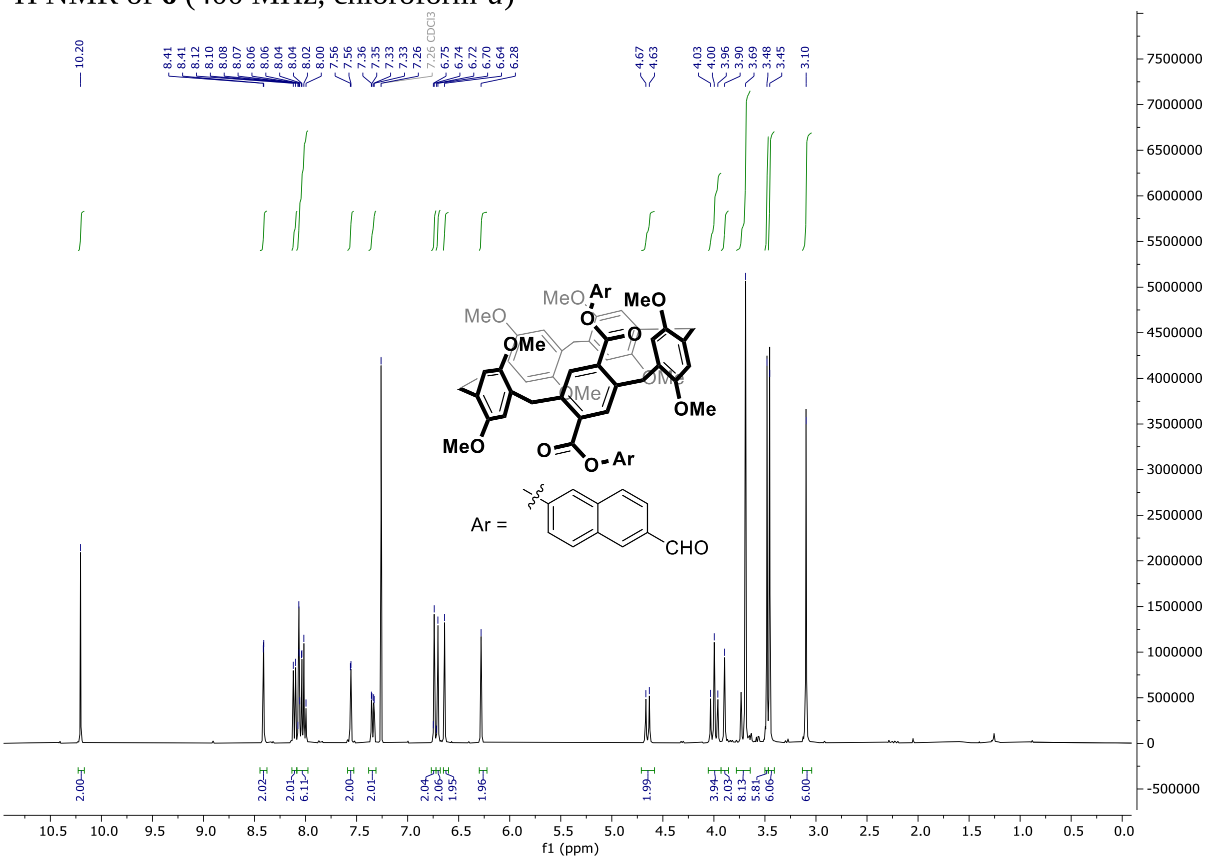

^{13}C{^{1}H} NMR of **6** (101 MHz, chloroform-*d*)

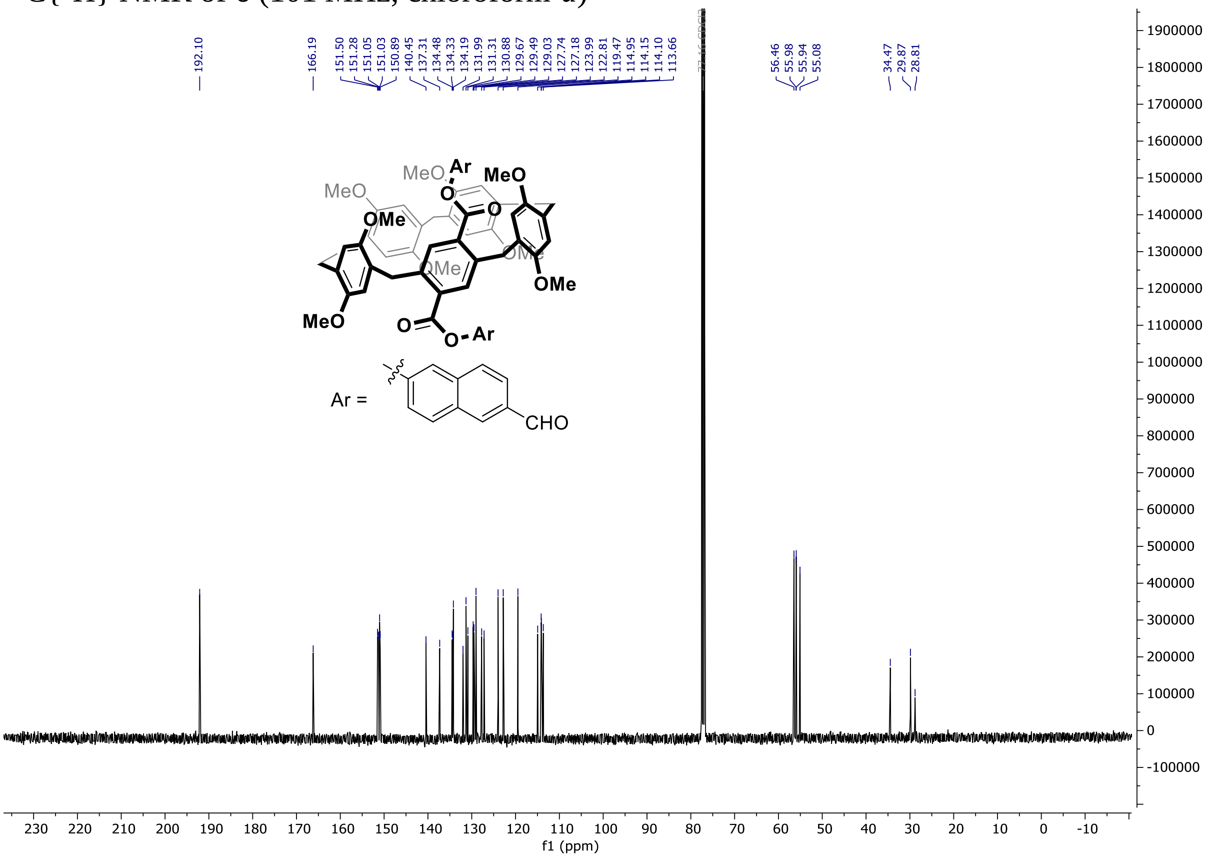

Bis(6-((*E*)-3-ethoxy-3-oxoprop-1-en-1-yl)naphthalen-2-yl) (*pS*)-3²,3⁵,5²,5⁵,7²,7⁵,9²,9⁵-octamethoxy-1,3,5,7,9(1,4)-pentabenzenacyclodecaphane-1²,1⁵-dicarboxylate (7)

^{1}H NMR of **7** (400 MHz, chloroform-*d*)

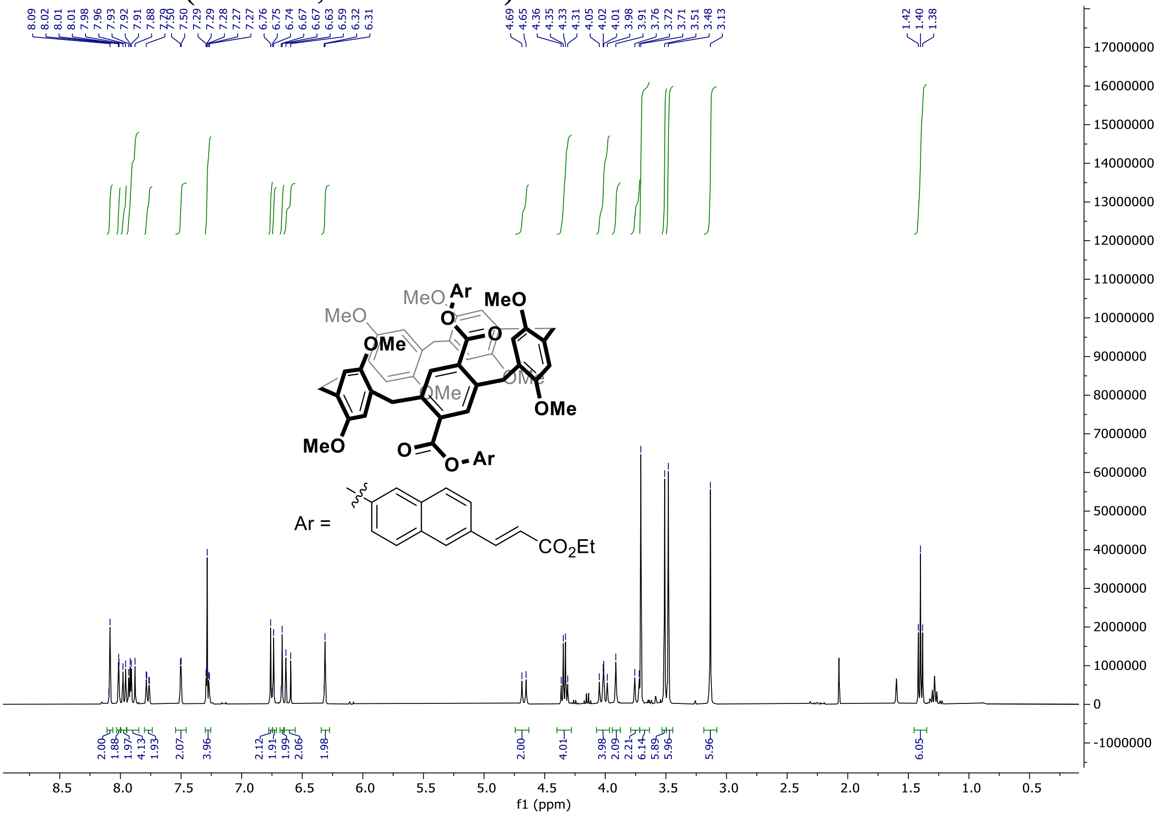

^{13}C{^{1}H} NMR of **7** (101 MHz, chloroform-*d*)

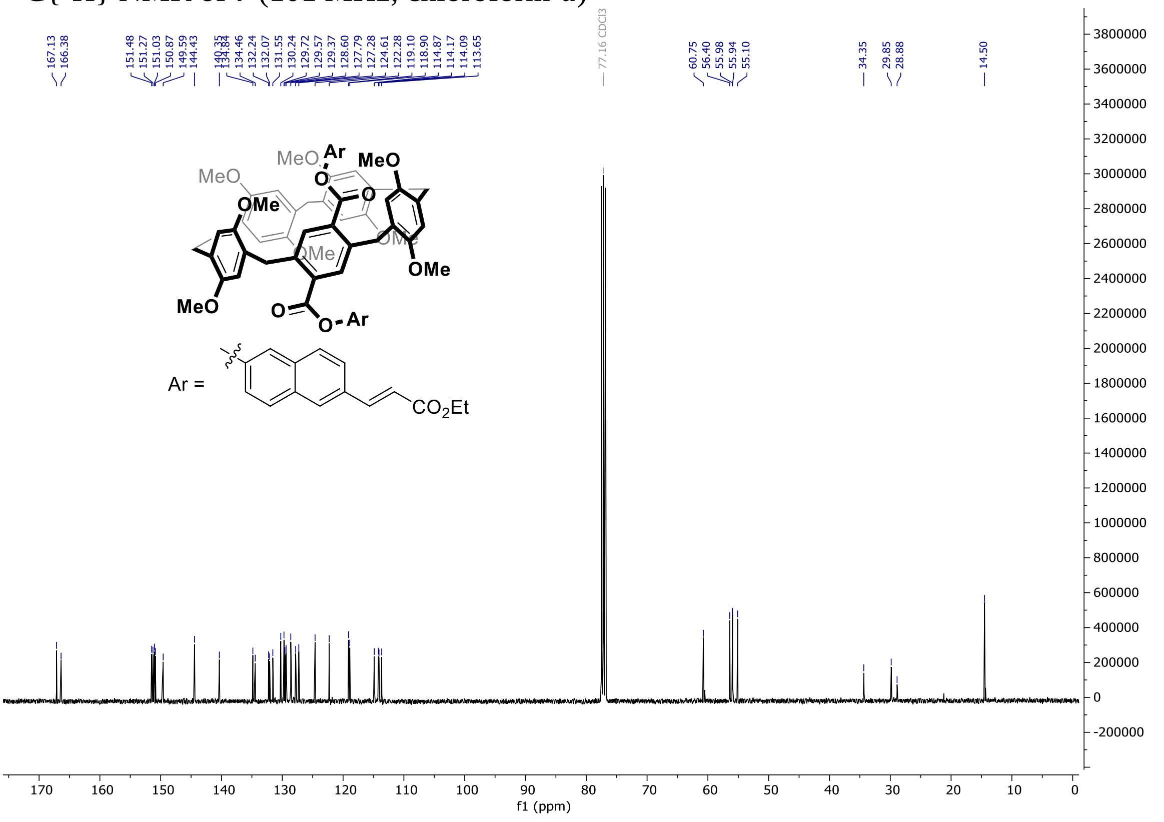

Rotaxane (8)

^{1}H NMR of **8** (400 MHz, chloroform-*d*)

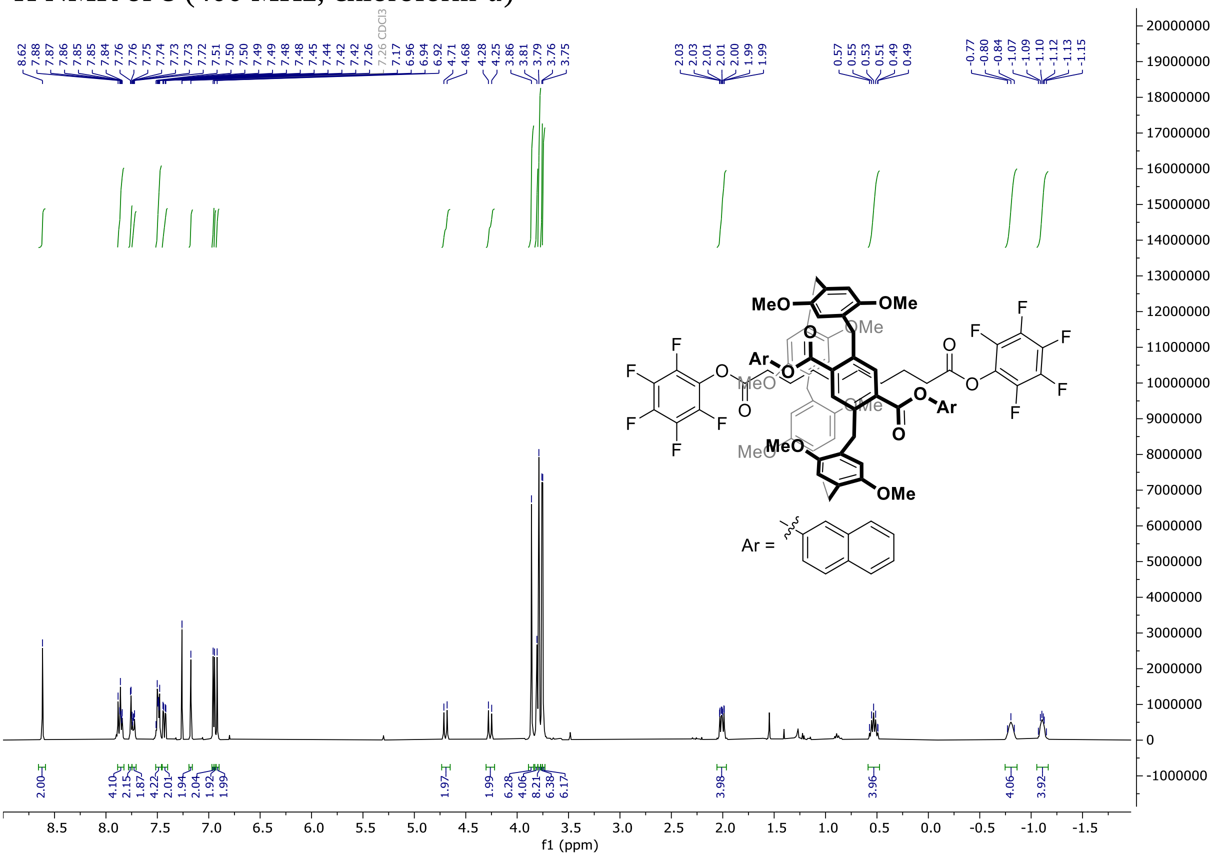

^{13}C{^{1}H} NMR of **8** (101 MHz, chloroform-*d*)

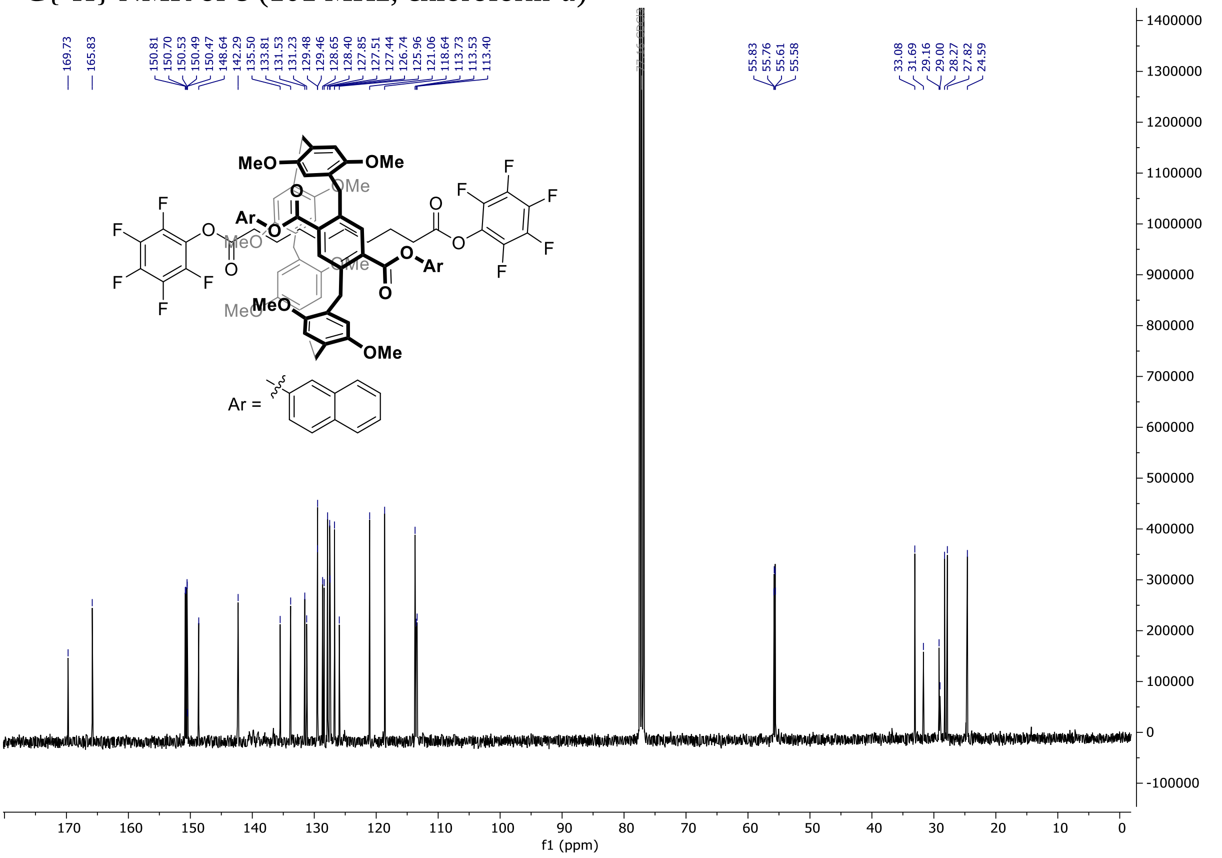

^{19}F NMR of **8** (376 MHz, chloroform-*d*)

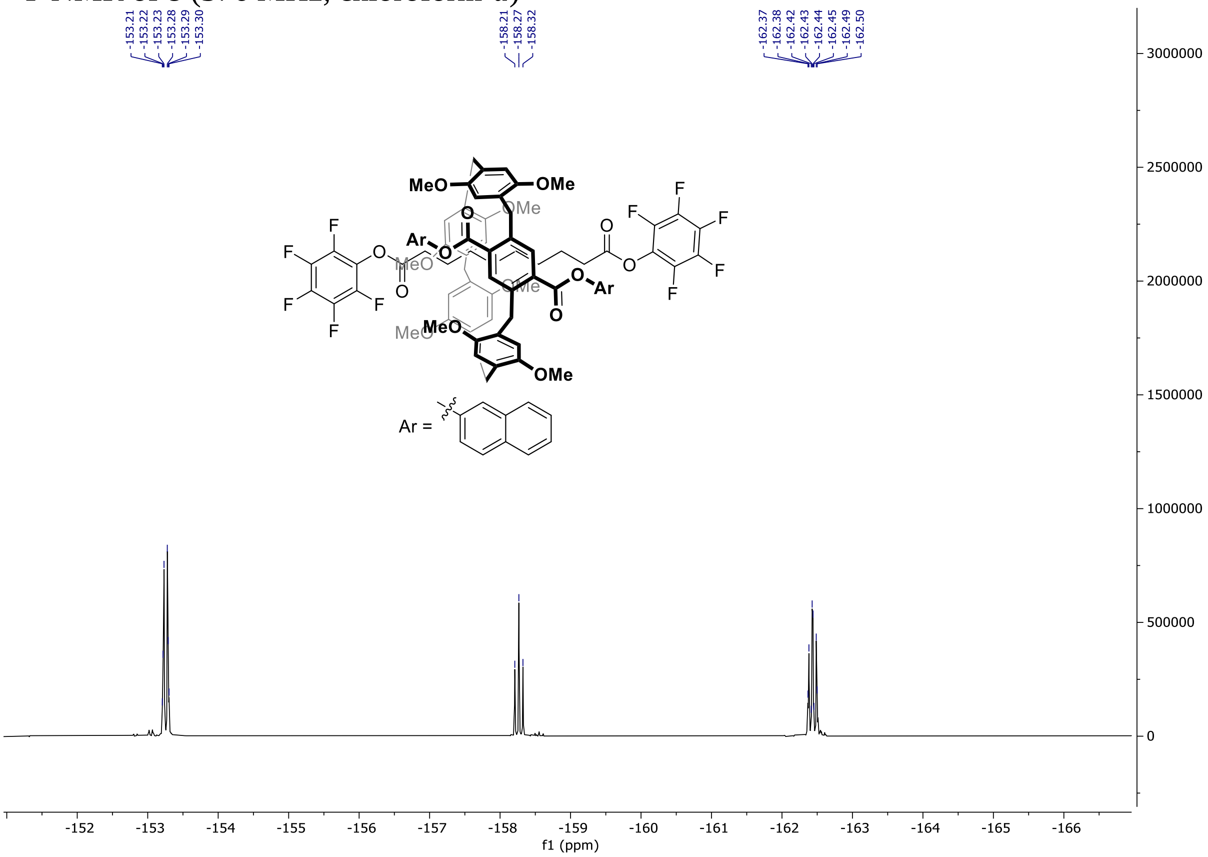

Chiral HPLC

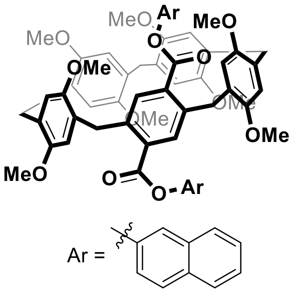

Conditions: Chiralpak IC column
mobile phase: *n*-heptane/propan-2-ol – 70:30
λ = 190 nm, V = 1.0 ml/min, t = 25 °C
for **3a**: t_R = 9.3 min (minor), t_R = 12.8 min (major).

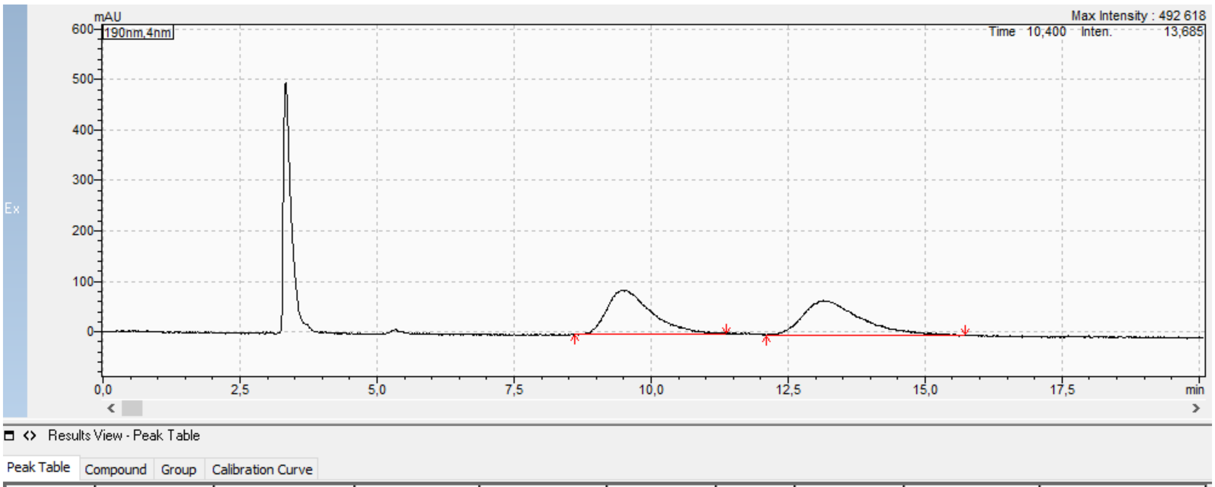

Peak#	Ret. Time	Area	Height	Peak Start	Peak End	Mark	Conc.	Unit	Area%
1	9,513	4966015	88170	8,608	11,371	M	50,104		50,104
2	13,152	4945302	68228	12,107	15,733	M	49,896		49,896
Total		9911316	156398				100,000		100,000

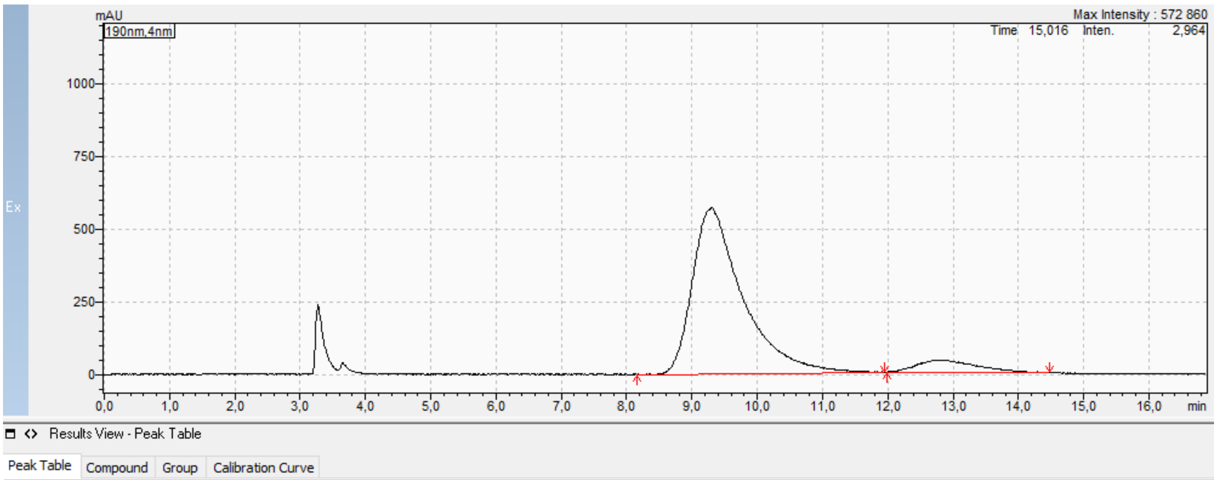

Peak#	Ret. Time	Area	Height	Peak Start	Peak End	Mark	Conc.	Unit	Area%
1	9,289	30330912	570661	8,149	11,957	M	91,799		91,799
2	12,844	2709564	42157	11,979	14,475	M	8,201		8,201
Total		33040477	612818				100,000		100,000

for **3a**: *er* = 92:8 *er* (*ee* = 84%)

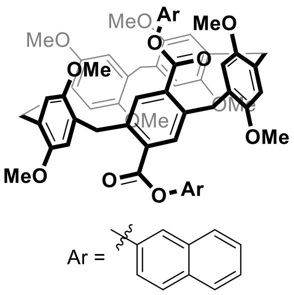

Conditions: Chiralpak IC column
mobile phase: *n*-heptane/propan-2-ol – 70:30
λ = 190 nm, V = 1.0 ml/min, t = 25 °C
for **3a**: t_R = 9.3 min (minor), t_R = 12.8 min (major).

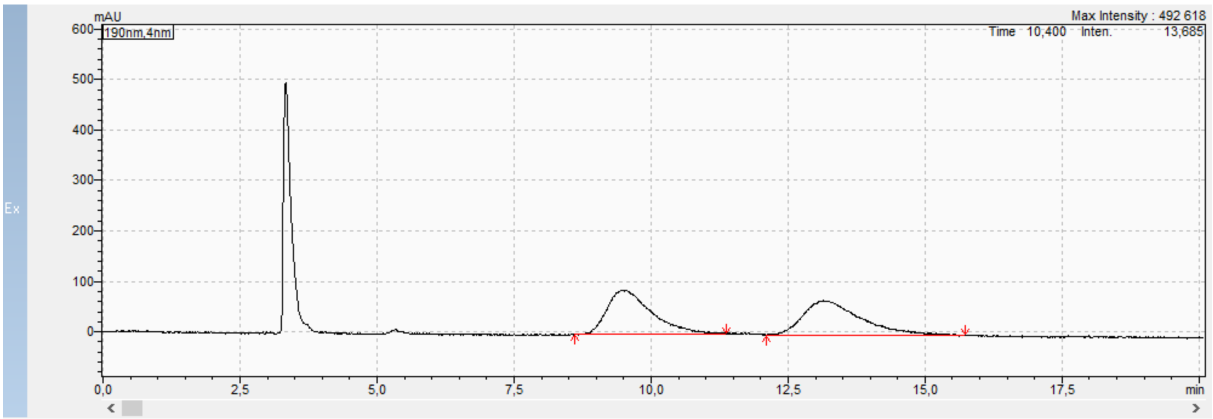

Results View - Peak Table

Peak Table | Compound | Group | Calibration Curve

Peak#	Ret. Time	Area	Height	Peak Start	Peak End	Mark	Conc.	Unit	Area%
1	9,513	4966015	88170	8,608	11,371	M	50,104		50,104
2	13,152	4945302	68228	12,107	15,733	M	49,896		49,896
Total		9911316	156398				100,000		100,000

Mother liquor from crystalization (er = 92:8)

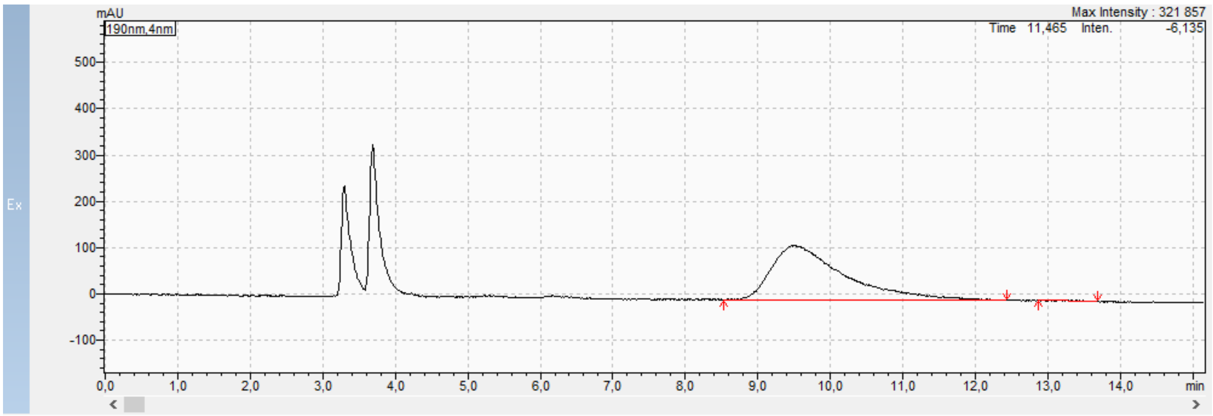

Results View - Peak Table

Peak Table | Compound | Group | Calibration Curve

Peak#	Ret. Time	Area	Height	Peak Start	Peak End	Mark	Conc.	Unit	Area%
1	9,467	8071684	117646	8,533	12,427	M	99,945		99,945
2	13,385	4464	2012	12,864	13,685	M	0,055		0,055
Total		8076148	119658				100,000		100,000

for **3a**: *er* ≥ 99.5:0.5 *er* (*ee* = 99.5%)

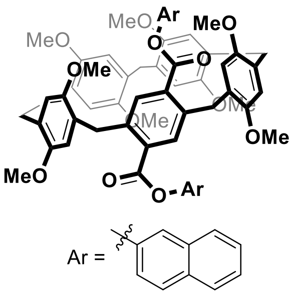

Conditions: Chiralpak IC column
mobile phase: *n*-heptane/propan-2-ol – 70:30
λ = 190 nm, V = 1.0 ml/min, t = 25 °C
for **3a**: t_R = 9.3 min (minor), t_R = 12.8 min (major).

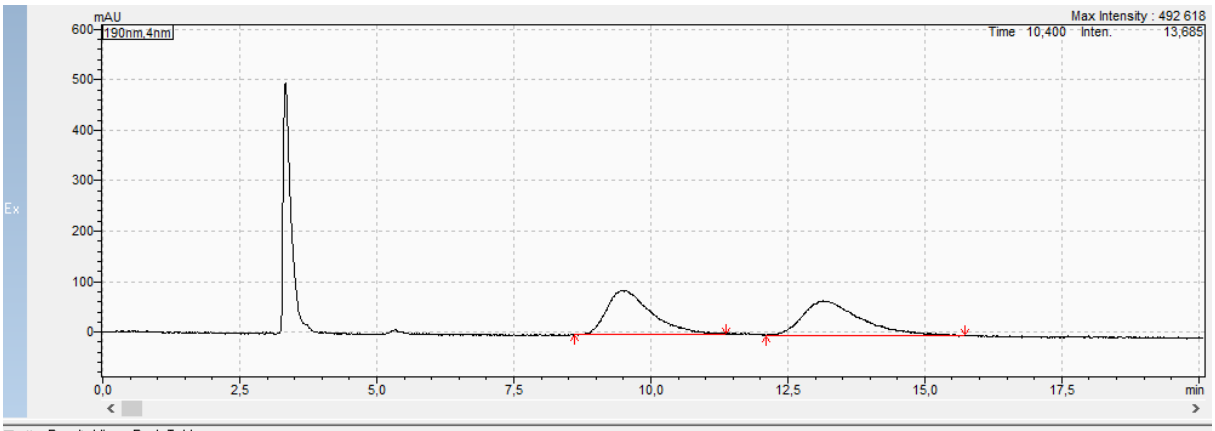

Results View - Peak Table

Peak Table | Compound | Group | Calibration Curve

Peak#	Ret. Time	Area	Height	Peak Start	Peak End	Mark	Conc.	Unit	Area%
1	9,513	4966015	88170	8,608	11,371	M	50,104		50,104
2	13,152	4945302	68228	12,107	15,733	M	49,896		49,896
Total		9911316	156398				100,000		100,000

Mother liquor from crystalization (er = 89:11)

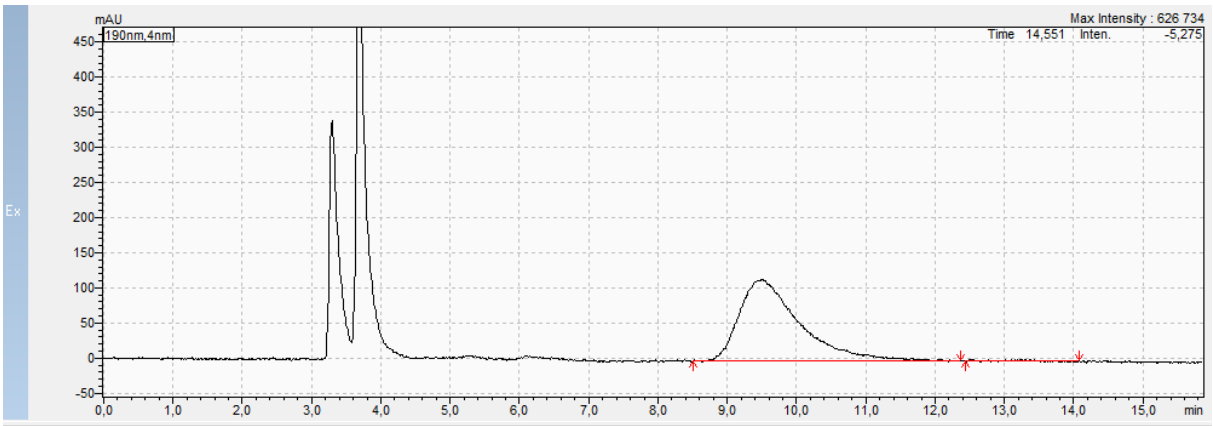

Results View - Peak Table

Peak Table | Compound | Group | Calibration Curve

Peak#	Ret. Time	Area	Height	Peak Start	Peak End	Mark	Conc.	Unit	Area%
1	9,526	6845557	114777	8,501	12,373	M	99,638		99,638
2	12,501	24847	2543	12,437	14,080	M	0,362		0,362
Total		6870404	117320				100,000		100,000

for **3a**: *er* = 99.5:0.5 *er* (*ee* = 99%)

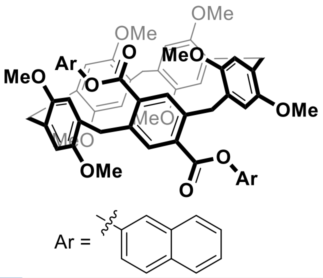

Conditions: Chiralpak IC column
mobile phase: *n*-heptane/propan-2-ol – 70:30
λ = 190 nm, V = 1.0 ml/min, t = 25 °C
for *ent*-**3a**: t_R = 9.3 min (minor), t_R = 12.8 min (major).

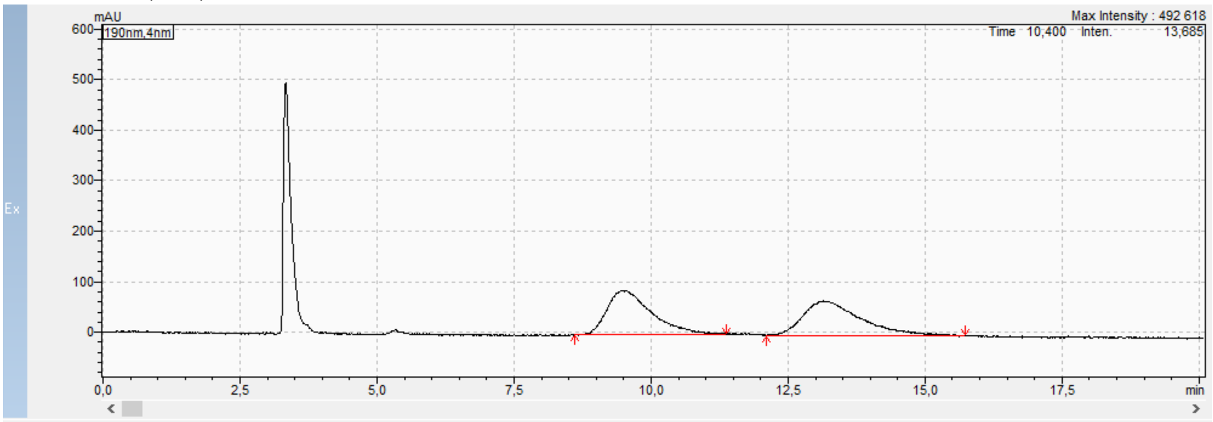

Results View - Peak Table

Peak Table | Compound | Group | Calibration Curve

Peak#	Ret. Time	Area	Height	Peak Start	Peak End	Mark	Conc.	Unit	Area%
1	9,513	4966015	88170	8,608	11,371	M	50,104		50,104
2	13,152	4945302	68228	12,107	15,733	M	49,896		49,896
Total		9911316	156398				100,000		100,000

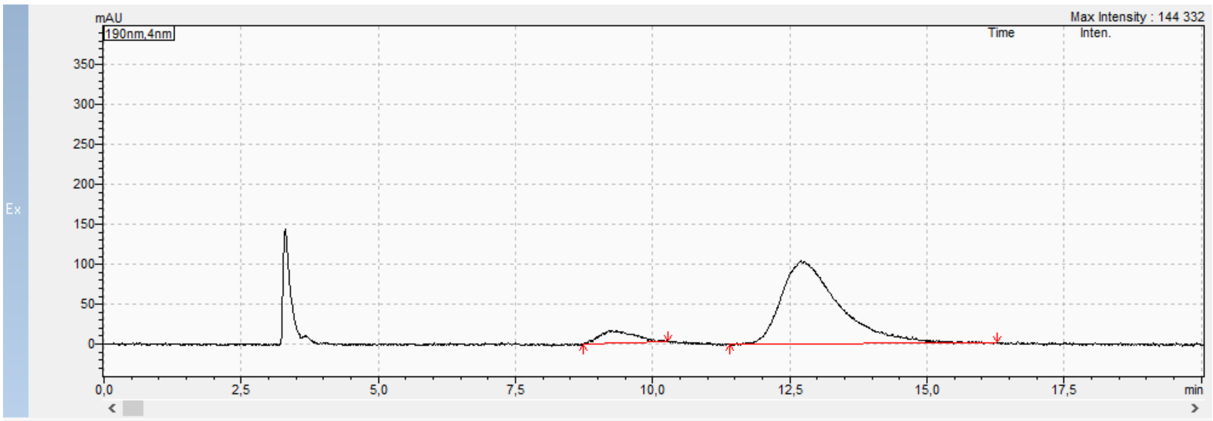

Results View - Peak Table

Peak Table | Compound | Group | Calibration Curve

Peak#	Ret. Time	Area	Height	Peak Start	Peak End	Mark	Conc.	Unit	Area%
1	9,267	697187	15891	8,736	10,272	M	8,310		8,310
2	12,706	7692886	104918	11,413	16,288	M	91,690		91,690
Total		8390073	120809				100,000		100,000

for *ent*-**3a**: *er* = 92:8 *er* (*ee* = 83%)

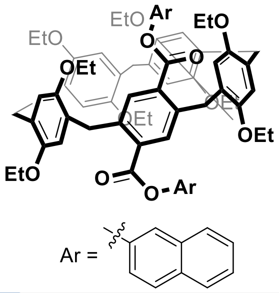

Conditions: Chiralpak IC column
mobile phase: *n*-heptane/propan-2-ol – 70:30
λ = 190 nm, V = 1.0 ml/min, t = 25 °C
for **3b**: t_R = 4.6 min (major), t_R = 6.5 min (minor).

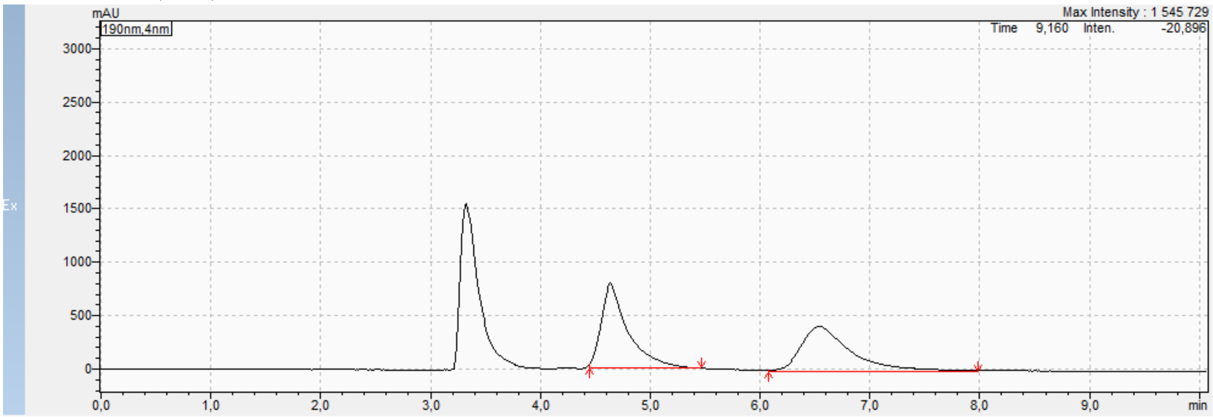

Results View - Peak Table

Peak Table | Compound | Group | Calibration Curve

Peak#	Ret. Time	Area	Height	Peak Start	Peak End	Mark	Conc.	Unit	Area%
1	4,641	13622909	798714	4,448	5,472	M	50,169		50,169
2	6,531	13531328	425894	6,080	7,989	M	49,831		49,831
Total		27154237	1224608				100,000		100,000

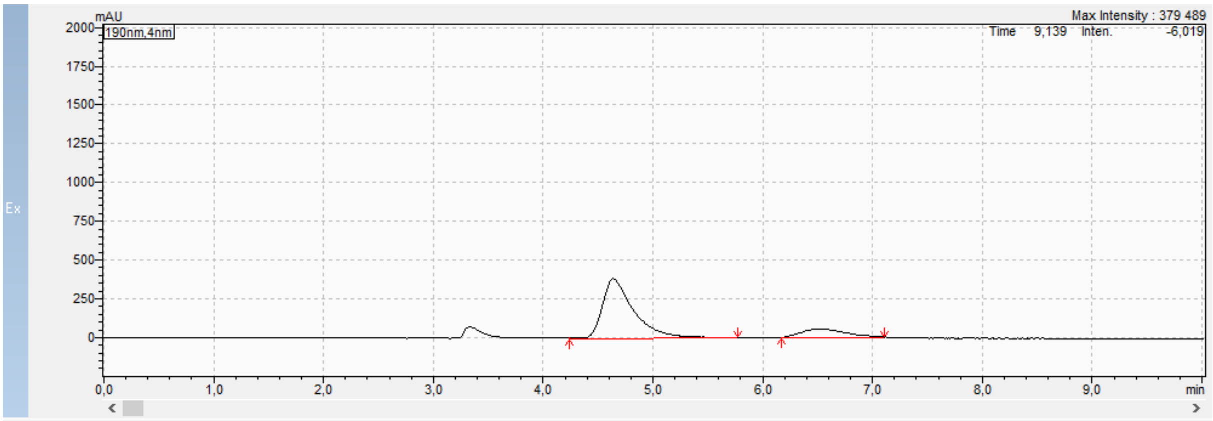

Results View - Peak Table

Peak Table | Compound | Group | Calibration Curve

Peak#	Ret. Time	Area	Height	Peak Start	Peak End	Mark	Conc.	Unit	Area%
1	4,642	7896786	385769	4,235	5,771	M	84,142		84,142
2	6,518	1488256	55730	6,165	7,104	M	15,858		15,858
Total		9385042	441499				100,000		100,000

for **3b**: *er* = 84:16 *er* (*ee* = 68%)

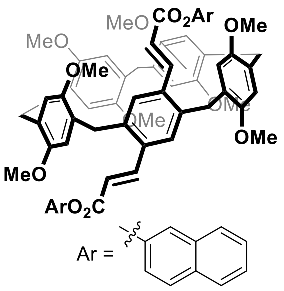

Conditions: Chiralpak IA column
mobile phase: *n*-heptane/propan-2-ol – 90:10
λ = 222 nm, V = 1.0 ml/min, t = 25 °C
for **3c**: t_R = 23.0 min (major), t_R = 30.8 min (minor).

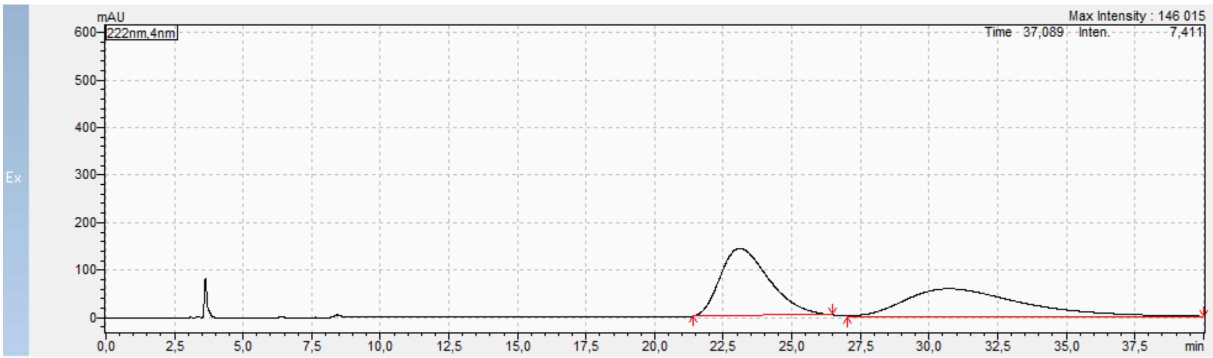

Results View - Peak Table

Peak Table | Compound | Group | Calibration Curve

Peak#	Ret. Time	Area	Height	Peak Start	Peak End	Mark	Conc.	Unit	Area%
1	23,111	17512925	141718	21,397	26,475	M	50,270		50,270
2	30,758	17325146	58731	27,029	39,989		49,730		49,730
Total		34838071	200449				100,000		100,000

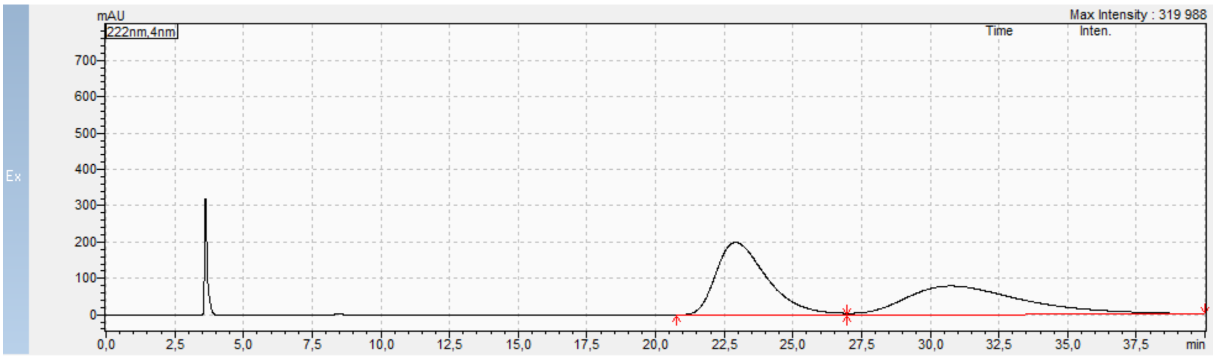

Results View - Peak Table

Peak Table | Compound | Group | Calibration Curve

Peak#	Ret. Time	Area	Height	Peak Start	Peak End	Mark	Conc.	Unit	Area%
1	22,920	25640804	199510	20,747	26,987		52,564		52,564
2	30,797	23139602	78104	26,987	40,000	V	47,436		47,436
Total		48780406	277614				100,000		100,000

for **3c**: *er* = 53:47 *er* (*ee* = 5%)

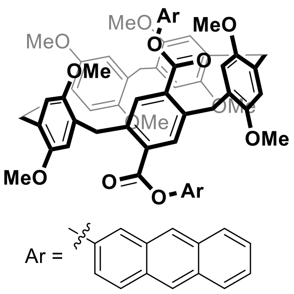

Conditions: Chiralpak IC column
mobile phase: *n*-heptane/propan-2-ol – 70:30
λ = 190 nm, V = 1.0 ml/min, t = 25 °C
for **3d**: t_R = 16.3 min (major), t_R = 25.3 min (minor).

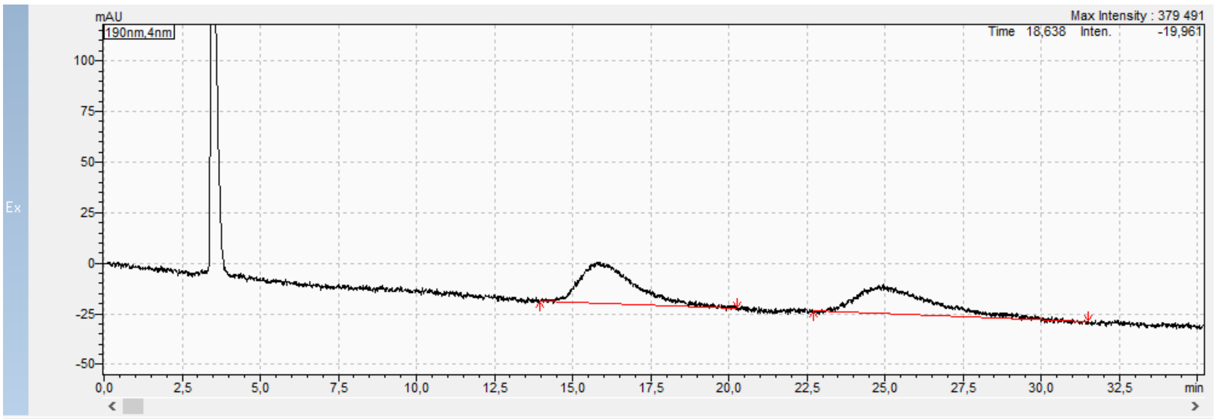

Results View - Peak Table

Peak Table | Compound | Group | Calibration Curve

Peak#	Ret. Time	Area	Height	Peak Start	Peak End	Mark	Conc.	Unit	Area%
1	15,768	2451419	20296	13,963	20,256	M	51,240		51,240
2	24,948	2332811	14599	22,709	31,509	M	48,760		48,760
Total		4784230	34896				100,000		100,000

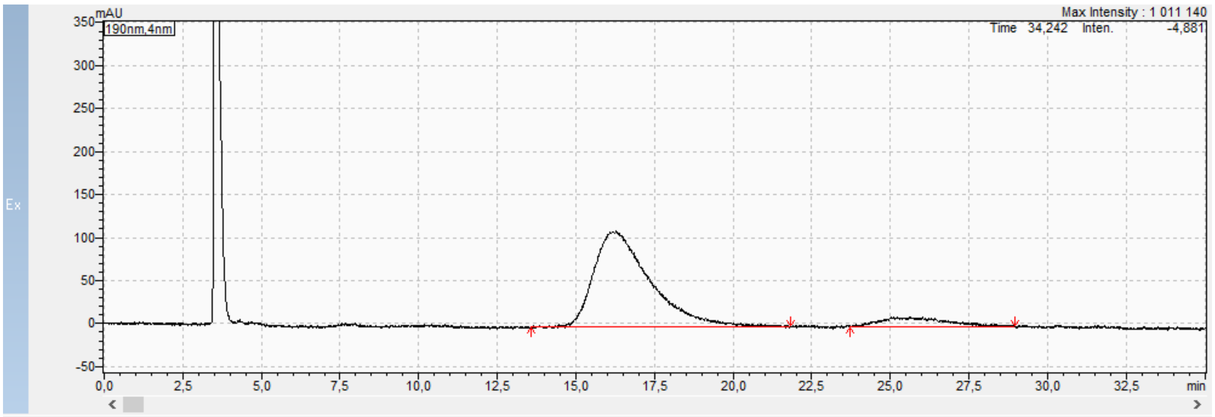

Results View - Peak Table

Peak Table | Compound | Group | Calibration Curve

Peak#	Ret. Time	Area	Height	Peak Start	Peak End	Mark	Conc.	Unit	Area%
1	16,267	13926631	111668	13,557	21,813	M	89,969		89,969
2	25,229	1552764	11179	23,733	28,960	M	10,031		10,031
Total		15479395	122846				100,000		100,000

for **3d**: *er* = 90:10 *er* (*ee* = 80%)

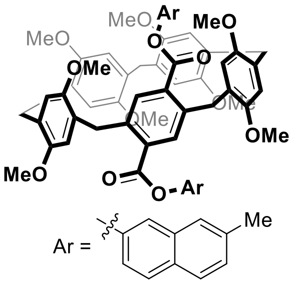

Conditions: Chiralpak IC column
mobile phase: *n*-heptane/propan-2-ol – 70:30
λ = 190 nm, *V* = 1.0 ml/min, *t* = 25 °C
for **3e**: t_R = 8.9 min (major), t_R = 15.3 min (minor).

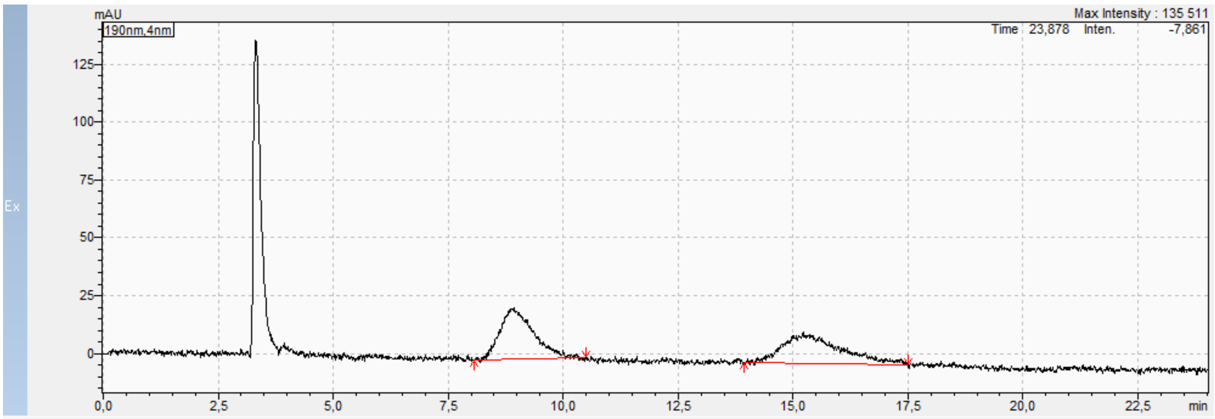

Results View - Peak Table

Peak Table | Compound | Group | Calibration Curve

Peak#	Ret. Time	Area	Height	Peak Start	Peak End	Mark	Conc.	Unit	Area%
1	8,938	1115972	22213	8,064	10,496	M	49,291		49,291
2	15,218	1148071	13628	13,941	17,504	M	50,709		50,709
Total		2264042	35841				100,000		100,000

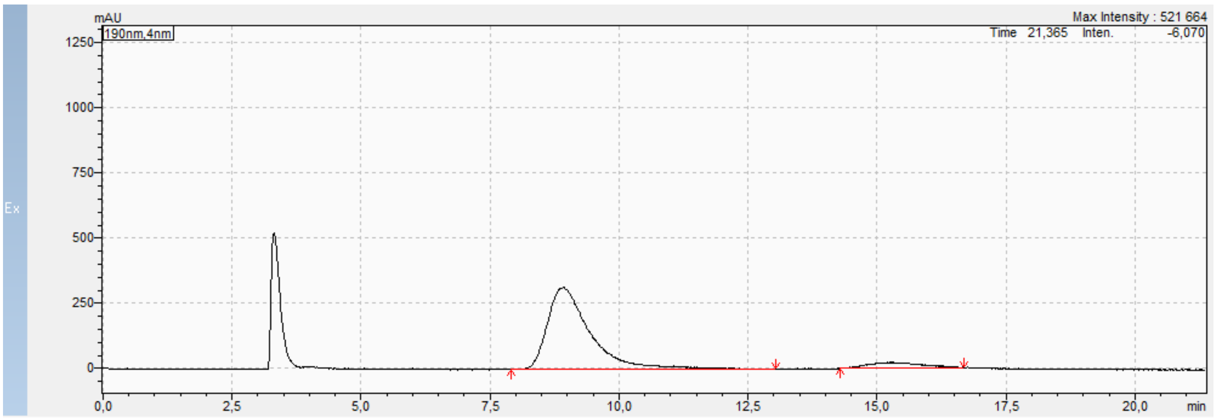

Results View - Peak Table

Peak Table | Compound | Group | Calibration Curve

Peak#	Ret. Time	Area	Height	Peak Start	Peak End	Mark	Conc.	Unit	Area%
1	8,926	17928149	315182	7,904	13,035	M	91,731		91,731
2	15,260	1616203	22378	14,272	16,672	M	8,269		8,269
Total		19544352	337561				100,000		100,000

for **3e**: *er* = 92:8 *er* (*ee* = 84%)

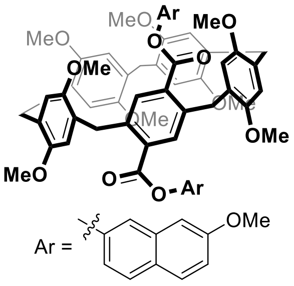

Conditions: Chiralpak IC column
mobile phase: *n*-heptane/propan-2-ol – 70:30
λ = 190 nm, V = 1.0 ml/min, t = 25 °C
for **3f**: t_R = 18.4 min (major), t_R = 27.0 min (minor).

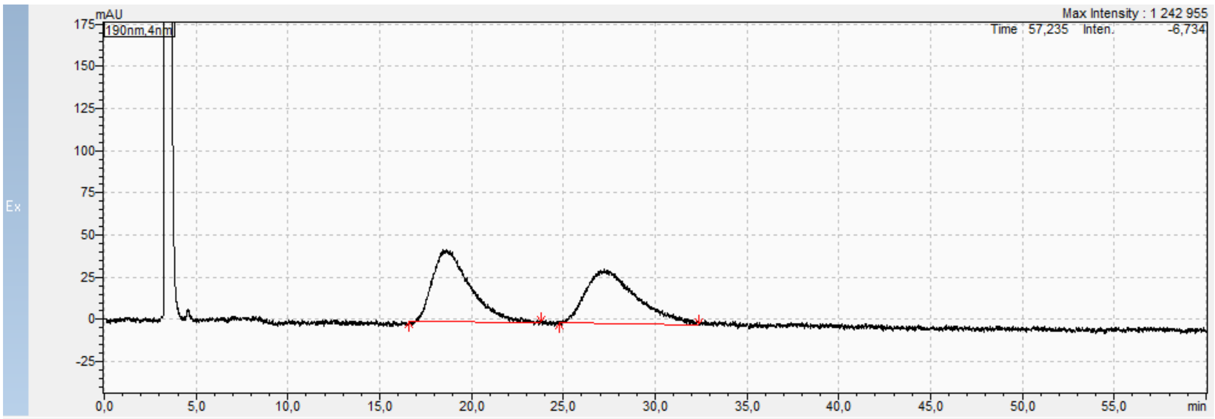

Results View - Peak Table

Peak Table | Compound | Group | Calibration Curve

Peak#	Ret. Time	Area	Height	Peak Start	Peak End	Mark	Conc.	Unit	Area%
1	18,656	6114920	43023	16,587	23,797	M	50,726		50,726
2	27,215	5939965	31721	24,789	32,384	M	49,274		49,274
Total		12054884	74744				100,000		100,000

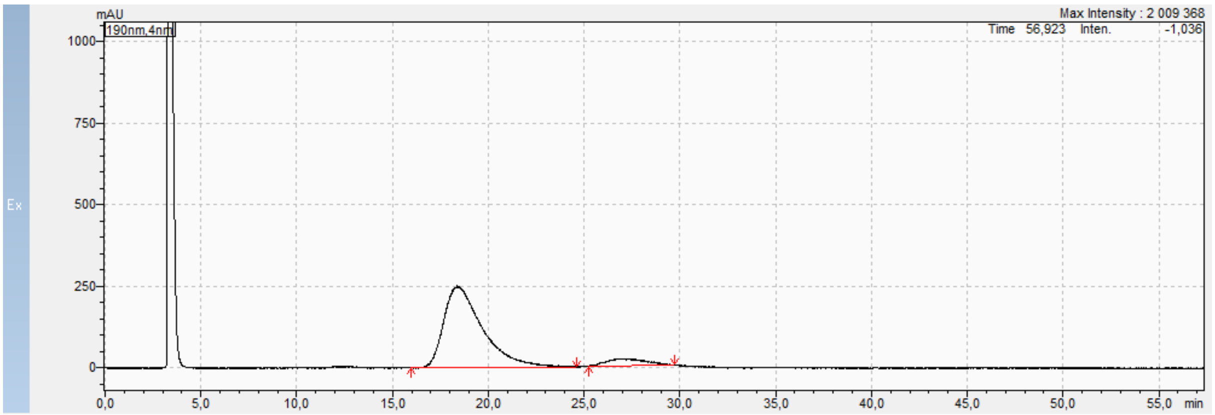

Results View - Peak Table

Peak Table | Compound | Group | Calibration Curve

Peak#	Ret. Time	Area	Height	Peak Start	Peak End	Mark	Conc.	Unit	Area%
1	18,381	34541527	249133	15,968	24,608	M	91,811		91,811
2	26,952	3080801	22410	25,248	29,728	M	8,189		8,189
Total		37622329	271542				100,000		100,000

for **3f**: *er* = 92:8 *er* (*ee* = 84%)

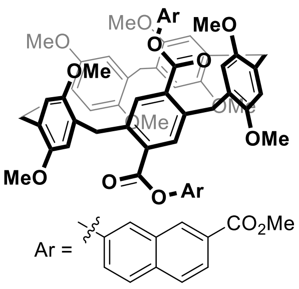

Conditions: Chiralpak IG column
mobile phase: *n*-heptane/propan-2-ol – 50:50
λ = 220 nm, V = 1.0 ml/min, t = 25 °C
for **3g**: t_R = 52.2 min (major), t_R = 101.1 min (minor).

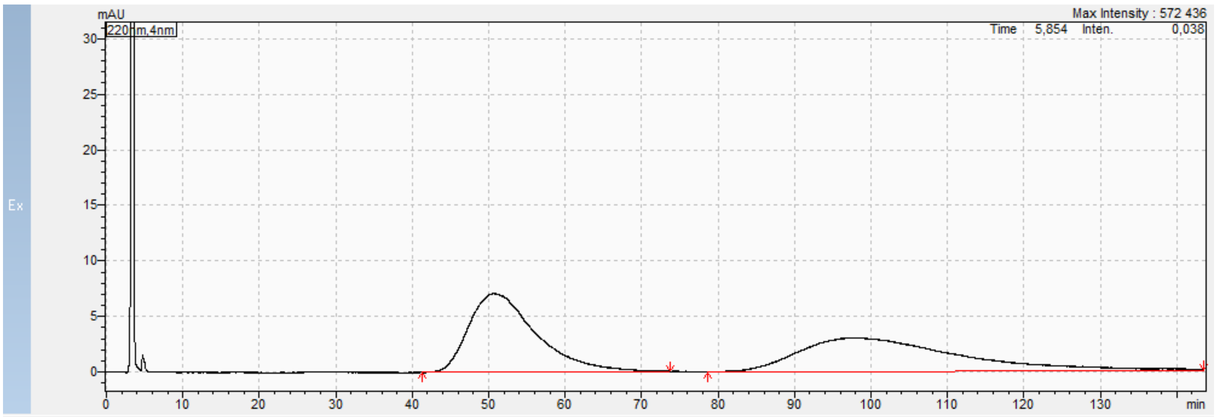

Results View - Peak Table

Peak Table | Compound | Group | Calibration Curve

Peak#	Ret. Time	Area	Height	Peak Start	Peak End	Mark	Conc.	Unit	Area%
1	50,739	4332428	7079	41,301	73,728	M	49,790		49,790
2	97,834	4368911	3026	78,635	143,509	M	50,210		50,210
Total		8701339	10105				100,000		100,000

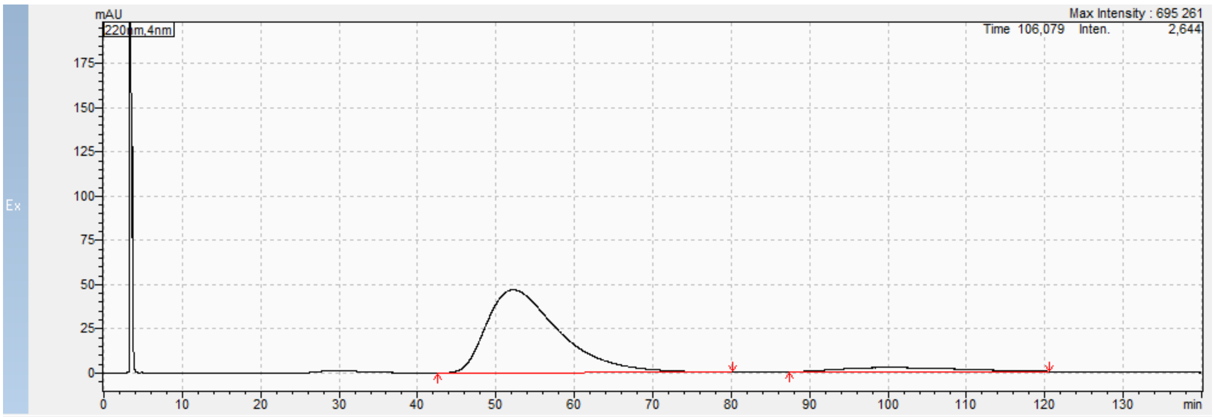

Results View - Peak Table

Peak Table | Compound | Group | Calibration Curve

Peak#	Ret. Time	Area	Height	Peak Start	Peak End	Mark	Conc.	Unit	Area%
1	52,236	30203200	46937	42,560	80,181	M	92,129		92,129
2	101,100	2580279	2370	87,424	120,576	M	7,871		7,871
Total		32783478	49307				100,000		100,000

for **3g**: *er* = 92:8 *er* (*ee* = 84%)

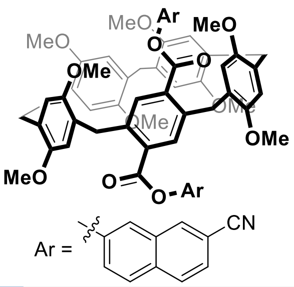

Conditions: Chiralpak IC column
mobile phase: *n*-heptane/propan-2-ol – 40:60
λ = 220 nm, V = 1.0 ml/min, t = 35 °C
for **3h**: t_R = 79.0 min (major), t_R = 142.1 min (minor).

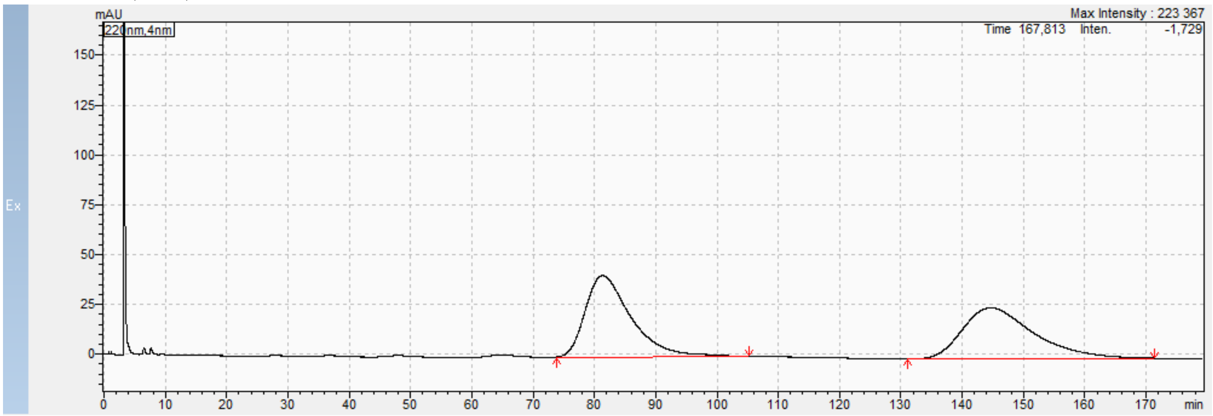

Results View - Peak Table

Peak Table | Compound | Group | Calibration Curve

Peak#	Ret. Time	Area	Height	Peak Start	Peak End	Mark	Conc.	Unit	Area%
1	81,351	21299416	40788	73,749	105,323	M	50,815		50,815
2	144,618	20616413	25511	131,019	171,424	M	49,185		49,185
Total		41915829	66299				100,000		100,000

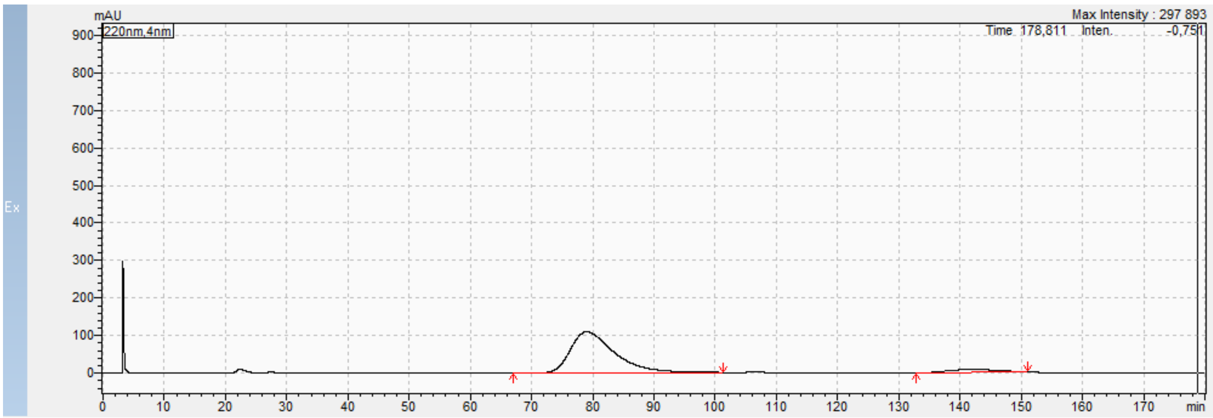

Results View - Peak Table

Peak Table | Compound | Group | Calibration Curve

Peak#	Ret. Time	Area	Height	Peak Start	Peak End	Mark	Conc.	Unit	Area%
1	79,020	54004161	109041	67,008	101,301	M	91,651		91,651
2	142,114	4919395	8345	132,736	151,051	M	8,349		8,349
Total		58923556	117386				100,000		100,000

for **3h**: *er* = 92:8 *er* (*ee* = 83%)

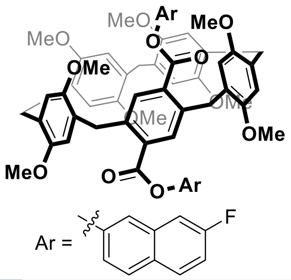

Conditions: Chiralpak IC column
mobile phase: *n*-heptane/propan-2-ol – 70:30
λ = 190 nm, V = 1.0 ml/min, t = 25 °C
for **3i**: t_R = 9.9 min (major), t_R = 14.2 min (minor).

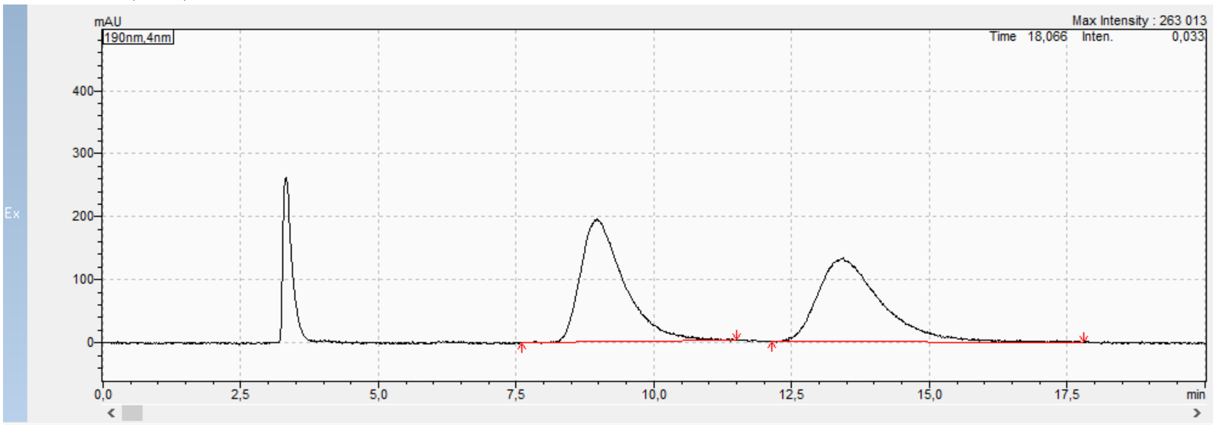

Results View - Peak Table

Peak Table | Compound | Group | Calibration Curve

Peak#	Ret. Time	Area	Height	Peak Start	Peak End	Mark	Conc.	Unit	Area%
1	8,969	10693799	194999	7,595	11,499	M	50,397		50,397
2	13,428	10525165	133138	12,149	17,792	M	49,603		49,603
Total		21218965	328137				100,000		100,000

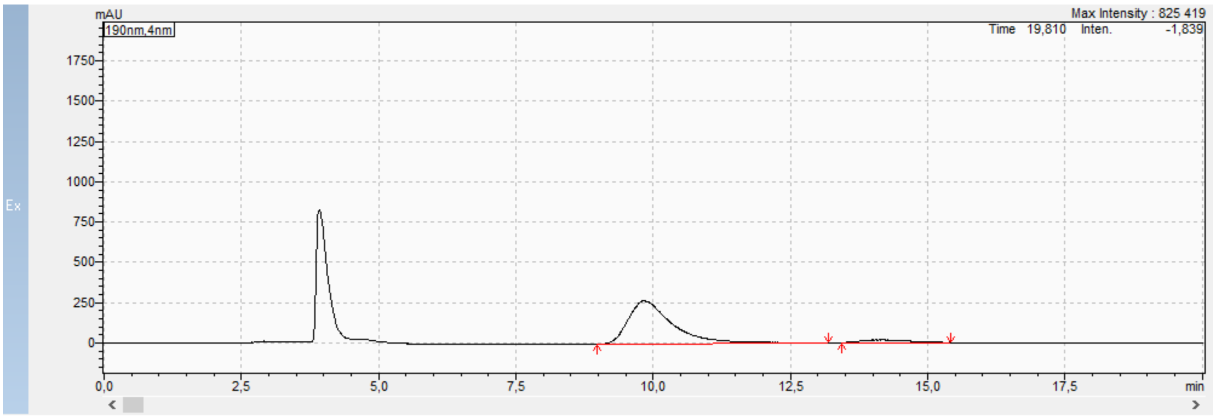

Results View - Peak Table

Peak Table | Compound | Group | Calibration Curve

Peak#	Ret. Time	Area	Height	Peak Start	Peak End	Mark	Conc.	Unit	Area%
1	9,848	15481283	267152	8,971	13,184	M	93,642		93,642
2	14,181	1051084	18333	13,429	15,413	M	6,358		6,358
Total		16532367	285485				100,000		100,000

for **3i**: *er* = 93:7 *er* (*ee* = 87%)

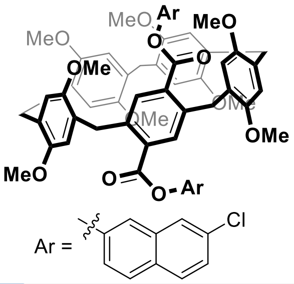

Conditions: Chiralpak IC column
mobile phase: *n*-heptane/propan-2-ol – 70:30
λ = 228 nm, V = 1.0 ml/min, t = 25 °C
for **3j**: t_R = 8.9 min (major), t_R = 13.9 min (minor).

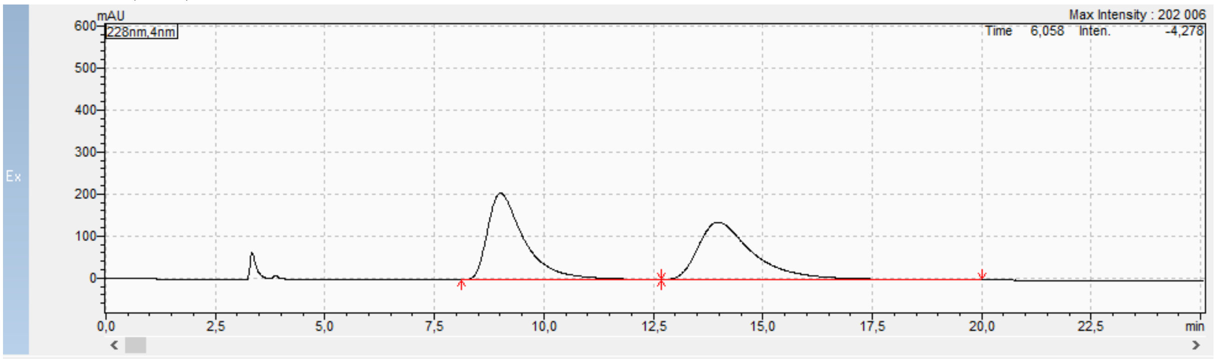

Results View - Peak Table

Peak Table | Compound | Group | Calibration Curve

Peak#	Ret. Time	Area	Height	Peak Start	Peak End	Mark	Conc.	Unit	Area%
1	9,010	12024074	205714	8,117	12,693	M	49,968		49,968
2	13,979	12039464	136635	12,693	20,011	M	50,032		50,032
Total		24063539	342349				100,000		100,000

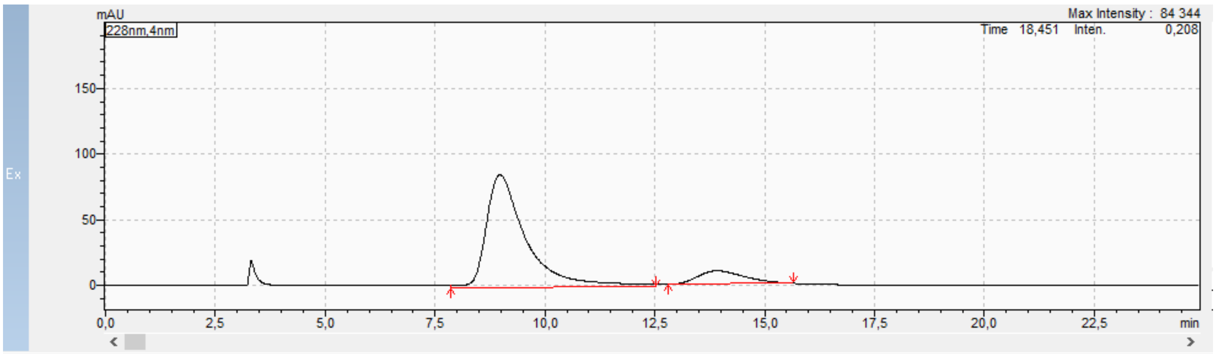

Results View - Peak Table

Peak Table | Compound | Group | Calibration Curve

Peak#	Ret. Time	Area	Height	Peak Start	Peak End	Mark	Conc.	Unit	Area%
1	8,977	5364295	85547	7,861	12,512	M	87,809		87,809
2	13,931	744782	10017	12,811	15,648	M	12,191		12,191
Total		6109077	95564				100,000		100,000

for **3j**: *er* = 88:12 *er* (*ee* = 76%)

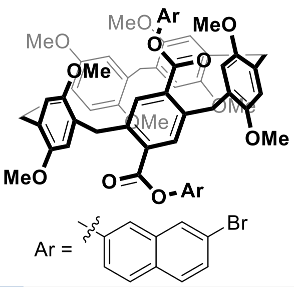

Conditions: Chiralpak IC column
mobile phase: *n*-heptane/propan-2-ol – 70:30
λ = 190 nm, V = 1.0 ml/min, t = 25 °C
for **3k**: t_R = 9.8 min (major), t_R = 16.0 min (minor).

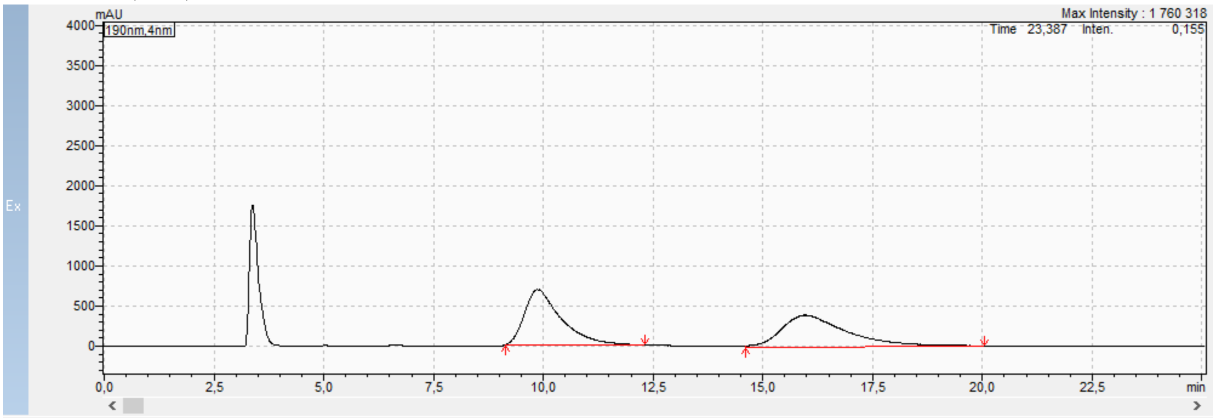

Results View - Peak Table

Peak Table | Compound | Group | Calibration Curve

Peak#	Ret. Time	Area	Height	Peak Start	Peak End	Mark	Conc.	Unit	Area%
1	9,860	41460895	699455	9,152	12,320	M	49,669		49,669
2	15,975	42014065	398741	14,603	20,053	M	50,331		50,331
Total		83474960	1098197				100,000		100,000

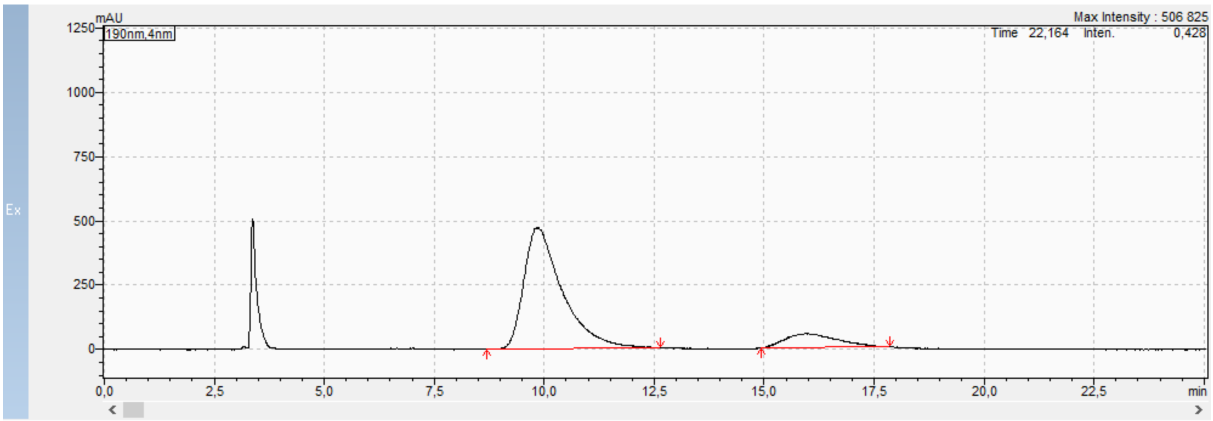

Results View - Peak Table

Peak Table | Compound | Group | Calibration Curve

Peak#	Ret. Time	Area	Height	Peak Start	Peak End	Mark	Conc.	Unit	Area%
1	9,839	28524611	469755	8,683	12,640	M	86,748		86,748
2	15,999	4357569	53789	14,944	17,856	M	13,252		13,252
Total		32882179	523544				100,000		100,000

for **3k**: *er* = 87:13 *er* (*ee* = 74%)

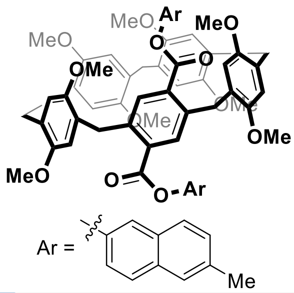

Conditions: Chiralpak IC column
mobile phase: *n*-heptane/propan-2-ol – 70:30
λ = 190 nm, V = 1.0 ml/min, t = 25 °C
for **3l**: t_R = 10.4 min (major), t_R = 14.2 min (minor).

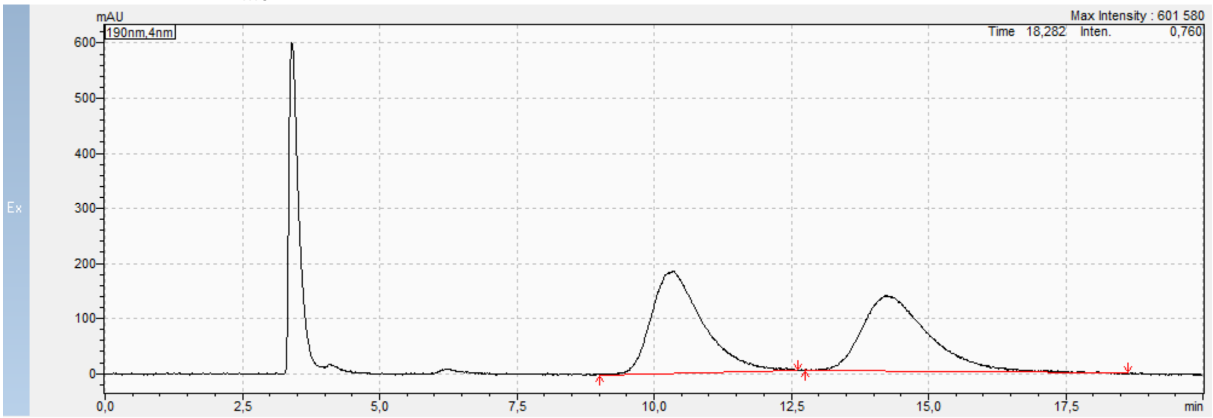

Results View - Peak Table

Peak Table | Compound | Group | Calibration Curve

Peak#	Ret. Time	Area	Height	Peak Start	Peak End	Mark	Conc.	Unit	Area%
1	10,355	11851000	184690	9,013	12,619	M	50,126		50,126
2	14,242	11791491	138107	12,757	18,635	M	49,874		49,874
Total		23642491	322797				100,000		100,000

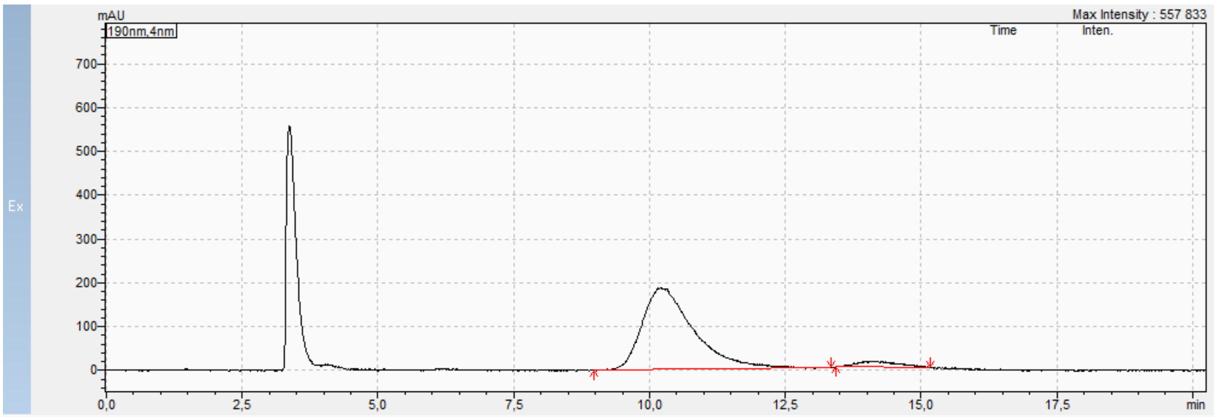

Results View - Peak Table

Peak Table | Compound | Group | Calibration Curve

Peak#	Ret. Time	Area	Height	Peak Start	Peak End	Mark	Conc.	Unit	Area%
1	10,201	12259219	186134	8,981	13,344	M	94,655		94,655
2	14,187	692262	13021	13,429	15,179	M	5,345		5,345
Total		12951481	199156				100,000		100,000

for **3l**: *er* = 95:5 *er* (*ee* = 89%)

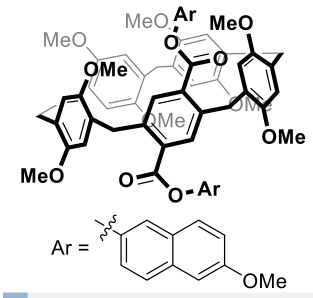

Conditions: Chiralpak IC column
mobile phase: *n*-heptane/propan-2-ol – 70:30
λ = 228 nm, V = 1.0 ml/min, t = 25 °C
for **3m**: t_R = 19.8 min (major), t_R = 33.7 min (minor).

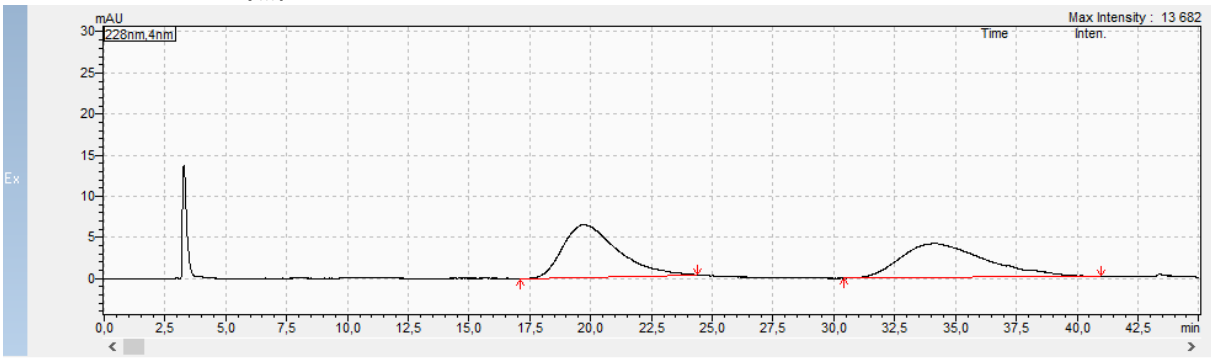

Results View - Peak Table

Peak Table | Compound | Group | Calibration Curve

Peak#	Ret. Time	Area	Height	Peak Start	Peak End	Mark	Conc.	Unit	Area%
1	19,704	998299	6381	17,120	24,405	M	49,169		49,169
2	34,174	1032047	4173	30,400	40,992	M	50,831		50,831
Total		2030346	10554				100,000		100,000

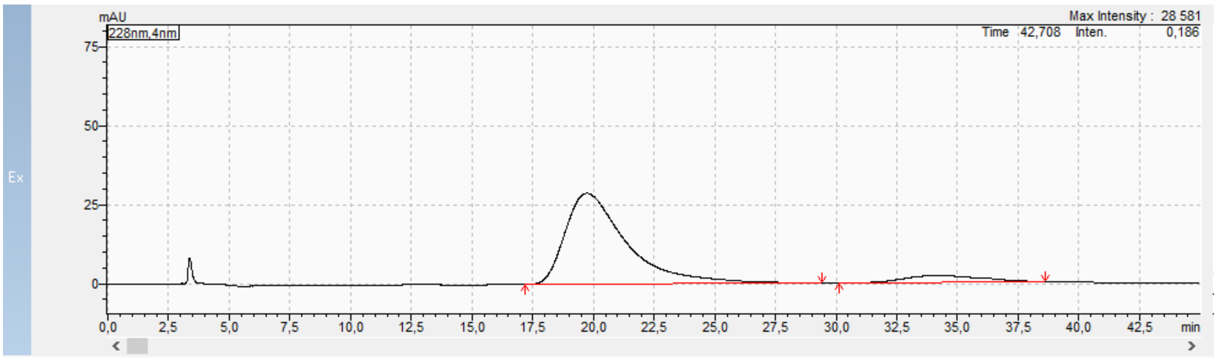

Results View - Peak Table

Peak Table | Compound | Group | Calibration Curve

Peak#	Ret. Time	Area	Height	Peak Start	Peak End	Mark	Conc.	Unit	Area%
1	19,740	5030399	28697	17,205	29,419	M	91,099		91,099
2	34,146	491495	2173	30,123	38,613	M	8,901		8,901
Total		5521894	30869				100,000		100,000

for **3m**: *er* = 91:9 *er* (*ee* = 82%)

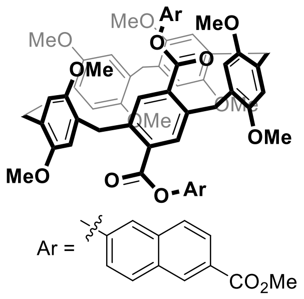

Conditions: Chiralpak IG column
mobile phase: *n*-heptane/propan-2-ol – 50:50
λ = 223 nm, V = 1.0 ml/min, t = 25 °C
for **3n**: t_R = 40.6 min (major), t_R = 62.3 min (minor).

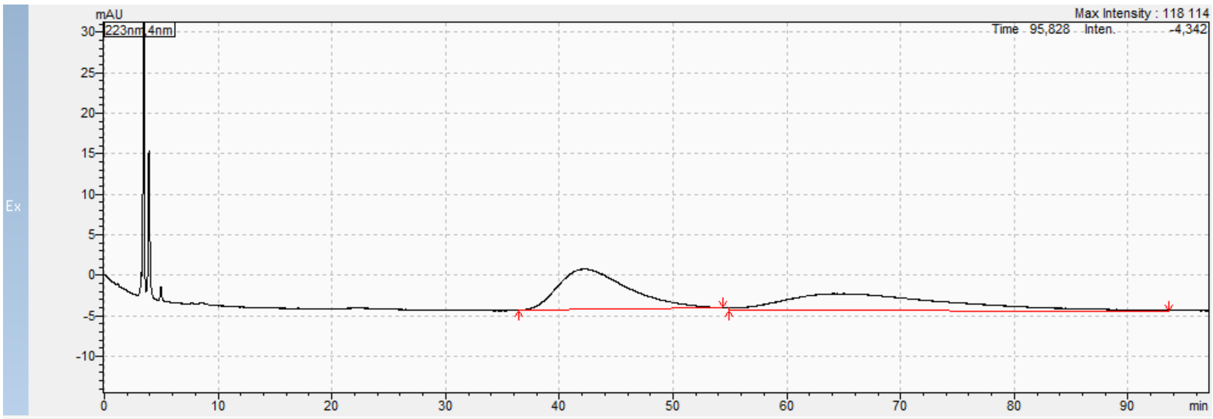

Results View - Peak Table

Peak Table | Compound | Group | Calibration Curve

Peak#	Ret. Time	Area	Height	Peak Start	Peak End	Mark	Conc.	Unit	Area%
1	42,349	2152937	5007	36,459	54,368	M	50,909		50,909
2	64,065	2076080	2053	54,901	93,589	M	49,091		49,091
Total		4229017	7060				100,000		100,000

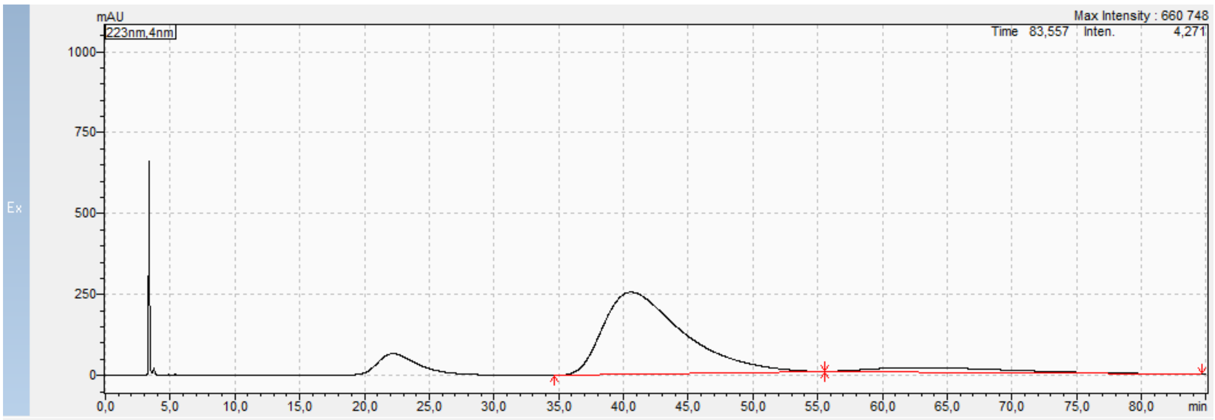

Results View - Peak Table

Peak Table | Compound | Group | Calibration Curve

Peak#	Ret. Time	Area	Height	Peak Start	Peak End	Mark	Conc.	Unit	Area%
1	40,610	108700114	253218	34,677	55,520	M	91,055		91,055
2	62,877	10678478	14116	55,520	84,683	M	8,945		8,945
Total		119378591	267334				100,000		100,000

for **3n**: *er* = 91:9 (*ee* = 82%)

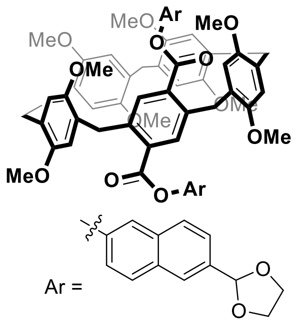

Conditions: Chiralpak IC column
mobile phase: *n*-heptane/propan-2-ol – 70:30
λ = 220 nm, V = 1.0 ml/min, t = 25 °C
for **3o**: t_R = 42.4 min (major), t_R = 89.9 min (minor).

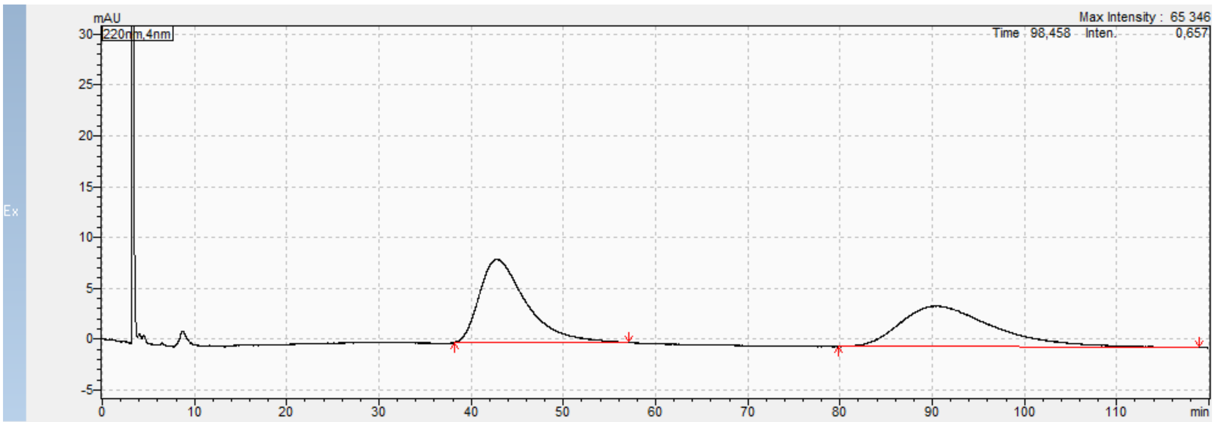

Results View - Peak Table

Peak Table | Compound | Group | Calibration Curve

Peak#	Ret. Time	Area	Height	Peak Start	Peak End	Mark	Conc.	Unit	Area%
1	42,765	2900908	8220	38,197	57,088	M	50,331		50,331
2	90,546	2862712	4005	79,829	118,944	M	49,669		49,669
Total		5763620	12226				100,000		100,000

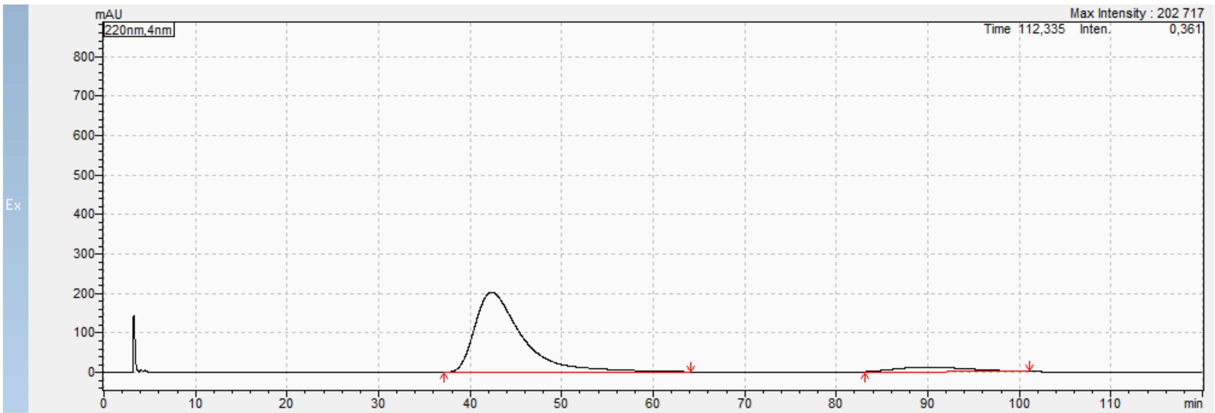

Results View - Peak Table

Peak Table | Compound | Group | Calibration Curve

Peak#	Ret. Time	Area	Height	Peak Start	Peak End	Mark	Conc.	Unit	Area%
1	42,362	72356058	202149	37,141	64,096	M	91,869		91,869
2	89,879	6403578	11418	83,125	101,099	M	8,131		8,131
Total		78759636	213566				100,000		100,000

for **3o**: *er* = 92:8 (*ee* = 84%)

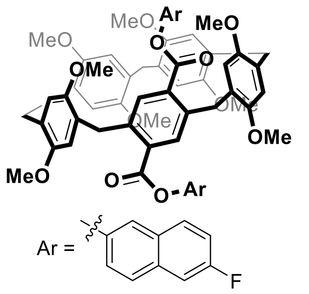

Conditions: Chiralpak IC column
mobile phase: *n*-heptane/propan-2-ol – 70:30
λ = 190 nm, V = 1.0 ml/min, t = 25 °C
for **3p**: t_R = 11.9 min (major), t_R = 23.6 min (minor).

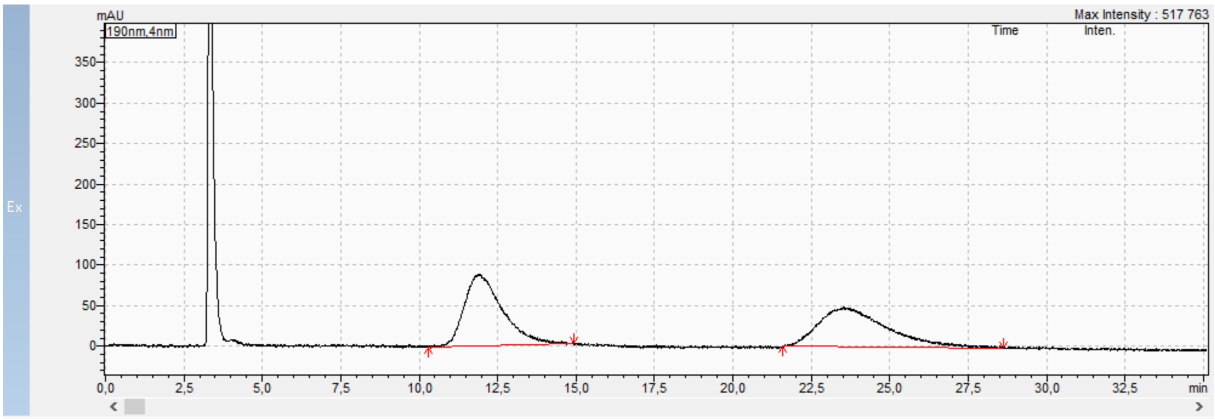

Results View - Peak Table

Peak Table | Compound | Group | Calibration Curve

Peak#	Ret. Time	Area	Height	Peak Start	Peak End	Mark	Conc.	Unit	Area%
1	11,934	7459350	88308	10,304	14,933	M	50,119		50,119
2	23,517	7423799	48906	21,568	28,619	M	49,881		49,881
Total		14883150	137214				100,000		100,000

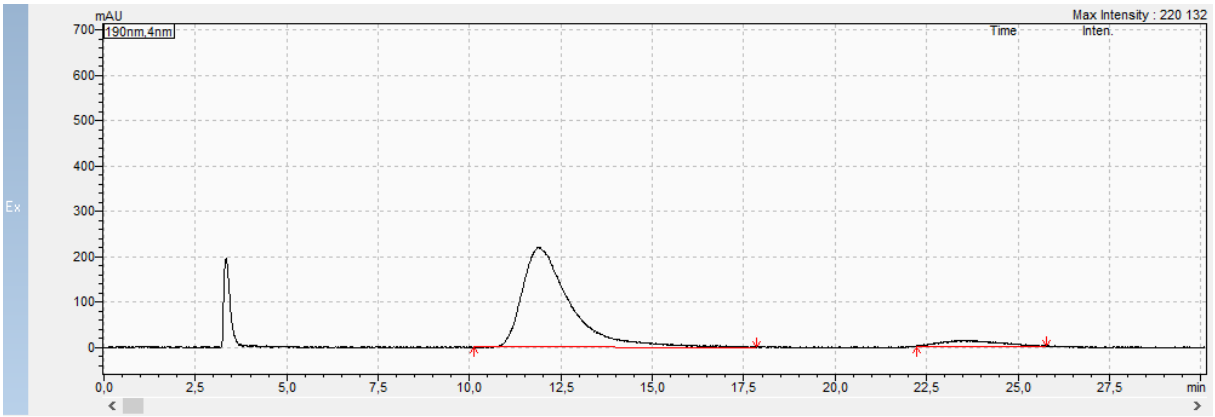

Results View - Peak Table

Peak Table | Compound | Group | Calibration Curve

Peak#	Ret. Time	Area	Height	Peak Start	Peak End	Mark	Conc.	Unit	Area%
1	11,858	19766610	219731	10,133	17,856	M	93,152		93,152
2	23,552	1453203	13585	22,219	25,781	M	6,848		6,848
Total		21219813	233316				100,000		100,000

for **3p**: *er* = 93:7 *er* (*ee* = 86%)

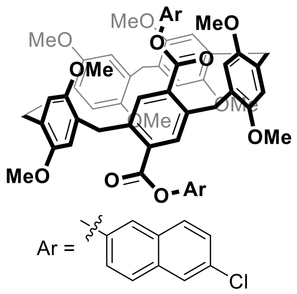

Conditions: Chiralpak IC column
mobile phase: *n*-heptane/propan-2-ol – 70:30
λ = 190 nm, V = 1.0 ml/min, t = 25 °C
for **3q**: t_R = 11.6 min (major), t_R = 19.3 min (minor).

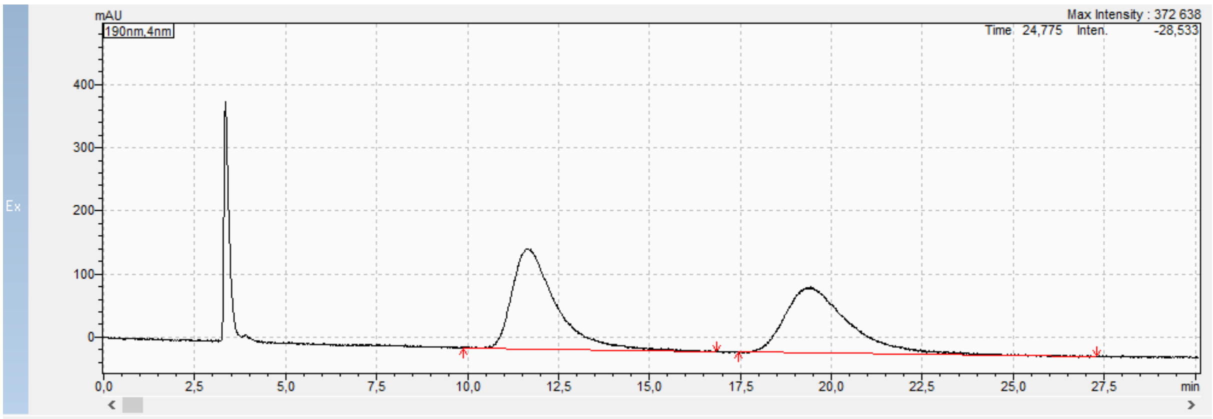

Results View - Peak Table

Peak Table | Compound | Group | Calibration Curve

Peak#	Ret. Time	Area	Height	Peak Start	Peak End	Mark	Conc.	Unit	Area%
1	11,681	13157065	158680	9,877	16,864	M	50,363		50,363
2	19,430	12967213	104906	17,440	27,307	M	49,637		49,637
Total		26124278	263586				100,000		100,000

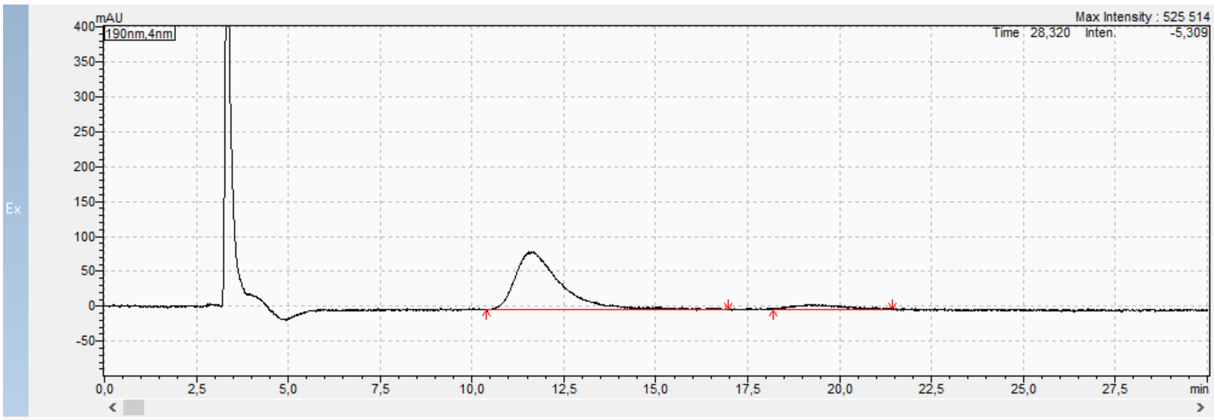

Results View - Peak Table

Peak Table | Compound | Group | Calibration Curve

Peak#	Ret. Time	Area	Height	Peak Start	Peak End	Mark	Conc.	Unit	Area%
1	11,604	7339608	83231	10,400	16,971	M	92,723		92,723
2	19,267	576051	6735	18,187	21,440	M	7,277		7,277
Total		7915658	89966				100,000		100,000

for **3q**: *er* = 93:7 *er* (*ee* = 86%)

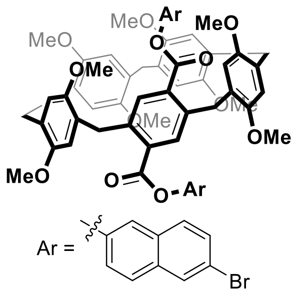

Conditions: Chiralpak IC column
mobile phase: *n*-heptane/propan-2-ol – 70:30
λ = 190 nm, V = 1.0 ml/min, t = 25 °C
for **3r**: t_R = 12.7 min (major), t_R = 21.7 min (minor).

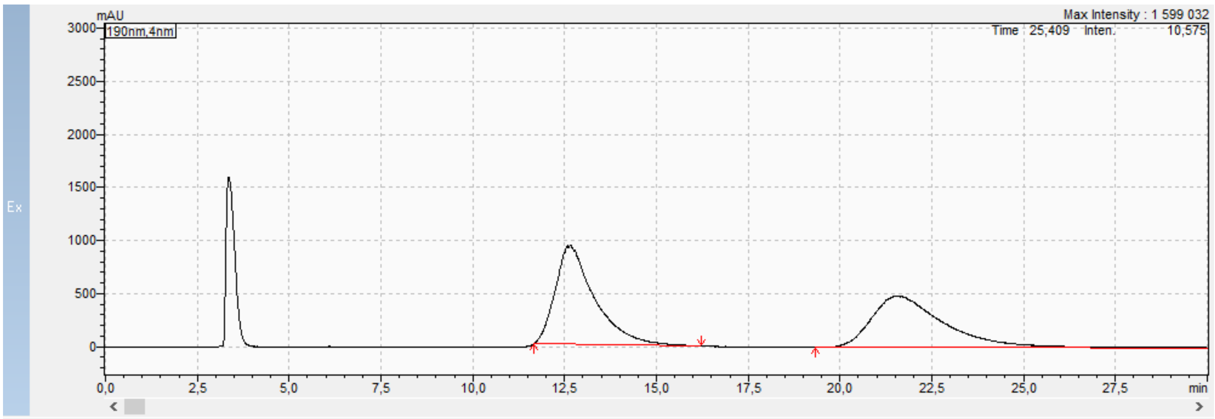

Results View - Peak Table

Peak Table | Compound | Group | Calibration Curve

Peak#	Ret. Time	Area	Height	Peak Start	Peak End	Mark	Conc.	Unit	Area%
1	12,642	68385397	932949	11,680	16,235	M	50,732		50,732
2	21,570	66412885	486168	19,328	34,251	M	49,268		49,268
Total		134798282	1419117				100,000		100,000

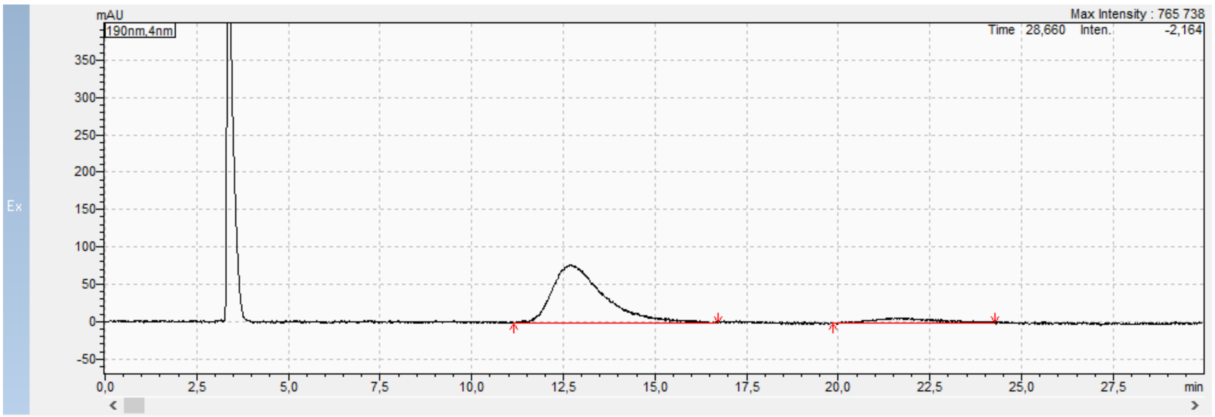

Results View - Peak Table

Peak Table | Compound | Group | Calibration Curve

Peak#	Ret. Time	Area	Height	Peak Start	Peak End	Mark	Conc.	Unit	Area%
1	12,673	7583043	78529	11,157	16,736	M	89,587		89,587
2	21,685	881413	7409	19,851	24,288	M	10,413		10,413
Total		8464456	85938				100,000		100,000

for **3r**: *er* = 90:10 (*ee* = 79%)

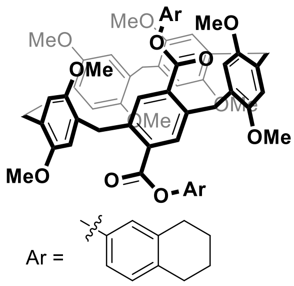

Conditions: Chiralpak IC column
mobile phase: *n*-heptane/propan-2-ol – 70:30
λ = 190 nm, V = 1.0 ml/min, t = 25 °C
for **3s**: t_R = 10.4 min (major), t_R = 17.5 min (minor).

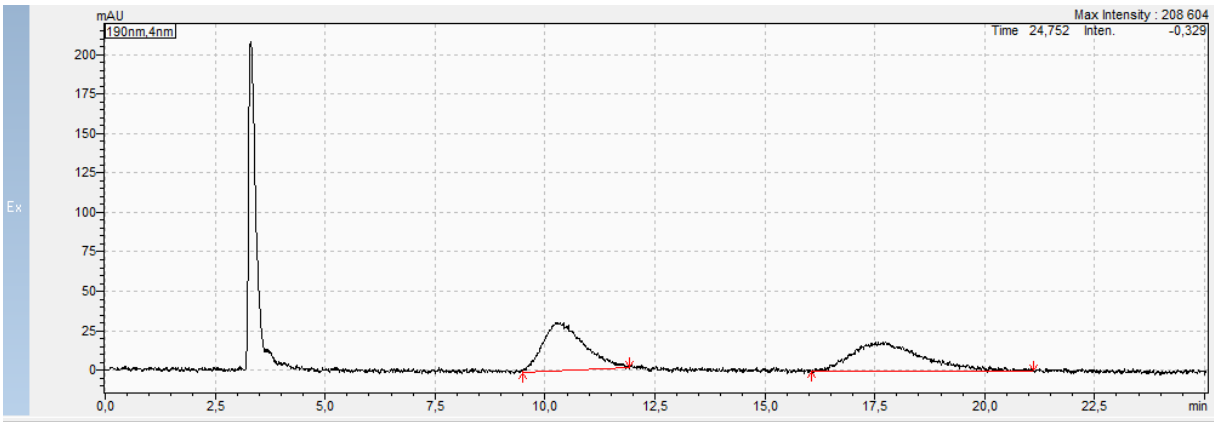

Results View - Peak Table

Peak Table | Compound | Group | Calibration Curve

Peak#	Ret. Time	Area	Height	Peak Start	Peak End	Mark	Conc.	Unit	Area%
1	10,259	1987435	30631	9,493	11,925	M	49,940		49,940
2	17,582	1992208	18267	16,064	21,109	M	50,060		50,060
Total		3979644	48898				100,000		100,000

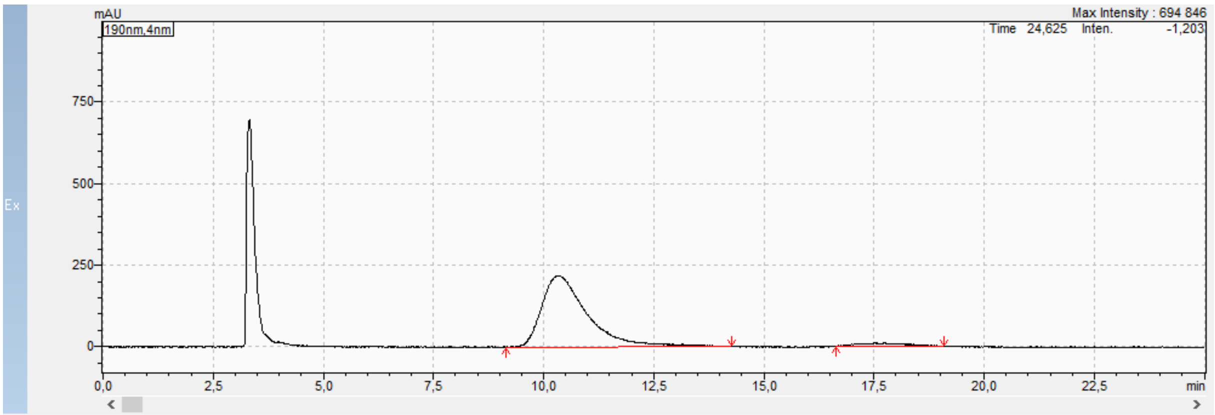

Results View - Peak Table

Peak Table | Compound | Group | Calibration Curve

Peak#	Ret. Time	Area	Height	Peak Start	Peak End	Mark	Conc.	Unit	Area%
1	10,366	15510957	218479	9,152	14,261	M	94,643		94,643
2	17,496	878037	11000	16,629	19,093	M	5,357		5,357
Total		16388994	229479				100,000		100,000

for **3s**: *er* = 95:5 (*ee* = 89%)

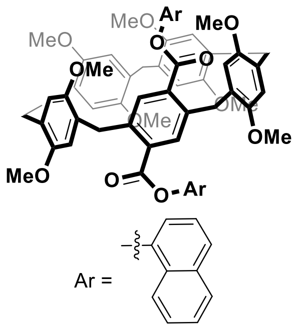

Conditions: Chiralpak IC column
mobile phase: *n*-heptane/propan-2-ol – 70:30
λ = 190 nm, V = 1.0 ml/min, t = 25 °C
for **3t**: t_R = 6.4 min (major), t_R = 9.0 min (minor).

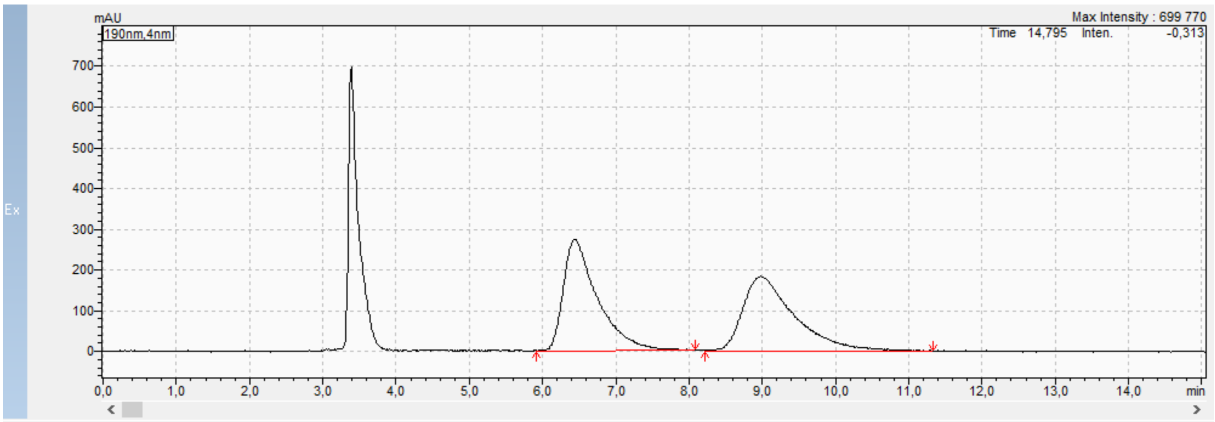

Results View - Peak Table

Peak Table | Compound | Group | Calibration Curve

Peak#	Ret. Time	Area	Height	Peak Start	Peak End	Mark	Conc.	Unit	Area%
1	6,436	8850355	273849	5,920	8,085	M	49,884		49,884
2	8,980	8891462	182455	8,213	11,328	M	50,116		50,116
Total		17741817	456304				100,000		100,000

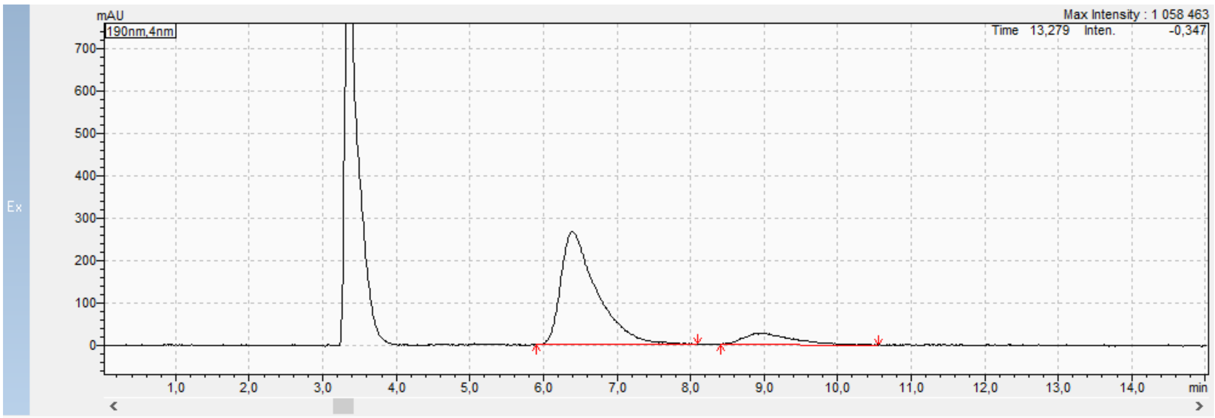

Results View - Peak Table

Peak Table | Compound | Group | Calibration Curve

Peak#	Ret. Time	Area	Height	Peak Start	Peak End	Mark	Conc.	Unit	Area%
1	6,386	8968069	265717	5,909	8,096	M	87,976		87,976
2	8,997	1225738	26565	8,405	10,549	M	12,024		12,024
Total		10193807	292282				100,000		100,000

for **3t**: *er* = 88:12 (*ee* = 76%)

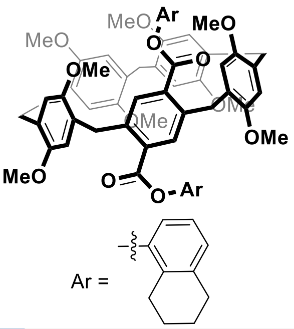

Conditions: Chiralpak IC column
mobile phase: *n*-heptane/propan-2-ol – 70:30
λ = 190 nm, V = 1.0 ml/min, t = 25 °C
for **3u**: t_R = 5.3 min (major), t_R = 7.4 min (minor).

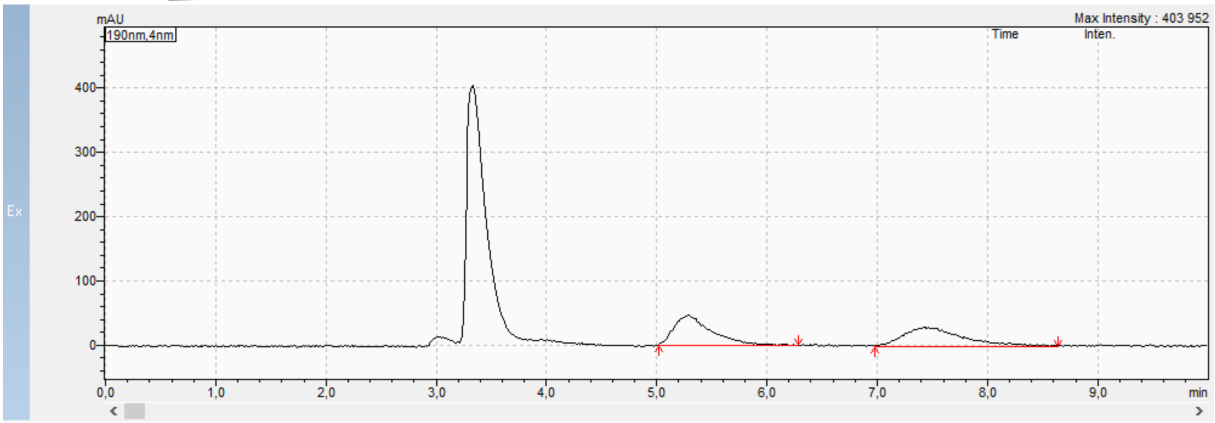

Results View - Peak Table

Peak Table | Compound | Group | Calibration Curve

Peak#	Ret. Time	Area	Height	Peak Start	Peak End	Mark	Conc.	Unit	Area%
1	5,284	1081342	46125	5,013	6,283	M	49,615		49,615
2	7,424	1098115	29012	6,976	8,640	M	50,385		50,385
Total		2179457	75137				100,000		100,000

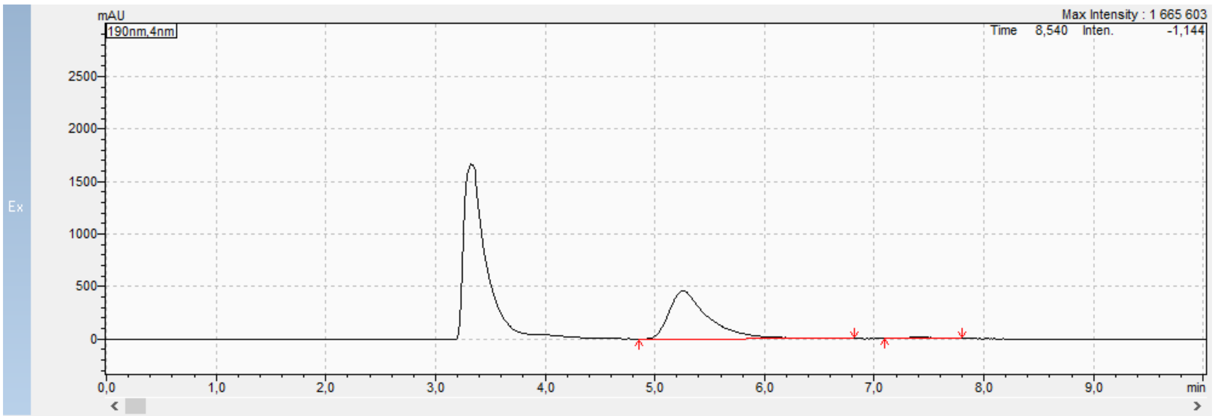

Results View - Peak Table

Peak Table | Compound | Group | Calibration Curve

Peak#	Ret. Time	Area	Height	Peak Start	Peak End	Mark	Conc.	Unit	Area%
1	5,250	11380933	454669	4,853	6,816	M	97,654		97,654
2	7,403	273440	12218	7,093	7,797	M	2,346		2,346
Total		11654373	466887				100,000		100,000

for **3u**: *er* = 98:2 (*ee* = 95%)

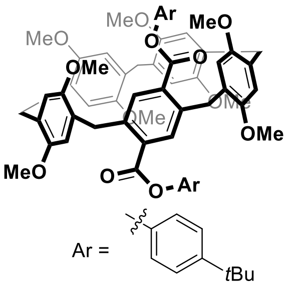

Conditions: Chiralpak IC column
mobile phase: *n*-heptane/propan-2-ol – 80:20
λ = 190 nm, V = 1.0 ml/min, t = 25 °C
for **4d**: t_R = 9.3 min (major), t_R = 12.6 min (minor).

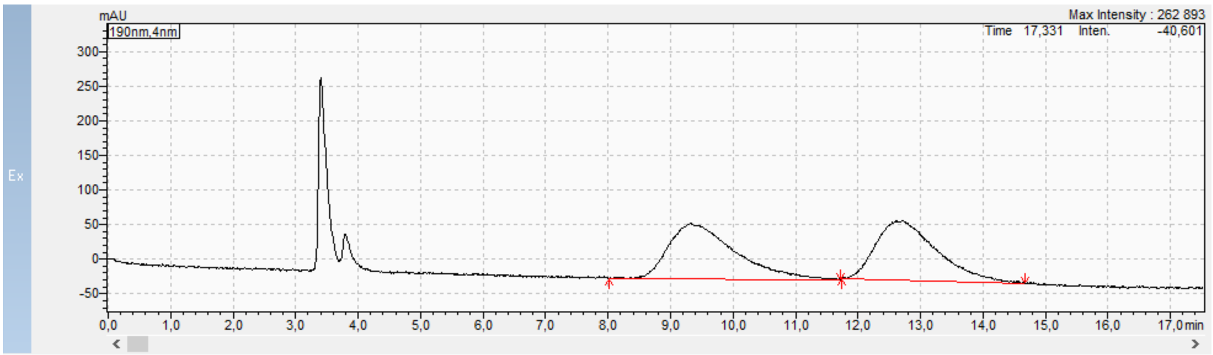

Results View - Peak Table

Peak Table | Compound | Group | Calibration Curve

Peak#	Ret. Time	Area	Height	Peak Start	Peak End	Mark	Conc.	Unit	Area%
1	9,325	5850051	80844	8,021	11,712	M	50,503		50,503
2	12,715	5733477	85447	11,733	14,677	M	49,497		49,497
Total		11583528	166291				100,000		100,000

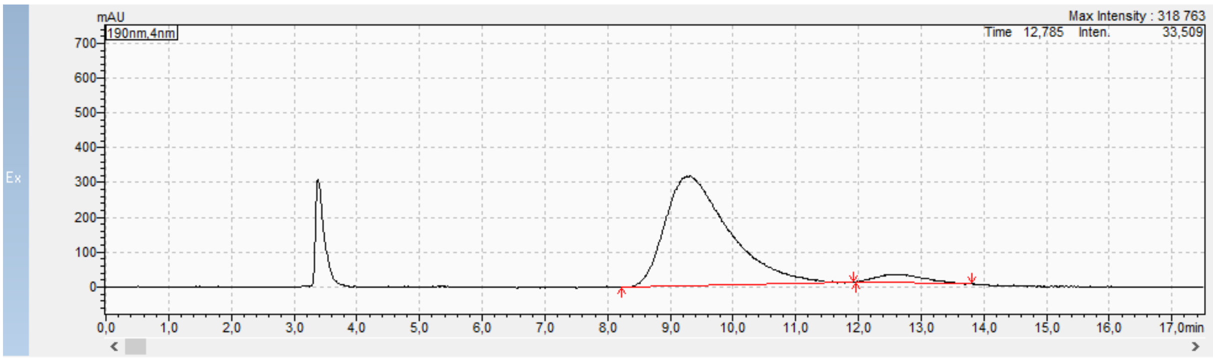

Results View - Peak Table

Peak Table | Compound | Group | Calibration Curve

Peak#	Ret. Time	Area	Height	Peak Start	Peak End	Mark	Conc.	Unit	Area%
1	9,314	21983222	314014	8,224	11,915	M	94,529		94,529
2	12,627	1272335	24574	11,957	13,803	M	5,471		5,471
Total		23255557	338588				100,000		100,000

for **4d**: *er* = 95:5 (*ee* = 89%)

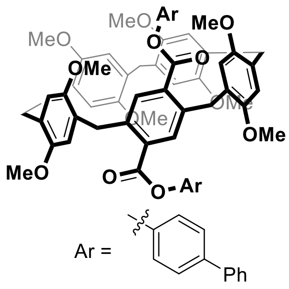

Conditions: Chiralpak IC column
mobile phase: *n*-heptane/propan-2-ol – 70:30
λ = 190 nm, V = 1.0 ml/min, t = 25 °C
for **4e**: t_R = 11.0 min (major), t_R = 20.4 min (minor).

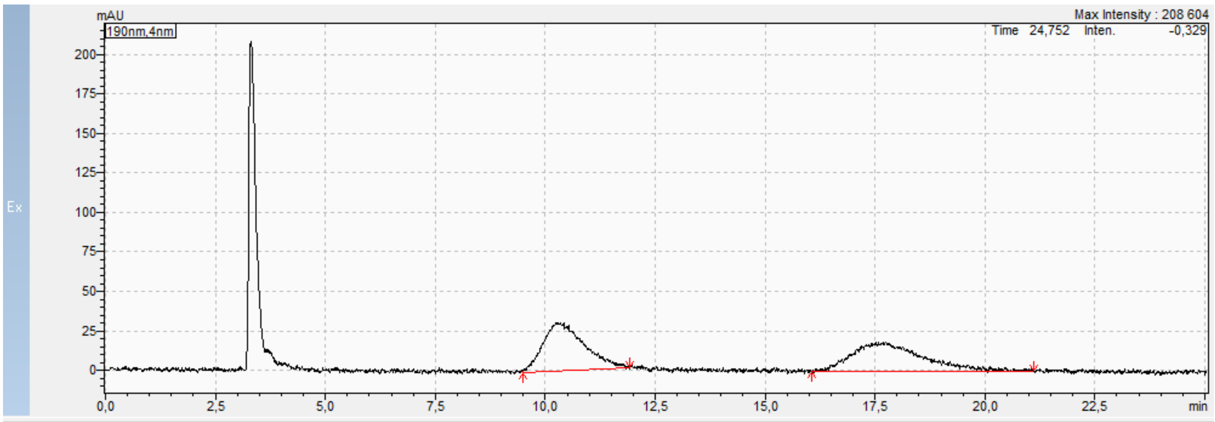

Results View - Peak Table

Peak Table | Compound | Group | Calibration Curve

Peak#	Ret. Time	Area	Height	Peak Start	Peak End	Mark	Conc.	Unit	Area%
1	10,259	1987435	30631	9,493	11,925	M	49,940		49,940
2	17,582	1992208	18267	16,064	21,109	M	50,060		50,060
Total		3979644	48898				100,000		100,000

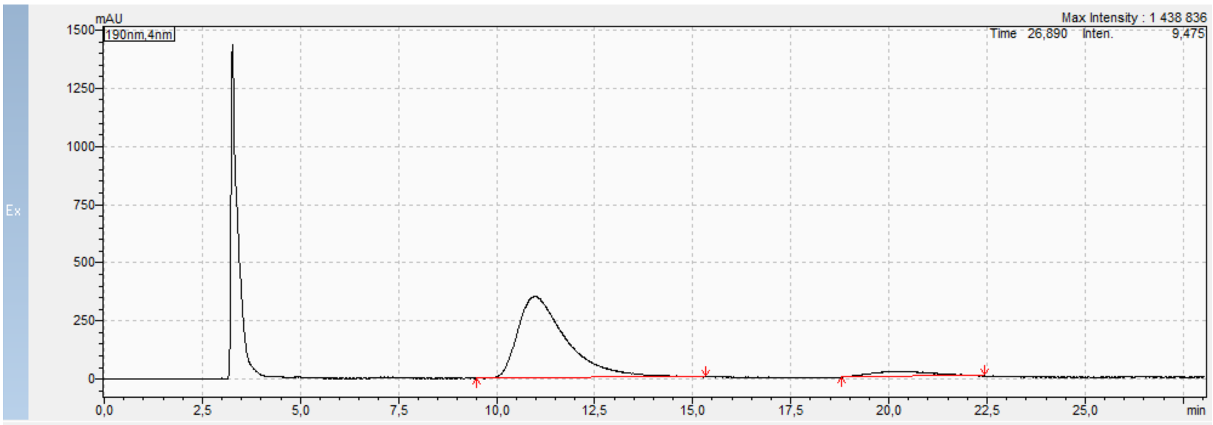

Results View - Peak Table

Peak Table | Compound | Group | Calibration Curve

Peak#	Ret. Time	Area	Height	Peak Start	Peak End	Mark	Conc.	Unit	Area%
1	10,988	30091683	352189	9,493	15,328	M	92,691		92,691
2	20,370	2372995	22418	18,795	22,443	M	7,309		7,309
Total		32464677	374607				100,000		100,000

for **4e**: *er* = 93:7 (*ee* = 86%)

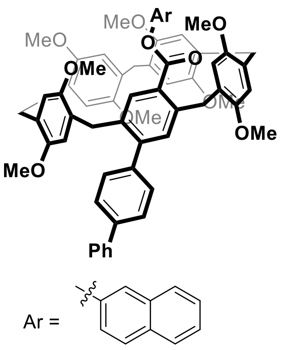

Conditions: Chiralpak IA column
mobile phase: *n*-heptane/propan-2-ol – 50:50
λ = 286 nm, V = 1.0 ml/min, t = 25 °C
for **5a**: t_R = 6.5 min (major), t_R = 11.2 min (minor).

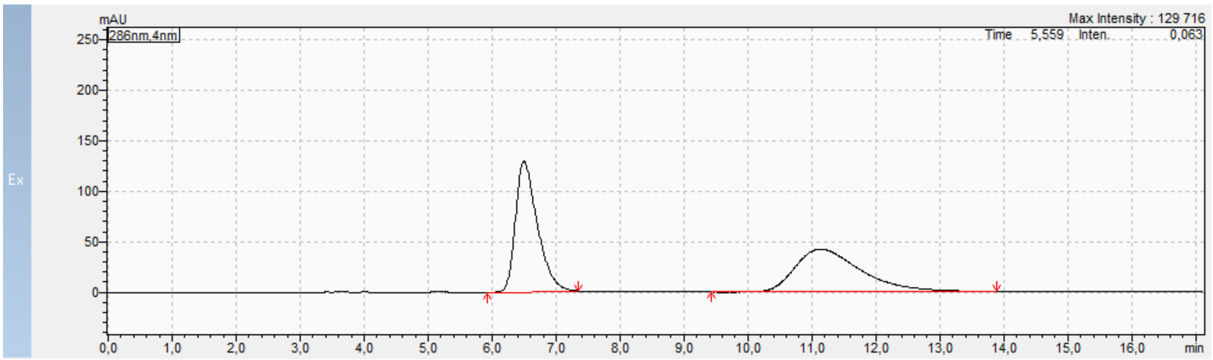

Results View - Peak Table

Peak Table | Compound | Group | Calibration Curve

Peak#	Ret. Time	Area	Height	Peak Start	Peak End	Mark	Conc.	Unit	Area%
1	6,501	3106926	129682	5,920	7,349	M	50,587		50,587
2	11,141	3034810	42306	9,429	13,888	M	49,413		49,413
Total		6141737	171988				100,000		100,000

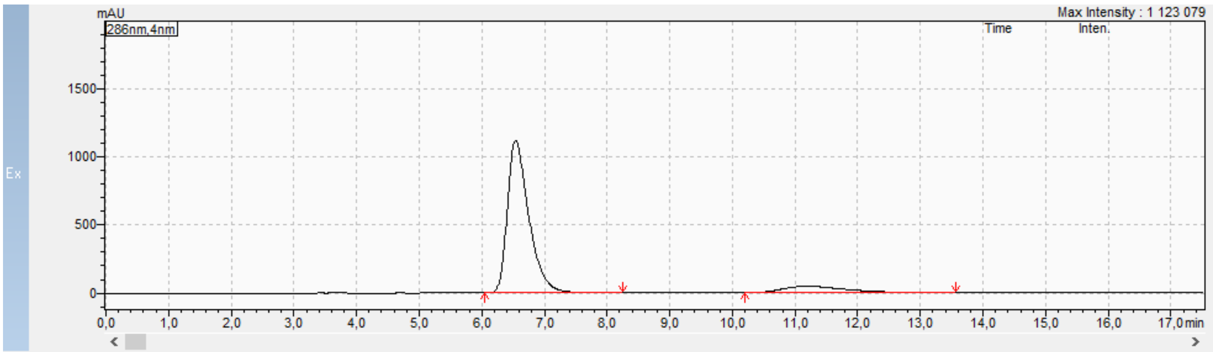

Results View - Peak Table

Peak Table | Compound | Group | Calibration Curve

Peak#	Ret. Time	Area	Height	Peak Start	Peak End	Mark	Conc.	Unit	Area%
1	6,542	26724341	1122124	6,048	8,256	M	88,682		88,682
2	11,190	3410518	48085	10,208	13,568	M	11,318		11,318
Total		30134859	1170210				100,000		100,000

for **5a**: *er* = 89:11 (*ee* = 77%)

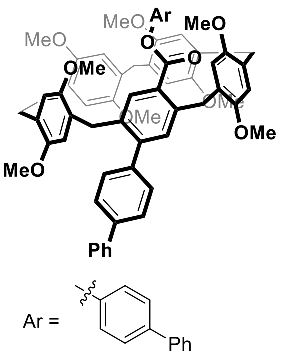

Conditions: Chiralpak IA column
mobile phase: *n*-heptane/propan-2-ol – 50:50
λ = 282 nm, V = 1.0 ml/min, t = 25 °C
for **5b**: t_R = 6.5 min (major), t_R = 14.0 min (minor).

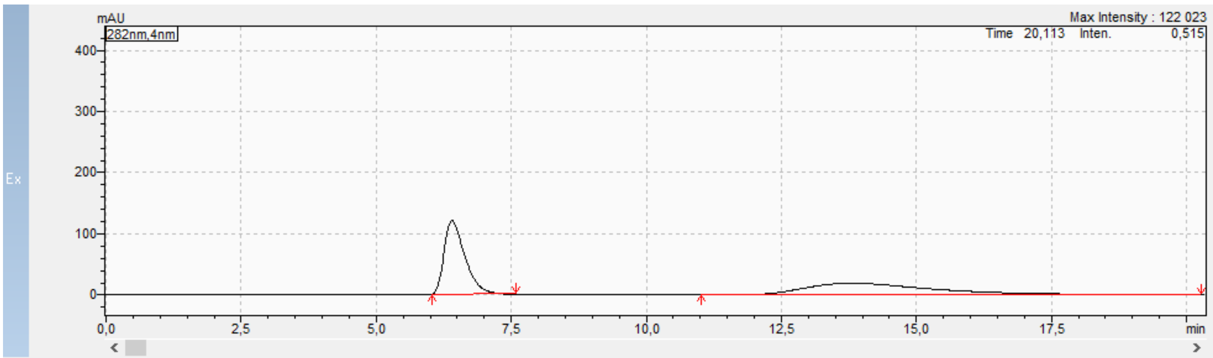

Results View - Peak Table

Peak Table | Compound | Group | Calibration Curve

Peak#	Ret. Time	Area	Height	Peak Start	Peak End	Mark	Conc.	Unit	Area%
1	6,403	3176900	120364	6,037	7,584	M	49,961		49,961
2	13,791	3181823	19076	11,019	20,277	M	50,039		50,039
Total		6358724	139439				100,000		100,000

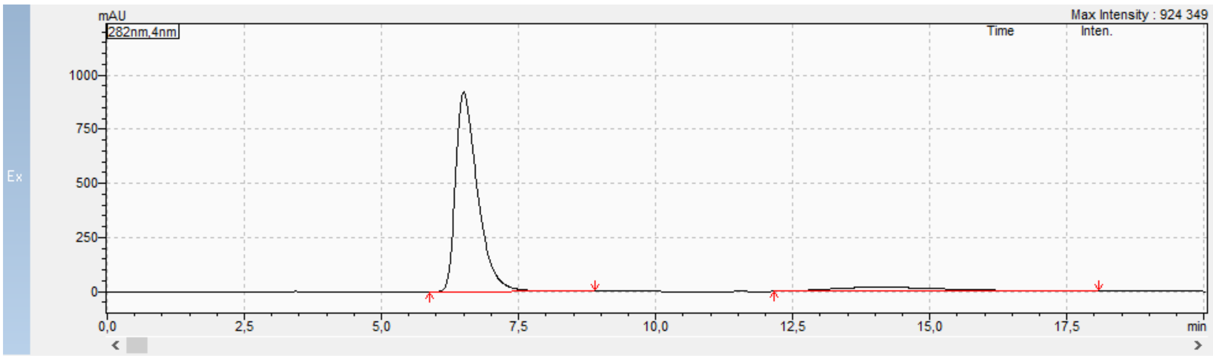

Results View - Peak Table

Peak Table | Compound | Group | Calibration Curve

Peak#	Ret. Time	Area	Height	Peak Start	Peak End	Mark	Conc.	Unit	Area%
1	6,499	25756269	923886	5,888	8,896	M	89,822		89,822
2	14,075	2918410	18739	12,160	18,091	M	10,178		10,178
Total		28674679	942625				100,000		100,000

for **5b**: *er* = 90:10 (*ee* = 80%)

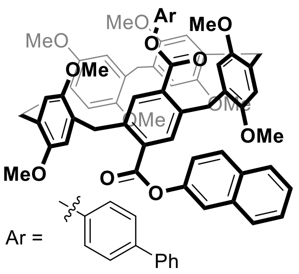

Conditions: Chiralpak IC column
mobile phase: *n*-heptane/propan-2-ol – 70:30
λ = 190 nm, V = 1.0 ml/min, t = 25 °C
for **5d**: t_R = 9.9 min (major), t_R = 16.3 min (minor).

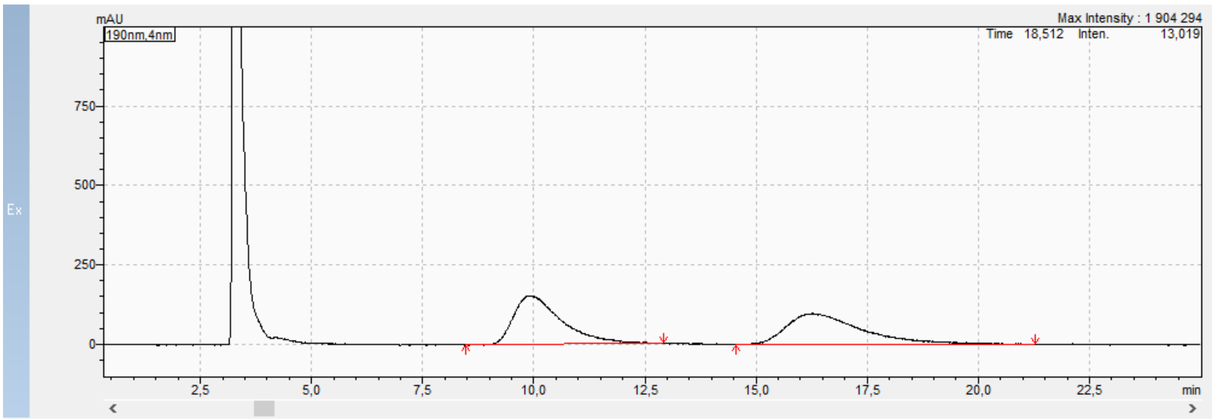

Results View - Peak Table

Peak Table | Compound | Group | Calibration Curve

Peak#	Ret. Time	Area	Height	Peak Start	Peak End	Mark	Conc.	Unit	Area%
1	9,901	11621175	153793	8,480	12,917	M	49,929		49,929
2	16,251	11654427	98582	14,539	21,269	M	50,071		50,071
Total		23275602	252375				100,000		100,000

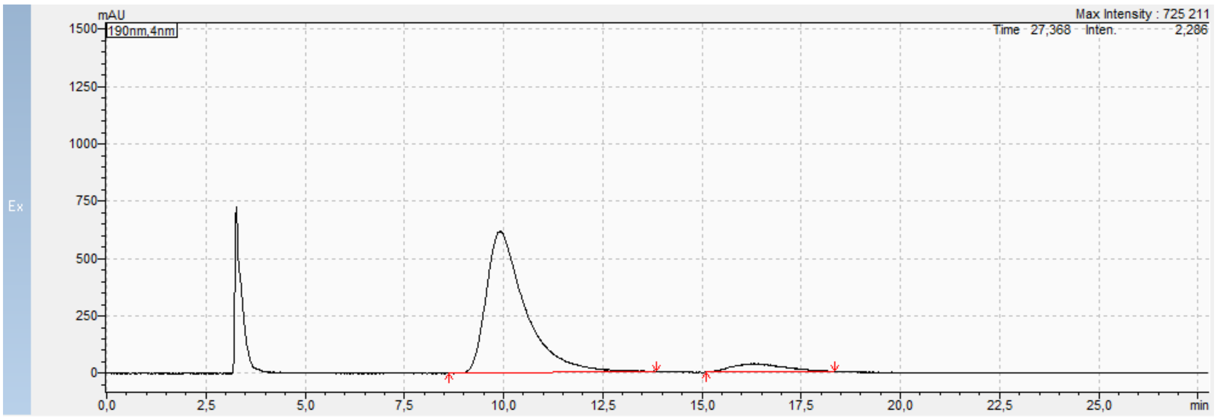

Results View - Peak Table

Peak Table | Compound | Group | Calibration Curve

Peak#	Ret. Time	Area	Height	Peak Start	Peak End	Mark	Conc.	Unit	Area%
1	9,922	42157540	618598	8,619	13,856	M	92,659		92,659
2	16,302	3339951	35902	15,104	18,347	M	7,341		7,341
Total		45497491	654500				100,000		100,000

for **5d**: *er* = 93:7 (*ee* = 85%)

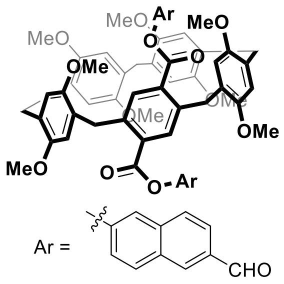

Conditions: Chiralpak IA column
mobile phase: *n*-heptane/propan-2-ol – 50:50
λ = 190 nm, V = 1.0 ml/min, t = 25 °C
for **6**: t_R = 12.6 min (major), t_R = 35.3 min (minor).

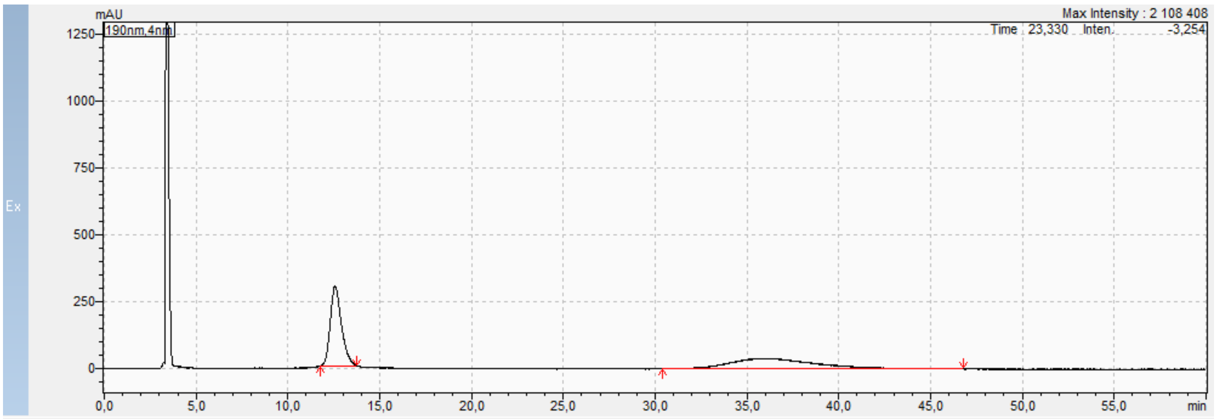

Results View - Peak Table

Peak Table | Compound | Group | Calibration Curve

Peak#	Ret. Time	Area	Height	Peak Start	Peak End	Mark	Conc.	Unit	Area%
1	12,570	12670678	299846	11,765	13,749	M	51,861		51,861
2	36,264	11761434	38108	30,400	46,795	M	48,139		48,139
Total		24432112	337954				100,000		100,000

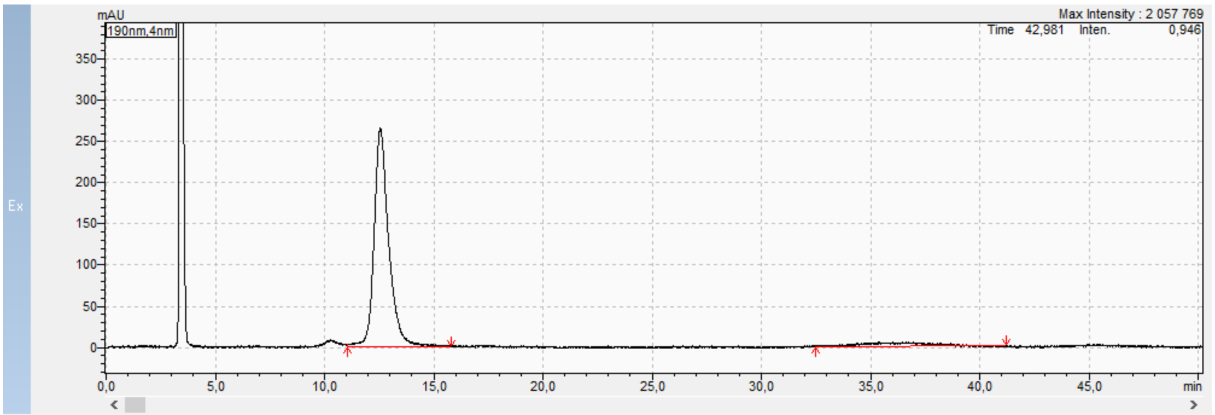

Results View - Peak Table

Peak Table | Compound | Group | Calibration Curve

Peak#	Ret. Time	Area	Height	Peak Start	Peak End	Mark	Conc.	Unit	Area%
1	12,566	12366845	265881	11,040	15,797	M	92,401		92,401
2	35,341	1017036	5083	32,512	41,237	M	7,599		7,599
Total		13383881	270964				100,000		100,000

for **6**: *er* = 92:8 (*ee* = 85%)

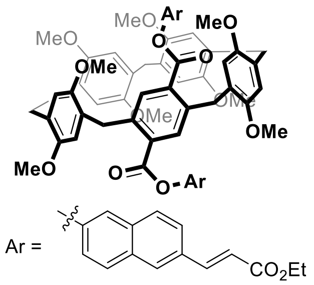

Conditions: Chiralpak IA column
mobile phase: *n*-heptane/propan-2-ol – 50:50
λ = 190 nm, V = 1.0 ml/min, t = 25 °C
for **7**: t_R = 20.0 min (major), t_R = 24.4 min (minor).

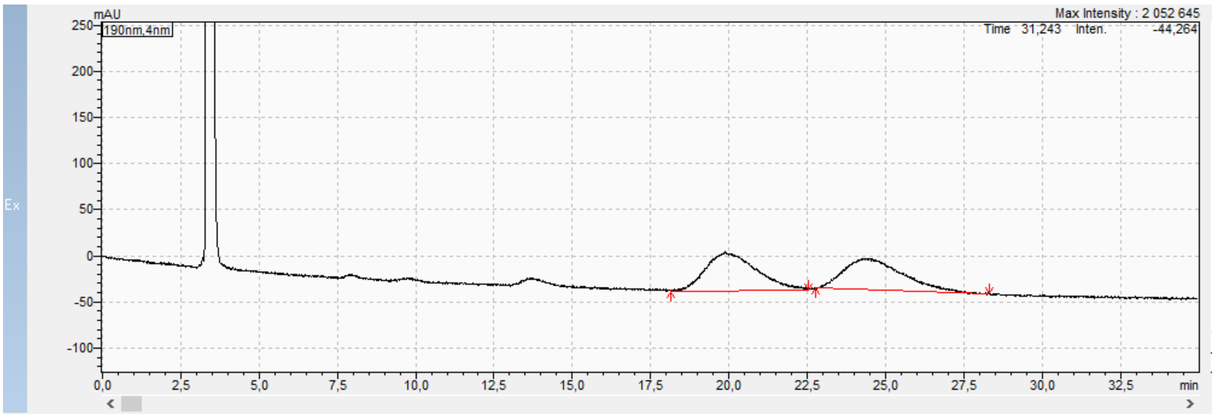

Results View - Peak Table

Peak Table | Compound | Group | Calibration Curve

Peak#	Ret. Time	Area	Height	Peak Start	Peak End	Mark	Conc.	Unit	Area%
1	19,885	4608967	42007	18,133	22,539	M	51,334		51,334
2	24,442	4369378	34030	22,763	28,320	M	48,666		48,666
Total		8978345	76037				100,000		100,000

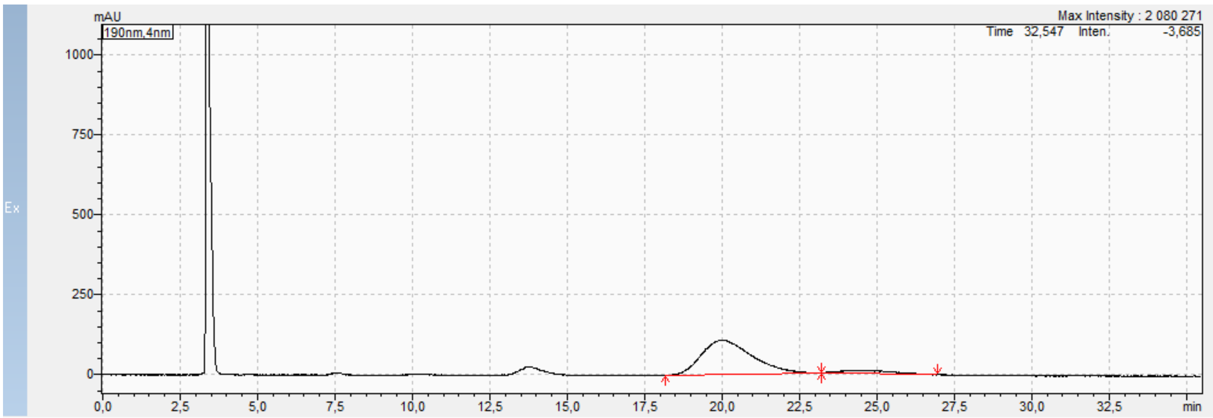

Results View - Peak Table

Peak Table | Compound | Group | Calibration Curve

Peak#	Ret. Time	Area	Height	Peak Start	Peak End	Mark	Conc.	Unit	Area%
1	20,011	12260536	107738	18,165	23,211	M	90,787		90,787
2	24,391	1244213	11190	23,211	26,965	M	9,213		9,213
Total		13504749	118927				100,000		100,000

for **7**: *er* = 91:9 (*ee* = 82%)

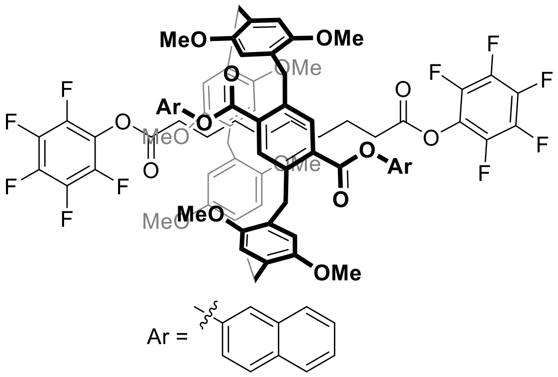

Conditions: Chiralpak IA column
mobile phase: *n*-heptane/propan-2-ol – 80:20
λ = 190 nm, V = 1.0 ml/min, t = 25 °C
for **8**: t_R = 4.9 min (major), t_R = 6.2 min (minor).

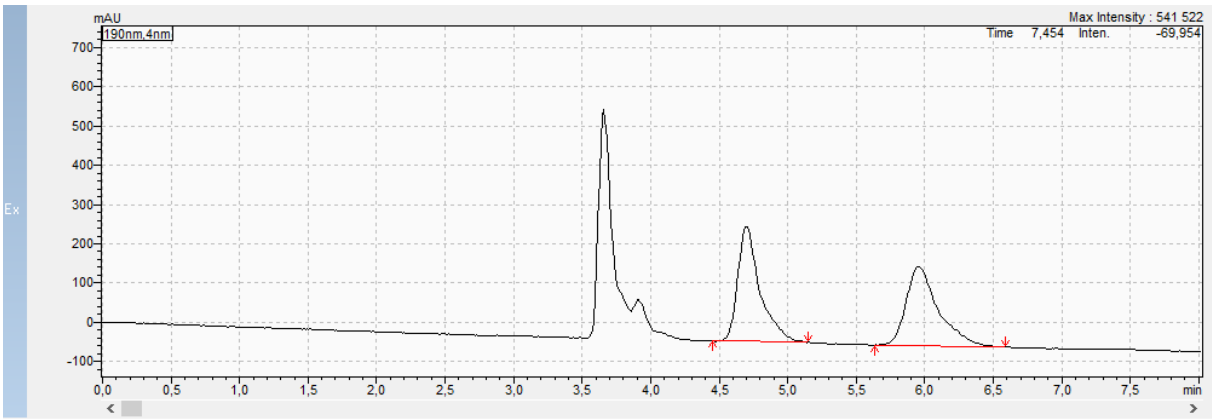

Results View - Peak Table

Peak Table | Compound | Group | Calibration Curve

Peak#	Ret. Time	Area	Height	Peak Start	Peak End	Mark	Conc.	Unit	Area%
1	4,699	3307019	292199	4,448	5,152	M	50,039		50,039
2	5,954	3301827	202547	5,632	6,592	M	49,961		49,961
Total		6608846	494745				100,000		100,000

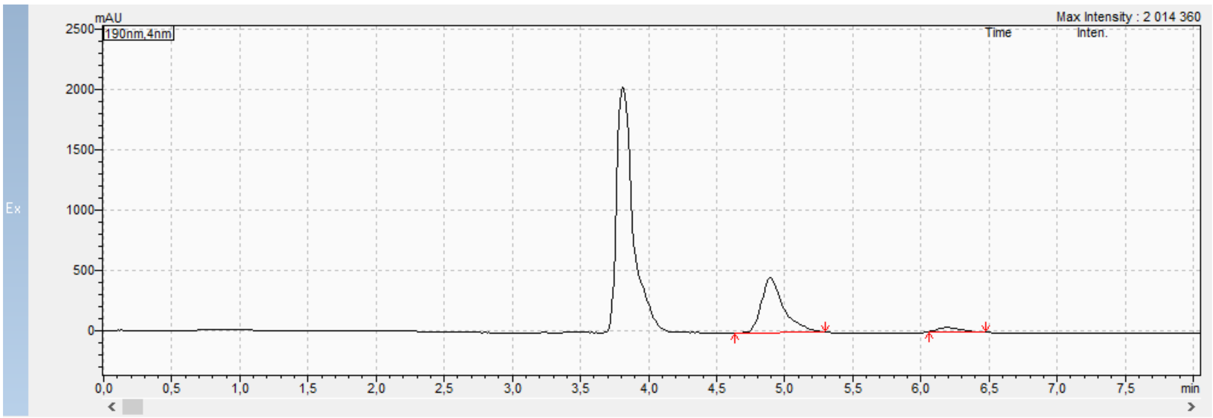

Results View - Peak Table

Peak Table | Compound | Group | Calibration Curve

Peak#	Ret. Time	Area	Height	Peak Start	Peak End	Mark	Conc.	Unit	Area%
1	4,896	5114731	452640	4,629	5,301	M	92,401		92,401
2	6,190	420643	36014	6,059	6,475	M	7,599		7,599
Total		5535374	488653				100,000		100,000

for **8**: *er* = 92:8 (*ee* = 85%)

References

1 Boinski, T., & Szumna A. A facile, moisture-insensitive method for synthesis of pillar[5]arenes-the solvent templation by halogen bonds. *Tetrahedron* **68**, 9419–9422 (2012).

2 Wang, G., Qiang, H., Guo, Y. Z., Yang, J., Wen, K. & Hu, W. B. Systematic Rim Cyano-Functionalization of Pillar[5]Arene and Corresponding Host-Guest Property Varieties. *Org. Biomol. Chem.* **17**, 4600–4604 (2019).

3 Luan, T. R., Sun, C., Le Tian, Y., Jiang, Y. K., Xi, L. L. & Liu, R. R. Enantioselective Construction of Inherently Chiral Pillar[5]Arenes via Palladium-Catalysed Suzuki–Miyaura Cross-Coupling. *Nat. Commun.* **16**, 2370 (2025).

4 a) Romanov-Michailidis, F., Besnard, C. & Alexakis, A. *N*-Heterocyclic Carbene-Catalyzed Annulation of α-Cyano-1,4-diketones with Ynals. *Org. Lett.* **14**, 4906–4909 (2012); for *pre*-**C1**. b) Schmidt, V. A. & Alexanian, E. J. Metal-Free, Aerobic Dioxygenation of Alkenes Using Hydroxamic Acids. *Angew. Chem. Int. Ed.* **49**, 4491–4494 (2010); for *pre*-**C6**.

5 Lian, Y., Struble, J. R. & Bode, J. W. Synthesis of a N-mesityl substituted aminoindanol-derived triazolium salt [(5a*S*,10b*R*)-5a,10b-Dihydro-2-(2,4,6-trimethylphenyl)-4*H*,6*H*-indeno[2,1-b]-1,2,4-triazolo[4,3-d]-1,4-oxazinium chloride]. *Org. Synth.* **87**, 362 (2010).

6 Kerr, M. S., Read de Alaniz, J. & Rovis, T. An Efficient Synthesis of Achiral and Chiral 1,2,4-Triazolium Salts: Bench Stable Precursors for *N*-Heterocyclic Carbenes. *J. Org. Chem.* **70**, 5725–5728 (2005).

7 Kreituss, I., Murakami, Y., Binanzer, M. & Bode, J. W. Kinetic Resolution of Nitrogen Heterocycles with a Reusable Polymer-Supported Reagent. *Angew. Chem. Int. Ed.* **51**, 10660–10663 (2012).

8 Liu, Y., Luo, G., Yang, X., Jiang, S., Xue, W., Chi, Y. R. & Jin, Z. Carbene-Catalyzed Enantioselective Aromatic N-Nucleophilic Addition of Heteroarenes to Ketones. *Angew. Chem. Int. Ed.* **59**, 442–448 (2020).

9 Xiao, Y., Zhao, Z.-Y., Irran, E. & Oestreich, M. Enantio- and Diastereoselective Desymmetrization of 1,1’-Biaryl-2,6-Dicarbaldehydes by Copper-Catalyzed 1,2-Addition of Silicon Nucleophiles. *Angew. Chem. Int. Ed.* **63**, e202414005 (2024)

10 Zhao, C., Li, F. & Wang, J. *N*-Heterocyclic Carbene Catalyzed Dynamic Kinetic Resolution of Pyranones. *Angew. Chem. Int. Ed.* **55**, 1820–1824 (2016).

11 Li, F., Wu, Z. & Wang, J. Oxidative Enantioselective α-Fluorination of Aliphatic Aldehydes Enabled by N-Heterocyclic Carbene Catalysis. *Angew. Chem. Int. Ed.* **54**, 656–659 (2015).

12 Allen, S. E., Mahatthananchai, J., Bode, J. W. & Kozlowski, M. C. Oxyanion Steering and CH−π Interactions as Key Elements in an N-Heterocyclic Carbene-Catalyzed [4 + 2] Cycloaddition. *J. Am. Chem. Soc.* **134**, 12098–12103 (2012).

13 Liu, L., Guo, D. & Wang, J. NHC-Catalyzed Asymmetric α-Regioselective [4 + 2] Annulation to Construct α-Alkylidene-δ-lactones. *Org. Lett.* **22**, 7025–7029 (2020).

14 Concellón, C., Duguet, N. & Smith, A. D. N-Heterocyclic Carbene-Mediated Enantioselective Addition of Phenols to Unsymmetrical Alkylarylketenes. *Adv. Synth. Catal.* **351**, 3001–3009 (2009).

15 Goodman, C. G., Walker, M. M., & Johnson, J. S. Enantioconvergent Synthesis of Functionalized γ-Butyrolactones via (3 + 2)-Annulation. *J. Am. Chem. Soc.* **137**, 122–125 (2015).

16 Campbell, C. D., Concellón, C. & Smith, A. D. Catalytic enantioselective Steglich rearrangements using chiral N-heterocyclic carbenes. *Tetrahedron Asym.* **22**, 797–811 (2011).

17 Huang, X.-L., He, L., Shao, P.-L. & Ye, S. [4+2] Cycloaddition of Ketenes with *N*-Benzoyldiazenes Catalyzed by *N*-Heterocyclic Carbenes. *Angew. Chem. Int. Ed.* **48**, 192–195 (2008).
18 Brand, J. P., Siles, J. I. O., & Waser, J. Synthesis of Chiral Bifunctional (Thio)Urea N-Heterocyclic Carbenes. *Synlett* **6**, 881–884 (2010)
19 Li, E., Chen, J. & Huang, Y. Enantioselective Seleno-Michael Addition Reactions Catalyzed by a Chiral Bifunctional N-Heterocyclic Carbene with Noncovalent Activation. *Angew. Chem. Int. Ed.* **61**, e202202040 (2022).
20 Barik, S., Ranganathappa, S. S. & Biju, A. T. *N*-heterocyclic carbene-catalyzed atroposelective synthesis of *N*-Aryl phthalimides and maleimides via activation of carboxylic acids. *Nat. Commun.* **15**, 5755 (2024).
21 Sheldrick, G. M. SHELXT-Integrated Space-Group and Crystal-Structure Determination. *Acta Crystallogr. Sect. A Found. Crystallogr.* **A71**, 3–8 (2015).
22 Sheldrick, G. M. Crystal structure refinement with SHELXL. *Acta Crystallogr. Sect. C Struct. Chem.* **C71**, 3–8 (2015).
23 Parsons, S., Flack, H. D. & Wagner, T. Use of intensity quotients and differences in absolute structure refinement. *Acta Crystallogr. Sect. B Struct. Sci. Cryst. Eng. Mater.* **B69**, 249–259 (2013).
